# Sulfonylation of RNA 2′-OH groups

**DOI:** 10.1021/acscentsci.2c01237

**Published:** 2023-03-01

**Authors:** Sayantan Chatterjee, Ryuta Shioi, Eric T. Kool

**Affiliations:** Department of Chemistry, Stanford University, Stanford, California 94305, United States

## Abstract

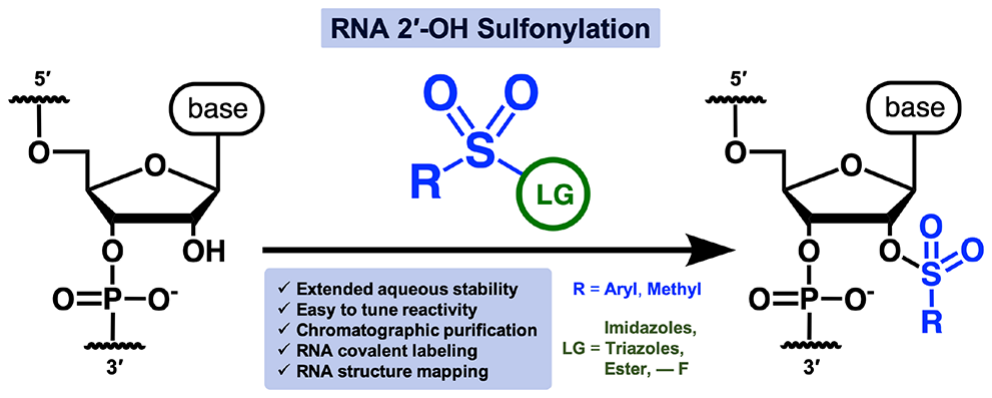

The nucleophilic reactivity of RNA 2′-OH groups in water has proven broadly useful in probing, labeling, and conjugating RNA. To date, reactions selective to ribose 2′-OH have been limited to bond formation with short-lived carbonyl electrophiles. Here we report that many activated small-molecule sulfonyl species can exhibit extended lifetimes in water and retain 2′-OH reactivity. The data establish favorable aqueous solubility for selected reagents and successful RNA-selective reactions at stoichiometric and superstoichiometric yields, particularly for aryl sulfonyltriazole species. We report that the latter are considerably more stable than most prior carbon electrophiles in aqueous environments and tolerate silica chromatography. Furthermore, an azide-substituted sulfonyltriazole reagent is developed to introduce labels into RNA via click chemistry. In addition to high-yield reactions, we find that RNA sulfonylation can also be performed under conditions that give trace yields necessary for structure mapping. Like acylation, the reaction occurs with selectivity for unpaired nucleotides over those in the duplex structure, and a sulfonate adduct causes reverse transcriptase stops, suggesting potential use in RNA structure analysis. Probing of rRNA is demonstrated in human cells, indicating possible cell permeability. The sulfonyl reagent class enables a new level of control, selectivity, versatility, and ease of preparation for RNA applications.

## Abstract 2

We introduce sulfonyl reagents as a new class of RNA 2′-OH reactive small molecules. These molecules are stable, easy to synthesize and purify, and useful for structure mapping and covalent labeling.

## Introduction

RNA acylation at 2′-OH groups has emerged as a broadly useful reaction strategy for multiple applications in RNA and transcriptome research.^1,2^ Methods were initially developed for mapping RNA structure, using trace levels of reaction that selectively occurs at unpaired regions relative to duplex regions in folded RNAs;^3,4^ these methods are widely applied both *in vitro* and in living cells.^5−8^ One of the most appealing features of this reactivity is its ability to modify and interrogate RNA at nearly every position, which is unlike other covalent RNA-probing reactions such as alkylation of adenine and cytosine bases with dimethylsulfate^9^ and cross-linking of pyrimidines with psoralens.^10^

It is noteworthy that until recently, only one class of small-molecule electrophile—the activated carbonyl group—has been reported for 2′-OH groups in RNA. Limitations of known RNA-reactive carbonyl species include modest water solubility, rapid hydrolysis limiting yields and requiring elevated concentrations, and restricted strategies for preparation due to a lack of stability to column chromatography.^7,8,11^ In contrast to RNA, a wide range of electrophiles are documented to react successfully with protein side chains,^12−17^ leading to the question of whether other reactive groups beyond C=O are possible for the 2′-hydroxyl of RNA molecules.

In particular, activated sulfonyl groups have proven highly useful recently in protein profiling, showing reactivity at tyrosine and other side chains in water.^18−20^ Encouragingly, a recent study has also reported that RNA-binding proteins that are genetically encoded to possess fluorosulfate-containing modified tyrosine residues can direct the formation of sulfate ester linkages at RNA 2′-OH.^21^ Following these precedents, we undertook a study of the potential for small-molecule sulfonylation in RNA. Our chief motivations were (1) finding reactive motifs beyond modified sulfur groups on proteins, which may have different reactivities than small alkyl/aryl sulfonyl compounds due to the macromolecular context; (2) discovering strategies to tune RNA reactivity of small-molecule sulfonylating reagents and establishing the scope of effective structures; and (3) investigating the potential for further application in RNA chemical biology research (*e.g*., RNA conjugation and structure analysis). Importantly, to establish a general approach with wide applicability, we tested small-molecule sulfonyl reagents whose reactivity would be independent of any RNA-macromolecular interactions, to reduce the possibility of reactions occurring by proximity effects and to remove the requirement for genetic encoding.^21^ Given the literature examples and our motivation, the oxophilic nature of sulfur and the hydrophilicity of sulfonyl groups, we wondered whether there might exist a small-molecule sulfonylating reagent structure that might enable aqueous reactivity with 2′-OH groups in RNA.

Desirable features for RNA 2′-OH-reactive reagents include sufficient reactivity to functionalize this relatively bulky secondary hydroxyl in high yields, selectivity for 2′-OH groups in unpaired structure over paired contexts, adequate lifetime and solubility in water to remain available for RNA, cell permeability for *in vivo* application, ease of synthesis and purification, versatility of structural modification, and tunability of electrophilicity. Considering the versatility of known reactive sulfonyl reagents, it seemed possible that many of these features might be found in carefully designed electrophilic sulfur species.

With these issues in mind, we have undertaken a study of a series of sulfur-based electrophilic reagents with the potential for use in functionalizing or mapping this nucleic acid. Here we report that sulfonyl species with carefully tuned electrophilicity and leaving groups can react successfully in high yields with the 2′-OH groups of RNA. Notably, they can be prepared readily from sulfonyl chlorides and purified easily via silica column chromatography. We explore the scope of varied reagent structure that can be incorporated into RNA via conjugation reactions. We further document a postsulfonylation conjugation reaction made possible by an azide-functionalized sulfonate reagent. Finally, we show that sulfonylation reactions applied in trace yields can potentially be applied to RNA structure mapping as well, demonstrating selectivity for unpaired nucleotides over those in a duplex structure. The results establish a new and versatile reagent class for RNA research.

## Results and Discussion

### Reagent Design and Initial Tests

**Figure 1:**
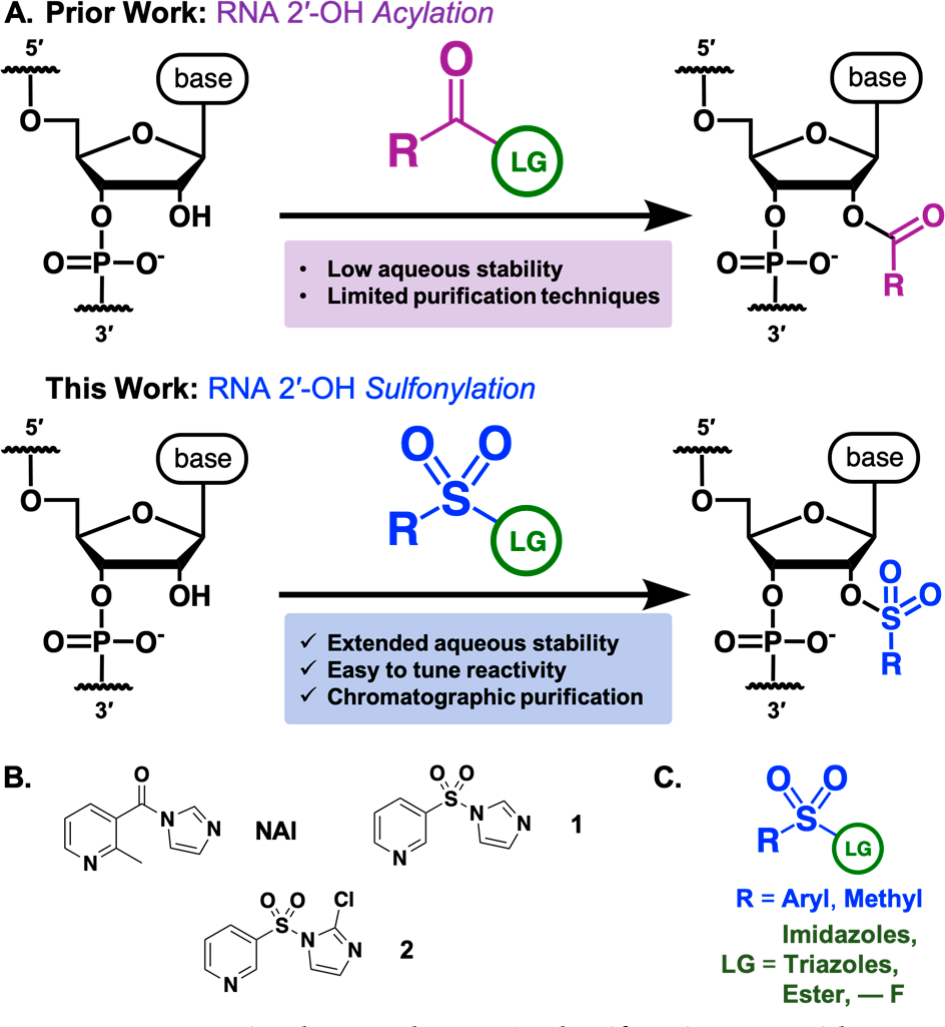
(**A**) Schematic comparison of prior methods of RNA 2′-OH covalent modification using small molecules with this work. (**B**) Structures of NAI (commonly used acylimidazole reagent), molecules **1** and **2** (used for initial tests). (**C**) General scaffold for RNA 2′-OH sulfonylating reagents.

We investigated the possibility of sulfonylation at RNA 2′-OH inspired by many recent literature reports of the utility of activated sulfonyl species in chemical biology applications such as protein profiling.^18,22,23^ A recent study of tyrosine side chain phenolic modification in aqueous environments with sulfonyl triazoles confirmed good yields,^18^ and given the relative acidity of 2′-OH in RNA (p*K*_a_ ≈ 12.5),^24^ a similar reaction in this distinct biopolymer seemed plausible. To begin our investigations, we prepared the pyridyl imidazole compounds **1** and **2**, inspired by the structure of the earlier nicotinyl acylimidazole compound NAI^7^ (Figure 1B), from commercially available pyridine-3-sulfonyl chloride (**8**, Table 1) via a simple one-step procedure with standard silica gel chromatographic purification (see Supporting Information (SI)). We found that under standard RNA reaction conditions (RNA 10 μM, reagent 100 mM in pH 7.5 buffer ([MOPS] = [NaCl] = 100 mM, [MgCl_2_] = 6 mM) with 20% DMSO, 24 h, 37 °C) both of these compounds demonstrated significant RNA 2′-OH reactions detectable by MALDI-TOF mass spectroscopy with a short 16 nt single-stranded RNA (Supporting Information; Figure S2, Figure S3). With **1**, a modest 6.4% of the test RNA strand underwent modification with a pyridine-3-sulfonyl group, presumably generating an O-sulfonate ester (see below for confirmation). Encouragingly, with the stronger 2-chloroimidazole leaving group (**2**) we observed 54% conversion of the test RNA strand (Figure S3). Similar levels of modification were also detected upon reaction of these molecules with other short RNA sequences and at varying DMSO composition percentages over 10–50% (Figure S2, Figure S3). Importantly, MALDI-TOF analysis of parallel reactions of **1** and **2** with a DNA strand having the same sequence as the test RNA gave no reaction, confirming that the RNA adducts occurred at 2′-OH groups rather than exocyclic amines (Figure S2, Figure S3). Based on these initial findings, we adopted a general scaffold for testing RNA 2′-OH sulfonylating agents, with a heteroaryl group linked to a leaving group through the sulfonyl moiety (Figure 1C) and investigated whether further improvements in yield could be achieved.

**Table 1:**
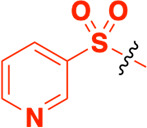

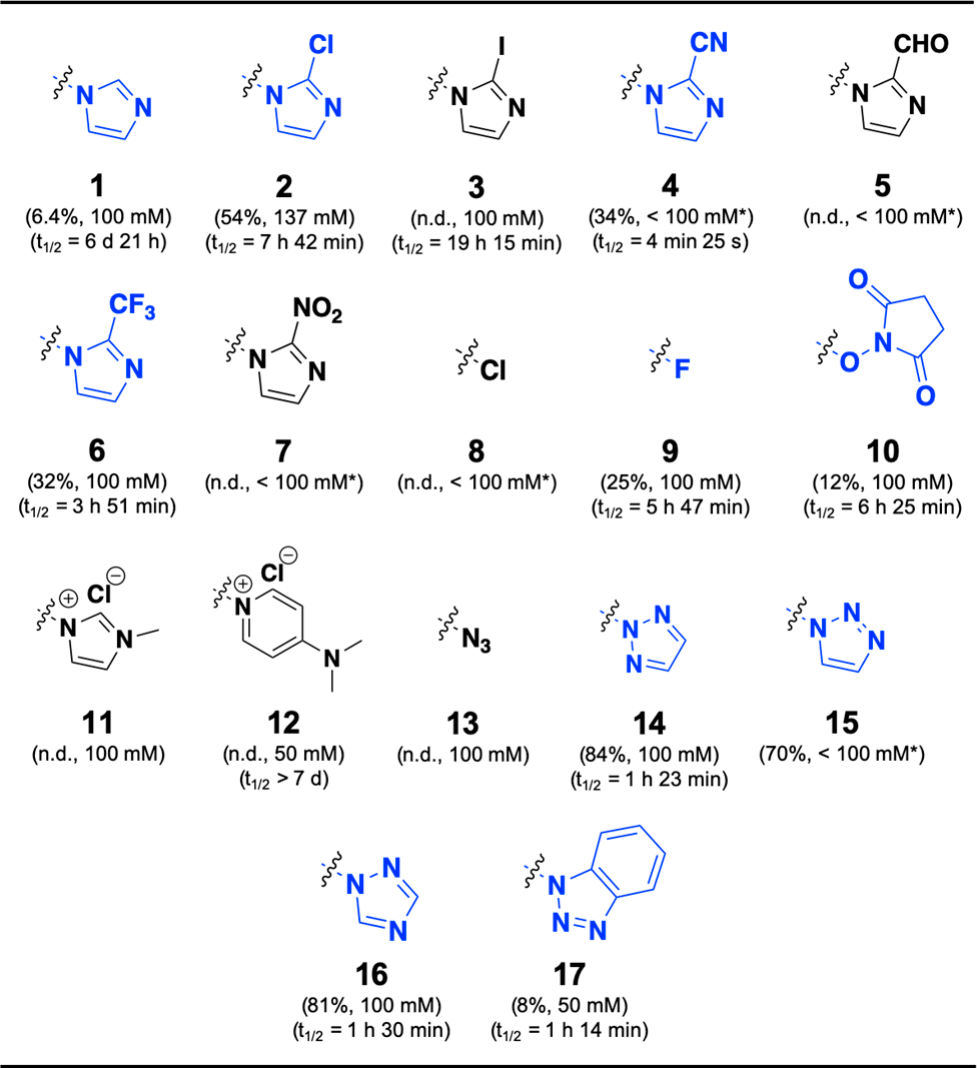
Scope of Leaving Groups for RNA 2′-OH Sulfonylation^a^

a Scope of leaving groups for RNA 2′-OH modification using pyridine-3-sulfonyl scaffold (in red). Leaving groups in blue showed detectable levels (by MALDI-TOF MS) of RNA 2′-OH sulfonylation. In parentheses: conversion yield (in %) estimated by MALDI-TOF MS after ethanol precipitation (refers to percentage of all RNA after reaction that has at least one 2′-OH covalently modified by sulfonylating agent) (n.d. denotes no conversion observed); reagent concentration (in mM) used in RNA reactions with Test RNA (*: molecule unstable in DMSO stock solution). *t*_1/2_ (hydrolysis half-life) measured by ^1^H NMR (Supporting Information). Sulfonylation reactions performed with 16-mer Test RNA, reaction volume 10 μL, 20% DMSO, reaction time 24 h, temperature 37 °C (detailed experimental procedures in Supporting Information).

### Scope of Leaving Groups

The significant difference observed in sulfonylation yields between imidazole vs the less basic 2-chloroimidazole leaving group led us to hypothesize that the yields might be tuned by simply changing the leaving groups, with the expectation that greater electron-withdrawing character might promote higher RNA 2′-OH sulfonylation reactivity. However, countering this is the likelihood that increasing the reactivity could also promote more rapid hydrolysis by water prior to reaction with RNA, leading to diminished sulfonylation yield. To investigate this, we prepared a set of 3-pyridylsulfonyl species having a range of leaving groups (Table 1). The compounds were used in parallel reactions with three different short RNA molecules (“Test”, “tRF”, “tRFau”, Supporting Information), under 10–50% DMSO conditions and the “TestDNA” control. Sulfonylation conversions were assessed by MALDI-TOF MS analysis after ethanol precipitation of the RNA. To measure aqueous stability, we also followed hydrolysis of several of the species via ^1^H NMR measurements in deuterated buffer (Supporting Information, Figure S4).

Our experiments revealed that several of the compounds were notably more stable than prior RNA 2′-OH modifying reagents in aqueous environments.^7,8,11,25^ For example, **1** and **2** displayed half-lives of nearly 7 days and 8 h respectively in the aqueous buffer, as compared with a 34 min *t*_1/2_ of acylimidazole reagent NAI and 14 s *t*_1/2_ of isatoic anhydride 1M7 (Figure 1B). More reactive leaving groups in **6** and **4** (2-trifluoromethylimidazole and 2-cyanoimidazole, respectively) resulted in shorter half-lives of ca. 4 h and 4 min respectively, consistent with expectation. **5** and **7** (with carbaldehyde and nitro substituents at C2 of imidazole) were found to be too reactive and were unstable in DMSO stock solution, and hence not used for further experiments.

Due to the limitations of C2 substituted imidazoles, we explored a wider variety of leaving group structures. Inspired by many literature examples of the wide applicability of SuFEx reagents as covalent tags in aqueous environments, particularly in protein profiling by reaction with nucleophilic side chains of amino acids^22,23,26^ and a recent report of engineered protein-directed RNA modification,^21^ we tested the simple pyridine-3-sulfonyl fluoride **9**. In addition, we tested an NHS sulfonyl ester (**10**). Both **9** and **10** were found to be relatively stable in aqueous environments, with half-lives of approximately 6 h each, allowing sufficient availability in solution for RNA 2′-OH sulfonylation to occur, and both displayed significant levels of RNA reaction (25% and 12% respectively), but were unable to surpass the 54% sulfonylation yield of **2**. Considerably less reactive than these were imidazolium, pyridinium, and azide leaving groups (**11**–**13**), which gave no detectable yields with RNA (Table 1).

Numerous reports in the literature have utilized sulfonyl triazoles (1,2,3- and 1,2,4- isomers) as electrophilic covalent modification agents for proteins (via sulfur(VI) triazole exchange, (SuTEx)),^18,27^ prompting us to investigate these as well. Reaction of pyridine-3-sulfonyl chloride with 1,2,3-triazole yielded a mixture of **14** (major, N2-linkage) and **15** (minor, N1-linkage) products; and reaction with 1,2,4-triazole yielded only **16** (N1-linkage). Encouragingly, **15** was found to achieve sulfonylation conversion with RNA of 70%; however, it was found to be particularly unstable in aqueous solution, and its half-life could not be measured. Compounds **14** and **16** were both found to improve on that yield, affording 84% and 81% conversion, respectively. Importantly, both of these molecules also have aqueous half-lives of ca. 90 min, nearly three times that of the structurally similar acylimidazole NAI.^6^ Again, control reactions with DNA with these three sulfonyltriazoles revealed little or no reaction, confirming the reaction site of 2′-OH in RNA (Figure S6). Analyzing this information, moving forward we employed 1,2,4-triazole as an optimal leaving group to maximize sulfonylation yield, avoiding the isomeric mixtures that result with 1,2,3-triazole. We followed up on this finding by testing a range of aryl and alkyl 1,2,4-triazole-activated sulfonyl species (Supporting Information; Table S3, Table S4), but the solubility and electron deficiency afforded by the pyridine scaffold proved optimal, and subsequent experiments and conjugations were carried out with this scaffold as a basis.

### Reagent Synthesis and Stability

**Figure 2:**
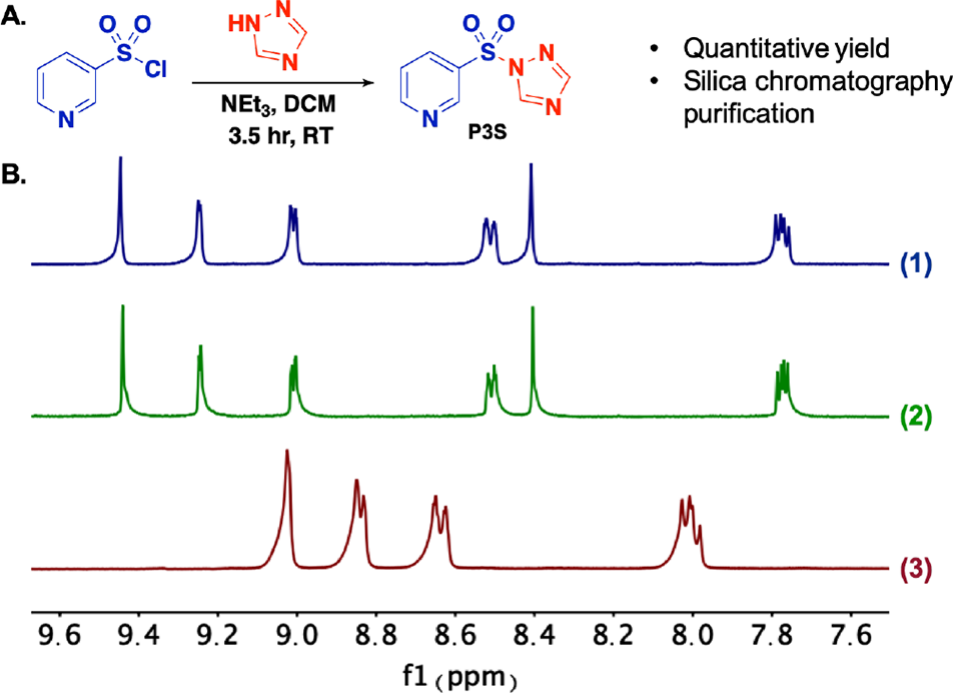
Synthetic scheme and stability analysis of reagent **P3S**. (**A**) 1-step synthetic scheme. (**B**) Stability analysis of **P3S** by ^1^H NMR (solvent: DMSO-*d*_6_, 500 MHz; full spectra in Supporting Information). Spectrum (**1**): Freshly synthesized **P3S** after column purification; Spectrum (**2**): Same batch of **P3S** after 8 months’ storage at −20 °C; Spectrum (**3**): Authentic commercial sample of pyridine-3-sulfonic acid. These results demonstrate the stability of **P3S** under common laboratory storage conditions.

Previously reported and commonly used RNA 2′-OH modifying acylimidazole reagents require either cold storage in frozen solution in their activated forms, or fresh synthesis of each batch of active molecule from inactive precursor. The standard preparation of the acylimidazoles directly in dry DMSO stock solution also generates one equivalent of imidazole, and although the imidazole is not known to interfere with applications, it can affect buffering power of solutions in which it is applied. Another commonly used class of RNA 2′-OH modifying agents, isatoic anhydrides,^28,29^ is also not amenable to chromatographic purification techniques, and must rely on extraction. In general, improvements in stability of reagents (while maintaining tunable RNA reactivity) and amenability to purification are desirable. Our preparation studies with test compound **16** (henceforth referred to as **P3S**) showed that it is readily accessible in near-quantitative yield in one simple synthetic step from commercially available pyridine-3-sulfonyl chloride and 1,2,4-triazole (Figure 2A and Supporting Information). Importantly, we were able to perform silica chromatography with the reaction mixture, enabling the isolation of purified **P3S** as a white powder without detected impurities. Conveniently, gram-scale synthesis of **P3S** is also feasible, with 89% preparative yield (Supporting Information).

Encouraged by such ease of synthesis and purification for **P3S** and supported by the extended hydrolysis half-life, we further probed its stability under laboratory storage conditions. We found that when it is stored under Ar at −20 °C, it is completely stable for at least 8 months with no hydrolysis (to the sulfonic acid) detected by ^1^H NMR (Figure 2B) and survives well for weeks to months at ambient temperature on the bench (Supporting Information, Figure S13). Finally, we also found that **P3S** adducts on Test RNA are stable for at least 8 days in water at 37 °C (Figure S14), whereas some acyl adducts of RNA are hydrolyzed after a few hours on RNA, thus requiring rapid analysis before the adduct is gone.^28^ Adduct stability is also important in covalent labeling, where extended stability of the conjugate can render it considerably more useful without risk of diminishment of the labeled signal due to hydrolysis of the linkage.

### Application of Sulfonylation to RNA Labeling

**Figure 3:**
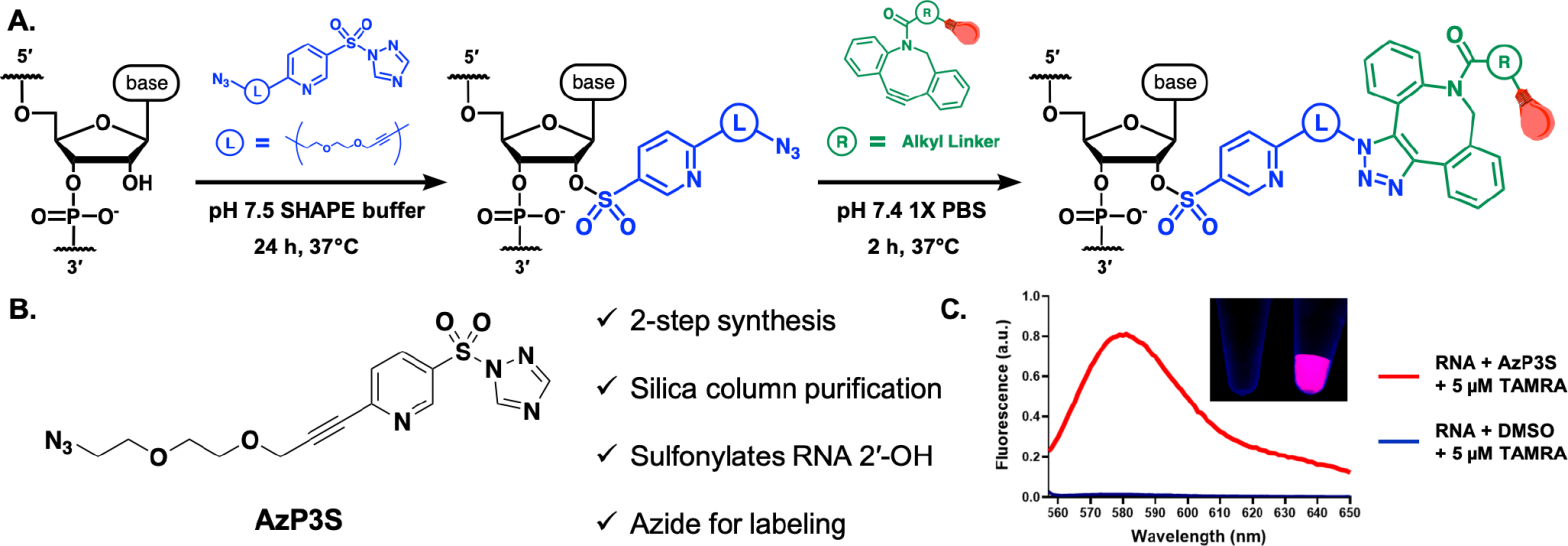
Sulfonylation-enabled labeling of RNA. Fluorescence labeling of tRF3 RNA sulfonylated with **AzP3S** using strain-promoted Cu-free click chemistry. (**A**) Scheme for labeling **AzP3S**-sulfonylated RNA with a fluorophore using Cu-free click chemistry. (**B**) **AzP3S** structure. (**C**) Fluorescence spectra of labeled RNA (100 nM) with excitation at 546 nm (a.u.: arbitrary units). Inset: Visual evidence of fluorescent labeling. 2 μM RNA excited on transilluminator at 365 nm. Left: tRF3 RNA treated with DMSO only and then 50 μM TAMRA-DBCO. Right: tRF3 RNA reacted with 50 mM **AzP3S**/DMSO and then 50 μM TAMRA-DBCO. For detailed experimental procedures, see Supporting Information.

Chemical agents that can covalently modify RNA offer the potential for conjugation with labels. For example, RNA can be covalently tagged with fluorophores for detection, imaging, and FRET analysis,^29−31^ or with biotin for pull-down and separation protocols.^7,32^ However, most RNA covalent labeling methods involve enzymatic nucleotide incorporation protocols or engineered RNA sequences,^33−37^ and only a few reagents based on short-lived activated carbonyl species have been tested for high-yield labeling.^7,11^ Given the stability and ease of synthesis of sulfonyltriazoles, we asked whether such species could be used to introduce a reactive handle in RNA. For our first test, we designed a sulfonyl triazole-containing reagent functionalized with an azide functional group that could potentially react with RNA 2′-OH groups, potentially allowing for further RNA conjugation. We synthesized derivative **26** (henceforth referred to as **AzP3S**, Figure 3B), a stable azide-containing pyridine-3-sulfonyltriazole compound, in only two steps from commercially available starting material in good yields (Supporting Information). Remarkably, the sulfonyltriazole moiety remained stable to Pd-mediated Sonogashira chemistry and to silica chromatography. Encouragingly, we found that **AzP3S** sulfonylates an 18-nucleotide tRF3 RNA with 87% conversion at 50 mM concentration over 24 h at 37 °C (Supporting Information, Figure S16). This is similar conversion as has been achieved with carbon-based electrophiles,^30^ albeit requiring longer time due to the lower reactivity of the sulfonyl species. To introduce a fluorescent label, we precipitated the RNA to remove residual small-molecule species and then redissolved and performed a strain-promoted cycloaddition reaction, employing commercial TAMRA-DBCO and Cy5-DBCO for 2 h in PBS buffer (Figure 3A). The cycloadditions occurred with >90% conversion by MALDI-TOF MS with both dyes under mild conditions, and the labeled RNA was readily separated from excess dye via ethanol precipitation (Figure S17, Figure S18). Fluorescence measurements confirmed the presence of the dye on the RNA and the requirement for sulfonylation by **AzP3S** for covalent labeling; and TAMRA-labeled RNA was detectable by eye over a transilluminator (Figure 3C). Denaturing PAGE gel analysis (20%) confirmed the presence of the Cy5 dye on tRF3 RNA and that **AzP3S** treatment is necessary for labeling (Figure S19).

**Figure 4:**
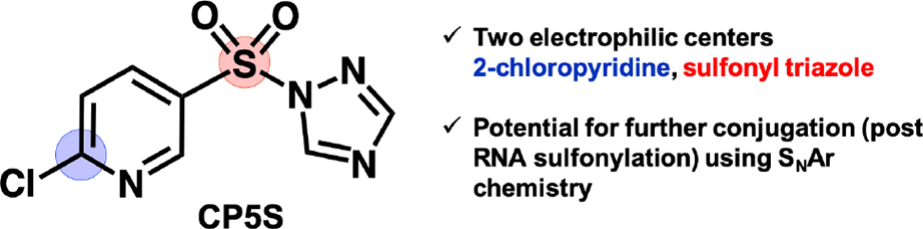
Structure of sulfonylating reagent **CP5S**, with two electrophilic centers highlighted. This reagent enables postfunctionalization via S_N_Ar (using alkyl and aryl thiols) at 2-chloropyridine after RNA 2′-OH sulfonylation.

Prompted by the versatility and ease of synthesis of the above sulfonyltriazole reagents, we investigated the possibility of a second covalent conjugation strategy that could further exploit the particular chemical characteristics of an electron-deficient pyridinesulfonyl group. Compound **19** (henceforth referred to as **CP5S**, Figure 4) possesses a chloro substituent at C-2 that is activated both by the pyridine nitrogen and by the *para*-situated electron-withdrawing sulfonyl group; and sulfonylates Test RNA with 55% conversion (Figure S9). Encouraged by reports of S_N_Ar strategies applied toward covalent tagging of nucleic acids using incorporated halopurine nucleotides, and cysteine modification in proteins using 2-sulfopyridine electrophiles,^38,39^ we hypothesized that C-2 of our pyridyl reagent might be subject to nucleophilic substitution via a S_N_Ar reaction with an appropriate nucleophile (Figure S20). After screening a small panel of simple thiols, we found that a short 18-nucleotide “tRF” hairpin RNA sulfonylated with **CP5S** could be further functionalized with thiophenol or a PEG thiol over 24 h at 25 °C in the presence of Et_3_N base, with >95% yield for these thiols (Figures S21–S23). Although further work is required to establish the scope and reactivity characteristics of this class of novel sulfonyl electrophile on RNA, we note that such S_N_Ar conjugation reactions were not previously possible without the use of solid phase synthesis to incorporate the electrophilic center into nucleic acids.

### Investigation of Sulfonylation-Based RNA Structure Mapping

Since most RNA functions are dependent on secondary/tertiary structure and on dynamic structural changes, methods of assessing folded structure are important tools for research.^40−45^ One of the most widely used empirical techniques is SHAPE, which yields structural information at single nucleotide resolution.^3,6^ SHAPE exploits the preference of RNA 2′-OH acylating agents to preferentially covalently modify RNA 2′-OH at unpaired locations on folded RNA molecules over paired residues, allowing RNA secondary structure readout after analysis of subsequent reverse transcriptase (RT) stops, which are commonly read by gel electrophoresis.^7^ In contrast to high-yield conjugation reactions on RNA, SHAPE probing employs only trace levels of reaction.

**Figure 5:**
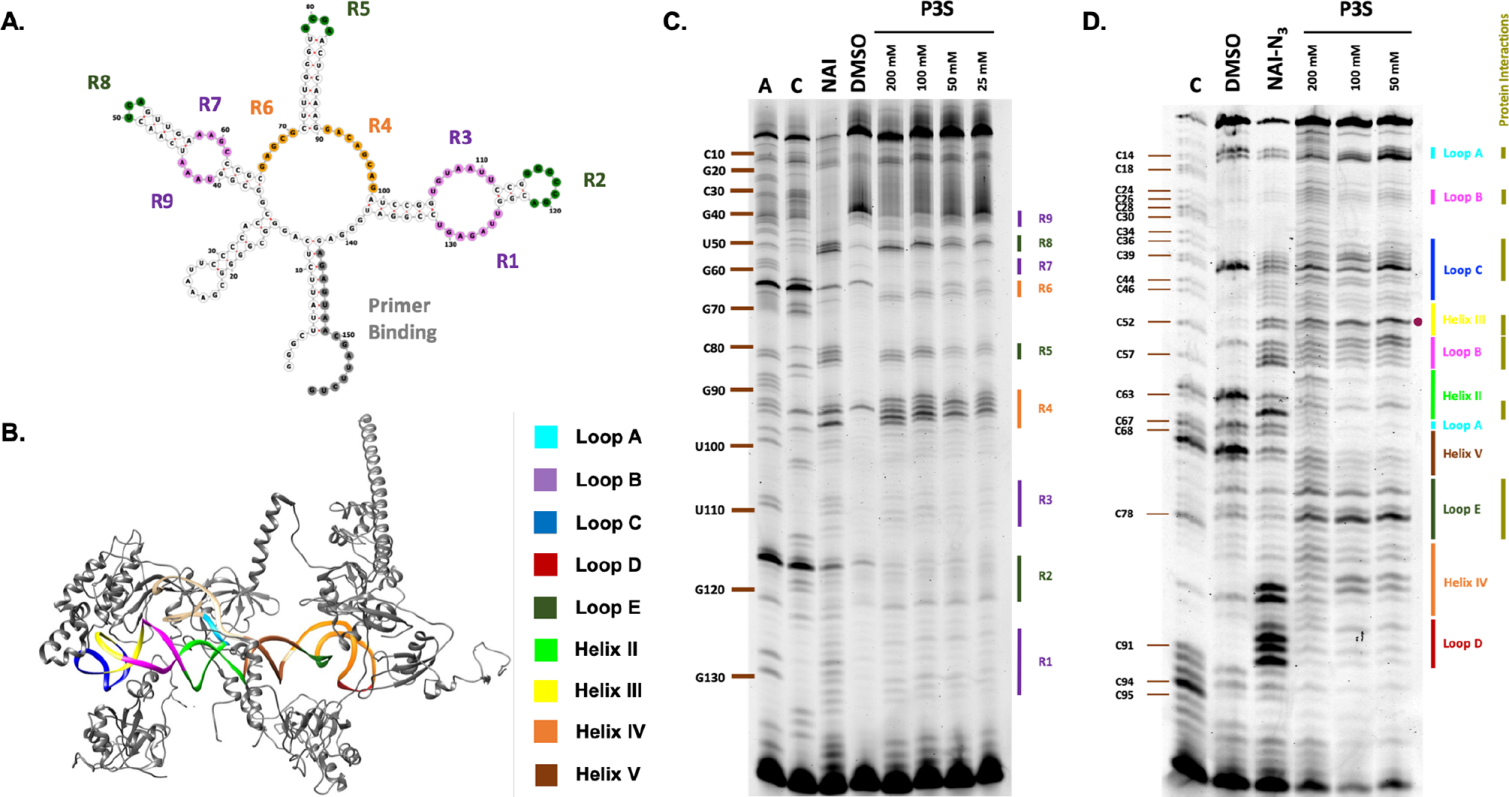
*In vitro* and *in vivo* RNA structure mapping with sulfonylating reagent **P3S**. (**A**) Secondary structure of flavin mononucleotide (FMN) riboswitch predicted by RNAFold folding program.^51^ Primer binding site (**gray**) for reverse transcriptase primer extension and detected hairpin loops (**R2**, **R5**, **R8**), multibranched loops (**R4**, **R6**) and internal loops (**R1**, **R3**, **R7**, **R9**) are as shown. (**B**) Structure of human 5S rRNA with interacting ribosomal proteins (PDB 4UG0, visualized with UCSF Chimera software^52^). (**C**) *In vitro* structure probing of FMN RNA by sulfonylating reagent **P3S** (200 mM, 100 mM, 50 mM, 25 mM; 1 h) compared with a common mapping reagent (200 mM NAI, 5 min) at 37 °C. (**D**) *In vivo* structure probing of human 5S rRNA with **P3S** (200 mM, 100 mM, 50 mM) for 1 h, and 100 mM NAI-N_3_ for 25 min in HeLa cells, at 37 °C. The structural features Loops **A**, **B**, **C**, **D**, **E** and Helices **II**, **III**, **IV**, **V** and **protein interaction** sites are denoted in the gel corresponding with the 5S rRNA structure; **A50** is denoted with a dot.

We hypothesized that since the sulfonyl species **P3S** is structurally similar to commonly used pyridine-based acyl SHAPE reagents such as NAI, NAI-N_3_, and 2A3,^7,46,47^**P3S** might also demonstrate structure-sensitive 2′-OH modification on folded RNAs. As an initial test of this possibility *in vitro*, we chose the well-studied 157 nt *E. coli* FMN riboswitch RNA,^48,49^ which possesses a variety of secondary structural elements (Figure 5A).^50^ Encouragingly, we discovered that over the sulfonylating reagent concentration range of 25–200 mM, incubation with the FMN riboswitch (1 h, 37 °C) afforded RT-stop bands that suggest structure-sensitive RNA modification. Experiments also revealed that the level of RNA modification increased over time from 5 to 40 min, while maintaining structure sensitivity (Figure S24). Specifically, we found that the established unpaired regions of the FMN riboswitch including hairpin loops (**R2**, **R5**, **R8**), multibranched loops (**R4**, **R6**) and internal loops (**R1**, **R3**, **R7**, **R9**) underwent higher levels of modification compared to other regions of the RNA (Figure 5A,C). Especially prominent were bands in the **R4**, **R5**, **R6**, and **R8** unpaired domains, which were also similar for the commercial reagent NAI. Interestingly, bands were not identical between **P3S** and NAI (Figure 5C); for example, NAI showed a bias of higher reactivity near the 5′-end of loops **R2** and **R6**, whereas **P3S** showed the opposite trend, with a greater reaction near the 3′ half of the loops. We hypothesize that the different chemical properties of the sulfonyl triazole moiety in **P3S** compared to the acyl imidazole in NAI (*e.g*., tetrahedral sulfonyl center vs planar carbonyl group) as well as lower reactivity of the sulfonyl molecule could be responsible for the differences in patterns. Overall, the data reveal clear selectivity for sulfonylation of unpaired regions over helices, and also confirm that the sulfonyl adduct, like acyl, causes reverse transcriptase stops, both of which enable structural mapping.

As a further investigation of RNA folded structure mapping, we performed tests of the ability of **P3S** to probe a 121 nt segment of human 5S rRNA in HeLa cells, as its structure has been widely studied in the literature and its interactions with proteins *in vivo* well-documented (Figure 5B).^53,54^ Using a range of **P3S** concentrations (50–100 mM) and limiting DMSO to 10% of total volume of the cell incubation mixture, we found that 1 h incubation at 37 °C with HeLa cells afforded sufficient rRNA modification for structure mapping (Figure 5D). Encouragingly, we found that the regions that underwent the most RNA modification upon treatment with **P3S** were the same as those in the acylimidazole NAI-N_3_ lane, at least with 50 mM and 100 mM **P3S**, suggesting structure-sensitive modification. Interestingly, within these regions we observed significant differences in RT-stop patterns; for instance, although NAI-N_3_ indicates significant RNA modification at some residues in **Loop D**, **Helix II**, and **Helix V**, **P3S** lanes suggest little modification at those regions. On the other hand, at **Loop B**, **Loop C**, and **Loop E** the RT-stop patterns indicate that the level of RNA modification is higher with **P3S** than that with NAI-N_3_. Interestingly, the section of the junction **Loop A** closer to the 5′ end of the rRNA is modified to a greater extent by **P3S** than NAI-N_3_, but at the other section we observed minimal **P3S** modification. Finally, at **Helix III** and **Helix IV** we observe significant modification with both **P3S** and NAI-N_3_; however, the location preference of modification within these regions is different for each molecule. In cells, the adenosine **A50** is known to be present in a bulge within the **Helix III** region, and we detected a high level of RNA modification at that location, further suggesting structure-sensitive **P3S** sulfonylation.

We also noted that in the structure mapping experiments *in vivo* (Figure 5D) the concentration of sulfonylating reagent **P3S** has a significant effect on RNA reactivity. Below 100 mM conditions, the locations of sulfonylation and reactivity pattern remain largely consistent with structure-sensitive RNA modification, as noted above. When the concentration of **P3S** was increased to 200 mM, more nonselective RNA modification was observed. We hypothesize that high concentrations and extended times may promote toxicity, potentially affecting RNA structure. This hypothesis is supported by the observation that *in vitro* mapping at 200 mM does not lead to loss of selectivity (Figure 5C). Additional studies are clearly required to test cellular structure-mapping applications of such compounds.

More work is also needed to further establish the reactivity trends of 2′-OH sulfonylating molecules with respect to a wider variety of folded RNA structures. However, the initial tests with the folded FMN riboswitch and 5S rRNA indicate potential utility of RNA 2′-OH sulfonylating agents as an alternative class of tunable RNA structure mapping reagents; with the added benefits of being considerably easier to synthesize, purify, and store than current acyl reagents, as well as the potential benefit of stability once the RNA has been modified. For potential cellular application, these initial results also indicate useful levels of cell permeability and structure-sensitive RNA modification. Reactivity of the current sulfonyl species is lower than that of acyl mapping agents, but given the wide tunability of reactivity (Table 1), future reagents with greater reactivity may also prove useful. We hypothesize that having a wide range of reactivities in mapping agents could prove useful in analyzing RNA structural dynamics in the future; for example, the widely used *in vitro* mapping reagent 1M7 has a half-life of ca. 14 s,^8^ while the current **P3S** reagent’s half-life is >300-fold longer, thus offering a wide dynamic range of reactivity.

## Conclusion

Our results overall establish that small-molecule sulfonyl species with appropriate leaving groups can react with RNA at 2′-OH groups, thus introducing a novel reagent class for RNA reaction, and providing new opportunities for conjugation and structure mapping. The sulfonyl reagent class offers a number of appealing features, including the ability to selectively functionalize RNA 2′-OH in practically useful yields, while also allowing the tuning of reactivity by simple, modular changes in reagent structure. The sulfonyl compounds are sufficiently soluble for aqueous application, and are remarkably stable in water (with half-lives of several hours) while retaining the ability to react with RNA 2′-OH. We find that they are stable in storage for extended periods even at room temperature. Importantly, they are readily synthesized, and can be easily purified by silica gel chromatography. Such facile purification and storage are significantly limited with most current RNA acylating agents; for example, most acylimidazoles used previously are best stored at low temperatures to prevent hydrolysis, and are commonly applied as a stoichiometric mixture in DMSO solution with imidazole, due in part to their lack of stability to column chromatography.

The sulfonyl reagents offer a practical alternative for covalent labeling of RNA. We successfully utilized the copper-free strain-promoted click strategy to attach TAMRA and Cy5 dyes onto **AzP3S**-sulfonylated RNA. **AzP3S** is synthesized in only two steps, and multiple strained-alkyne reagents are commercially available as potential reactive partners for **AzP3S**-modified RNA, likely allowing labeling future application with dyes of varied emission properties as well as affinity labels. In addition, we show that a 2-chloropyridine sulfonate adduct on RNA can be conjugated in high yields with simple alkyl and aryl thiols via a S_N_Ar reaction.

The current experiments also suggest the potential for sulfonyltriazole compounds as alternatives for RNA structure mapping. Like common isatoic anhydride and acylimidazole structure-mapping reagents, we show that the sulfonyl reagents react preferentially at 2′-OH groups. Here we demonstrated with a folded riboswitch RNA that the simple pyridine sulfonyl triazole molecule **P3S** is able to perform RNA 2′-OH sulfonylation *in vitro* with a reactivity preference for unpaired RNA regions that is similar to that for carbon-electrophile RNA SHAPE reagents. While development of RNA 2′-OH sulfonylation is still at an early stage when compared to other well-established RNA reactive molecules, this suggests the potential for the use of these easy-to-handle sulfonylating agents for future RNA structural mapping studies. Efforts are currently underway to determine the reactivity characteristics of these sulfonylating agents toward a wider variety of RNA secondary structures. Encouragingly, we also observed structure-sensitive 2′-OH reactivity of **P3S** with a rRNA in HeLa cells. The lower reactivity of sulfonylating molecules compared to cell-permeable acylimidazoles (e.g., NAI) necessitates the use of a longer incubation time (1 h) in cellular experiments for a sufficient signal in gel analysis, which may interfere with cell biology during the time of the experiment. However, the results do suggest useful levels of cell permeability of the reagent tested here, and further studies with more reactive sulfonyl species are merited in the future for *in vivo* application in RNA research.

The sulfonyl reagent class offers new chemistry for RNA functionalization. To date, all RNA 2′-OH reactive small molecules reported in the literature have possessed just one class of functional group—activated carbonyl—as an electrophile to allow for bond formation with the nucleophilic ribose 2′-hydroxyl. The use of carbonyl electrophiles has restricted the adducts to ester and carbonate linkages with RNA 2′-OH. This is in sharp contrast to the protein literature, which documents a wide variety of electrophilic modifications at amino acid side chains. Our new findings diversify the type of selective small-molecule covalent linkages possible at RNA 2′-OH, generating O-sulfonate ester linkages at RNA ribose 2′-carbon using a variety of different sulfonyl species. The considerably different chemical properties of the tetrahedral sulfonate ester group (compared to the planar ester/carbonate groups) could also be potentially exploited in future studies to develop new chemistries and applications for RNA. For example, sulfonate esters are well-known as intermediates for substitution and elimination reactions,^55,56^ raising this future possibility for RNA.

## Supporting Information Available

Oligonucleotides used in this work, reagents, materials, supplementary data and figures, experimental details, synthetic procedures, NMR spectra (PDF)
