# Supplementary material for: Sulfonylation of RNA 2′-OH groups
Source: ACS Cent Sci. 2023 Mar 1;9(3):531–9. doi: 10.1021/acscentsci.2c01237 (PMC10037496; doi:10.1021/acscentsci.2c01237)
Supplement: Supplementary file 1 — oc2c01237_si_002.pdf [file oc2c01237_si_002.pdf]

Supplementary Information

Sulfonylation of RNA 2′-OH groups

Sayantan Chatterjee, Ryuta Shioi and Eric T. Kool*

Department of Chemistry, Stanford University, Stanford, CA 94305
**Author to whom correspondence should be addressed: kool@stanford.edu*

Table of Contents

1. Table S1 (Oligonucleotides used in this work) S2
2. Table S2 (Reagents and materials used) S3-S5
3. Table S3 (Aryl sulfonyl triazole molecules tested for RNA reactivity) S6
4. Table S4 (Alkyl sulfonyl molecules tested for RNA reactivity) S7
5. Supplementary Figures S8-S35
6. Experimental Procedures S36-S39
7. Synthesis of reagents S40-S55
8. NMR spectra (^{1}H, ^{13}C) S56-S120
9. References S121

Table S1. Oligonucleotides used in this work

Name	Sequence (left to right: 5′ to 3′)
**RNA oligo**	
Test	GCUCCCCAGCUUUCGC
tRF	UGGCGGCCGACUACGCCA
tRFau	UGUAUAGGCCGACUACUAUACA
tRF3	AUCCUGCCGACUACGCCA
polyU	UUUUUUUUUUUUUUUUUUUU
**DNA oligo**	
TestDNA	GCTCCCCAGCTTTCGC
FMN RT primer	/Cy5/ CAGAATCGTTACTCTCTCC
Human 5S rRNA RT primer	/Cy5/ AAAGCCTACAGCACCCGGTAT
DNA template for FMN RNA	TAATACGACTCACTATAGGGCTTATTCTCAGGGCGGGGCGAAATTCCCCACCGGCGGTAAATCAACTCAGTTGAAAGCCCGCGAGCGCTTTGGGTGCGAACTCAAAGGACAGCAGATCCGGTGTAATTCCGGGGCCGACGGTTAGAGTCCGGATGGGAGAGAGTAACGATTCTG
Forward PCR primer for FMN template amplification	TAATACGACTCACTATAGGGC
Reverse PCR primer for FMN template amplification	CAGAATCGTTACTCTCTCC

Table S2. Reagents and materials used

REAGENTS	SOURCE	IDENTIFIER
**Reagents and Enzymes**		
5M NaCl	Invitrogen™	# AM9760G
1M $MgCl_2$	Invitrogen™	# AM9530G
MOPS, 1.0M buffer soln., pH 7.5	Thermo Scientific™	# J61843.AP
UltraPure DTT	Invitrogen™	# 15508013
NTP Set (100 mM Solution)	Thermo Scientific™	# R0481
dNTP mix (10 mM each)	Thermo Scientific™	# R0194
SYBR Gold Nucleic Acid gel stain (10,000X)	Thermo Scientific™	# S11494
SequaGel - UreaGel Concentrate	National diagnostics™	# EC-830
SequaGel - UreaGel Diluent	National diagnostics™	# EC-840
SequaGel - UreaGel Buffer	National diagnostics™	# EC-835
Agarose	Fisher Scientific™	# BP1356500
Ammonium Persulfate	Thermo Scientific™	# 17874
N, N, N′, N′-Tetramethyl ethylenediamine	Sigma-Aldrich™	# 110732
Deuterium oxide	Sigma-Aldrich™	# 151882
Dimethyl sulfoxide-d6	Sigma-Aldrich™	# 151874
Chloroform-d	Sigma-Aldrich™	# 151823
TAMRA DBCO	Click Chemistry Tools™	# A-131
Cy5-DBCO	Click Chemistry Tools™	# A-130
Thiophenol	Sigma-Aldrich™	# 240249
2-(2-Methoxyethoxy)ethanethiol	Sigma-Aldrich™	# 632295
1-Butanol	Sigma-Aldrich™	# 537993
O-Methylhydroxylamine Hydrochloride	1-PlusChem™	# 1P0035W8
Potassium iodide	Sigma-Aldrich™	# 221945
Thioacetic acid	Sigma-Aldrich™	# T30805
Potassium thioacetate	Sigma-Aldrich™	# 241776
TrypLE™ Express Enzyme (1X), phenol red	Thermo Scientific™	# 12605010
Urea	Sigma-Aldrich™	# U5128
Orange G	Thermo Scientific™	# 416551000
Bromophenol Blue	Thermo Scientific™	# A18469.09
Pyridine-3-sulfonyl chloride	Ambeed™	# A626684
Dichloromethane	Fisher Scientific™	# 041835.M1
Imidazole	Sigma-Aldrich™	# I2399
N,N-Diisopropylethylamine	Sigma-Aldrich™	# D125806
Hexane	Fisher Scientific™	# H292-20
Ethyl acetate	Fisher Scientific™	# E124-20
Acetonitrile	Fisher Scientific™	# 042311.AK
2-iodoimidazole	AA Blocks™	# AA002ZQC
Chloroform	Sigma-Aldrich™	# C2432
Sodium sulfate	Fisher Scientific™	# S415-500
1H-Imidazole-2-carbonitrile hydrochloride	AA Blocks™	# AA001AKM
2-formylimidazole	AA Blocks™	# AA0003OI
2-(Trifluoromethyl)-1H-imidazole	1PlusChem™	# 1P0037G3
2-Nitroimidazole	AA Blocks™	# AA003HPX

N-Hydroxysuccinimide	Sigma-Aldrich™	# 130672
1-Methylimidazole	Fisher Scientific™	# A12575.36
4-Dimethylaminopyridine	Fisher Scientific™	# 148275000
1H-1,2,3-Triazole	Fisher Scientific™	# A16999.03
1,2,4- Triazole	Sigma-Aldrich™	# T46108
1H-Benzotriazole	Fisher Scientific™	# A15423.0B
Pyridine-2-sulfonyl chloride	Ambeed™	# A439415
6-Chloro-3-pyridinesulfonyl chloride	ChemScene™	# CS-W009066
6-bromopyridine-3-sulfonyl chloride	AA Blocks™	# AA00GS0I
6-Nitropyridine-3-sulfonyl chloride	AA Blocks™	# AA01AI0H
Quinoxaline-5-sulfonyl chloride	ChemScene™	# CS-0110639
Furan-3-sulfonyl chloride	AA Blocks™	# AA00DCO7
1H-Imidazole-4-sulfonyl chloride, 1-methyl-	1PlusChem™	# 1P001389
1-Methyl-1h-pyrazole-4-sulfonyl chloride	AA Blocks™	# AA002WJ0
Propargyl-PEG2-azide	Axis Pharm™	# AP10131
Bis(triphenylphosphine)palladium(II) dichloride	Sigma-Aldrich™	# 412740
Copper(I) iodide	Thermo Scientific™	# 011606.A1
4-(Dimethylamino)azobenzene-4′-sulfonyl chloride	Sigma-Aldrich™	# 39068
2-Methoxy-1-ethanesulfonyl chloride	Combi-blocks™	# ST-7060
2-Chloro-1H-imidazole	Ambeed™	# A265274
Methanesulfonyl chloride	Sigma-Aldrich™	# 471259
Morpholine-4-sulfonyl chloride	AA Blocks™	# AA00242T
4-Oxo-cyclohexanesulfonylchloride	ChemScene™	# CS-0130420
Toluene	Sigma-Aldrich™	# 244511
Phosphorus pentachloride	Sigma-Aldrich™	# 157775
pent-4-yne-1-sulfonyl chloride	Ambeed™	# A1064707
chloromethanesulfonyl chloride	Fisher Scientific™	# AC222460050
Ethanesulfonyl fluoride	Sigma-Aldrich™	# ALD00142
Pyridine-3-sulfonyl azide	Enamine™	# EN300-68832
DMSO	ACROS Organics™	# B0532976
Triethylamine	Fisher Scientific™	# O4885
Ammonium citrate dibasic	Sigma-Aldrich™	# 09833
2′,4′,6′-Trihydroxyacetophenone monohydrate	Sigma-Aldrich™	# T64602
10xTBE buffer	KD Medical™	# RGF-3330
RNaseOUT™ Recombinant Ribonuclease Inhibitor	Invitrogen™	# 10777019
SuperScript™ III Reverse Transcriptase	Invitrogen™	# 18080044
SuperScript™ II Reverse Transcriptase	Invitrogen™	# 18064022
Trizol LS Reagent	Thermo Scientific™	# 10296028
Dulbecco’s Modified Eagle Medium (DMEM)	gibco™	# 11995-065
1xPBS, pH=7.4	gibco™	# 10010-023
UltraPure DNase/RNase-free Distilled water	Thermo Scientific™	# 10977023
Glycogen, RNA grade	Thermo Scientific™	# R0551
3 M sodium acetate, pH=5.2	Thermo Scientific™	# AM9740
96% Ethanol	Fisher Scientific™	# BP8202-500
Fetal bovine serum (FBS)	gibco™	# 26140-079

RNA Gel loading dye (2x)	Thermo Scientific™	# R0641
**Commercial kits**		
Q5 hot start high fidelity PCR master mix	New England BioLabs™	# M0494S
HiScribe™ T7 Quick High Yield RNA Synthesis Kit	New England BioLabs™	# E2050S
MiniElute gel extraction Kit	QIAGEN™	# 28604
Quick-RNA Midiprep kit	Zymo Research™	# R1056
**Cell lines**		
HeLa	ATCC™	#CCL-2
**Software**		
ImageJ	NIH	N/A
GraphPad Prism 9	GraphPad Prism Software, Inc. ™	N/A
StepOnePlus Real-Time PCR System	Applied Biosystems™	N/A
Typhoon Scanner	GE healthcare™	N/A
MestReNova	Mestrelab™	N/A
ChemDraw 18.1	PerkinElmer™	N/A
UCSF Chimera	University of California, San Francisco	N/A

Table S3. Aryl sulfonyltriazole compounds tested for RNA reactivity

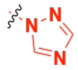

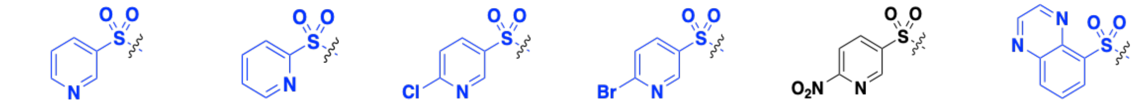

16 (81%, 100 mM) | **18** (53%, 100 mM) | **19** (55%, 100 mM) | **20** (54%, 100 mM) | **21** (n.d., < 100 mM*) | **22** (8.5%, < 25 mM)

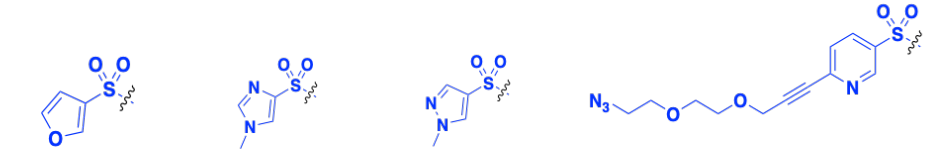

23 (29%, 100 mM) | **24** (5.1%, < 50 mM) | **25** (17%, 100 mM) | **26** (69%, 100 mM)

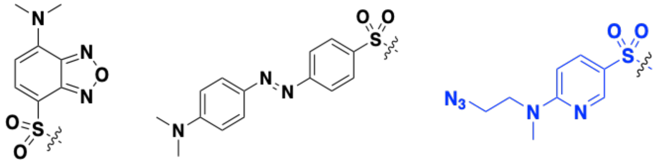

27 (n.d., < 20 mM) | **28** (n.d., < 20 mM) | **29** (0.48%, 100 mM)

Table S3. Scope of aryl sulfonyl-1,2,4-triazole molecules tested for RNA 2′-OH sulfonylation ability. Molecules in blue demonstrated conversion of RNA into sulfonylated products. In parentheses: % conversion of Test RNA achieved by each reagent under 20% DMSO conditions, concentration in reaction. “n.d.” indicates no measurable conversion. *: reagent unstable in DMSO (Reaction conditions: 37°C, 24 h, volume = 10 µL, [RNA/DNA] = 10 µM, [MOPS] = [NaCl] = 100 mM, [$MgCl_2$] = 6.06 mM; 10, 20, 50% DMSO, pH 7.5)

Table S4. Alkyl sulfonyl compounds tested for RNA reactivity

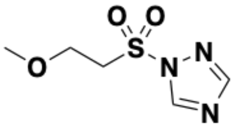

30
(n.d., < 33.3 mM)

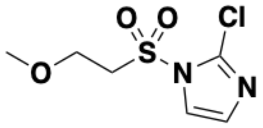

31
(n.d., 33.3 mM)

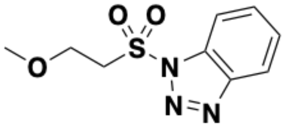

32
(n.d., 100 mM)

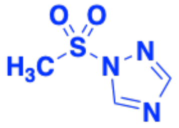

33
(3.2%, 100 mM)

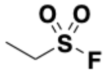

34
(n.d., < 100 mM)

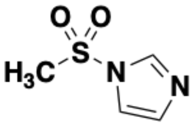

35
(n.d., 100 mM)

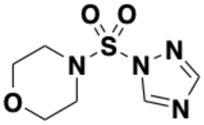

36
(n.d., < 50 mM)

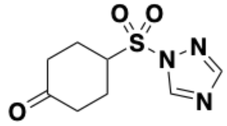

37
(n.d., < 50 mM)

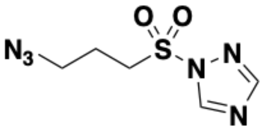

38
(n.d., 100 mM)

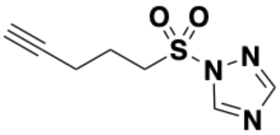

39
(n.d., < 50 mM)

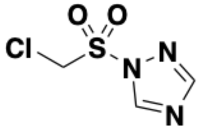

40
(n.d., < 100 mM*)

Table S4. Scope of alkyl sulfonyl molecules tested for RNA 2′-OH sulfonylation ability. Only **33** demonstrated conversion of RNA into sulfonylated products. In parentheses: % conversion of Test RNA achieved by each reagent under 20% DMSO conditions, concentration in reaction. "n.d." indicates no measurable conversion. *: reagent unstable in DMSO
(Reaction conditions: 37°C, 24 h, volume = 10 µL, [RNA/DNA] = 10 µM, [MOPS] = [NaCl] = 100 mM, [$MgCl_2$] = 6.06 mM; 10, 20, 50% DMSO, pH 7.5)

Supplementary Figures

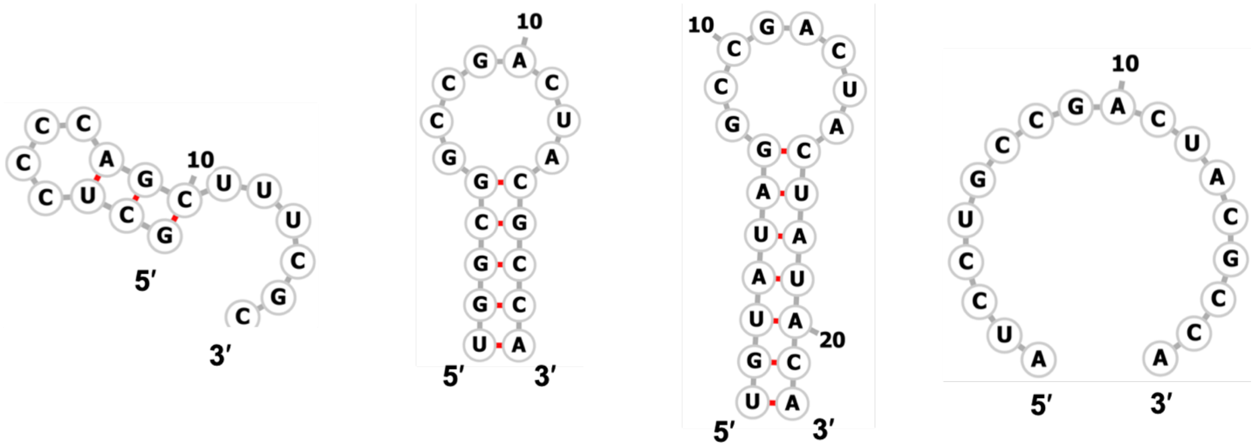

Figure S1. Predicted secondary structures of short RNAs used in MALDI-TOF analysis in this study. Left to Right: Test, tRF, tRFau, tRF3. TestDNA has the same sequence as Test RNA, with T instead of U. Predicted folds evaluated on RNAFold Web Server[1] with default parameters (37°C) and no isolated base pairs

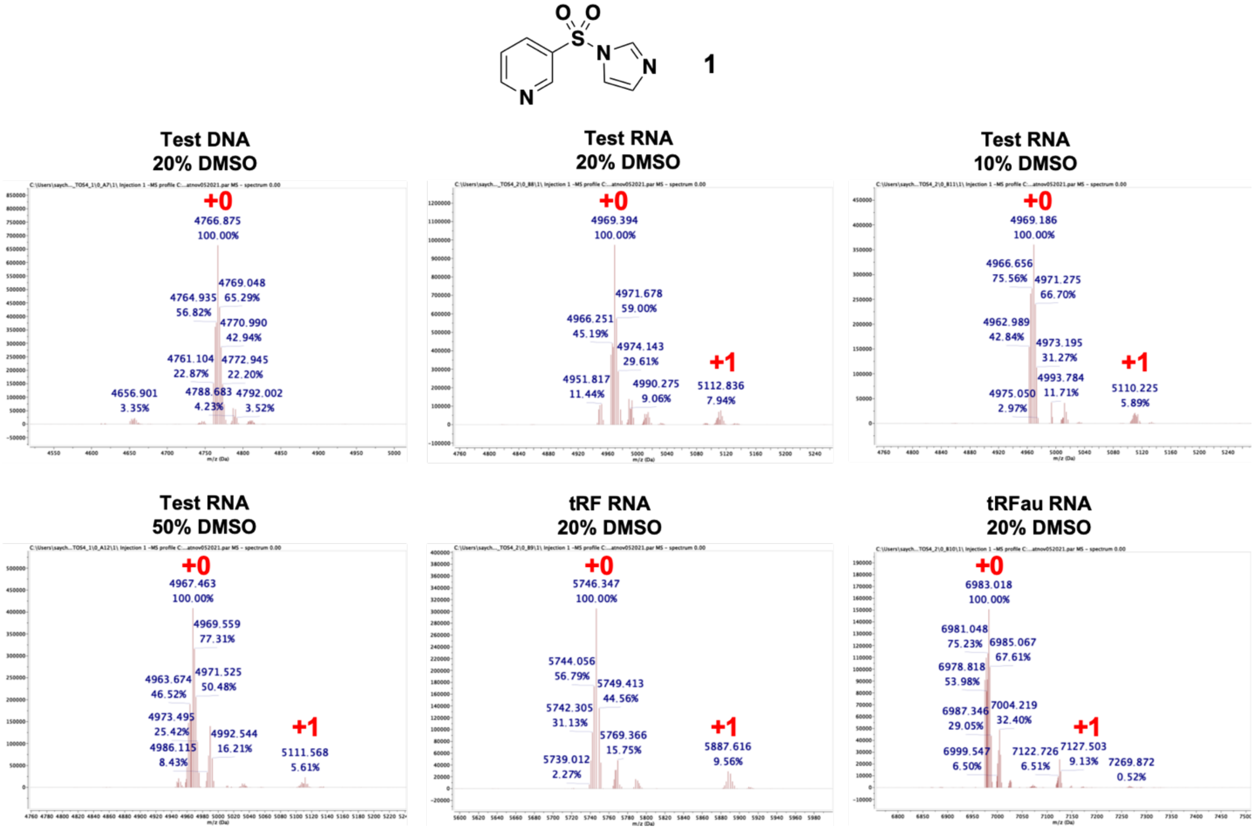

Figure S2. MALDI-TOF MS analysis of RNA reactions with reagent **1**. The compound reacts with Test RNA but not DNA with the same sequence, confirming preference for reaction at RNA 2′-OH over exocyclic amines. RNA reactivity is also observed over varying DMSO% and different RNA molecules. Representative spectra shown, experiment repeated three times with similar results. (Reaction conditions: 37°C, 24 h, volume = 10 µL, [**1**] = 100 mM, [RNA/DNA] = 10 µM, [MOPS] = [NaCl] = 100 mM, [$MgCl_2$] = 6.06 mM, pH 7.5)

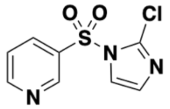

2

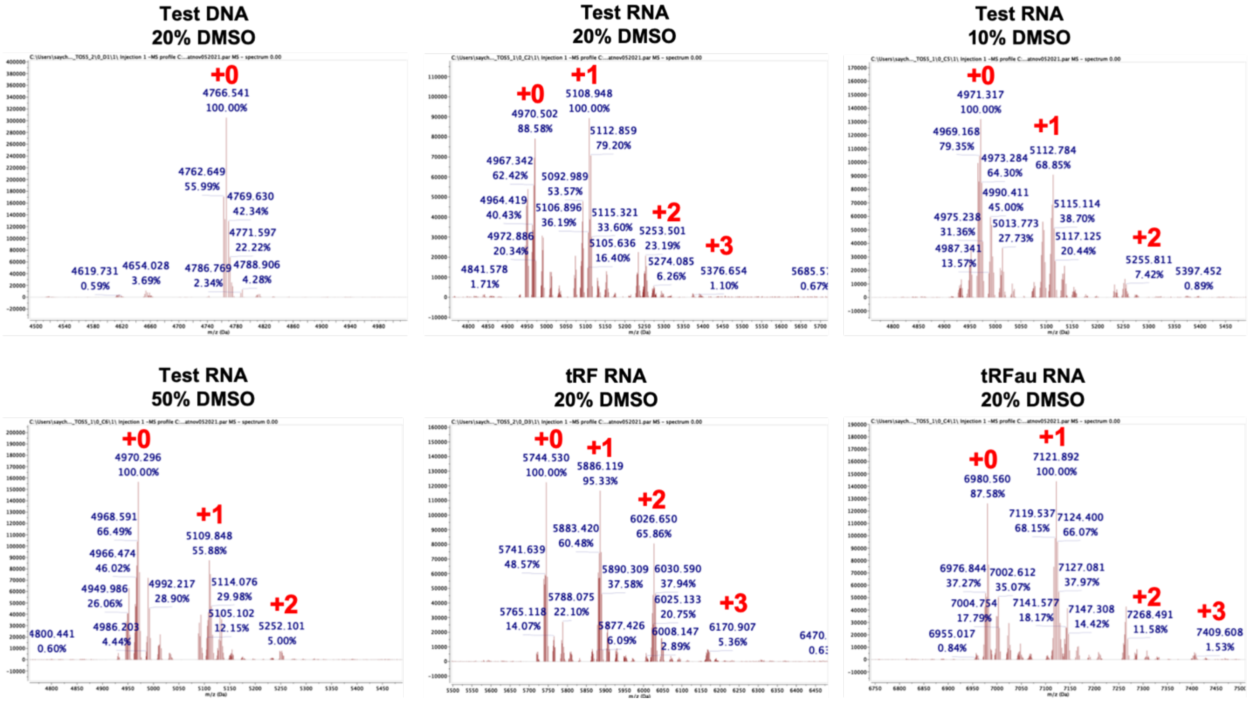

Figure S3. MALDI-TOF MS analysis of RNA reactions with molecule **2**. The reagent reacts with Test RNA but not DNA with the same sequence. This indicates preference for reaction at RNA 2′-OH over exocyclic amines. RNA reactivity is also observed over varying DMSO% and different RNA molecules. Representative spectra shown, experiment repeated three times with similar results.
(Reaction conditions: 37°C, 24 h, volume = 10 µL, [**2**] = 137 mM, [RNA/DNA] = 10 µM, [MOPS] = [NaCl] = 100 mM, [$MgCl_2$] = 6.06 mM, pH 7.5)

1

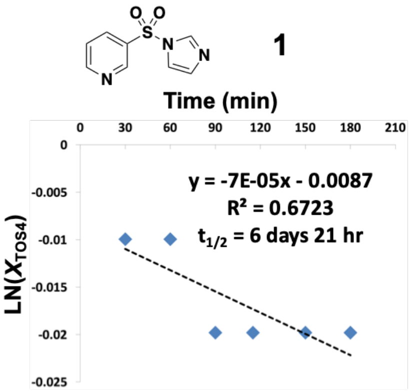

2

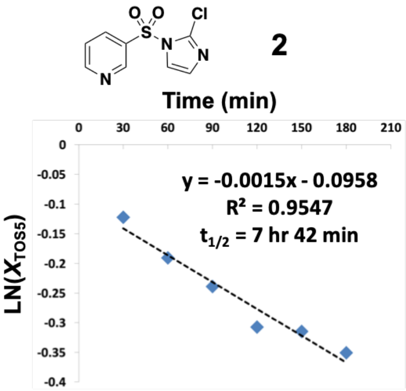

3

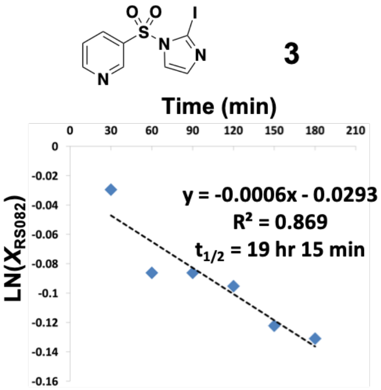

4

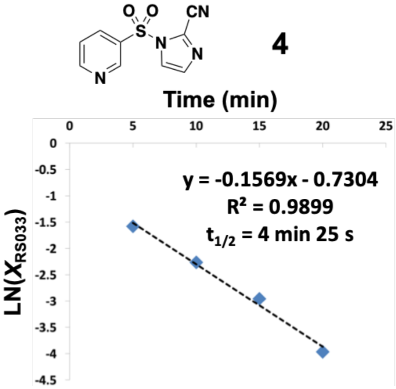

6

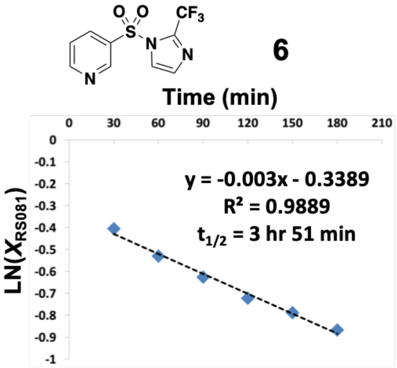

9

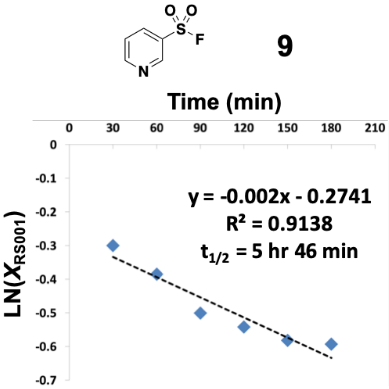

10

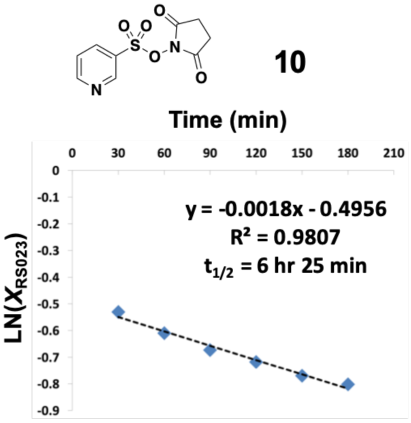

12

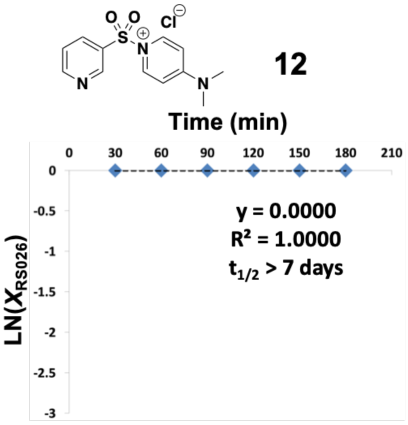

14

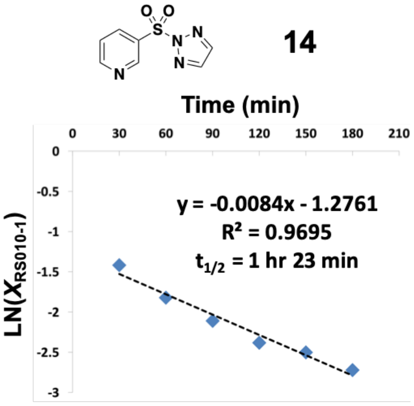

16

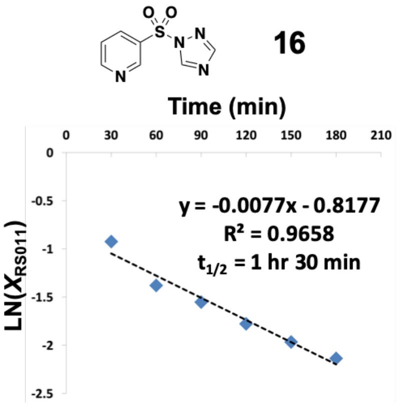

17

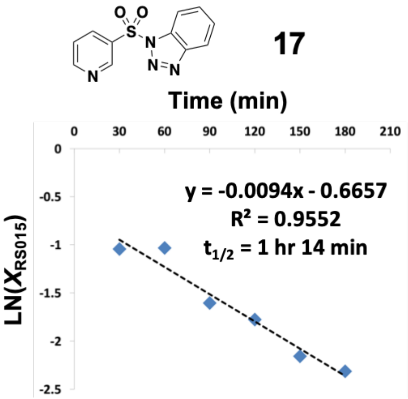

Figure S4. Hydrolysis half-life measurements of selected pyridine-3-sulfonyl reagents. Half-lives display a range from 4 min to over a week. **16** (**P3S**) with 1,2,4-triazole as leaving group is estimated to have a half-life of approximately 90 min, which is almost three times that of widely-used acyl imidazole reagent NAI. Hydrolysis of sulfonyl reagents is measured by analyzing changes in ^1H NMR spectra over 3 hr. NMR sample preparation: 10-20 mg of reagent is dissolved in 350 μL DMSO-d_6 and mixed with 350 μL of 50 mM pH 7.4 aqueous phosphate buffer evaporated to dryness and redissolved in D_2O. LN($X_{molecule}$) represents natural logarithm of the mole fraction of each molecule in the NMR mixture.

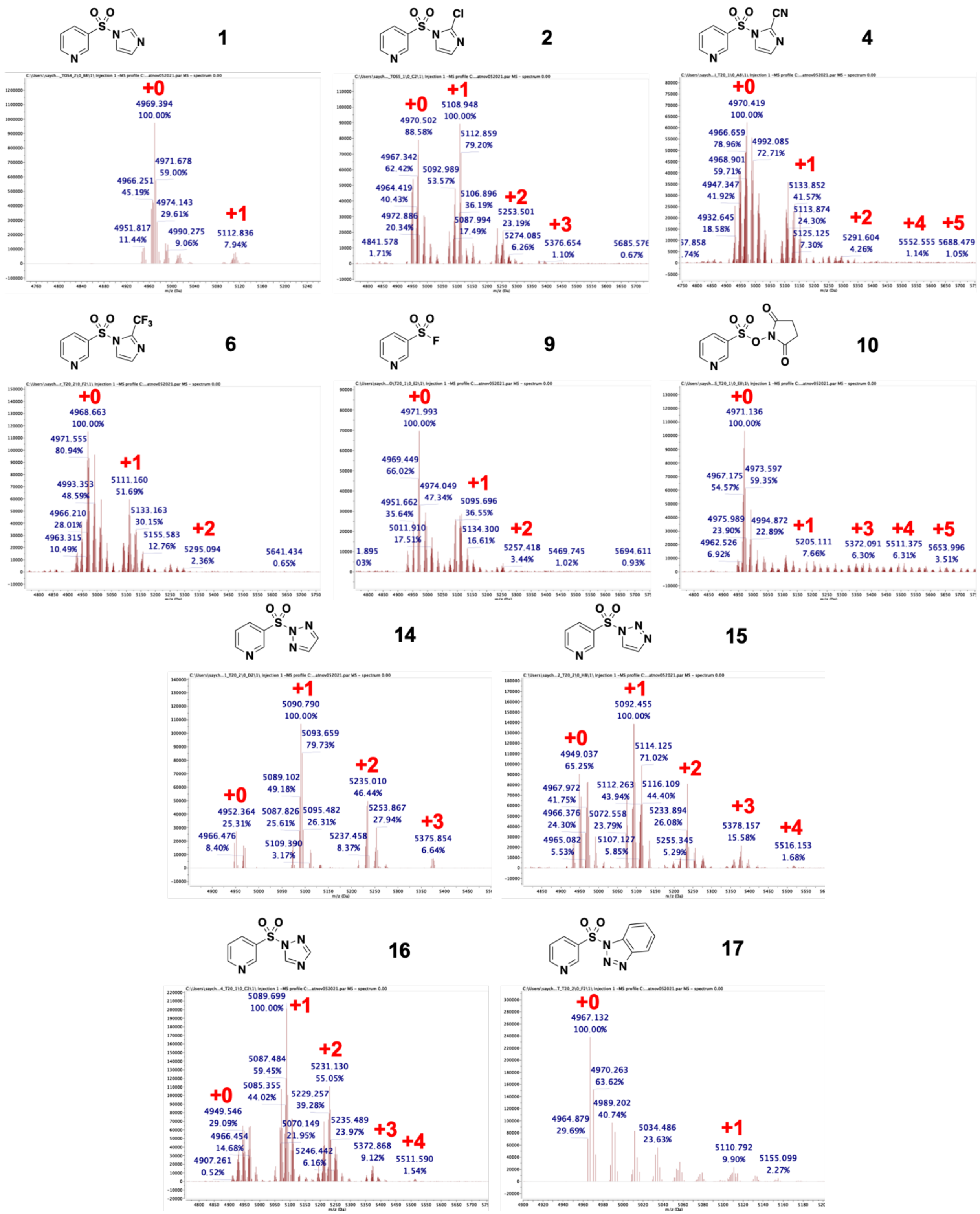

Figure S5. MALDI-TOF analysis of RNA 2′-OH modifying pyridine-3-sulfonyl reagents' reaction with Test RNA. Concentration of reagent in all reactions is 100 mM, except **17** (50 mM), **4** (unstable in DMSO, <100 mM), **15** (unstable in DMSO, <100 mM), and **2** (137 mM). Numbers in red indicate mass peaks corresponding to RNA molecules modified with indicated number of

sulfonyl modifications. Representative spectra shown, experiment repeated three times with similar results.
(Reaction conditions: 37°C, 24 h, volume = 10 μL, [Test] = 10 μM, [MOPS] = [NaCl] = 100 mM, [$MgCl_2$] = 6.06 mM, 20% DMSO, pH 7.5)

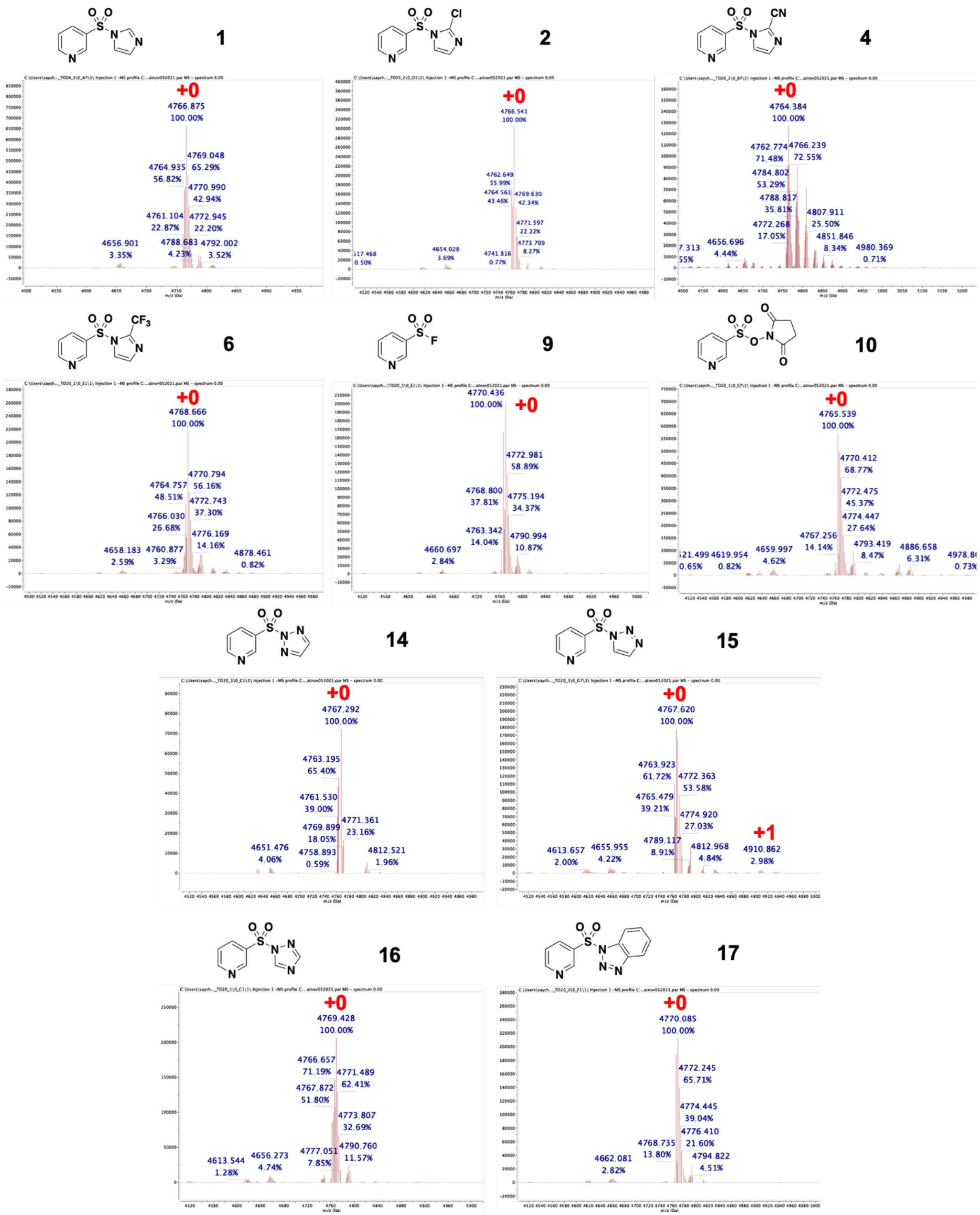

Figure S6. MALDI-TOF analysis of RNA 2′-OH modifying pyridine-3-sulfonyl reagents' reaction with Test DNA. Concentration of reagent in all reactions is 100 mM, except **17** (50 mM), **4** (unstable in DMSO, <100 mM), **15** (unstable in DMSO, <100 mM), and **2** (137 mM). Numbers in red indicate mass peaks corresponding to DNA molecules modified with indicated number of

sulfonyl modifications. These data indicate that reaction is specific and limited to RNA 2′-OH, not at exocyclic amines or phosphate backbone. Representative spectra shown, experiment repeated three times with similar results.
(Reaction conditions: 37°C, 24 h, volume = 10 μL, [Test DNA] = 10 μM, [MOPS] = [NaCl] = 100 mM, [$MgCl_2$] = 6.06 mM, 20% DMSO, pH 7.5)

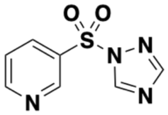

P3S

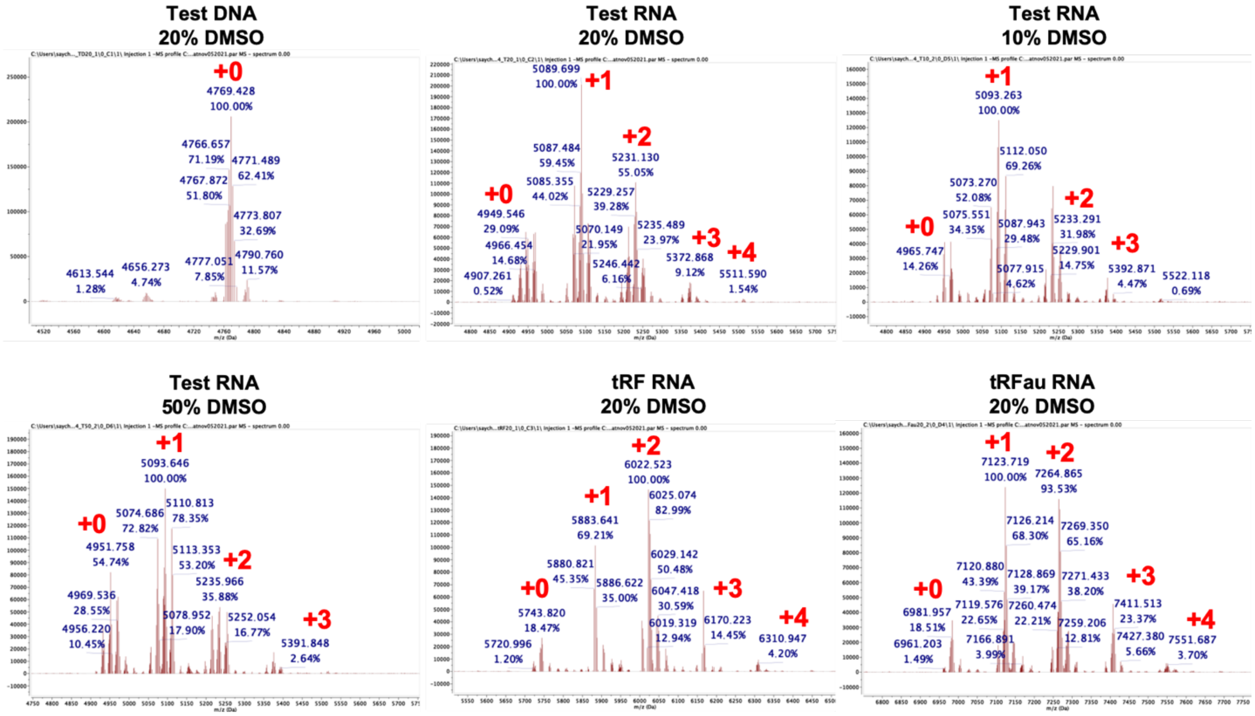

Figure S7. MALDI-TOF analysis of **P3S** reaction with Test DNA, Test, tRF, tRFau RNAs at different DMSO %. Concentration of reagent in all reactions is 100 mM. Numbers in red indicate mass peaks corresponding to DNA/RNA molecules modified with indicated number of sulfonyl modifications. These data indicate that conversion of RNA to 2′-OH sulfonylated products occurs with high yields under 20% and 10% DMSO conditions. Representative spectra shown, experiment repeated three times with similar results.
(Reaction conditions: 37°C, 24 h, volume = 10 μL, [RNA/DNA] = 10 μM, [MOPS] = [NaCl] = 100 mM, [$MgCl_2$] = 6.06 mM, pH 7.5)

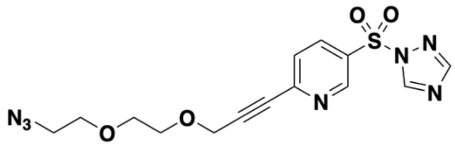

AzP3S

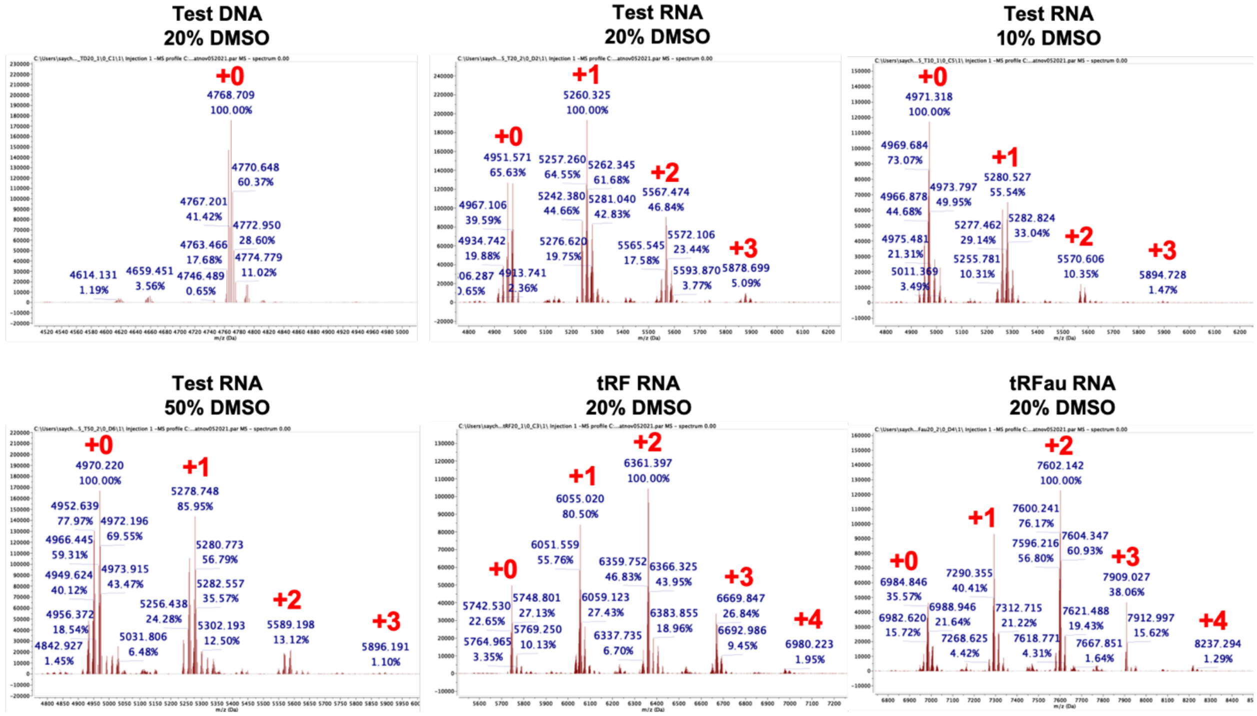

Figure S8. MALDI-TOF analysis of **AzP3S** reaction with Test DNA, Test, tRF, tRFau RNAs at different DMSO %. Concentration of reagent in all reactions is 100 mM. Numbers in red indicate mass peaks corresponding to DNA/RNA molecules modified with indicated number of sulfonyl modifications. These data indicate that conversion of RNA to 2′-OH sulfonylated products occur with high yields under 20% and 10% DMSO conditions. Representative spectra shown, experiment repeated three times with similar results.

(Reaction conditions: 37°C, 24 h, volume = 10 µL, [RNA/DNA] = 10 µM, [MOPS] = [NaCl] = 100 mM, [$MgCl_2$] = 6.06 mM, 20% DMSO, pH 7.5)

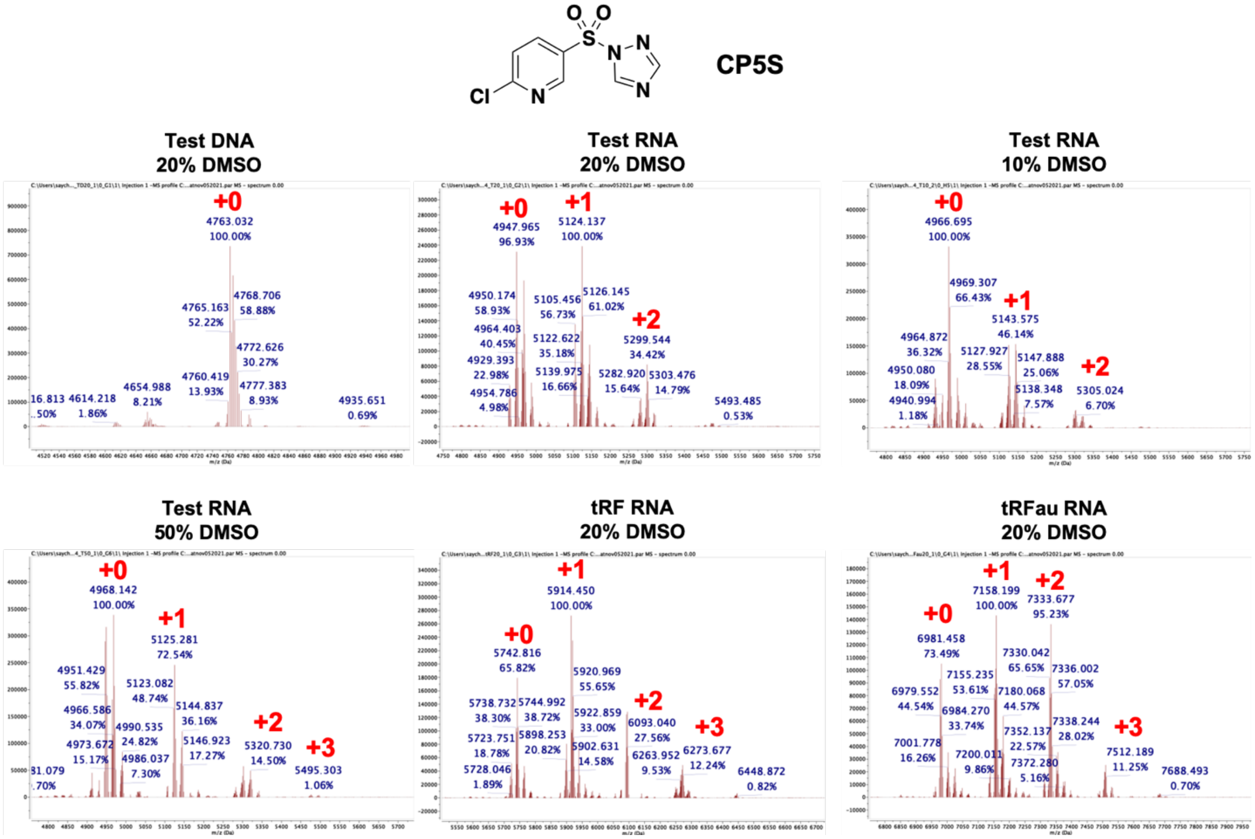

Figure S9. MALDI-TOF analysis of CP5S reaction with Test DNA, Test, tRF, tRFau RNAs at different DMSO %. Concentration of reagent in all reactions is 100 mM. Numbers in red indicate mass peaks corresponding to DNA/RNA molecules modified with indicated number of sulfonyl modifications. These data indicate that conversion of RNA to 2′-OH sulfonylated products occur with high yields under 20% and 10% DMSO conditions. Representative spectra shown, experiment repeated three times with similar results.
(Reaction conditions: 37°C, 24 h, volume = 10 μL, [RNA/DNA] = 10 μM, [MOPS] = [NaCl] = 100 mM, [$MgCl_2$] = 6.06 mM, pH 7.5)

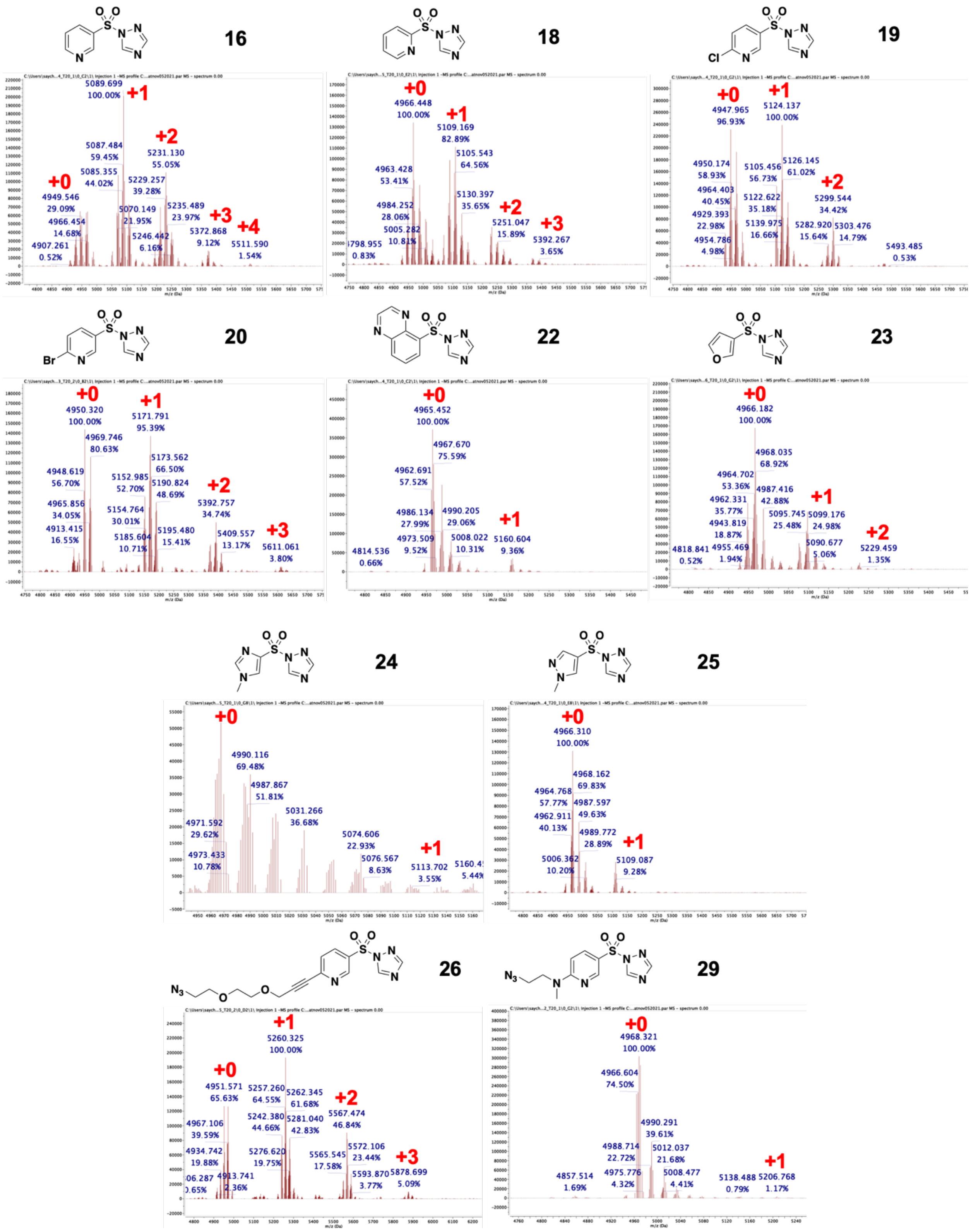

Figure S10. MALDI-TOF analysis of RNA 2′-OH modifying heterocyclic sulfonyl triazole reagents' reactions with Test RNA. Concentration of reagent in all reactions is 100 mM, except **22** (< 25 mM) and **24** (< 50 mM). Numbers in red indicate mass peaks corresponding to RNA molecules

modified with indicated number of sulfonyl modifications. Representative spectra shown, experiment repeated three times with similar results.
(Reaction conditions: 37°C, 24 h, volume = 10 μL, [Test RNA] = 10 μM, [MOPS] = [NaCl] = 100 mM, [$MgCl_2$] = 6.06 mM, 20% DMSO, pH 7.5)

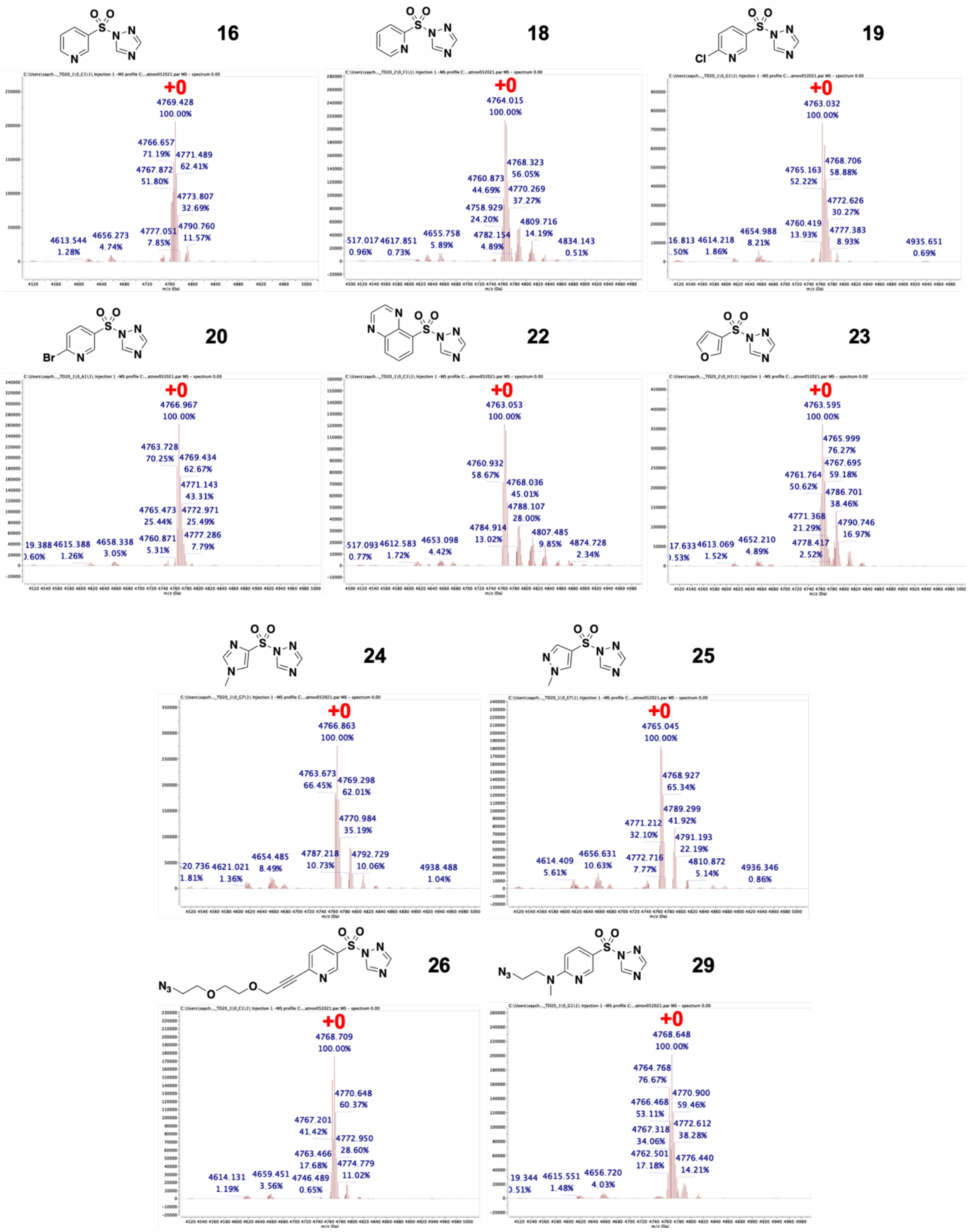

Figure S11. MALDI-TOF analysis of RNA 2′-OH modifying heterocyclic sulfonyl triazole reagents' reactions with Test DNA. Concentration of reagent in all reactions is 100 mM, except **22** (< 25 mM) and **24** (< 50 mM). Numbers in red indicate mass peaks corresponding to DNA molecules

modified with indicated number of sulfonyl modifications. These data indicate that reaction is specific and limited to RNA 2′-OH, not at exocyclic amines or phosphate backbone. Representative spectra shown, experiment repeated three times with similar results.
(Reaction conditions: 37°C, 24 h, volume = 10 μL, [Test DNA] = 10 μM, [MOPS] = [NaCl] = 100 mM, [$MgCl_2$] = 6.06 mM, 20% DMSO, pH 7.5)

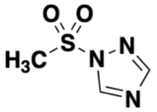

33

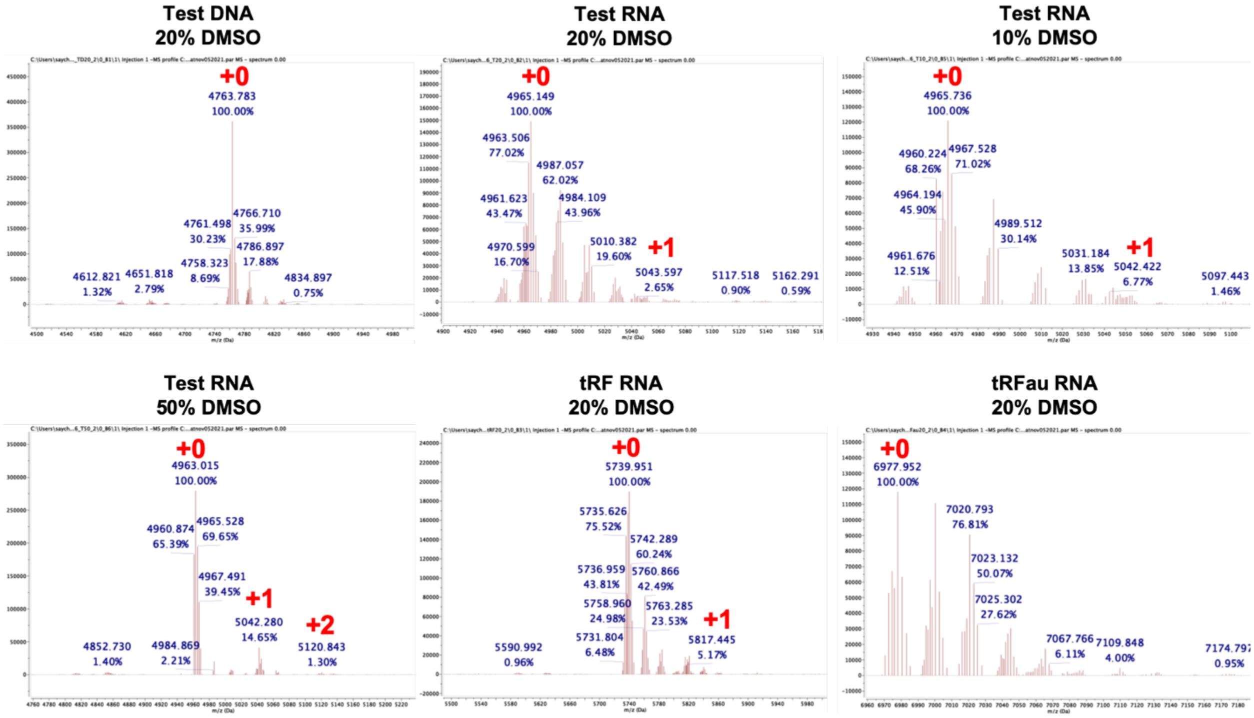

Figure S12. MALDI-TOF analysis of reactions of molecule **33** with Test DNA, Test, tRF, tRFau RNAs at different DMSO %. Concentration of reagent in all reactions is 100 mM. Numbers in red indicate mass peaks corresponding to DNA/RNA molecules modified with indicated number of sulfonyl modifications. These data indicate that conversion of RNA to 2′-OH sulfonylated products occur with high yields under 20% and 10% DMSO conditions. Representative spectra shown, experiment repeated three times with similar results.
(Reaction conditions: 37°C, 24 h, volume = 10 μL, [RNA/DNA] = 10 μM, [MOPS] = [NaCl] = 100 mM, [$MgCl_2$] = 6.06 mM, pH 7.5)

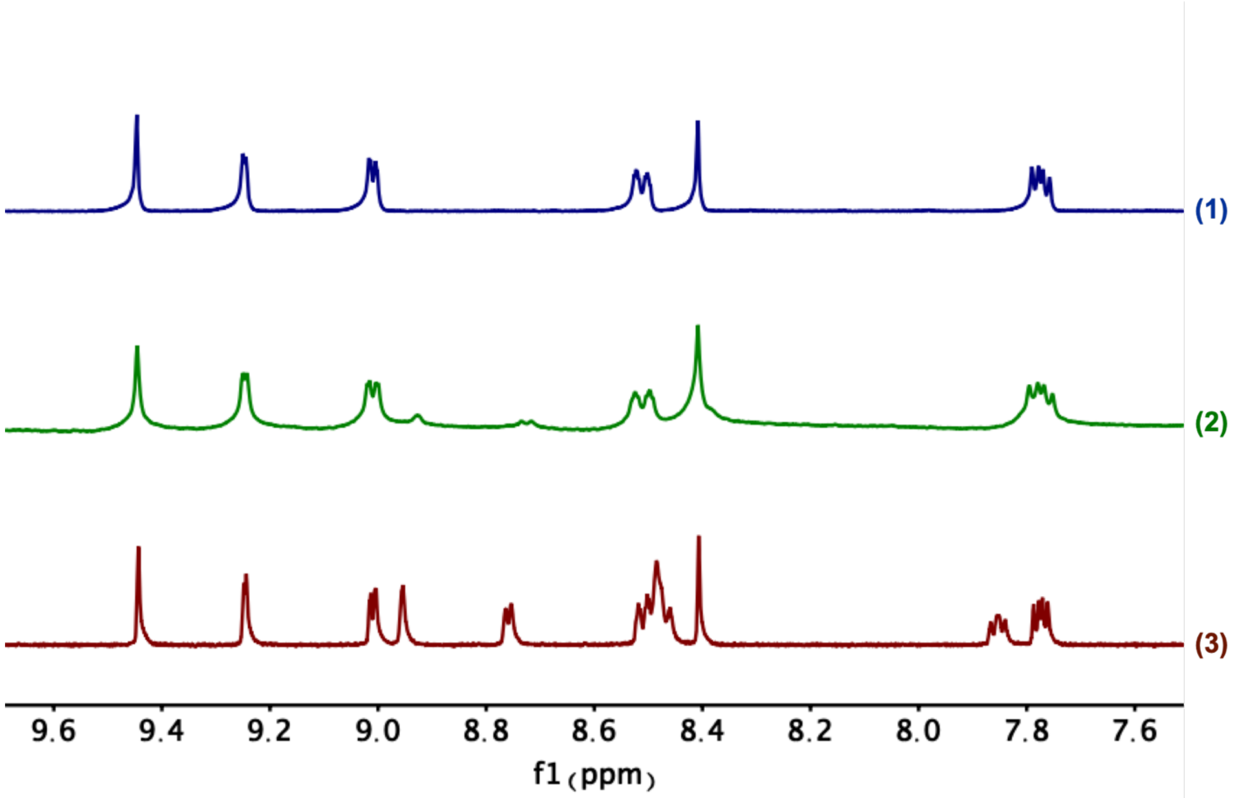

Figure S13. Stability analysis (by ^{1}H NMR in DMSO-d_6, 500 MHz; full spectra in NMR spectra section of this document) of **P3S** under Ar at ambient temperature. Spectrum (**1**): Freshly synthesized P3S after column purification; Spectrum (**2**): Same batch of **P3S** after 7 weeks' storage at ambient temperature (25°C) under Ar; Spectrum (**3**): Same batch of P3S after 6 months' storage at ambient temperature (25°C) under Ar. Spectrum **3** indicates 41% decomposition (by mole) of the sample of **P3S**. These results demonstrate the stability of **P3S** for weeks under common laboratory storage conditions.

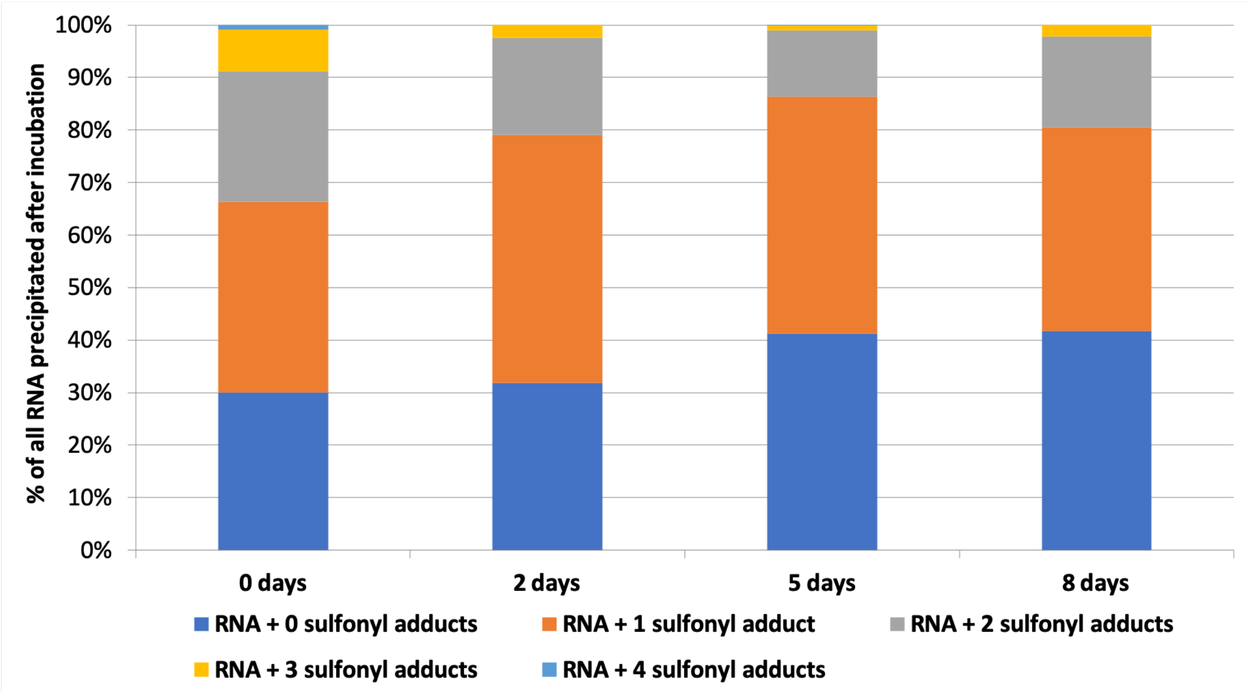

Figure S14. Stability analysis of **P3S** sulfonyl adducts on Test RNA. 100 picomoles of **P3S**-treated Test RNA was dissolved in 10 µL RNAse-free water in 200 µL PCR tubes and incubated for 0 days, 2 days, 5 days and 8 days at 37°C. Subsequent MALDI-TOF analysis (after ethanol precipitation) of the samples demonstrated minimal differences in the sulfonylation profile of the RNA samples, suggesting the stability of **P3S** adducts on RNA 2′-OH for at least 8 days at 37°C. Representative data, experiment repeated twice with similar results.
(Test RNA sulfonylation reaction conditions: 37°C, 24 h, volume = 10 µL, [RNA] = 10 µM, [**P3S**] = 100 mM, [MOPS] = [NaCl] = 100 mM, [$MgCl_2$] = 6.06 mM, 10% DMSO, pH 7.5)

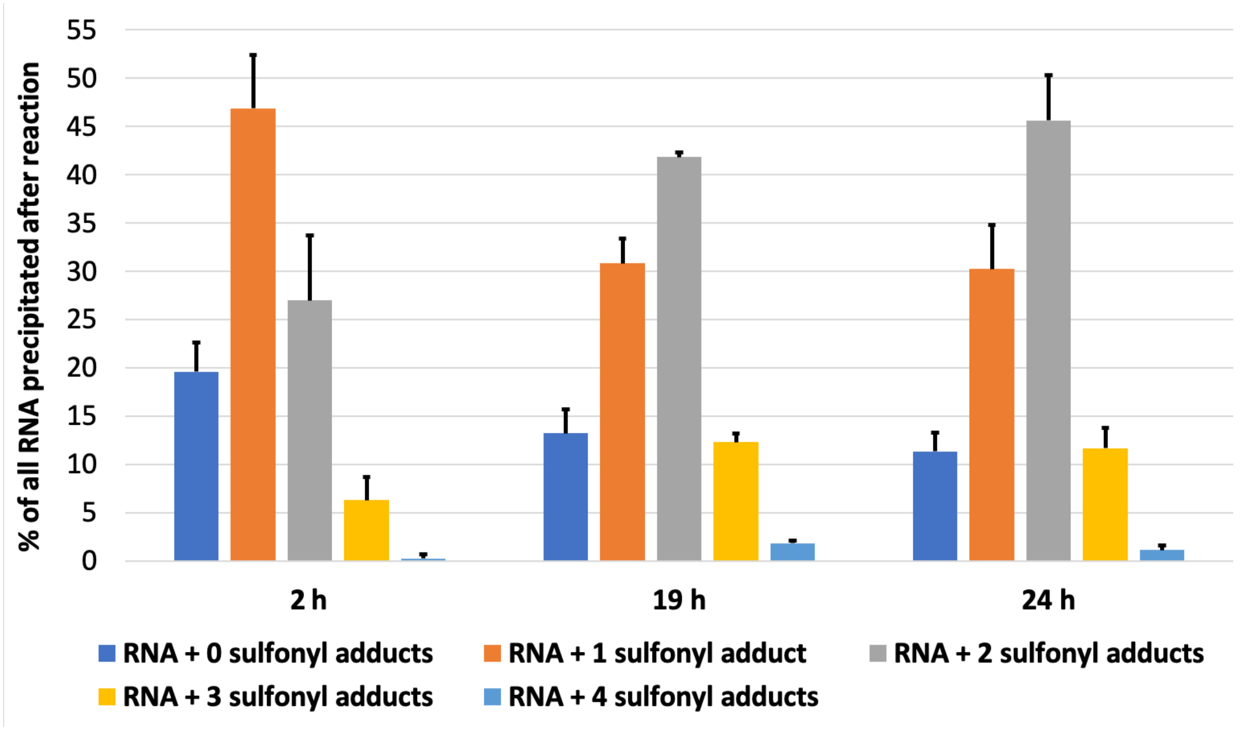

Figure S15. Time course of **P3S** reaction with tRF RNA. Sulfonylation profile is determined by MALDI-TOF analysis (after ethanol precipitation) of each RNA reaction, each timepoint is represented by the mean of the sulfonylation profile of 3 independent reactions. Error bars represent standard deviations. This data suggests that the yield of tRF RNA reaction with **P3S** levels off after 19 h. 24 h timepoint is chosen for most RNA covalent modification purposes (except structure analysis) for convenience.
(Reaction conditions: 37°C, 24 h, volume = 10 μL, [tRF RNA] = 10 μM, [**P3S**] = 100 mM, [MOPS] = [NaCl] = 100 mM, [$MgCl_2$] = 6.06 mM, 20% DMSO, pH 7.5)

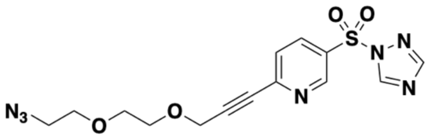

AzP3S

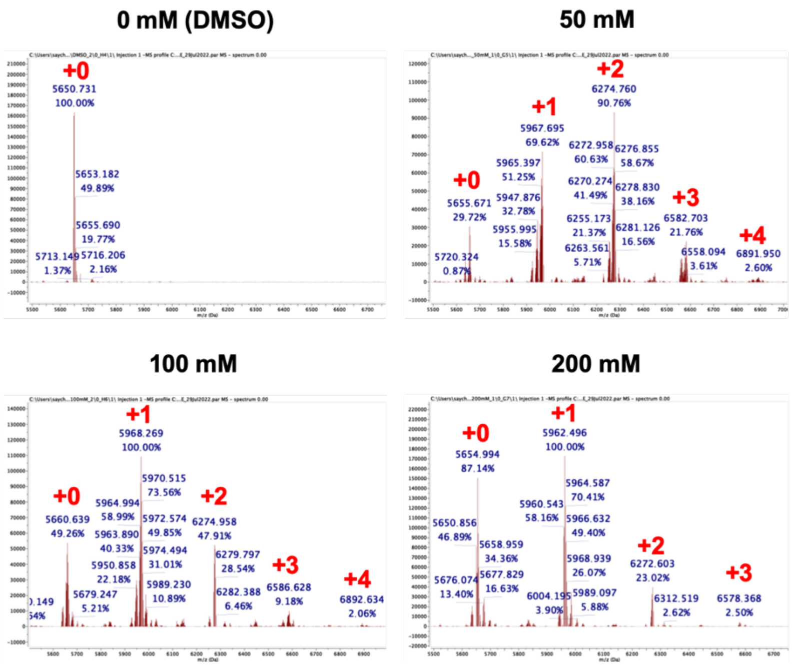

Figure S16. Analysis of tRF3 RNA reaction with **AzP3S** at different concentrations. The results demonstrate that the highest level of conversion of RNA into 2′-OH sulfonylated adducts among the tested conditions is achieved by utilizing 50 mM RS215 (87%). Representative spectra, experiment repeated twice with similar results.

(Reaction conditions: 37°C, 24 h, volume = 10 µL, [tRF3 RNA] = 10 µM, [MOPS] = [NaCl] = 100 mM, [$MgCl_2$] = 6.06 mM, 20% DMSO, pH 7.5)

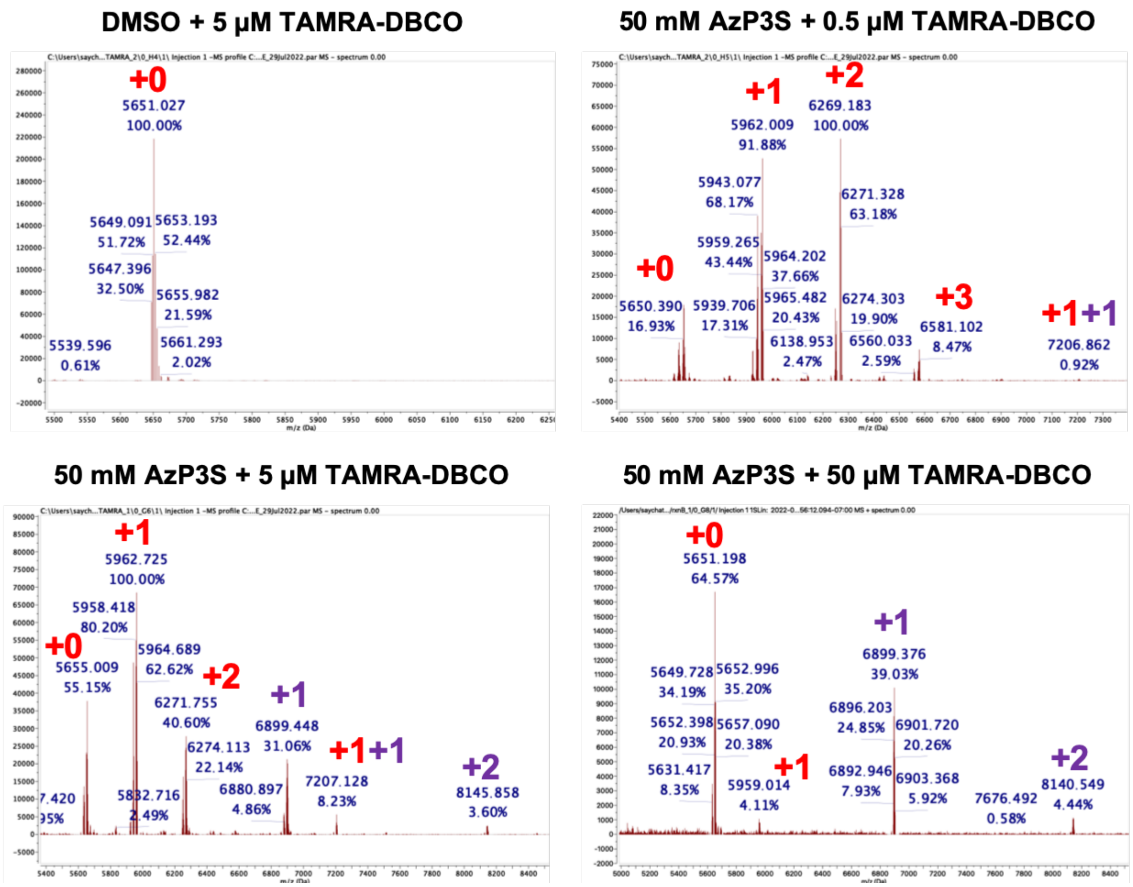

Figure S17. MALDI-TOF analysis of 50 mM **AzP3S**- (or DMSO-) treated tRF3 RNA reaction with TAMRA-DBCO in 1X PBS for 2 h. Numbers in red indicate peaks corresponding to RNA with indicated number of AzP3S adducts. Numbers in purple indicate peaks corresponding to RNA with indicated number of **AzP3S**+TAMRA adducts. These spectra demonstrate that with AzP3S-treated RNA, the yield of the click reaction with TAMRA-DBCO depends on the concentration of DBCO reagent; from 0.3% (0.5 μM TAMRA-DBCO) to 20% (5 μM TAMRA-DBCO) to 92% (50 μM TAMRA-DBCO). Representative spectra, experiment repeated twice with similar results.

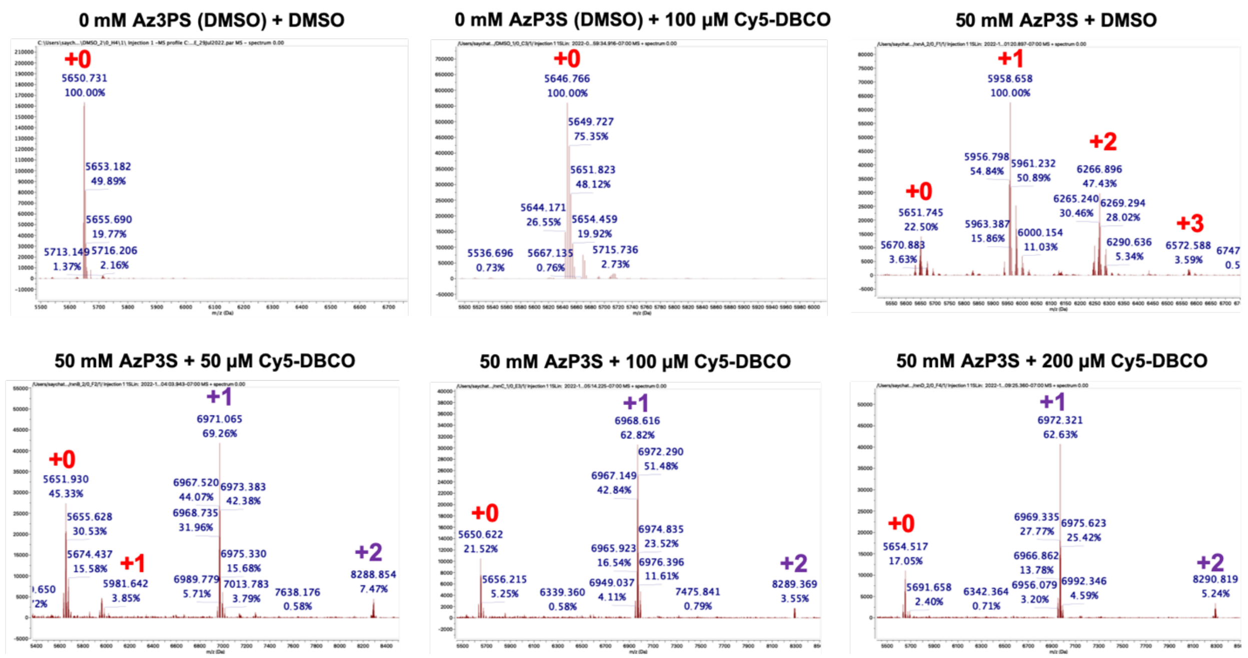

Figure S18. MALDI-TOF analysis of 50 mM **AzP3S**- (or DMSO-) treated tRF3 RNA reaction with Cy5-DBCO in 1X PBS for 2 h. Numbers in red indicate peaks corresponding to RNA with indicated number of **AzP3S** adducts. Numbers in purple indicate peaks corresponding to RNA with indicated number of **AzP3S**+Cy5 adducts. These spectra demonstrate that with **AzP3S**-treated RNA, the yield of the click reaction is nearly quantitative with 100 μM Cy5-DBCO reagent treatment. Representative spectra, experiment repeated twice with similar results.

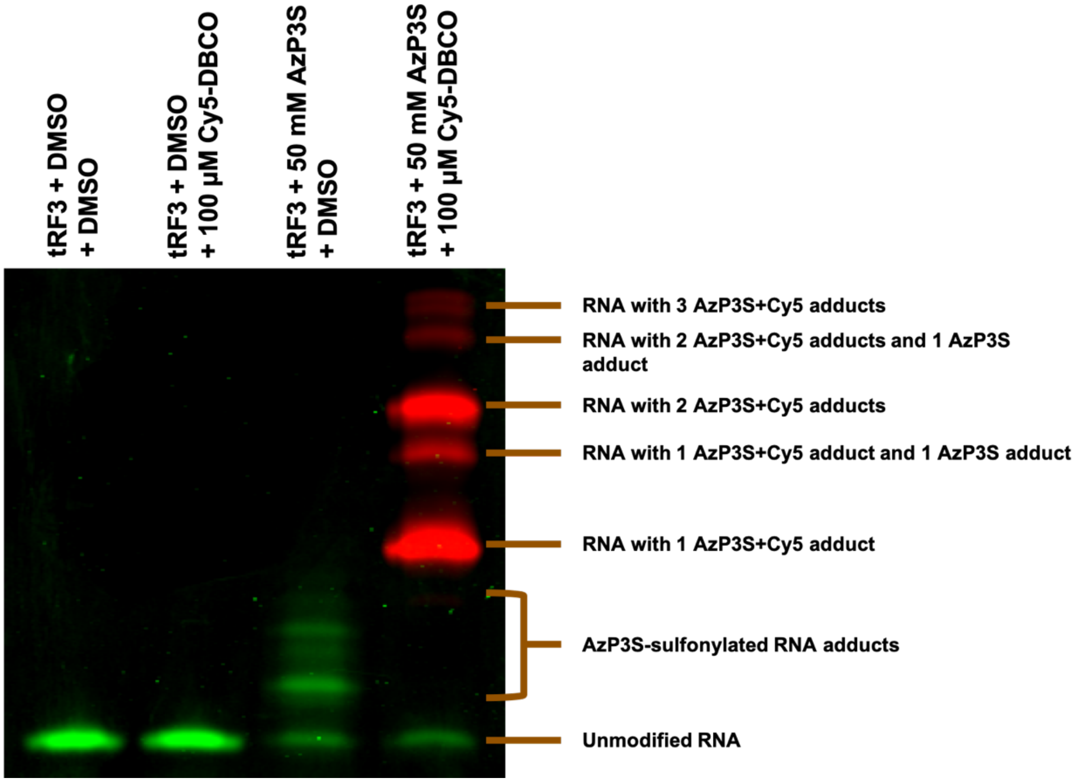

Figure S19. 20% denaturing urea PAGE gel analysis of 50 mM **AzP3S**- (or DMSO-) treated tRF3 RNA in a reaction with Cy5-DBCO (or DMSO) in 1X PBS pH 7.4 for 2 h. SYBR Gold staining (green, excitation at 488 nm) confirms the presence of RNA as well as **AzP3S** sulfonyl adducts on RNA. Imaging with Cy5 mode (blue, excitation at 633 nm) of the same gel confirms that the cycloaddition occurs to afford **AzP3S**+Cy5 adducts on RNA. These results indicate that RNA sulfonylated using azide-containing sulfonylating reagent **AzP3S** can be labeled with high yields under mild conditions. (**tRF3 + DMSO + DMSO**: tRF3 RNA reacted with DMSO under 20% DMSO conditions, and then reacted with DMSO under 20% DMSO conditions after ethanol precipitation. **tRF3 + DMSO + 100 µM Cy5-DBCO**: tRF3 RNA reacted with DMSO under 20% DMSO conditions, and then reacted with 100 µM Cy5-DBCO under 20% DMSO conditions after ethanol precipitation. **tRF3 + 50 mM AzP3S + DMSO**: tRF3 RNA reacted with 50 mM AzP3S under 20% DMSO conditions, and then reacted with DMSO under 20% DMSO conditions after ethanol precipitation. **tRF3 + 50 mM AzP3S + 100 µM Cy5-DBCO**: tRF3 RNA reacted with 50 mM AzP3S under 20% DMSO conditions, and then reacted with 100 µM Cy5-DBCO under 20% DMSO conditions after ethanol precipitation.)

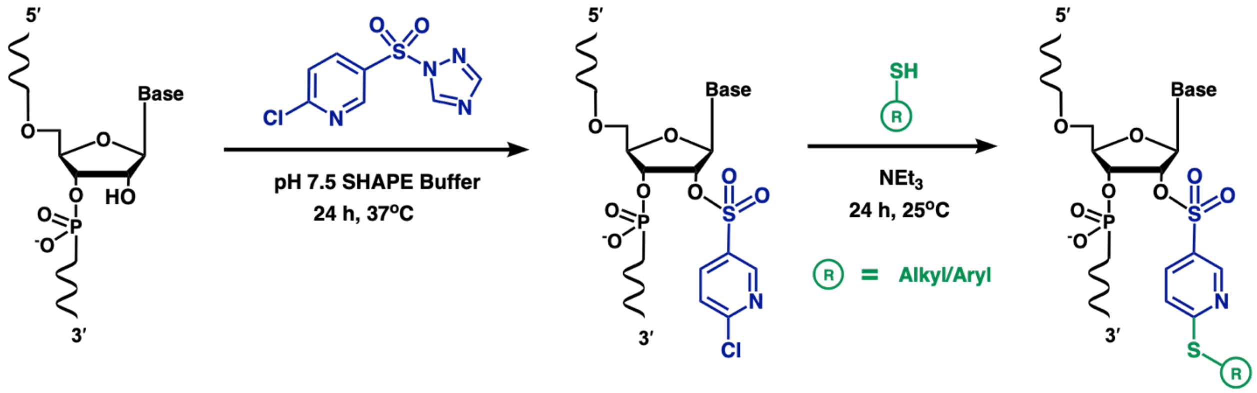

Figure S20. Scheme for S_NAr reaction of **CP5S**-sulfonylated RNA with simple alkyl/aryl thiols. For details on experiment, see “Experimental Procedures” in the next section.

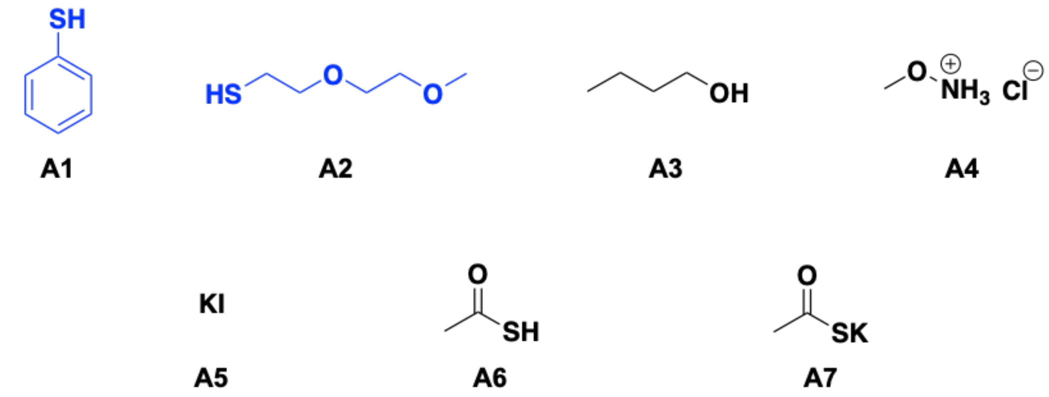

Figure S21. List of nucleophiles screened for S_NAr reaction with **CP5S**-sulfonylated RNA with simple alkyl/aryl thiols. Molecules in blue displayed SNAr adduct formation on RNA with > 95% yields (Figure S21, Figure S22). For details on experiment, see “Experimental Procedures” in the next section.

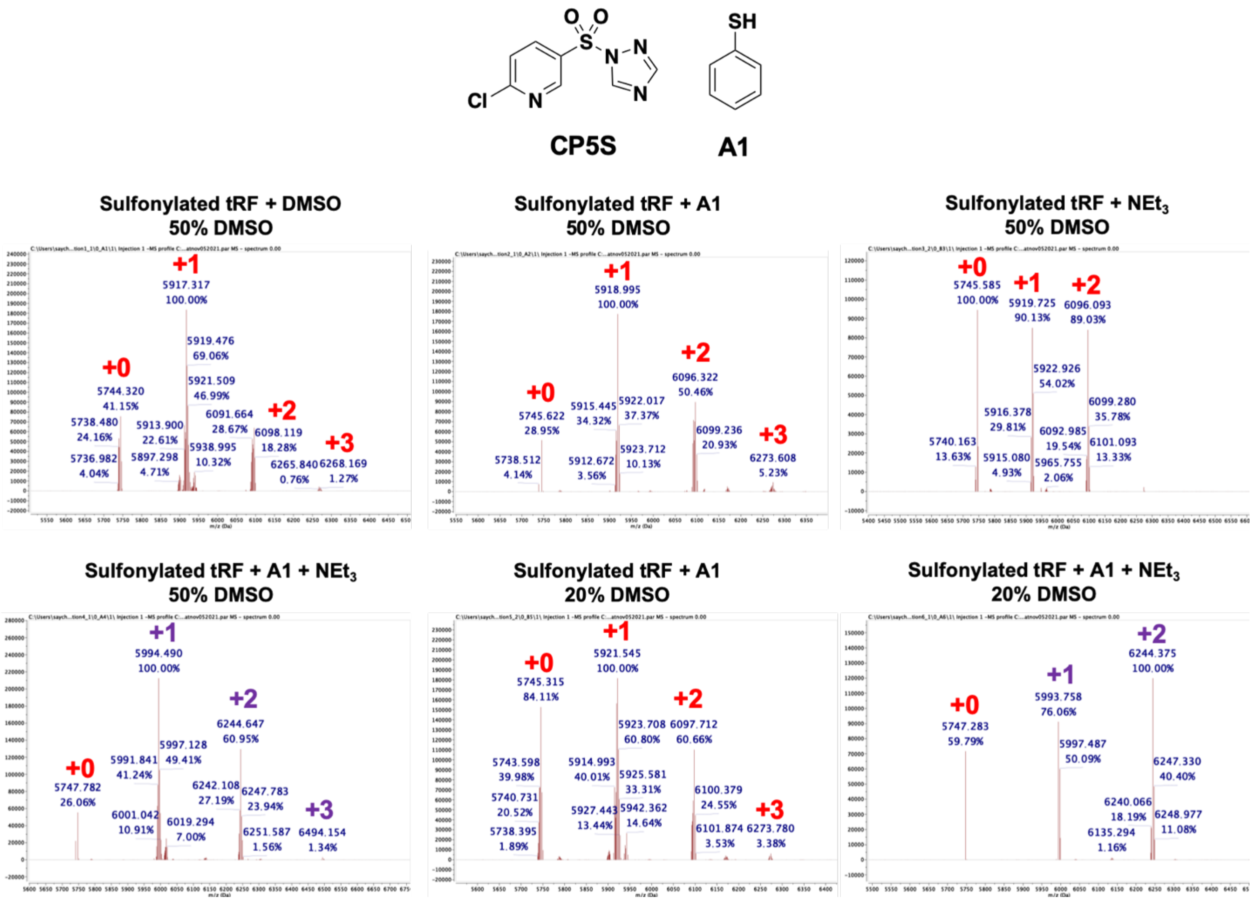

Figure S22. MALDI-TOF analysis of 100 mM **CP5S**-treated tRF RNA in a reaction with 100 mM **A1** in water-DMSO systems for 24 h. Numbers in red indicate peaks corresponding to RNA with indicated number of **CP5S** adducts. Numbers in purple indicate peaks corresponding to RNA with indicated number of **CP5S+A1** adducts These results indicate nearly quantitative conversion of **CP5S**-RNA adducts into **CP5S+A1** adducts after 24 h reaction in the presence of 100 mM NEt_3, under both 50% and 20% DMSO conditions. No RNA degradation was detected by MALDI. For detailed experimental procedure see next section “**Experimental Procedures**”.

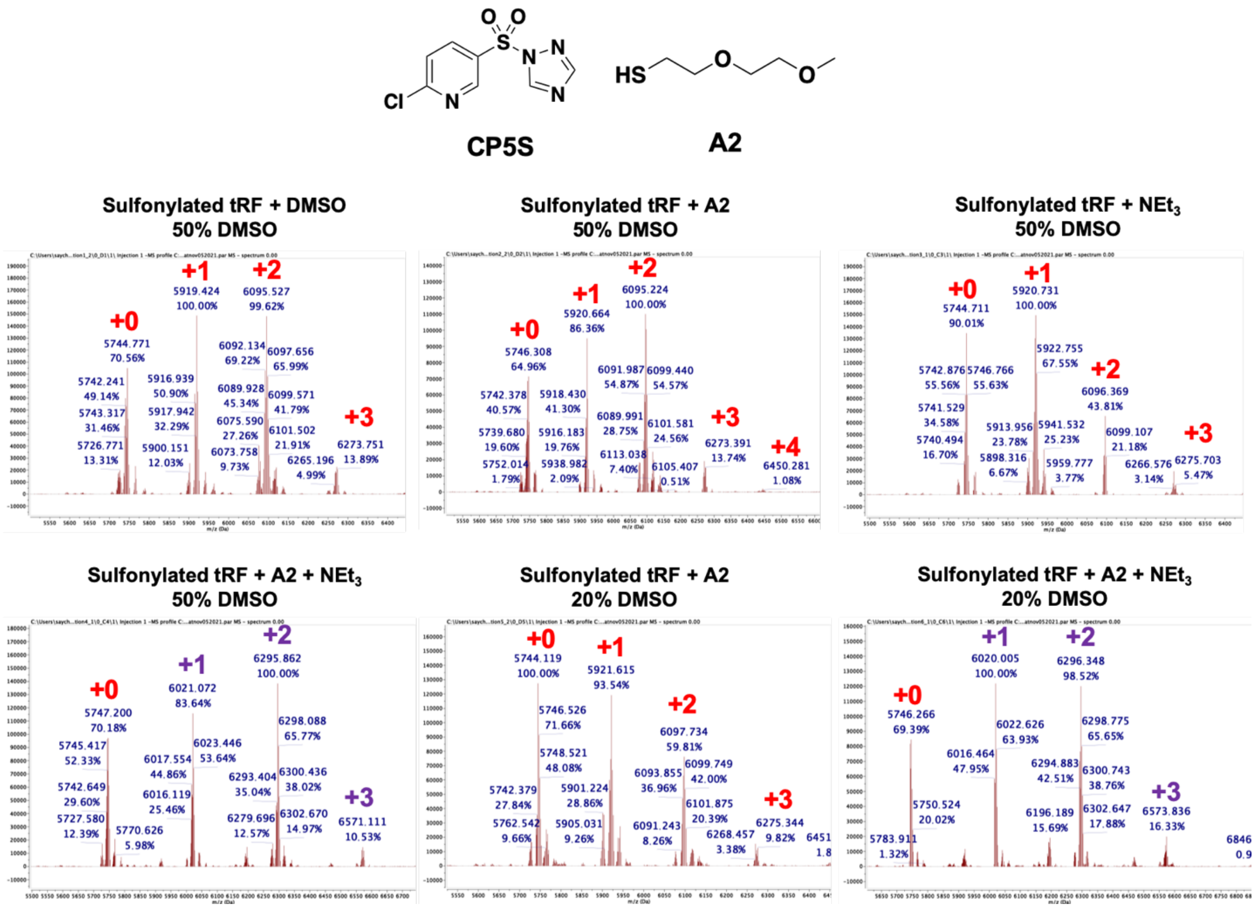

Figure S23. MALDI-TOF analysis of 100 mM **CP5S**-treated tRF RNA in a reaction with 100 mM **A2** in water-DMSO systems for 24 h. Numbers in red indicate peaks corresponding to RNA with indicated number of **CP5S** adducts. Numbers in purple indicate peaks corresponding to RNA with indicated number of **CP5S+A1** adducts These results indicate > 95% conversion of **CP5S**-RNA adducts into **CP5S+A1** adducts after 24 h reaction in the presence of 100 mM NEt_3, under both 50% and 20% DMSO conditions. No RNA degradation was detected by MALDI. For detailed experimental procedure see next section "**Experimental Procedures**".

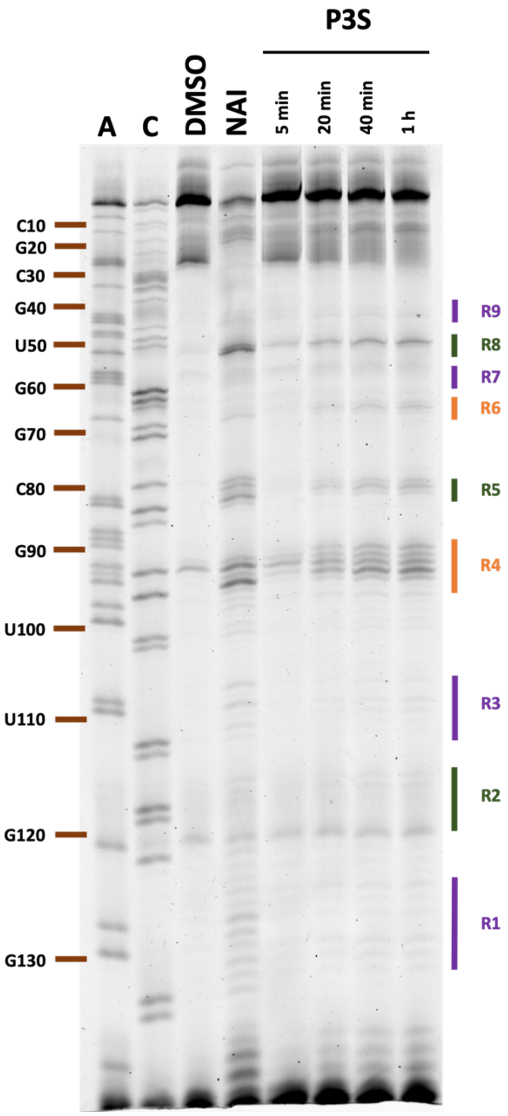

Figure S24. Time course experiment of *in vitro* RNA structure mapping using **P3S** (100 mM) reagent. The RNA used is the *E. coli* FMN riboswitch. The data documents an increase in the level of RNA covalent modification with time, over 5 min to ~40 min. The gel also suggests structure-sensitive RNA modification, as the location of reverse transcription-stop bands correlate to secondary structural features in the FMN riboswitch (Figure 5A). Except the incubation times for the RNA modification reactions with **P3S**, all other experimental details are as outlined in the "**Experimental Procedures**" section of this Supporting Information document.

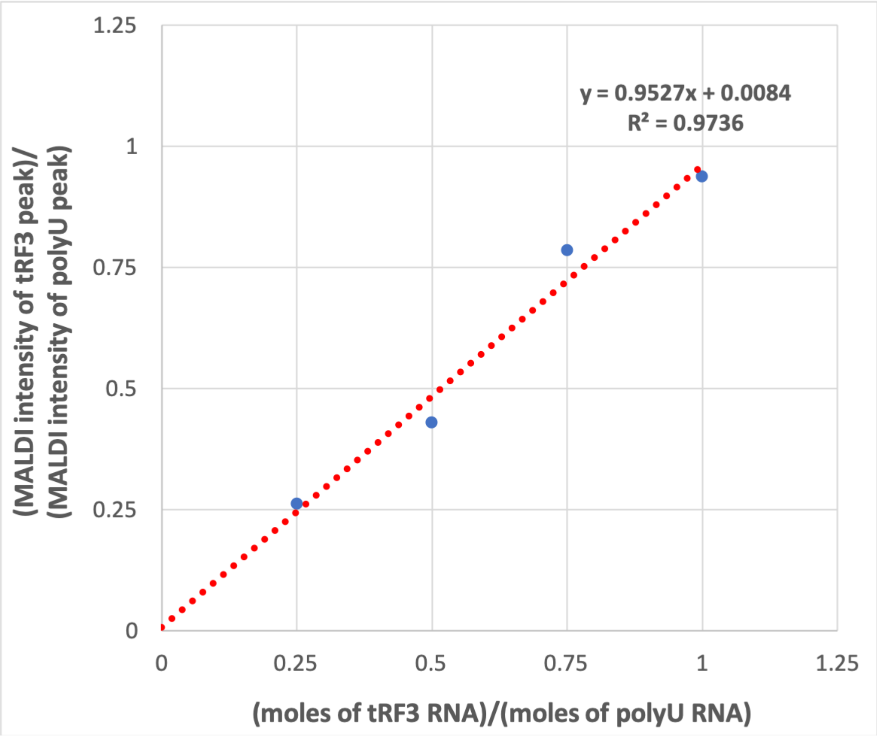

Figure S25. Investigating linearity of the relationship between MALDI-TOF peak intensity of an RNA species and the concentration of the RNA. Aqueous solutions of 20 μM polyU RNA (internal standard) were prepared and four such identical solutions were spiked with tRF3 RNA to final concentrations of 5 μM, 10 μM, 15μM and 20 μM. MALDI-TOF spectra were recorded following the procedure outlined in the "**Experimental Procedures**" section below. The ratio of MALDI peak intensities of tRF3 and polyU RNAs was found to be linearly related to the mole ratio of tRF3 and polyU RNA.

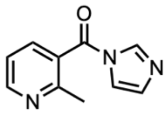

NAI

Test DNA
20% DMSO

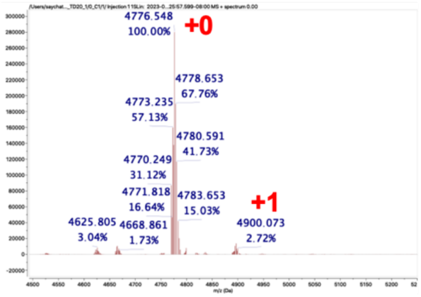

Test RNA
20% DMSO

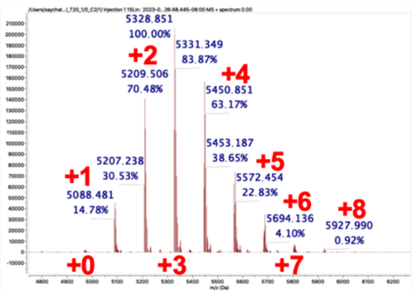

Test RNA
10% DMSO

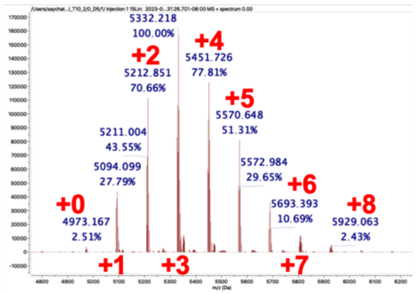

Test RNA
50% DMSO

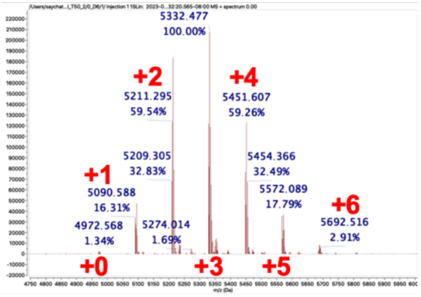

tRF RNA
20% DMSO

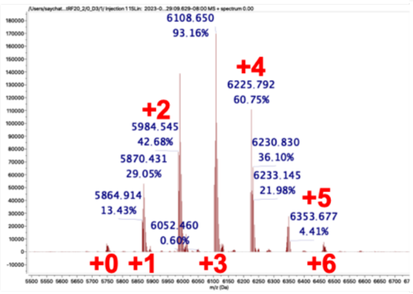

tRFau RNA
20% DMSO

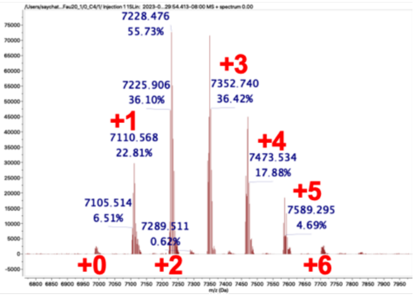

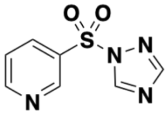

P3S

Test DNA
20% DMSO

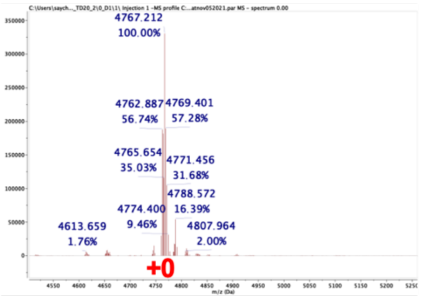

Test RNA
20% DMSO

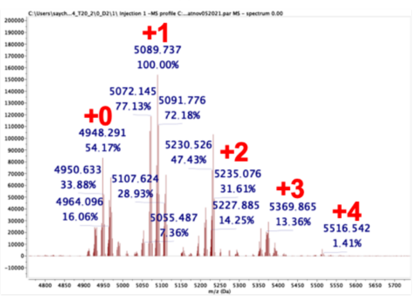

Test RNA
10% DMSO

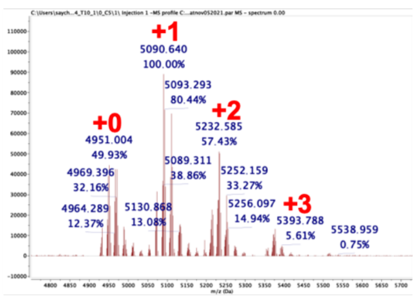

Test RNA
50% DMSO

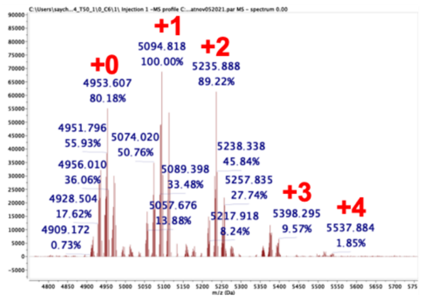

tRF RNA
20% DMSO

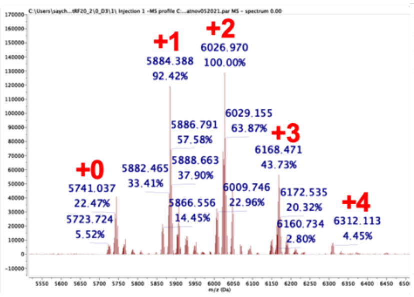

tRFau RNA
20% DMSO

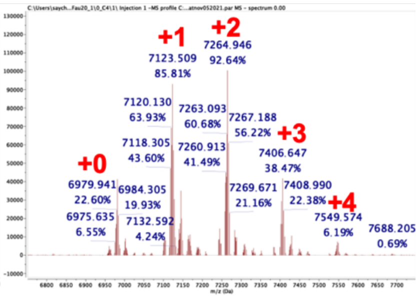

Figure S26. MALDI-TOF analysis of parallel reactions of molecule **NAI** and **P3S** with Test DNA, Test, tRF, and tRFau RNAs, showing relative reactivity of the two electrophiles under the same conditions. Concentration of reagent in all reactions is 100 mM; DMSO concentration is varied as shown. Numbers in red indicate mass peaks corresponding to DNA/RNA molecules modified with indicated number of acyl (in reactions with **NAI**) or sulfonyl (in reactions with **P3S**) modifications. These data indicate that the RNA 2′-OH modification efficiency of **NAI** is higher than that of **P3S**. The conversion of Test RNA into 2'-OH modified species by **NAI** is calculated to be greater than 99%, compared to 81% for **P3S** under the same conditions (20% DMSO).
Representative spectra shown; experiments repeated three times with similar results.
(Reaction conditions: 37°C, 24 h, volume = 10 μL, [RNA/DNA] = 10 μM, [MOPS] = [NaCl] = 100 mM, [$MgCl_2$] = 6.06 mM, pH 7.5)

Experimental Procedures

RNA Reactions with Sulfonylating Reagents: In a sterile 200 µL PCR tube, 3.3 µL of SHAPE 3.3X Buffer (333 mM MOPS pH 7.5, 333 mM NaCl, 20 mM $MgCl_2$ in water), was mixed with 5.7 µL 17.5 µM (for 10% DMSO reactions), 4.7 µL 21.3 µM (for 20% DMSO reactions) or 1.7 µL 58.8 µM (for 50% DMSO reactions) RNA stock solution. Fresh stocks of sulfonylating reagents were prepared in DMSO; and 1 µL of 1 M stock, 2 µL of 500 mM stock, or 5 µL of 200 mM stock were added to 10%, 20% and 50% DMSO reactions respectively. These reactions were incubated for 24 hr at 37°C and the RNA was subsequently purified by ethanol precipitation. The level of RNA modification was measured by MALDI-TOF M/S and analyzed using MestReNova™ software. Note: For every sulfonylating reagent, 6 parallel reactions were undertaken and analyzed with TestDNA, tRF, tRFau (20% DMSO), and Test (10%, 20%, 50% DMSO).

Ethanol precipitation of RNA reactions: For an RNA reaction with total volume X µL, 9X µL RNA precipitation solution (0.33 M NaOAc (pH 5.2) in water containing 0.2 mg/mL glycogen) was added and mixed well. 30X µL of ice-cold absolute ethanol was then added, and the mixture mixed by vortexing for at least 30 s. After storage at -80°C overnight, the mixture was centrifuged at 14.8k RPM for 60 mins at 4°C. The supernatant was discarded to obtain a pellet, which was washed with 70% ethanol. The obtained pellet was air dried for 15 min and subsequently either stored at -80°C for future use or dissolved in water/PBS for direct use in further experiments.

PCR amplification of FMN Riboswitch template: Crude FMN Riboswitch DNA Template was purchased from IDT Technologies and 5 ng of this was PCR amplified using Q5™ High-Fidelity 2X Master Mix following the manufacturer's protocol. The amplified product was purified using 1% agarose gel electrophoresis. PCR thermocycler program: 98°C 30 s (Step 1, 1 cycle); 98°C 10 s, 65°C 30 s, 72°C 30 s (Step 2, 30 cycles); 72°C 2 min (Step 3, 1 cycle)

***In vitro* transcription**: FMN Riboswitch RNAs were synthesized by *in vitro* transcription using the PCR amplified DNA Template and the HiScribe™ T7 Quick High Yield RNA Synthesis Kit (NEB) following the manufacturer's protocol. The reaction was incubated at 37°C overnight. Transcribed RNAs were purified by Quick-RNA MidiPrep™ kit following the manufacturer's protocol.

***In vitro* RNA SHAPE probing.** For transcribed FMN RNA structure probing, 500 ng FMN RNA was heated in the folding buffer (50mM NaCl) at 95°C for 2 min and then step-cooled to 37°C by 0.1 ℃/s. 3.3 µL 3.3X SHAPE buffer was added to the mixture. In a final 10 µL reaction 1 µL 2M, 1M, 0.5 M, 0.25 M P3S or 2 µL 1 M NAI stock solutions in DMSO was added. After incubation for 5 mins (NAI reaction) or 1 hr (P3S reactions) at 37°C, the reactions were purified by ethanol precipitation and the pellets used directly for further analysis. Note: parallel reaction was performed with just DMSO (no dissolved reagent) as a control for RNA modification. Longer times (6 hr, 24 hr) were investigated for P3S but no significant difference was observed over 1 hr timepoint.

***In vivo* RNA SHAPE probing.** Using DMEM supplemented by 10% FBS as media, HeLa cells were grown on 15 cm plates until they attained 90% confluence. After washing once with 10 mL

of 1X PBS, 4 mL of TrypLE Express™ was used to collect cells off the plate after incubation for 5 mins at 37°C. The cell suspension was transferred to a conical tube and the TrypLE inactivated by using 40 mL of cell media mixture. After centrifugation at 1000g for 2 min (25°C) and removal of supernatant, the cell pellet was resuspended in 1X PBS such that the concentration was $3x10^6$ cells in 900 µL. 900 µL of this suspension was transferred to sterile 1.5 mL tube and 100 µL 1M P3S or 100 µL 1M $NAI-N_3$ was added. These reactions were incubated at 37°C for 25 mins ($NAI-N_3$) or 1 hr (P3S). After such time points, each reaction was aliquoted into 500 µL of suspension and lysed with 6 mL of Trizol LS™ reagent by vortexing. To this mixture, 1.2 mL of chloroform was added and it was further vortexed. This mixture was incubated at 25°C for 5 min, after which centrifugation for 15 min at 4°C was performed to obtain phase separated layers. The aqueous phase was mixed with 1X volume of absolute ethanol and purified using a Quick-RNA MidiPrep™ kit following the manufacturer's protocol. Note: parallel reaction was performed with just DMSO (no dissolved reagent) as a control for RNA modification

PAGE analysis of reverse transcriptase (RT) stops. For FMN RNA experiment, 20 ng DMSO/P3S/NAI-treated RNA was mixed with 0.6 pmol Cy5-labeled RT Primer and 0.25 µL 10 mM dNTP mix (for sequencing lane, transcribed FMN RNA was directly used with no reagent treatment, ddNTP:dNTP=8:1), and incubated for 5 min at 65℃, then immediately chilled on ice for 2 min. 2 µL 5X First-Strand Buffer, 1 µL 0.1 M DTT, 0.25 µL RNaseOUT and 0.25 µL SuperScript II (200 U/µl) were added to the final volume of 10 µL. The reaction was incubated with the following program: 25℃ for 10 min, 42℃ for 50 min, and 52℃ for 50 min.
For human 5S rRNA *in vivo* SHAPE experiments, 1.6 µg isolated total RNA treated with $DMSO/P3S/NAI-N_3$ was mixed with 2 pmol Cy5-labeled 5S rRNA RT primer and 0.5 µL 10 mM dNTP mix (for sequencing lane, directly isolated RNA was used with no reagent treatment, ddNTP:dNTP=8:1), and incubated at 95℃ for 2 min; then cooled the samples to 4℃ by stepping down 2℃/s. 2 µL 5x First-Strand Buffer, 1 µL 0.1 M DTT, 0.25 µL RNaseOUT and 0.25 µL SuperScript III (200 U/µl) were added to the final volume of 10 µL. The reaction was incubated with the following program: 25℃ for 10 min, 52℃ for 50 min, and 55℃ for 50 min. After the reverse transcription reaction, 1 µL 1M NaOH was added and incubated at 95℃ for 5 min to remove the RNAs.
Finally, for both FMN RNA as well as human 5S RNA, 10 µL loading dye (8 M Urea, 0.05% Orange G, 0.05% Bromophenol blue) was added and the mixture was denatured at 95℃ for 3 min, and loaded on a denaturing or 8% polyacrylamide gel. Products were separated in a gel in 1x TBE (pH 8.3, Sigma Aldrich), 25mA, ~2.5 h. The cDNA gel was visualized by fluorescence imaging.

TAMRA-DBCO/Cy5-DBCO conjugation: In a sterile 200 µL PCR tube, 10 µM tRF3 RNA was treated with stock solution of AzP3S in DMSO (or just DMSO for control reactions) following the RNA sulfonylation protocol for 20% DMSO RNA reactions (section titled "**RNA Reactions with Sulfonylating Reagents"** above**)**. 50 picomoles of treated tRF3 RNA was then dissolved in 40 µL 1X PBS (pH 7.4), and incubated at room temperature for 10 mins. 10 µL of TAMRA-DBCO/Cy5-DBCO stock solution in DMSO was then added to the RNA solution, and mixed well. After incubation for 2 hr at 37°C, the samples were subjected to ethanol precipitation to isolate the RNA and remove unreacted TAMRA or Cy5 reagent. The obtained pellet was then either dissolved in water for MALDI-TOF and PAGE analysis, or 1X PBS for fluorimeter experiments.

Fluorimeter Measurements: 60 picomoles of TAMRA-DBCO-treated RNA was dissolved in 600 µL 1X PBS and transferred to a quartz cuvette. Using a Horiba Jobin-Yvon Spex Fluorolog-3™ fluorimeter and the FluoroEssence™ software, emission spectra was recorded with excitation at 546 nm, emission range 557-650 nm and slit width 5 nm. Three independent measurements were recorded for each condition tested.

Visualization of Cy5-labeled RNA on PAGE gel: tRF3 RNA treated with 50 mM AzP3S (or DMSO for control) was reacted with Cy5-DBCO (or DMSO for control) according to the protocol in the section titled "**TAMRA-DBCO/Cy5-DBCO conjugation**" above. The final RNA pellets for different treatment conditions were redissolved in RNAse-free water to obtain 1 µM stock solution. 10 µL of this stock solution was mixed with 10 µL loading dye (8 M Urea, 0.05% Orange G, 0.05% Bromophenol blue) and the mixture was denatured at 65°C for 2 min, and loaded on a denaturing 20% polyacrylamide gel. Products were separated in the gel in 1x TBE (pH 8.3, Sigma Aldrich), 25mA, ~2.5 h. The gel was subsequently incubated with 50 mL 1x SYBR Gold solution at ambient temperature, and visualized by fluorescence imaging using appropriate channels for Cy5 (excitation at 633 nm) or SyBR Gold (excitation at 488 nm) using a Typhoon GE scanner.

S_NAr reaction on CP5S-treated RNA with nucleophiles: tRF RNA was reacted with 100 mM **CP5S** as per the 20% DMSO RNA reaction protocol (outlined in the section **RNA Reactions with Sulfonylating Reagents** above). After precipitation, the final pellet was redissolved in RNAse-free water to obtain 50 µM stock solution. In 4 separate 200 µL PCR tubes, 2 µL of this stock solution was diluted with 3 µL (for 50% DMSO reactions) and 6 µL (for 20% DMSO reactions) water in 2 separate PCR tubes. For the 50% DMSO reactions, a total of 5 µL stock solutions of nucleophile and/or triethylamine (NEt_3) in DMSO were added, to create 10 µL reaction mixtures with the conditions: DMSO control, 100 mM nucleophile, 100 mM NEt_3, 100 mM nucleophile and 100 mM NEt_3. For the 20% DMSO reactions, a total of 2 µL stock solutions of nucleophile and/or triethylamine (NEt_3) in DMSO were added, to create 10 µL reaction mixtures with the conditions: 100 mM nucleophile, 100 mM nucleophile and 100 mM NEt_3. After incubation at ambient temperature (25°C) for 24 h, the reactions were worked up with ethanol precipitation and analyzed by MALDI-TOF MS.

MALDI-TOF MS: All MALDI-TOF spectra were recorded at the Stanford University Mass Spectrometry facility, using the Bruker Daltonik Microflex MALDI-TOF spectrometer equipped with an N_2 laser. All spectra were recorded in linear negative mode and samples were plated on an MSP Anchorchip 96 target plate. 0.3 M trihydroxyacetophenone in EtOH (matrix) and 0.1 M aqueous ammonium citrate (co-matrix) were mixed in a 2:1 ratio by volume to be used as a matrix mix for MALDI. This mix was always freshly prepared before analysis. After RNA precipitation, the RNA pellet was redissolved in RNAse-free water to prepare 10 µM sample solution. 1 µL of this solution was transferred to the target plate and dried under an Ar stream. 1 µL of the matrix mix was then added directly on top of the dried sample and completely dried under Ar stream. The spectral data was then recorded using Flex Control software (Bruker), and analyzed using MNova (Mestrenova).

NMR Spectroscopy: All NMR spectra were recorded at the Stanford University Department of Chemistry NMR facility. Varian 300 MHz, 400 MHz and 500 MHz NMR instruments were used to record the 1H and 13C spectra. The spectra were analyzed using MNova software.

ESI-MS: All ESI-MS spectra were recorded at the Stanford University Mass Spectrometry Facility. The instrument used was a Waters 2795 HPLC system with dual wavelength UV detector, and ZQ single quadrupole MS with electrospray ionization source

Synthesis of reagents

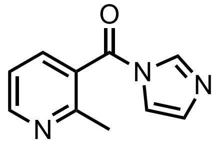

NAI

Synthesized according to the procedure previously reported in the literature.[2]

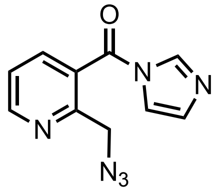

NAI-N_3

Synthesized according to the procedure previously reported in the literature.[3]

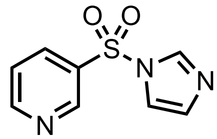

1

In a dry 25 mL round-bottom flask equipped with a stirrer and placed in an ice bath, 200 mg of pyridine-3-sulfonyl chloride (1.13 mmol) was dissolved in 5.63 mL anhydrous dichloromethane. 383 mg (5.63 mmol) of imidazole and 392 uL (291 mg, 2.25 mmol) of N,N-Diisopropylethylamine were added sequentially at 0°C and stirred for 5 min. The ice bath was removed and the reaction was stirred overnight (18 h) at ambient temperature (25°C). After the reaction, the mixture was concentrated in a rotary evaporator. The residue was purified by silica gel column chromatography (hexane : ethyl acetate = 100 : 0 to 40 : 60) to afford **1** (137 mg, 0.655 mmol, 58.1%) as a white solid.

^{1}H NMR (300 MHz, DMSO-d_6) δ 9.28 (s, 1H), 8.97 (s, 1H), 8.50 (d, *J* = 8.4 Hz, 1H), 8.44 (s, 1H), 7.85 (s, 1H), 7.75 (m, 1H), 7.17 (s, 1H)

^{13}C NMR (100 MHz, DMSO-*d*) δ 155.65, 147.71, 137.38, 135.51, 134.14, 131.65, 124.91, 118.24

ESI-MS [M+H]: Calculated: 210.03; Observed: 210.10

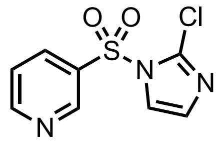

2

To a solution of pyridine-3-sulfonyl chloride (100 mg, 0.56 mmol) in anhydrous acetonitrile (2.8 mL) was added 2-chloroimidazole (173 mg, 1.69 mmol) and stirred for 30 minutes at ambient temperature under argon. Then the reaction mixture was added triethylamine (64

μL, 0.62 mmol) and stirred for 18 hours. After the reaction, the mixture was diluted with chloroform, filtered and washed with chloroform. The filtrate was washed with water and brine. The organic layer was dried over anhydrous sodium sulfate, filtered and concentrated in vacuo. The residue was purified by silica gel column chromatography (hexane : ethyl acetate = 99 : 1 to 50 : 50) to afford **3** (136 mg, 0.56 mmol, quant.) as a white solid.

^{1}H NMR (400 MHz, DMSO-*d*) δ 9.27 (d, *J* = 3.0 Hz, 1H), 9.03 (d, *J* = 4.7 Hz, 1H), 8.50 (d, *J* = 7.8 Hz, 1H), 7.96 (d, *J* = 1.9 Hz, 1H), 7.80 (dd, *J* = 8.5, 4.3 Hz, 1H), 7.16 (dd, *J* = 3.9, 1.9 Hz, 1H)

^{13}C NMR (100 MHz, DMSO-*d*) δ 156.20, 148.21, 136.22, 132.89, 129.70, 129.13, 125.12, 122.37

ESI-MS [M+H]: Calculated: 243.99; Observed: 244.08

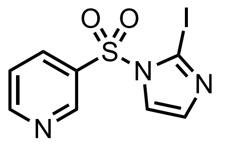

3

To a solution of pyridine-3-sulfonyl chloride (100 mg, 0.56 mmol) in anhydrous acetonitrile (2.8 mL) was added 2-iodoimidazole (91 mg, 1.69 mmol) and stirred for 30 minutes at ambient temperature under argon. Then the reaction mixture was added triethylamine (64 μL, 0.62 mmol) and stirred for 18 hours. After the reaction, the mixture was diluted with chloroform, filtered and washed with chloroform. The filtrate was washed with water and brine. The organic layer was dried over anhydrous sodium sulfate, filtered and concentrated in vacuo. The residue was purified by silica gel column chromatography (chloroform : ethyl acetate = 67 : 33 to 50 : 50) to afford **3** (120 mg, 0.56 mmol, quant.) as a white solid.

^{1}H NMR (400 MHz, DMSO-*d*) δ 9.25 (d, *J* = 2.1 Hz, 1H), 9.01 (d, *J* = 5.0 Hz, 1H), 8.43 (d, *J* = 8.5 Hz, 1H), 8.01 (d, *J* = 1.5 Hz, 1H), 7.79 (dd, *J* = 8.0, 5.0 Hz, 1H), 7.16 (dd, *J* = 1.2 Hz, 1H)

^{13}C NMR (75 MHz, DMSO-*d*) δ 155.90, 148.14, 135.99, 133.42, 133.14, 124.99, 123.80, 89.70

ESI-MS [M+H]: Calculated: 335.93; Observed: 336.04

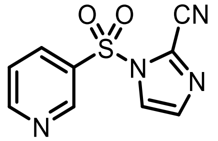

4

To a solution of pyridine-3-sulfonyl chloride (67 μL, 0.56 mmol) in anhydrous dichloromethane (2.8 mL) was added 1H-Imidazole-2-carbonitrile hydrochloride salt (218 mg, 1.69 mmol) and stirred for 30 minutes at ambient temperature under argon. Then the reaction mixture was added triethylamine (174 μL, 1.24 mmol) and stirred for 3 hours. After the reaction, the mixture was diluted with dichloromethane and washed with water and brine. The organic layer was dried over anhydrous sodium sulfate, filtered and concentrated in vacuo. The residue was purified by silica gel column

chromatography (dichloromethane : ethylacetate = 75 : 25) to afford the **4** (67 mg, 0.28 mmol, 51%) as a white solid.
^{1}H NMR (400 MHz, DMSO-d_6) δ 9.32 (d, *J* = 1.4 Hz, 1H), 9.05 (d, *J* = 4.7 Hz, 1H), 8.55 (d, *J* = 7.7 Hz, 1H), 8.35 (d, *J* = 1.6 Hz, 1H), 7.83 (d, *J* = 8.2, 5.0 Hz, 1H), 7.50 (s, 1H)
ESI-MS [M+H]: Calculated: 235.02; Observed: 235.07
^{13}C NMR could not be measured due to poor stability in any NMR solvent.

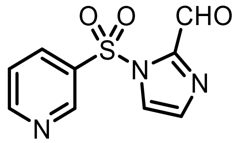

5

To a solution of pyridine-3-sulfonyl chloride (67 μL, 0.56 mmol) in anhydrous dichloromethane (2.8 mL) was added 2-formylimidazole (163 mg, 1.69 mmol) and stirred for 30 minutes at ambient temperature under argon. Then the reaction mixture was added triethylamine (87 μL, 0.62 mmol) and stirred for 1.5 hours. After the reaction, the mixture was diluted with dichloromethane and washed with water and brine. The organic layer was dried over anhydrous sodium sulfate, filtered and concentrated in vacuo. The residue was purified by silica gel column chromatography (dichloromethane : ethyl acetate = 75 : 25) to afford **5** (123 mg, 0.51 mmol, 92%) as a white solid.
^{1}H NMR (400 MHz, DMSO-*d*) δ 9.65 (s, 1H), 8.93 (s, 1H), 8.72 (d, *J* = 5.1 Hz, 1H), 8.39 (d, *J* = 8.0 Hz, 1H), 7.77-7.80 (m, 1H), 7.49 (s, 2H)
ESI-MS [M+H]: Calculated: 238.02; Observed: 238.09
^{13}C NMR could not be measured due to poor stability in any NMR solvent.

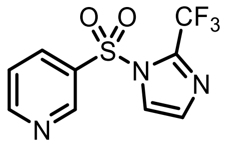

6

To a solution of pyridine-3-sulfonyl chloride (100 mg, 0.56 mmol) in anhydrous acetonitrile (2.8 mL) was added 2-(trifluoromethyl)-1H-imidazole (230 mg, 1.69 mmol) and stirred for 30 minutes at ambient temperature under argon. Then the reaction mixture was added triethylamine (83 μL, 0.59 mmol) and stirred for 8 hours. After the reaction, the mixture was diluted with chloroform, filtered and washed with chloroform. The filtrate was washed with water and brine. The organic layer was dried over anhydrous sodium sulfate, filtered and concentrated in vacuo. The residue was purified by silica gel column chromatography (chloroform : ethyl acetate = 80 : 20 to 67 : 33) to afford **6** (152 mg, 0.55 mmol, 97%) as a white solid.
^{1}H NMR (400 MHz, DMSO-*d*) δ 9.28 (d, *J* =2.5 Hz, 1H), 9.01 (dd, *J* = 4.8, 1.5 Hz, 1H), 8.51-8.53 (m, 1H), 8.35 (d, *J* = 1.7 Hz, 1H), 7.77-7.81 (m, 1H), 7.40 (d, *J* = 1.7 Hz, 1H)
^{13}C NMR (75 MHz, Chloroform-*d*) δ 155.80, 148.90, 135.86, 134.39, 129.16, 124.31, 123.81, 123.36, 119.47, 115.87.
ESI-MS [M+H]: Calculated: 278.02; Observed: 278.06

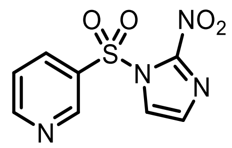

7

To a solution of pyridine-3-sulfonyl chloride (67 µL, 0.56 mmol) in anhydrous dichloromethane (2.8 mL) was added 2-nitroimidazole (192 mg, 1.69 mmol) and stirred for 30 minutes at ambient temperature under argon. Then the reaction mixture was added triethylamine (87 µL, 0.62 mmol) and stirred for 1.5 hours. After the reaction, the mixture was diluted with dichloromethane and washed with water and brine. The organic layer was dried over anhydrous sodium sulfate, filtered and concentrated in vacuo. The residue was purified by silica gel column chromatography (dichloromethane : ethyl acetate = 90 : 10) to afford **7** (84 mg, 0.32 mmol, 58%) as a white solid.

^{1}H NMR (400z MHz, DMSO-*d*) δ 8.96 (s, 1H), 8.75 (d, *J* = 4.9 Hz, 1H), 8.47 (d, *J* = 8.1 Hz, 1H), 7.86 (dd, *J* = 8.5, 5.8 Hz, 1H), 7.40 (s, 2H)

ESI-MS [M+H]: Calculated: 255.01; Observed: 255.09

^{13}C NMR could not be measured due to poor stability in any NMR solvent.

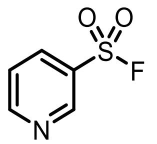

9

Synthesized according to the procedure previously reported in the literature.[4]

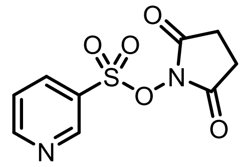

10

To a solution of pyridine-3-sulfonyl chloride (67 µL, 0.56 mmol) in anhydrous dichloromethane (2.8 mL) was added N-hydroxy succinimide (65 mg, 0.56 mmol) and stirred for 30 minutes at ambient temperature under argon. Then the reaction mixture was added triethylamine (87 µL, 0.62 mmol) and stirred for 15 minutes. After the reaction, the mixture was diluted with ethyl acetate and washed with water and brine. The organic layer was dried over anhydrous sodium sulfate, filtered and concentrated in vacuo. The residue was purified by silica gel column chromatography (dichloromethane : hexane = 83 : 17 to afford **10** (105 mg, 0.41 mmol, 73%.) as a white solid.

^{1}H NMR (400 MHz, DMSO-*d6*) δ 9.14 (dd, *J* = 1.9 Hz, 1H), 9.01 (dd, *J* = 4.7, 1.5 Hz, 1H), 8.47 (d, *J* = 7.9 Hz, 1H), 7.75 (dd, *J* = 7.8, 4.9 Hz, 1H), 2.70 (s, 1H)

^{13}C NMR (100 MHz, DMSO-*d6*) δ 167.73, 156.04, 149.00, 137.21, 130.66, 124.74, 25.52.

ESI-MS [M+H]: Calculated: 257.02; Observed: 257.10

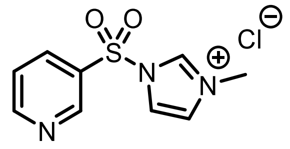

11
To a solution of 1-Methylimidazole (456 μL, 5.69 mmol) in anhydrous ethyl acetate (56 mL) was slowly added pyridine-3-sulfonyl chloride (683 μL, 5.64 mmol) at ambient temperature under argon. The reaction mixture was stirred at 70℃ for 1 hour and concentrated in vacuo. The residue was washed with 33% ethyl acetate in hexane and recrystallized with acetonitrile and ethyl acetate to afford **11** (1.16 g, 4.47 mmol, 79%) as a white slurry.
^{1}H NMR (400 MHz, DMSO-*d*) δ 9.08 (s, 1H), 8.90 (s, 1H), 8.74 (d, *J* = 5.4 Hz, 1H), 8.39 (d, *J* = 7.7 Hz, 1H), 7.78-7.81 (m, 1H), 7.69 (s, 1H), 7.66 (s, 1H), 3.86 (s, 3H)
^{13}C NMR (100 MHz, DMSO-*d*) δ 145.74, 144.40, 140.86, 140.01, 135.68, 126.33, 123.18, 229.57, 35.46.
LCMS could not be measured due to poor solubility in water/acetonitrile systems.

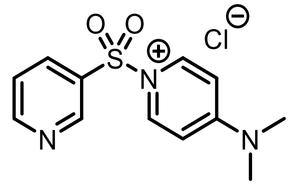

12
To a solution of N,N-dimethyl-4-aminopyridine (702 mg, 5.64 mmol) in anhydrous ethyl acetate (56 mL) was slowly added pyridine-3-sulfonyl chloride (683 μL, 5.64 mmol) at ambient temperature under argon. The reaction mixture was stirred for 30 minutes, and the white precipitate was filtered off, washed with cold ethyl acetate and hexane to afford **12** (1.14 g, 3.81 mmol, 68%) as a white solid.
^{1}H NMR (300 MHz, DMSO-*d*) δ 8.94 (s, 1H), 8.80 (d, *J* = 3.8 Hz, 1H), 8.53 (d, *J* = 7.7 Hz, 1H), 8.21 (d, *J* = 5.6 Hz, 2H), 7.89-7.94 (m, 1H), 6.97 (d, *J* = 6.5 Hz, 2H), 3.17 (s, 6H)
^{13}C NMR (100 MHz, DMSO-*d*) δ 156.92, 146.35, 143.24, 141.32, 139.81, 138.95, 126.79, 106.95, 39.64
ESI-MS [M+]: Calculated: 264.08; Observed: 264.17

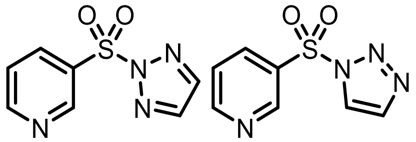

14, **15** (left, right)
To a solution of pyridine-3-sulfonyl chloride (67 μL, 0.56 mmol) in anhydrous dichloromethane (2.8 mL) was added 1,2,3-triazole (98 μL, 1.69 mmol) and stirred for 30 minutes at ambient temperature under argon. Then the reaction mixture was added triethylamine (87 μL, 0.62 mmol) and stirred for 3 hours. After the reaction, the mixture was diluted with ethyl acetate and washed with water and brine. The organic layer was dried over anhydrous sodium sulfate, filtered and concentrated in vacuo. The residue was purified by silica gel column chromatography (dichloromethane : ethyl acetate = 90 : 10) to afford the **14** (96 mg, 0.45 mmol, 81%) and **15** (12 mg, 56 μmol, 10%) as respectively a white solid.
15:

^{1}H NMR (400 MHz, Chloroform-*d*) δ 9.29 (d, *J* = 1.8 Hz, 1H), 8.89 (d, *J* = 4.9 Hz, 1H), 8.37 (d, *J* = 8.1 Hz, 1H), 7.89 (s, 2H), 7.52 (dd, *J* = 8.41, 4.77 Hz, 1H)
^{13}C NMR (100 MHz, Chloroform-*d*) δ 155.48, 149.26, 139.15, 136.55, 133.16, 124.21.
ESI-MS [M+H]: Calculated: 211.02; Observed: 211.08
16: ^{1}H NMR (400 MHz, DMSO-*d*) δ 9.30 (d, *J* = 1.7 Hz, 1H), 9.03 (s, 1H), 9.02 (s, 1H), 8.55 (d, *J* = 7.6 Hz, 1H), 8.07 (s, 1H), 7.78 (dd, *J* = 8.1, 4.8 Hz, 1H)
^{13}C NMR and LCMS could not be measured due to poor stability in any NMR solvent and water/acetonitrile systems.

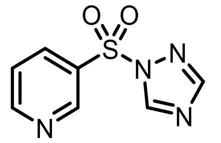

16 (P3S)
To a solution of pyridine-3-sulfonyl chloride (67 µL, 0.56 mmol) in anhydrous dichloromethane (2.8 mL) was added 1,2,4-triazole (117 mg, 1.69 mmol) and stirred for 30 minutes at ambient temperature under argon. Then the reaction mixture was added triethylamine (87 µL, 0.62 mmol) and stirred for 3 hours. After the reaction, the mixture was diluted with ethyl acetate and washed with water and brine. The organic layer was dried over anhydrous sodium sulfate, filtered and concentrated in vacuo. The residue was purified by silica gel column chromatography (dichloromethane : ethyl acetate = 83 : 17 to 75 : 25) to afford **16** (120 mg, 0.56 mmol, quant.) as a white solid.
^{1}H NMR (400z MHz, DMSO-*d*) δ 9.45 (s, 1H), 9.24 (d, *J* = 2.5 Hz, 1H), 9.00 (dd, *J* = 4.8, 1.5 Hz, 1H), 8.50-8.53 (m, 1H), 8.41 (s, 1H), 7.77 (dd, *J* = 8.4, 4.9 Hz, 1H)
^{13}C NMR (100 MHz, Chloroform-*d*) δ 155.94, 154.86, 149.34, 144.87, 136.54, 133.02, 124.25.
ESI-MS [M+H]: Calculated: 211.02; Observed: 211.09

Gram scale synthesis of 16 (P3S)
To a solution of pyridine-3-sulfonyl chloride (671 µL, 5.64 mmol) in anhydrous acetonitrile (28 mL) was added 1,2,4-triazole (585 mg, 16.9 mmol) and stirred for 30 minutes at ambient temperature under argon. Then the reaction mixture was added triethylamine (413 µL, 5.92 mmol) and stirred for 18 hours. After the reaction, the mixture was concentrated in vacuo and the residue was diluted with chloroform, washed with water and brine. The organic layer was dried over anhydrous sodium sulfate, filtered and concentrated in vacuo to afford the **16** (1.2 g, calc. 0.63 mmol, 89% purity with 11% of pyridine-3-sulfonic acid) as a white solid without further purification.

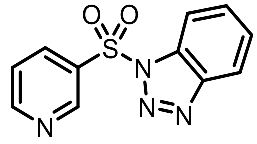

17
To a solution of pyridine-3-sulfonyl chloride (67 µL, 0.56 mmol) in anhydrous dichloromethane (2.8 mL) was added benzotriazole (202 mg, 1.69 mmol) and stirred for

30 minutes at ambient temperature under argon. Then the reaction mixture was added triethylamine (87 µL, 0.62 mmol) and stirred for 3 hours. After the reaction, the mixture was diluted with ethyl acetate and washed with water and brine. The organic layer was dried over anhydrous sodium sulfate, filtered and concentrated in vacuo. The residue was purified by silica gel column chromatography (dichloromethane : hexane = 50 : 50 to 95 : 5) to afford **18** (151 mg, 0.56 mmol, quant.) as a white solid.
^{1}H NMR (300 MHz, DMSO-*d*) δ 9.33 (d, *J* = 2.6 Hz, 1H), 8.96 (dd, *J* = 4.9, 1.6 Hz, 1H) , 8.54-8.58 (m, 1H) , 8.28 (d, *J* = 8.2 Hz, 1H), 8.19 (d, *J* = 8.6 Hz, 1H), 7.85 (dd, *J* = 8.5, 7.7 Hz, 1H), 7.71 (dd, *J* = 8.0, 4.7 Hz, 1H), 7.64 (dd, *J* = 7.7, 7.63 Hz, 1H)
^{13}C NMR (100z MHz, DMSO-*d*) δ 156.27, 147.79, 144.91, 135.97, 132.81, 131.44, 130.76, 126.78, 125.17, 120.67, 111.81.
ESI-MS [M+H]: Calculated: 261.04; Observed: 261.11

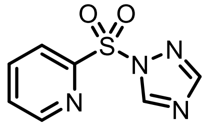

18
To a solution of pyridine-2-sulfonyl chloride (100 mg, 0.56 mmol) in anhydrous acetonitrile (5.6 mL) was added 1,2,4-triazole (117 mg, 1.69 mmol) and stirred for 30 minutes at ambient temperature under argon. Then the reaction mixture was added triethylamine (83 µL, 0.62 mmol) and stirred for 16 hours. After the reaction, the mixture was diluted with chloroform, filtered and washed with chloroform. The filtrate was washed with water and brine. The organic layer was dried over anhydrous sodium sulfate, filtered and concentrated in vacuo to afford **18** (95 mg, 0.45mmol, 80%) as a white solid.
^{1}H NMR (400 MHz, DMSO-*d*) δ 9.52 (s, 1H), 8.75 (d, J = 3.8 Hz, 1H), 8.38 (s, 1H), 8.36 (dd, J = 6.9 Hz, 1H), 8.25 (ddd, J = 7.9, 7.8, 1.7 Hz, 1H), 7.84-7.87 (m, 1H)
^{13}C NMR (125 MHz, DMSO-*d*) δ 154.87, 152.44, 151.19, 148.15, 139.90, 129.82, 124.01.
ESI-MS [M+H]: Calculated: 211.02; Observed: 211.10

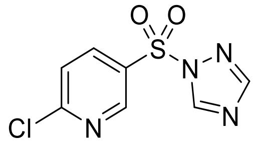

19 (CP5S)
To a solution of 6-chloropyridine-3-sulfonyl chloride (100 mg, 0.94 mmol) in anhydrous acetonitrile (9.4 mL) was added 1,2,4-triazole (195 mg, 2.82 mmol) and stirred for 30 minutes at ambient temperature under argon. Then the reaction mixture was added triethylamine (138 µL, 0.99 mmol) and stirred for 16 hours. After the reaction, the mixture was diluted with chloroform, filtered and washed with chloroform. The filtrate was washed with water and brine. The organic layer was dried over anhydrous sodium sulfate, filtered and concentrated in vacuo. The residue was purified by silica gel column chromatography (hexane : ethyl acetate = 83 : 17 to 75 : 25) to afford **19** (233 mg, 0.95 mmol, quant.) as a white solid.
^{1}H NMR (400 MHz, DMSO-*d*) δ 9.23 (s, 1H), 8.79 (s, 1H), 8.34 (s, 1H), 9.14 (d, J = 2.6 Hz, 1H), 8.53 (dd, J = 8.6, 2.7 Hz, 1H), 8.43 (s, 1H), 7.90 (d, J = 8.6 Hz, 1H)

^{13}C NMR (75 MHz, Chloroform-*d*) δ 158.77, 154.99, 149.83, 144.86, 138.78, 131.83, 125.47.
ESI-MS [M+H]: Calculated: 244.99; Observed: 245.01

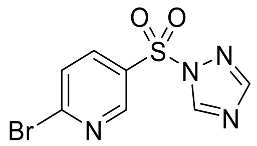

20

To a solution of 6-bromopyridine-3-sulfonyl chloride (248 mg, 0.97 mmol) in anhydrous acetonitrile (9.7 mL) was added 1,2,4-triazole (200 mg, 2.90 mmol) and stirred for 30 minutes at ambient temperature under argon. Then the reaction mixture was added triethylamine (142 μL, 1.2 mmol) and stirred for 2.5 hours. After the reaction, the mixture was diluted with chloroform, filtered and washed with chloroform. The filtrate was washed with water and brine. The organic layer was dried over anhydrous sodium sulfate, filtered and concentrated in vacuo. The residue was purified by silica gel column chromatography (chloroform : ethyl acetate = 80 : 20) to afford **20** (250 mg, 0.87 mmol, 90%) as a white solid.
^{1}H NMR (300 MHz, Chloroform-*d*) δ 9.02 (d, *J* = 2.6 Hz, 1H), 8.76 (s, 1H), 8.18 (dd, *J* = 8.5, 2.6 Hz, 1H), 8.08 (s, 1H), 7.74 (d, *J* = 8.3 Hz, 1H),
^{13}C NMR (75 MHz, Chloroform-*d*) δ 155.00, 150.11, 149.85, 144.86, 138.14, 132.27, 129.32.
ESI-MS [M+H]: Calculated: 288.93; Observed: 288.88

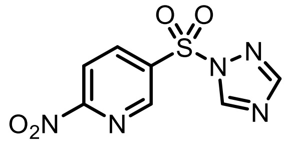

21

To a solution of 6-nitropyridine-3-sulfonyl chloride (100 mg, 0.45 mmol) in anhydrous acetonitrile (4.0 mL) was added 1,2,4-triazole (82 mg, 1.19 mmol) and stirred for 30 minutes at ambient temperature under argon. Then the reaction mixture was added triethylamine (66 μL, 0.47 mmol) and stirred for 15 hours. After the reaction, the mixture was diluted with dichloromethane and washed with water and brine. The organic layer was dried over anhydrous sodium sulfate, filtered and concentrated in vacuo. The residue was purified by silica gel column chromatography (dichloromethane : ethyl acetate = 83 : 17 to 75 : 25) to afford **21** (114 mg, 0.36 mmol, 79%) as a white solid.
^{1}H NMR (300 MHz, DMSO-*d*) δ 9.48 (s, 1H), 9.35 (s, 1H), 8.91 (d, *J* = 8.9 Hz, 1H), 8.54 (d, *J* = 8.5 Hz, 1H), 8.46 (s, 1H)
ESI-MS [M+H]: Calculated: 256.01; Observed: 256.07
^{13}C NMR could not be measured due to poor stability in any NMR solvent.

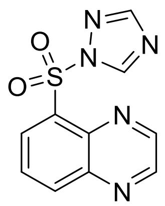

22

To a solution of quinoxaline-5-sulfonyl chloride (100 mg, 0.44 mmol) in anhydrous acetonitrile (4.4 mL) was added 1,2,4-triazole (91 mg, 1.31 mmol) and stirred for 30 minutes at ambient temperature under argon. Then the reaction mixture was added triethylamine (64 µL, 0.46 mmol) and stirred for 18 hours. After the reaction, the mixture was diluted with chloroform, filtered and washed with chloroform. The filtrate was washed with water and brine. The organic layer was dried over anhydrous sodium sulfate, filtered and concentrated in vacuo to afford **22** (116 mg, 0.44 mmol, quant.) as a white solid.

^{1}H NMR (300 MHz, DMSO-*d*) δ 9.62 (d, J = 3.4 Hz, 1H), 9.10 (s, 1H)), 8.99 (s, 1H, 8.79 (d, J = 7.4 Hz, 1H), 8.59 (d, J = 8.8 Hz, 1H), 8.22 (d, J = 3.3 Hz, 1H), 8.12-8.19 (m, 1H)

^{13}C NMR (75 MHz, DMSO-*d*) δ 154.16, 148.48, 147.56, 146.84, 142.07, 138.07, 137.71, 134.28, 131.33, 129.61

ESI-MS [M+H]: Calculated: 262.03; Observed: 262.13

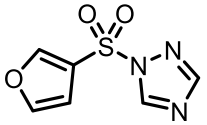

23

To a solution of furan-3-sulfonyl chloride (100 mg, 0.60 mmol) in anhydrous acetonitrile (6.0 mL) was added 1,2,4-triazole (124 mg, 1.80 mmol) and stirred for 30 minutes at ambient temperature under argon. Then the reaction mixture was added triethylamine (88 µL, 0.63 mmol) and stirred for 18 hours. After the reaction, the mixture was diluted with chloroform, filtered and washed with chloroform. The filtrate was washed with water and brine. The organic layer was dried over anhydrous sodium sulfate, filtered and concentrated in vacuo to afford **23** (119 mg, 0.59 mmol, 98%) as a white solid.

^{1}H NMR (400 MHz, DMSO-*d*) δ 9.36 (s, 1H), 8.93 (dd, J = 1.2, 0.9 Hz, 1H), 8.40 (s, 1H), 8.03 (dd, J = 2.0, 1.8 Hz, 1H), 7.01 (dd, J = 2.1, 1.9 Hz, 1H)

^{13}C NMR (75 MHz, DMSO-*d*) δ 154.53, 150.56, 147.07, 146.24, 123.06, 108.27.

ESI-MS [M+H]: Calculated: 200.01; Observed: 200.02

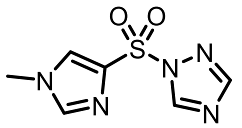

24

To a solution of 1-methyl-pyrazole-4-sulfonyl chloride (100 mg, 0.55 mmol) in anhydrous acetonitrile (5.5 mL) was added 1,2,4-triazole (115 mg, 1.69 mmol) and stirred for 30 minutes at ambient temperature under argon. Then the reaction mixture was added triethylamine (81 µL, 0.58 mmol) and stirred for 16 hours. After the reaction, the mixture was concentrated in vacuo. The residue was eluted with small amount of dichloromethane, filtrated and washed with dichloromethane. The filtrate was diluted with

diethyl ether, washed with water and brine. The organic layer was dried over anhydrous sodium sulfate, filtered and to afford the **24** (94 mg, 0.44 mmol, 79%) as a white solid.
^{1}H NMR (400 MHz, DMSO-*d6*) δ 9.33 (s, 1H), 8.44 (s, 1H), 8.31 (s, 1H), 7.92 (s, 1H), 3.74 (s, 3H)
^{13}C NMR (75 MHz, DMSO-*d6*) δ 162.24, 154.19, 146.51, 141.64, 133.32, 129.78.
ESI-MS [M+H]: Calculated: 214.03; Observed: 214.13

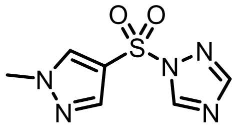

25

To a solution of 1-methyl-pyrazole-4-sulfonyl chloride (100 mg, 0.55 mmol) in anhydrous acetonitrile (5.5 mL) was added 1,2,4-triazole (115 mg, 1.69 mmol) and stirred for 30 minutes at ambient temperature under argon. Then the reaction mixture was added triethylamine (81 µL, 0.58 mmol) and stirred for 16 hours. After the reaction, the mixture was concentrated in vacuo. The residue was eluted with small amount of dichloromethane, filtrated and washed with dichloromethane. The filtrate was diluted with diethyl ether, washed with water and brine. The organic layer was dried over anhydrous sodium sulfate, filtered and to afford **25** (52 mg, 0.25 mmol, 44%) as a white solid.
^{1}H NMR (400 MHz, DMSO-*d6*) δ 9.28 (s, 1H), 8.79 (s, 1H), 8.35 (s, 1H), 8.14 (s, 1H), 3.91 (s, 3H)
^{13}C NMR (75 MHz, DMSO-*d6*) δ 154.19, 145.54, 139.62, 135.78, 116.09
ESI-MS [M+H]: Calculated: 214.03; Observed: 214.09

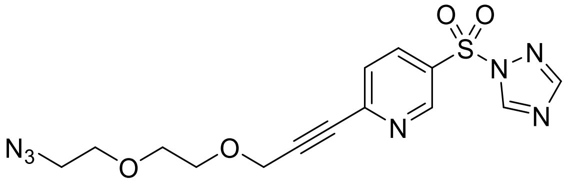

26 (AzP3S)

To a solution of **20** (50 mg, 0.17 mmol) in anhydrous tetrahydrofuran (1.4 mL) and triethylamine (0.29 mL) was added diethylene 3-(2-(2-azidoethoxy)ethoxy)prop-1-yne (31, 0.18mmol) and the mixture was degassed at ambient temperature under argon. Then to the reaction mixture was added copper(I) iodide (6.0 mg, 8.68 µmol) and Bis(triphenylphosphine)palladium(II) dichloride (1.6 mg, 8.68 µmol) and subsequently stirred for 2 hours. After the reaction, the mixture was diluted with dichloromethane and washed with water and brine. The organic layer was dried over anhydrous sodium sulfate, filtered and concentrated in vacuo. The residue was purified by silica gel column chromatography (chloroform : ethyl acetate = 75 : 25 to 50 :50) to afford **26** (18 mg, 0.05 mmol, 27%) as a yellow oil.
^{1}H NMR (500 MHz, Chloroform-*d*) δ 9.19 (s, 1H), 8.76 (s, 1H), 8.31-8.33 (m, 1H), 8.07 (s, 1H), 7.62 (d, *J* = 8.2 Hz, 1H), 4.50 (s, 2H), 3.78-3.79 (m, 2H), 3.68-3.72 (m, 4H), 3.39-3.41 (d, *J* = 4.5 Hz, 2H)
^{13}C NMR (75 MHz, Chloroform-*d*) δ 154.88, 149.41, 149.05, 144.85, 136.74, 131.38, 127.31, 91.63, 84.48, 70.58, 70.24, 69.88, 59.07, 50.76.
ESI-MS [M+H]: Calculated: 378.09; Observed: 378.01

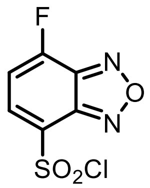

27-1

Synthesized according to the procedure previously reported in the literature.[5]

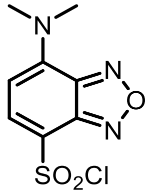

27-2

Synthesized (from **27-1**) according to the procedure previously reported in the literature.[6]

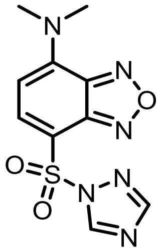

27

To a solution of **27-2** (100 mg, 0.38 mmol) in anhydrous acetonitrile (3.8 mL) was added 1,2,4-triazole (79 mg, 1.14 mmol) and stirred for 30 minutes at ambient temperature under argon. Then the reaction mixture was added triethylamine (56 μL, 0.40 mmol) and stirred for 2 hours. After the reaction, the mixture was diluted with dichloromethane, added water, and extracted with dichloromethane two times. The organic layer was dried over anhydrous sodium sulfate, filtered and concentrated in vacuo. The residue was purified by silica gel column chromatography (dichloromethane : ethyl acetate = 83 : 17) to afford **28** (53 mg, 0.18 mmol, 47%) as a yellow solid.

^{1}H NMR (400 MHz, Chloroform-*d*) δ 8.98 (s, 1H), 8.21 (d, *J* = 8.8 Hz, 1H), 7.95 (s, 1H), 6.13 (d, *J* = 8.7 Hz, 1H), 3.86 (s, 6H)

^{13}C NMR (100 MHz, DMSO-*d*) δ 153.97, 146.41, 146.35, 145.72, 144.55, 141.81, 102.05, 98.96.

ESI-MS [M+Na]: Calculated: 317.04; Observed: 317.20

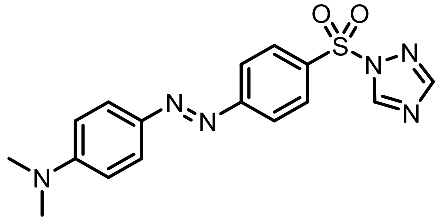

28

To a solution of dabsyl chloride (100 mg, 0.31 mmol) in anhydrous dichloromethane (3.0 mL) was added 1,2,4-triazole (64 mg, 0.93 mmol) and stirred for 30 minutes at ambient temperature under argon. Then the reaction mixture was added triethylamine 47 μL, 0.32 mmol) and stirred for 2 hours. After the reaction, the mixture was diluted with dichloromethane and washed with water and brine. The organic layer was dried over anhydrous sodium sulfate, filtered and concentrated in vacuo. The residue was purified by silica gel column chromatography (chloroform : acetone = 95 : 5) to afford **28** (85 mg, 0.24 mmol, 77%) as a red solid.
^{1}H NMR (400 MHz, Chloroform-*d*) δ 8.77 (s, 1H), 8.15 (d, *J* = 8.6 Hz, 2H), 8.04 (s, 1H), 7.97 (d, *J* = 8.6 Hz, 2H), 7.92 (d, *J* = 9.5 Hz, 2H), 6.76 (d, *J* = 9.2 Hz, 2H), 3.15 (s, 6H)
ESI-MS [M+H]: Calculated: 357.11; Observed: 357.21
^{13}C NMR could not be measured due to poor solubility in any NMR solvent.

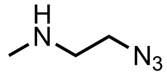

29-1
Synthesized according to the procedure previously reported in the literature.[7] CAUTION: small-molecule azides are a potential hazard.

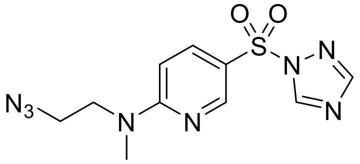

29
To a solution of **19** (739 mg, 3.02 mmol) and **29-1** (328 mg, 3.18 mmol) in anhydrous acetonitrile (30 mL) was added triethylamine (443 μL, 3.18mmol) and the reaction mixture was added and stirred for 18 hours. After the reaction, the mixture was diluted with dichloromethane and washed with water and brine. The organic layer was dried over anhydrous sodium sulfate, filtered and concentrated in vacuo. The residue was purified by silica gel column chromatography (chloroform : ethyl acetate = 75 : 25) to afford **29** (442 mg, 1.43 mmol, 47%) as a white solid.
^{1}H NMR (300 MHz, Chloroform-*d*) δ 8.77 (s, 1H), 8.72 (s, 1H), 8.02 (s, 1H), 7.98-8.02 (m, 2H), 7.26 (s, 1H), 6.56 (d, *J* = 9.2 Hz, 1H), 3.86 (t, J = 5.6 Hz, 1H), 3.57 (d, J = 5.9 Hz, 2H), 3.2 (s, 3H)
^{13}C NMR (75 MHz, Chloroform-*d*) δ 160.83, 154.11, 150.59, 144.12, 137.43, 118.40, 105.47, 50.02, 49.41, 38.20.
ESI-MS [M+H]: Calculated: 309.08; Observed: 309.16

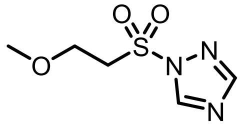

30
To a solution of 1,2,4-triazole (131 mg, 1.89 mmol) in anhydrous acetonitrile (3 mL) was slowly added 2-methoxy-1-ethanesulfonyl chloride (74 μL, 0.63 mmol) at ambient temperature under argon. The reaction mixture was stirred for 4 hours. After the reaction, the mixture was filtered off, washed with chloroform and concentrated in vacuo. The

residue was eluted with 50% chloroform in hexane, filtered off and washed with 50% chloroform in hexane. The organic layer was washed with water and brine, dried over anhydrous sodium sulfate, filtered and concentrated in vacuo to afford **30** (133mg, 0.63 mmol, quant.) as a pale yellow solid.
^{1}H NMR (300 MHz, Chloroform-*d*) δ 8.63 (s, 1H), 8.13 (s, H), 3.78 (s, 4H), 3.21 (s, 3H)
^{13}C NMR (100 MHz, Chloroform-*d*) δ 154.32, 145.66, 65.46, 59.06, 54.17.
ESI-MS [M+H]: Calculated: 192.04; Observed: 192.13

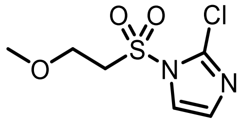

31

To a solution of 2-chloroimidazole (194 mg, 1.89 mmol) in anhydrous acetonitrile (3 mL) was slowly added 2-methoxy-1-ethanesulfonyl chloride (74 µL, 0.63 mmol) at ambient temperature under argon. The reaction mixture was stirred for 4 hours. After the reaction, the mixture was filtered off, washed with chloroform and concentrated in vacuo. The residue was eluted with 50% chloroform in hexane, filtered off and washed with 50% chloroform in hexane. The organic layer was washed with water and brine, dried over anhydrous sodium sulfate, filtered and concentrated in vacuo to afford **31** (120mg, 0.63 mmol, quant.) as a white solid.
^{1}H NMR (400 MHz, Chloroform-*d*) δ 7.35 (s, 1H), 6.97 (s, 1H), 3.75-3.78 (m, 5H), 3.24 (d, J = 2.2 Hz, 2H)
^{13}C NMR (100 MHz, Chloroform-*d*) δ 131.20, 128.37, 121.67, 65.37, 59.06, 55.17.
ESI-MS [M+H]: Calculated: 225.01; Observed: 225.08

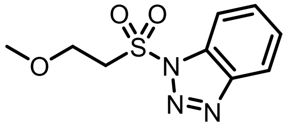

32

To a solution of 2-methoxy-1-ethanesulfonylchloride (37 µL, 0.31 mmol) in anhydrous dichloromethane (0.79 mL) was added benzotriazole (45 mg, 0.38 mmol) and N,N-diisopropylamine (132 µL, 0.76 mmol) at ambient temperature. The reaction mixture was stirred at ambient temperature for 14 hours under argon. After the reaction, the mixture was diluted with ethyl acetate and washed with water and brine. The organic layer was dried over anhydrous sodium sulfate, filtered and concentrated in vacuo. The residue was purified by silica gel column chromatography (chloroform : hexane = 67 : 33 to 99 : 1) to afford **32** (47 mg, 0.19 mmol, 60%) as a white solid.
^{1}H NMR (400 MHz, Chloroform-*d*) δ 8.14 (d, *J* = 8.4 Hz, 1H), 8.00 (d, *J* = 8.4 Hz, 1H), 7.66 (dd, *J* = 9.5, 7.8, Hz, 1H), 7.52 (d, *J* = 9.3, 7.8 Hz, 1H), 3.82 (t, *J* = 5.0 Hz, 2H), 3.73 (t, *J* = 5.0 Hz, 2H), 2.94 (s, 3H)
^{13}C NMR (100 MHz, DMSO-*d*) δ 144.73, 131.91, 130.53, 126.03, 120.17, 11.92, 64.90, 57.51, 55.04.
ESI-MS [M+H]: Calculated: 242.05; Observed: 242.45

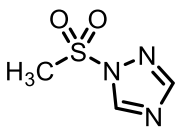

33

To a solution of 1,2,4-triazole (362 mg, 5.23 mmol) in anhydrous acetonitrile (17 mL) was slowly added methyl sulfonyl chloride (135 μL, 1.74 mmol) at ambient temperature under argon. The reaction mixture was stirred for 15 hours. After the reaction, the mixture was concentrated in vacuo, and eluted with chloroform. The white precipitate was filtered off and washed with chloroform. The filtrate was washed with water and brine, dried over anhydrous sodium sulfate, filtered and concentrated in vacuo to afford **33** (193 mg, 1.31 mmol, 75%) as a white solid.

^{1}H NMR (400 MHz, Chloroform-*d*) δ 8.70 (s, 1H), 8.16 (s, 1H), 3.45 (s, 3H)

^{13}C NMR (100 MHz, Chloroform-*d*) δ 154.48, 144.66, 41.92.

ESI-MS [M+H]: Calculated: 148.01; Observed: 148.83

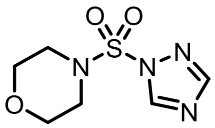

36

To a solution of 1,2,4-triazole (223 mg, 3.22 mmol) in anhydrous acetonitrile (10.7 mL) was slowly added 4-morpholinesulfonyl chloride (200 mg, 1.07 mmol) at ambient temperature under argon. The reaction mixture was refluxed for 2 hours. After the reaction, the mixture was filtered off, washed with chloroform and concentrated in vacuo. The residue was eluted with 50% chloroform in hexane, filtered off and washed with 50% chloroform in hexane. The organic layer was washed with water and brine, dried over anhydrous sodium sulfate and concentrated in vacuo, filtered and concentrated in vacuo. The residue was purified by silica gel column chromatography (chloroform : ethyl acetate = 83 : 17 to 75 : 25) to afford **36** (166 mg, 0.76 mmol, 70%) as a white solid.

^{1}H NMR (300 MHz, Chloroform-*d*) δ 8.59 (s, 1H), 8.10 (s, 1H), 3.77 (t, J = 4.7 Hz, 2H), 3.39 (t, J = 4.8 Hz, 2H), 3.30 (t, J = 4.8 Hz, 4H)

^{13}C NMR (100 MHz, DMSO-*d*) δ 153.84, 146.46, 64.92, 46.51.

ESI-MS [M+H]: Calculated: 219.05; Observed: 219.17

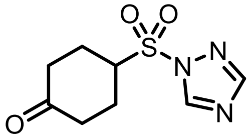

37

To a solution of 1,2,4-triazole (52 mg, 0.76 mmol) in anhydrous 50% acetonitrile in dichloromethane (6 mL) was slowly added 4-oxo-cyclohexanesulfonyl chloride (52 mg, 0.26 mmol) and triethylamine (39 μL, 0.28 mmol) at ambient temperature under argon. The reaction mixture was refluxed for 3 hours. After the reaction, the mixture was filtered off, washed with chloroform and concentrated in vacuo. The residue was eluted with 50% chloroform in hexane, filtered off and washed with 50% chloroform in hexane. The organic

layer was washed with water and brine, dried over anhydrous sodium sulfate, filtered and concentrated in vacuo to afford **37** (61 mg, 0.26 mmol, quant.) as a colorless oil.
^{1}H NMR (400 MHz, Chloroform-*d*) δ 8.68, (s, 1H), 8.15 (s, 1H), 3.94 (t, *J* = 10.64 Hz, 1H), 2.56 (d, *J* = 16.83 Hz, 2H), 2.33-2.43 (m, 4H), 2.07-2.17 (m, 2H)
^{13}C NMR (100 MHz, Chloroform-*d*) δ206.07, 154.69, 145.94, 60.52, 38.35, 25.22.
ESI-MS [M+H]: Calculated: 230.05; Observed: 230.13

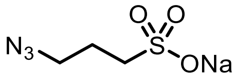

38-1
Synthesized according to the procedure previously reported in the literature.[8] CAUTION: small-molecule azides are a potential hazard.

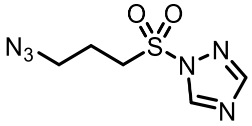

38
CAUTION: small-molecule azides are a potential hazard.
To a solution of **38-1** (200 mg, 1.07 mmol) in anhydrous toluene (11 mL) was added phosphorus pentachloride (315 mg, 1.12 mmol) and refluxed for 3 hours. After the reaction, the mixture filtered off, and washed with chloroform, and the filtrate concentrated in vacuo to afford the intermediate sulfonyl chloride as brown oil. To a solution of this crude intermediate in anhydrous toluene (10 mL) was added 1,2,4-triazole (206 mg, 1.12 mmol) and stirred for 1.5 hours. After the reaction, the mixture filtered off, washed with acetonitrile, and concentrated in vacuo. The residue was eluted with dichloromethane and filtered off again. The filtrate was washed with water two times, and dried over anhydrous sodium sulfate, filtered and concentrated in vacuo to afford **38** (187 mg, 0.87 mmol, 82% as 2 steps) as a yellow oil.
^{1}H NMR (400 MHz, Chloroform-*d*) δ 8.70 (s, 1H), 8.17 (s, 1H), 3.65 (t, *J* = 7.5 Hz, 2H), 3.51 (t, *J* = 5.4 Hz, 2H), 2.24-2.05 (m, 2H)
^{13}C NMR (100 MHz, Chloroform-*d*) δ154.64, 145.27, 51.70, 48.89, 23.02.
ESI-MS [M+H]: Calculated: 217.05; Observed: 217.91

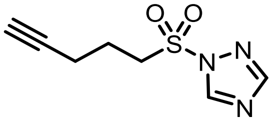

39
To a solution of 1,2,4-triazole (62 mg, 0.90 mmol) in anhydrous 50% acetonitrile in dichloromethane (6 mL) was slowly added pent-4-yne-1-sulfonyl chloride (50 mg, 0.30 mmol) at ambient temperature under argon. The reaction mixture was stirred for 17 hours. After the reaction, the mixture was filtered off, washed with chloroform and concentrated in vacuo. The residue was eluted with 50% chloroform in hexane, filtered off and washed with 50% chloroform in hexane. The organic layer was washed with water and brine, dried over anhydrous sodium sulfate, filtered and concentrated in vacuo to afford **39** (59 mg, 0.29 mmol, 98%) as a pale yellow oil.

^{1}H NMR (400 MHz, Chloroform-*d*) δ 8.68 (s, 1H), 8.14 (s, 1H), 6.68 (t, *J* = 7.8 Hz, 2H), 2.33-2.37 (m, 2H), 2.04 (t, *J* = 2.6 Hz, 1H), 1.95 (tt, *J* = 7.8, 6.6 Hz, 2H)
^{13}C NMR (100 MHz, Chloroform-*d*) δ 154.52, 145.28, 80.85, 71.16, 53.09, 21.96, 16.97.
ESI-MS [M+H]: Calculated: 200.04; Observed: 200.15

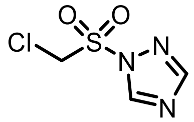

40

To a solution of 1,2,4-triazole (139 mg, 2.01 mmol) in anhydrous acetonitrile (6.7 mL) was slowly added chloromethanesulfonyl chloride (61 μL, 0.67 mmol) at ambient temperature under argon. The reaction mixture was stirred for 4 hours. After the reaction, the mixture was concentrated in vacuo, and eluted with chloroform. The white precipitate was filtered off, and washed with chloroform. The filtrate was washed with water and brine, dried over anhydrous sodium sulfate and concentrated in vacuo to afford **40** (101 mg, 0.68 mmol, quant.) as an orange solid.
^{1}H NMR (300 MHz, Chloroform-*d*) δ 8.73 (s, 1H), 8.21 (s, 1H), 4.96 (s, 2H)
^{13}C NMR (75 MHz, Chloroform-*d*) δ 155.05, 147.22, 56.25.
Unstable in water/acetonitrile systems, LCMS could not be recorded

^{1}H and ^{13}C NMR spectra

1

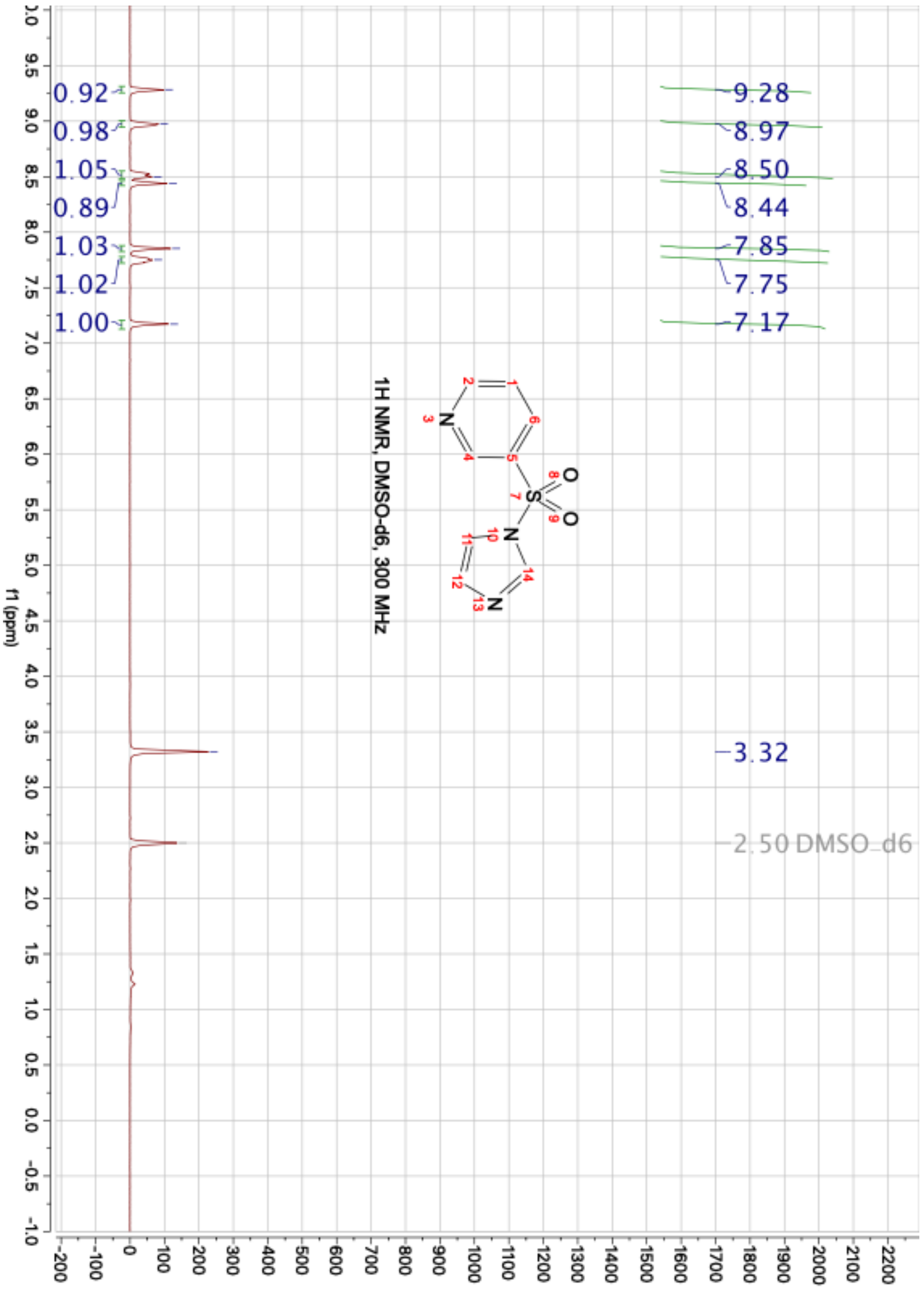

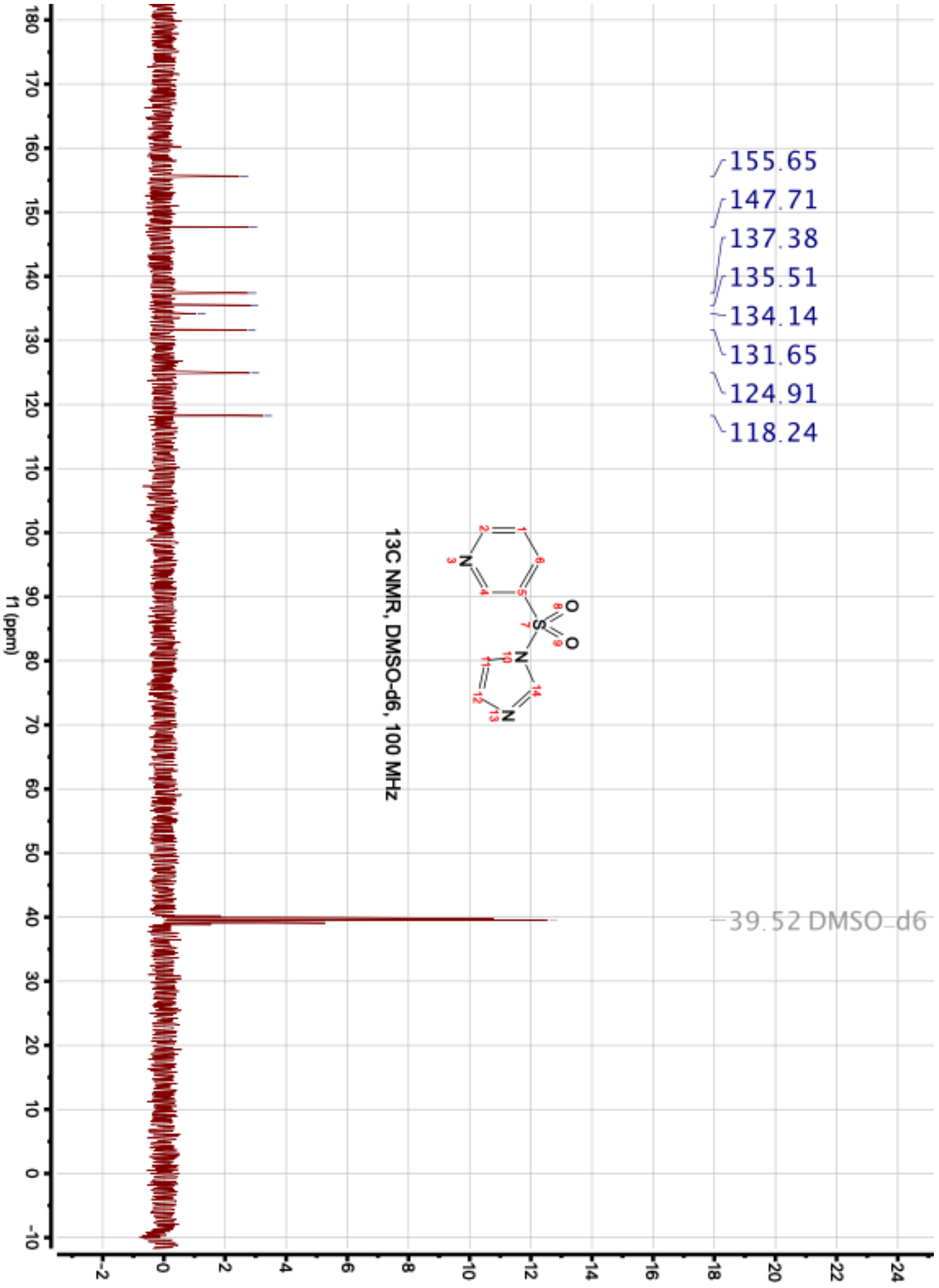
13C NMR, DMSO-d6, 100 MHz
155.65
147.71
137.38
135.51
134.14
131.65
124.91
118.24
39.52 DMSO-d6
f1 (ppm)

2

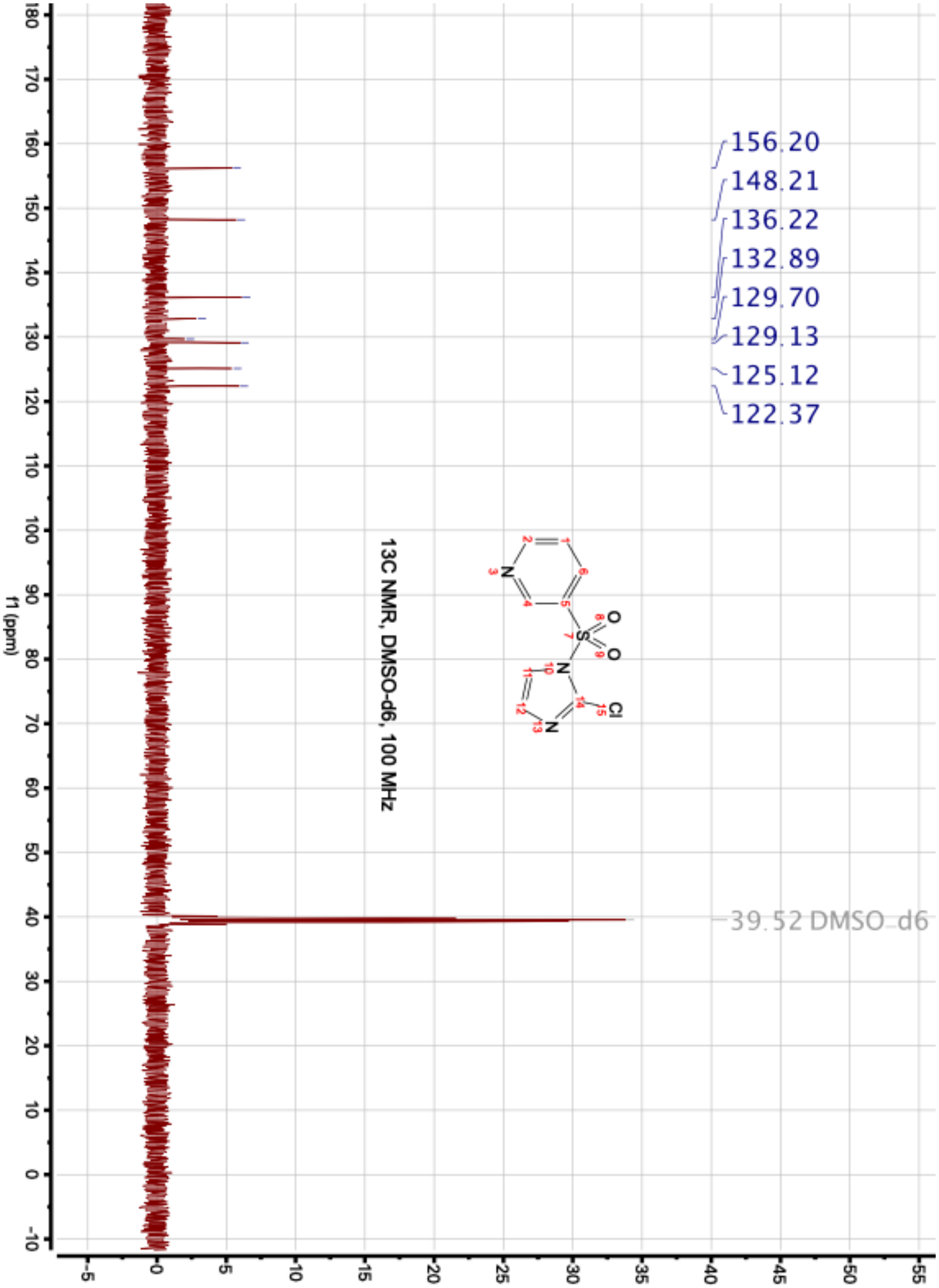
156.20
148.21
136.22
132.89
129.70
129.13
125.12
122.37
39.52 DMSO_d6
13C NMR, DMSO-d6, 100 MHz
f1 (ppm)

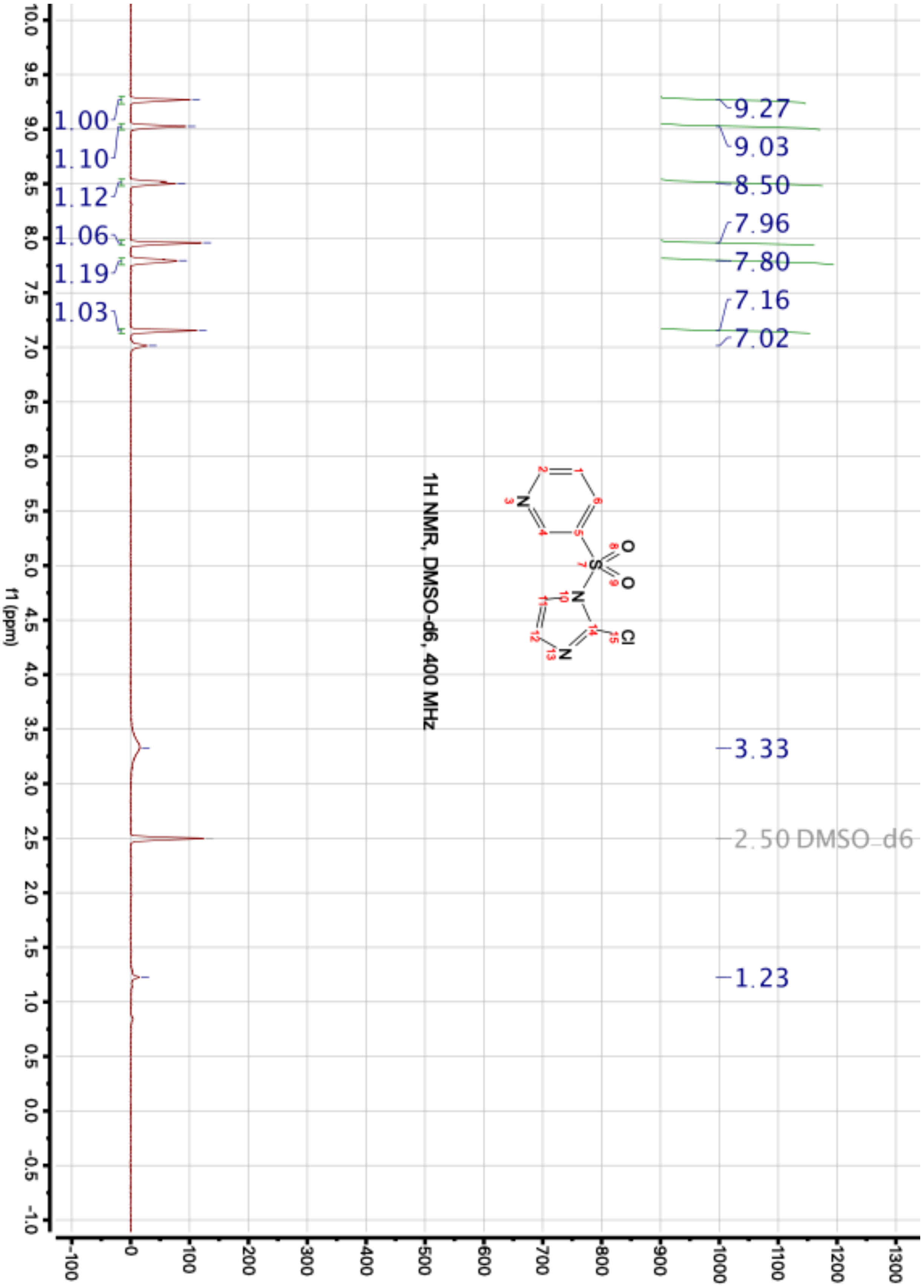
1H NMR, DMSO-d6, 400 MHz
9.27
9.03
8.50
7.96
7.80
7.16
7.02
3.33
2.50 DMSO-d6
1.23
1.00
1.10
1.12
1.06
1.19
1.03
f1 (ppm)

3

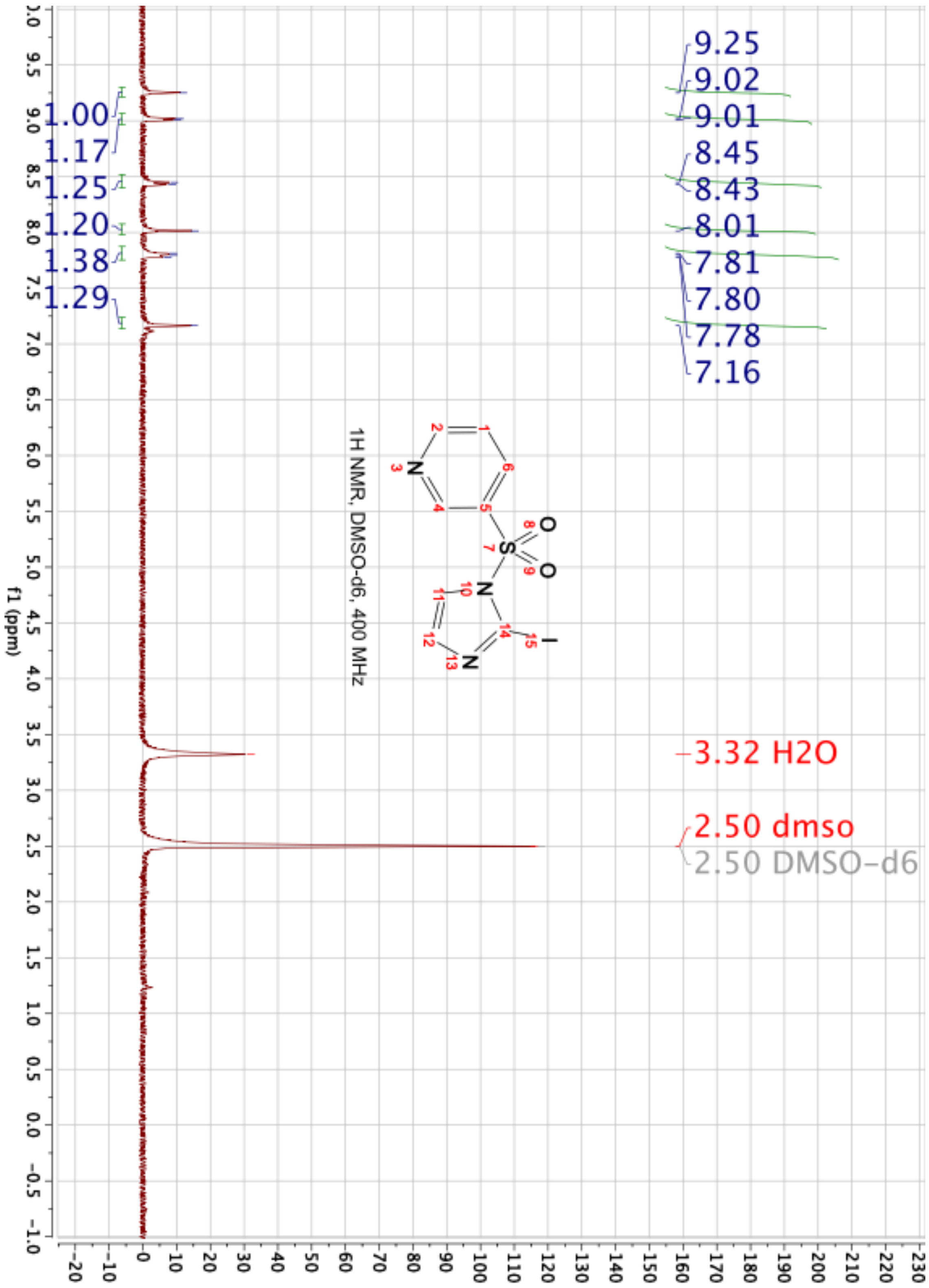
1H NMR, DMSO-d6, 400 MHz
9.25
9.02
9.01
8.45
8.43
8.01
7.81
7.80
7.78
7.16
3.32 H2O
2.50 dmso
2.50 DMSO-d6
1.00
1.17
1.25
1.20
1.38
1.29
f1 (ppm)

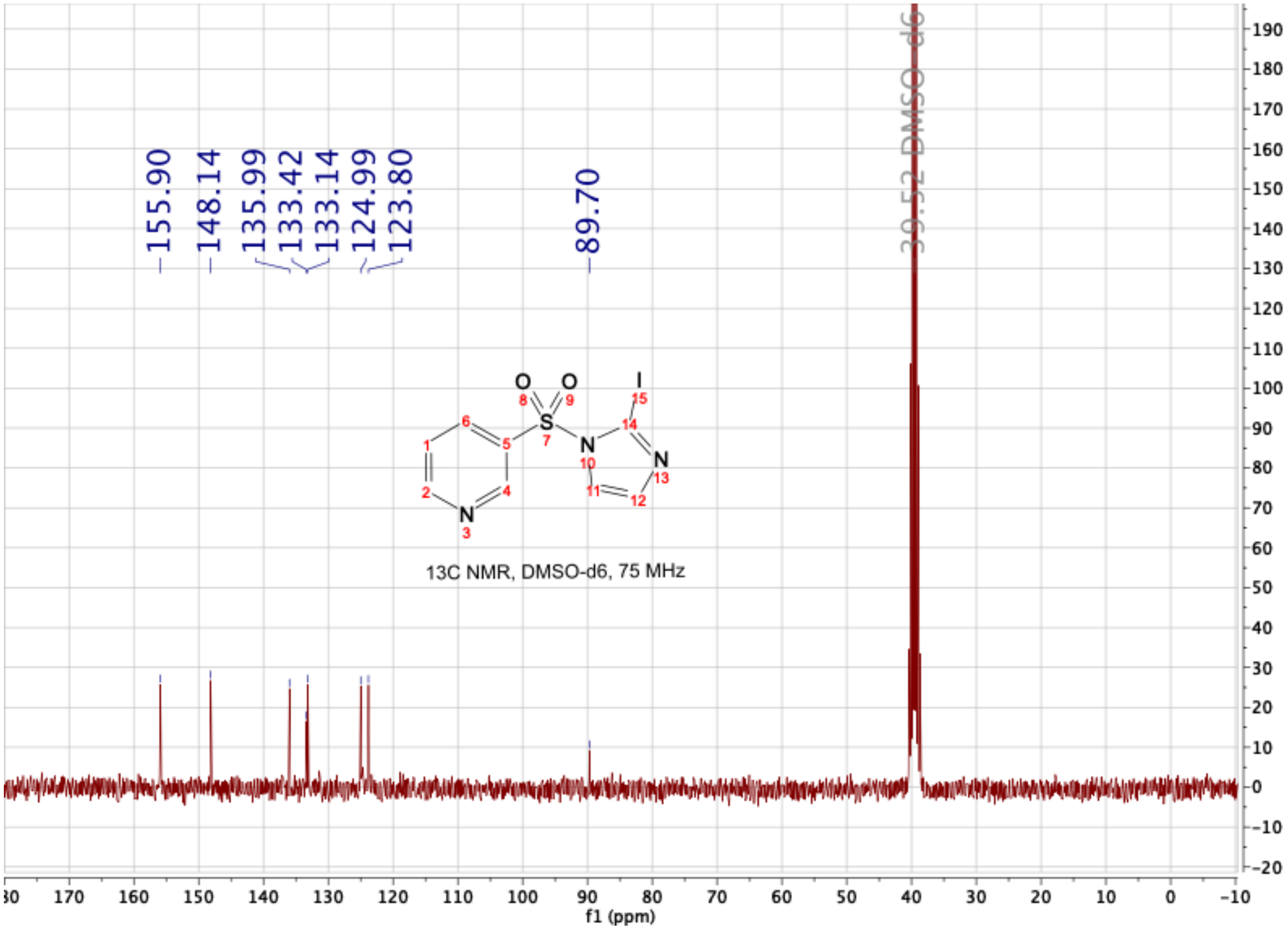
155.90
148.14
135.99
133.42
133.14
124.99
123.80
89.70
39.52 DMSO-d6
13C NMR, DMSO-d6, 75 MHz
f1 (ppm)

4

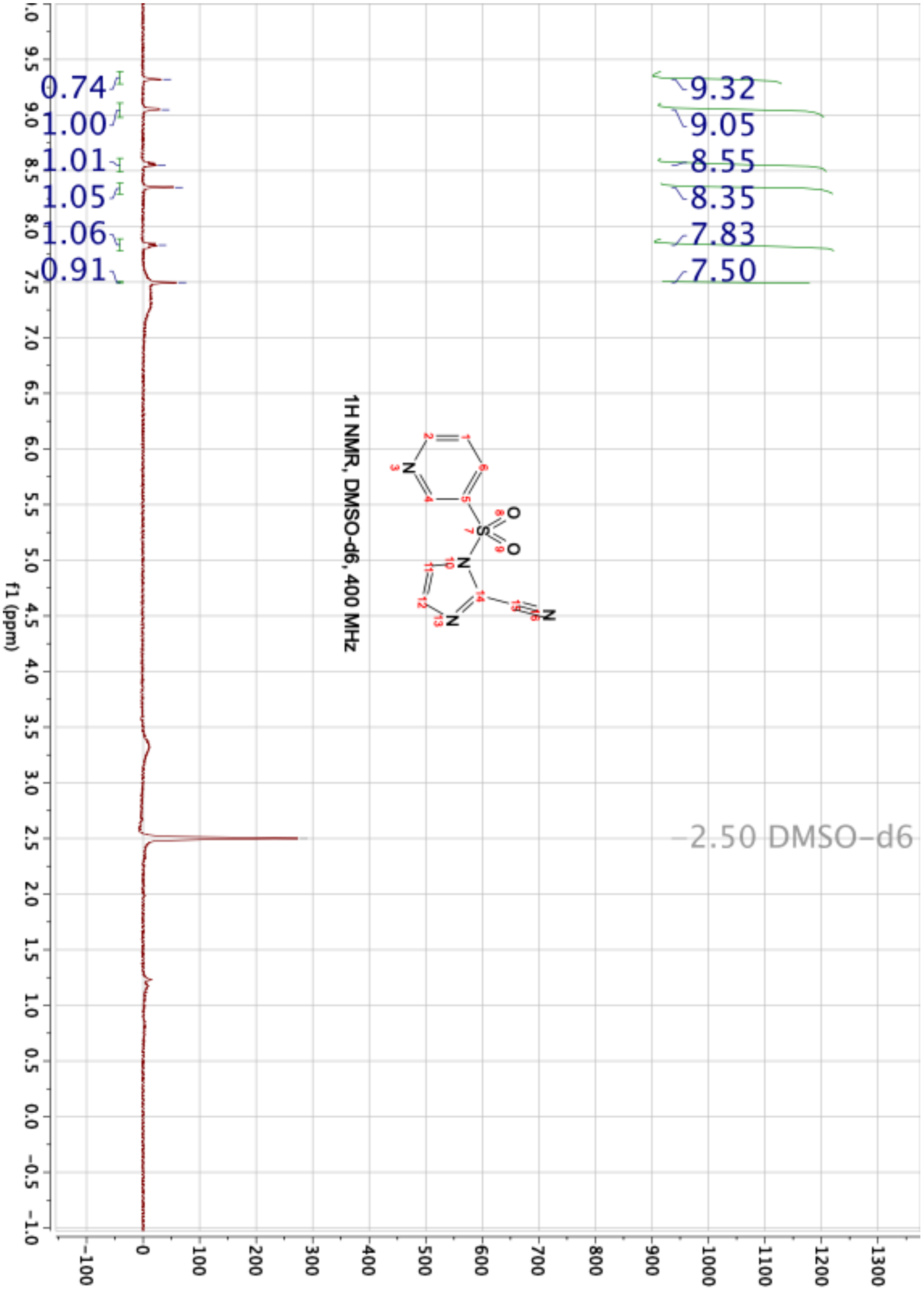
1H NMR, DMSO-d6, 400 MHz
9.32
9.05
8.55
8.35
7.83
7.50
2.50 DMSO-d6
0.74
1.00
1.01
1.05
1.06
0.91
f1 (ppm)

5

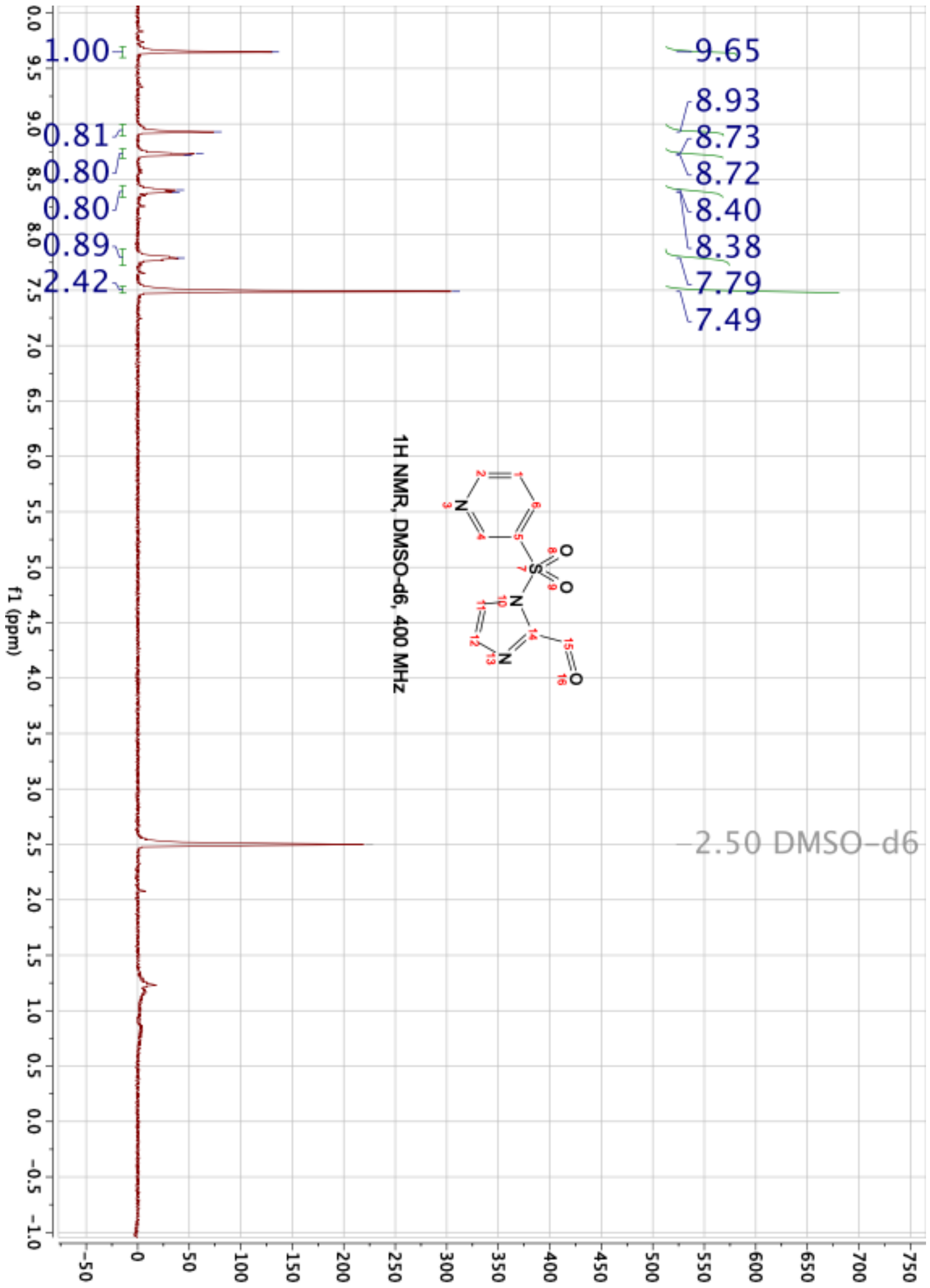
1H NMR, DMSO-d6, 400 MHz
9.65
8.93
8.73
8.72
8.40
8.38
7.79
7.49
2.50 DMSO-d6
1.00
0.81
0.80
0.80
0.89
2.42
f1 (ppm)

6

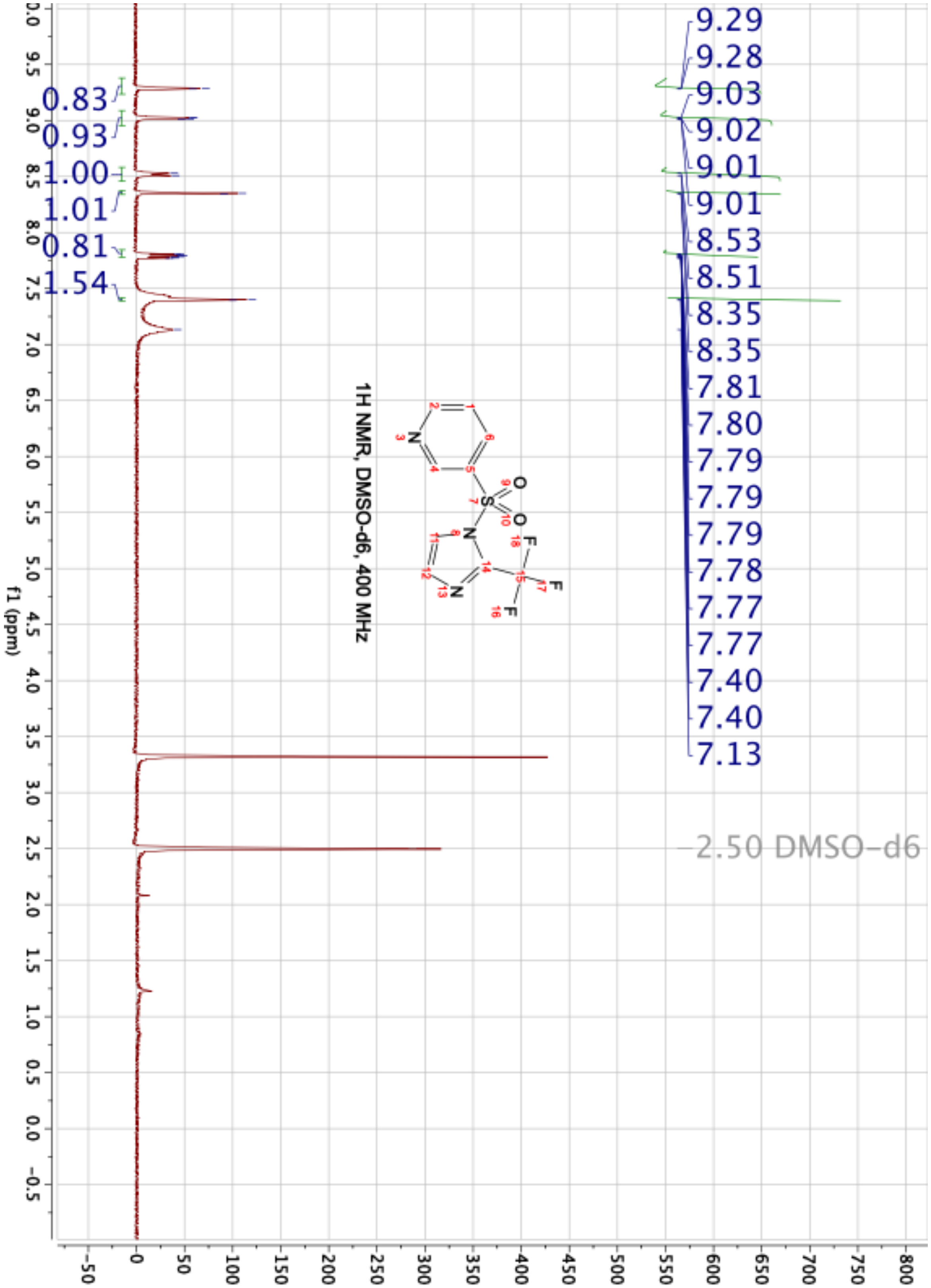
1H NMR, DMSO-d6, 400 MHz
9.29
9.28
9.03
9.02
9.01
9.01
8.53
8.51
8.35
8.35
7.81
7.80
7.79
7.79
7.79
7.78
7.77
7.77
7.40
7.40
7.13
2.50 DMSO-d6
0.83
0.93
1.00
1.01
0.81
1.54
f1 (ppm)

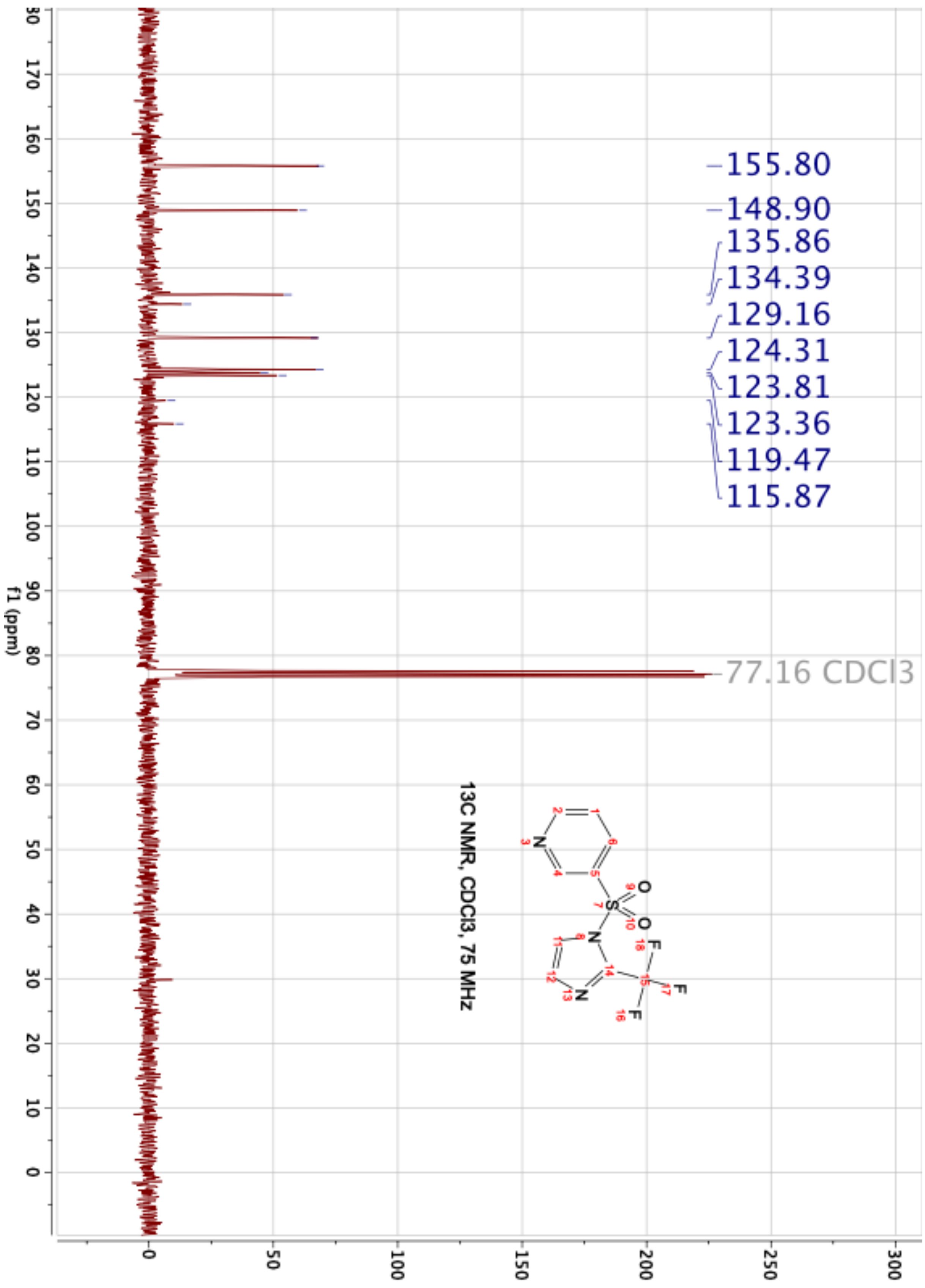

155.80
148.90
135.86
134.39
129.16
124.31
123.81
123.36
119.47
115.87
77.16 CDCl3
13C NMR, CDCl3, 75 MHz
f1 (ppm)

7

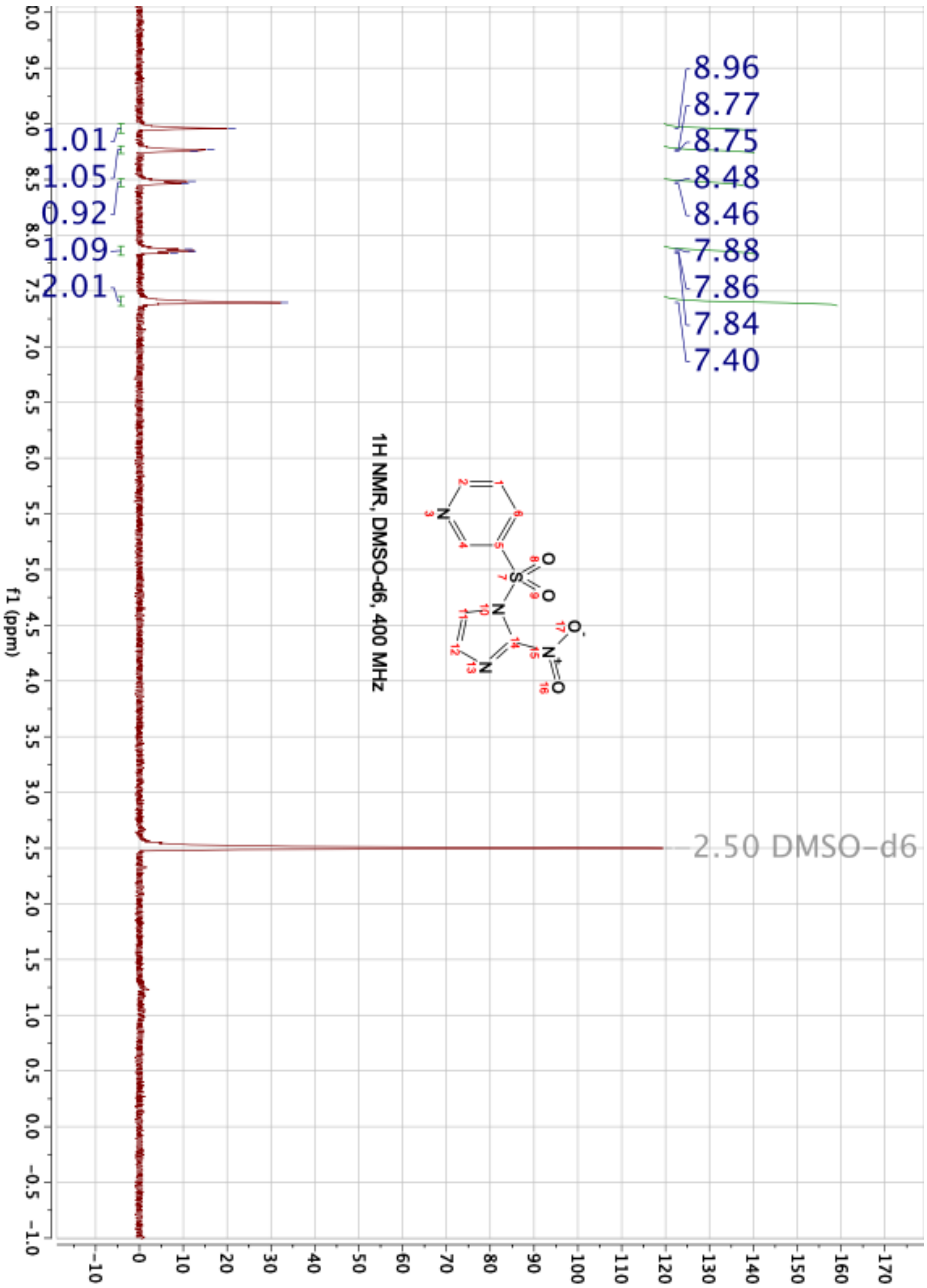
1H NMR, DMSO-d6, 400 MHz
8.96
8.77
8.75
8.48
8.46
7.88
7.86
7.84
7.40
2.50 DMSO-d6
1.01
1.05
0.92
1.09
2.01
f1 (ppm)

10

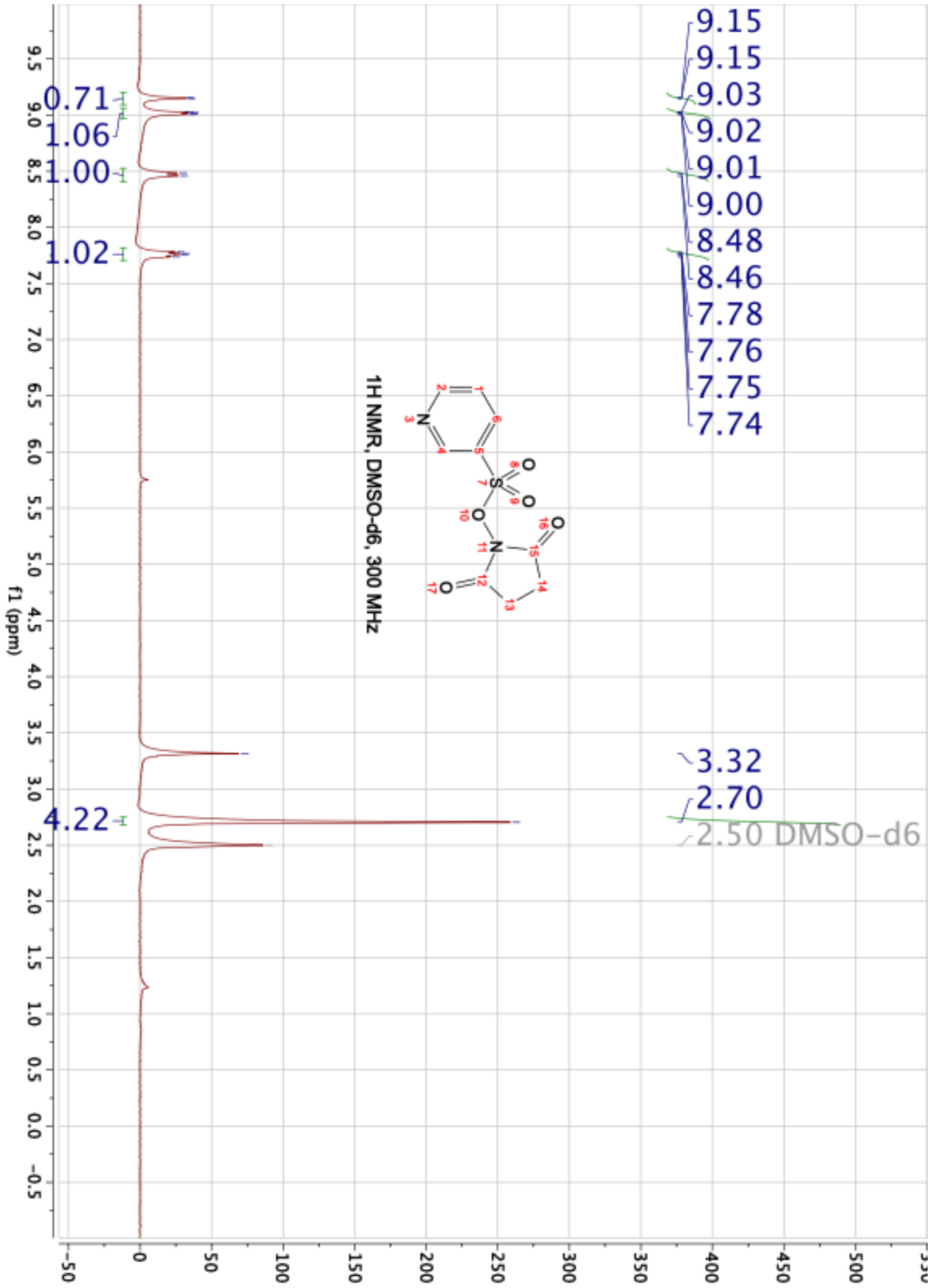
1H NMR, DMSO-d6, 300 MHz
9.15
9.15
9.03
9.02
9.01
9.00
8.48
8.46
7.78
7.76
7.75
7.74
3.32
2.70
2.50 DMSO-d6
0.71
1.06
1.00
1.02
4.22
f1 (ppm)

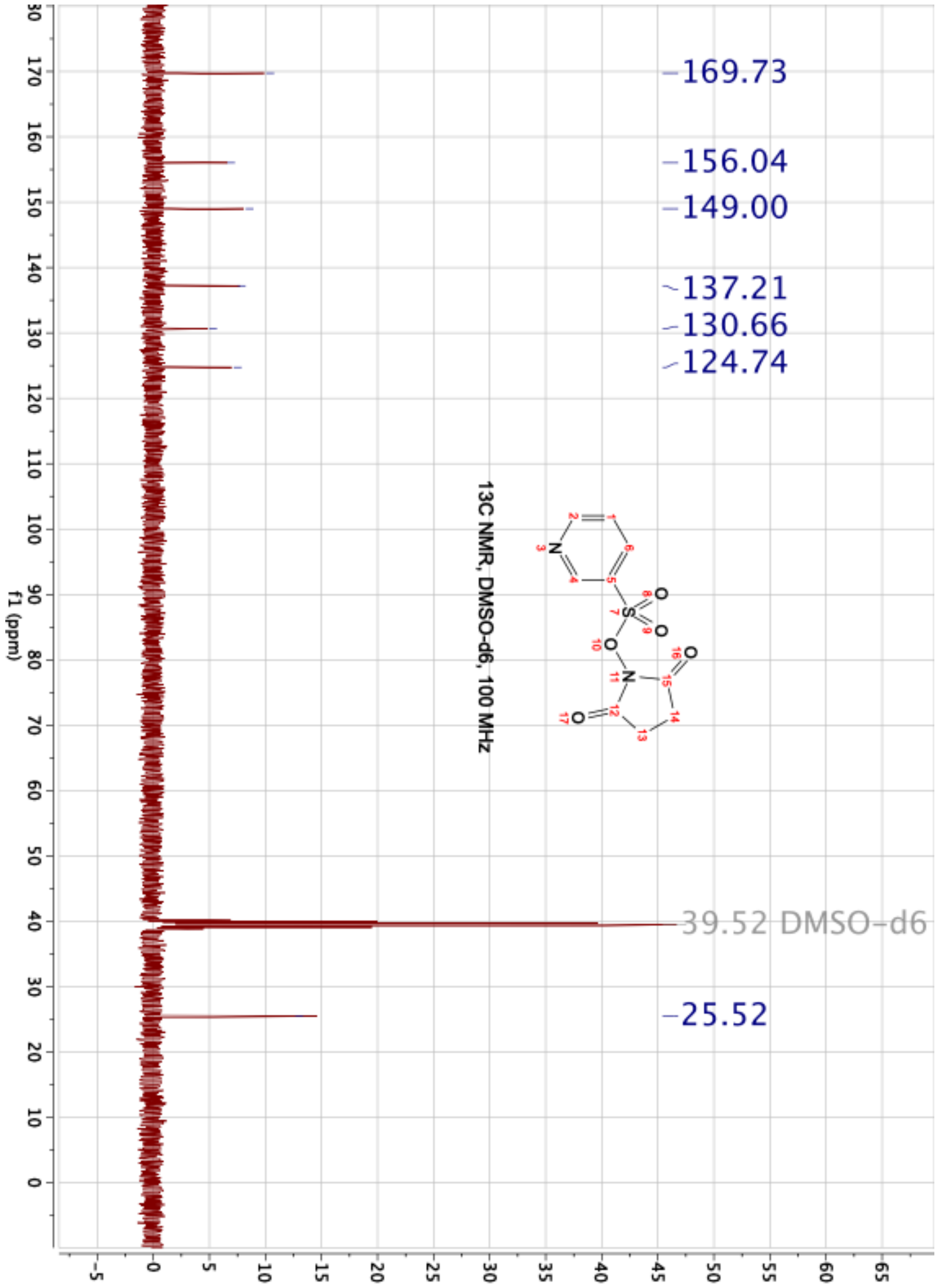
13C NMR, DMSO-d6, 100 MHz
169.73
156.04
149.00
137.21
130.66
124.74
39.52 DMSO-d6
25.52
f1 (ppm)

11

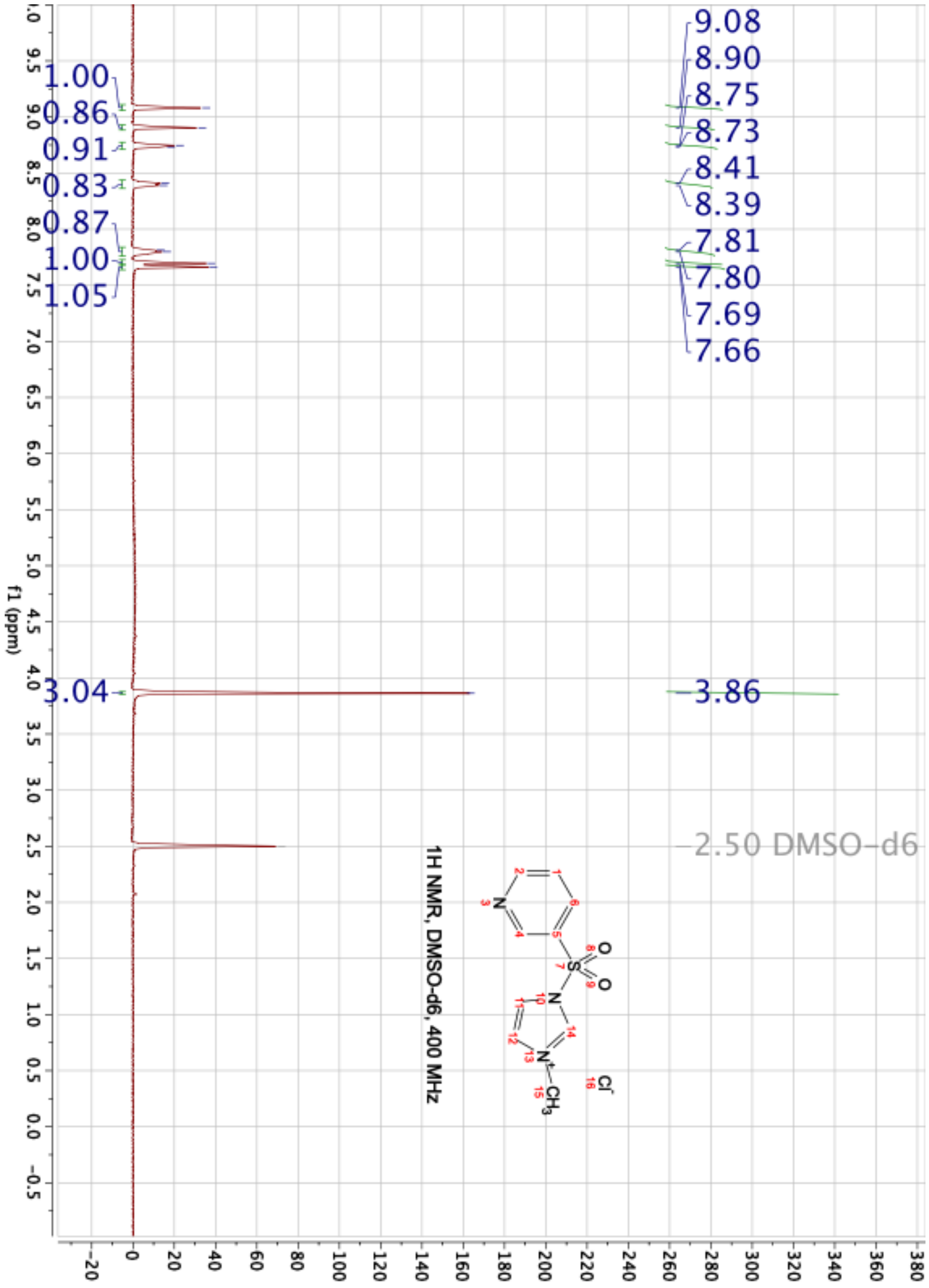

9.08
8.90
8.75
8.73
8.41
8.39
7.81
7.80
7.69
7.66
3.86
2.50 DMSO-d6
1.00
0.86
0.91
0.83
0.87
1.00
1.05
3.04
1H NMR, DMSO-d6, 400 MHz
f1 (ppm)

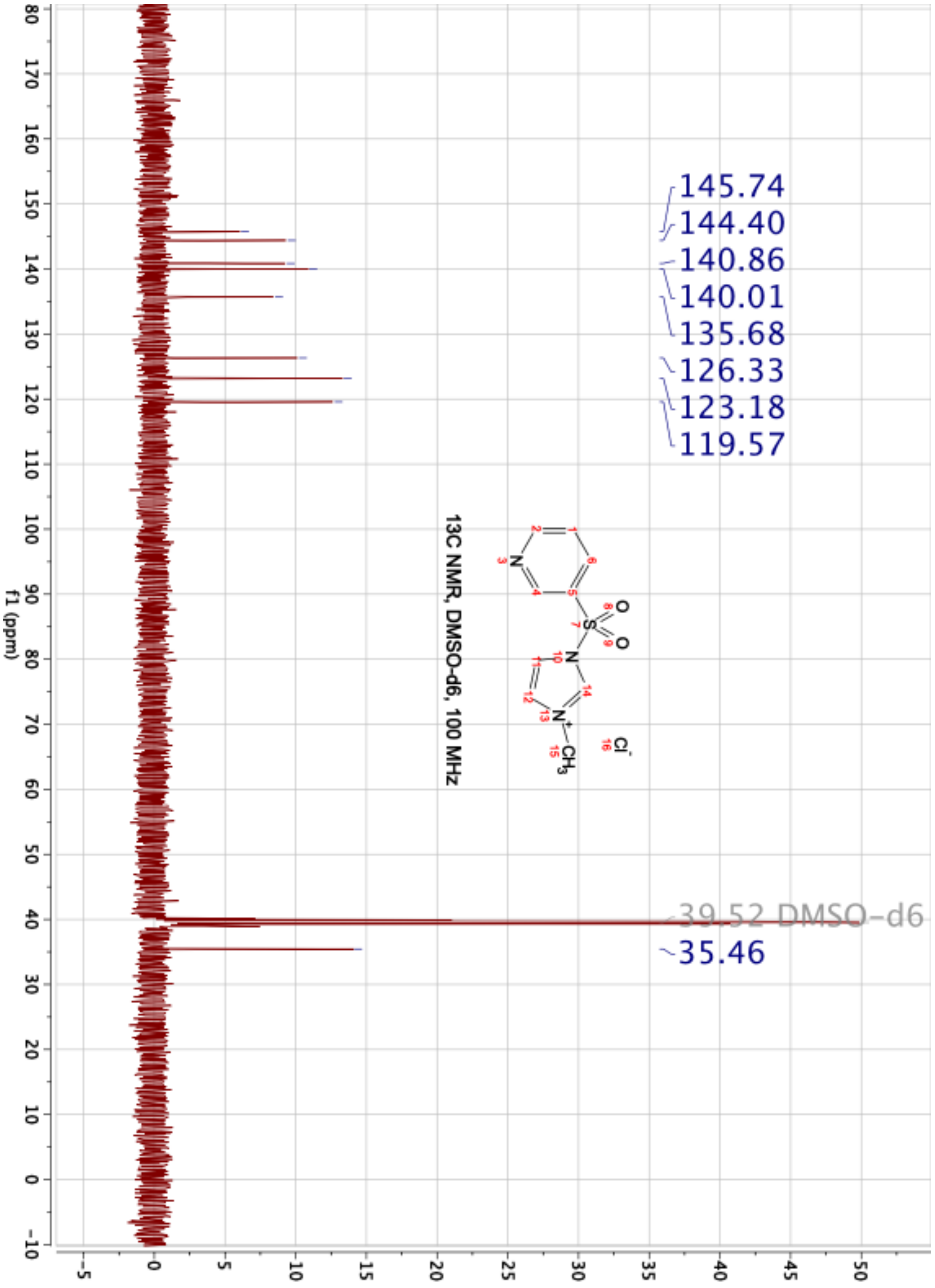

13C NMR, DMSO-d6, 100 MHz
145.74
144.40
140.86
140.01
135.68
126.33
123.18
119.57
39.52 DMSO-d6
35.46
f1 (ppm)

12

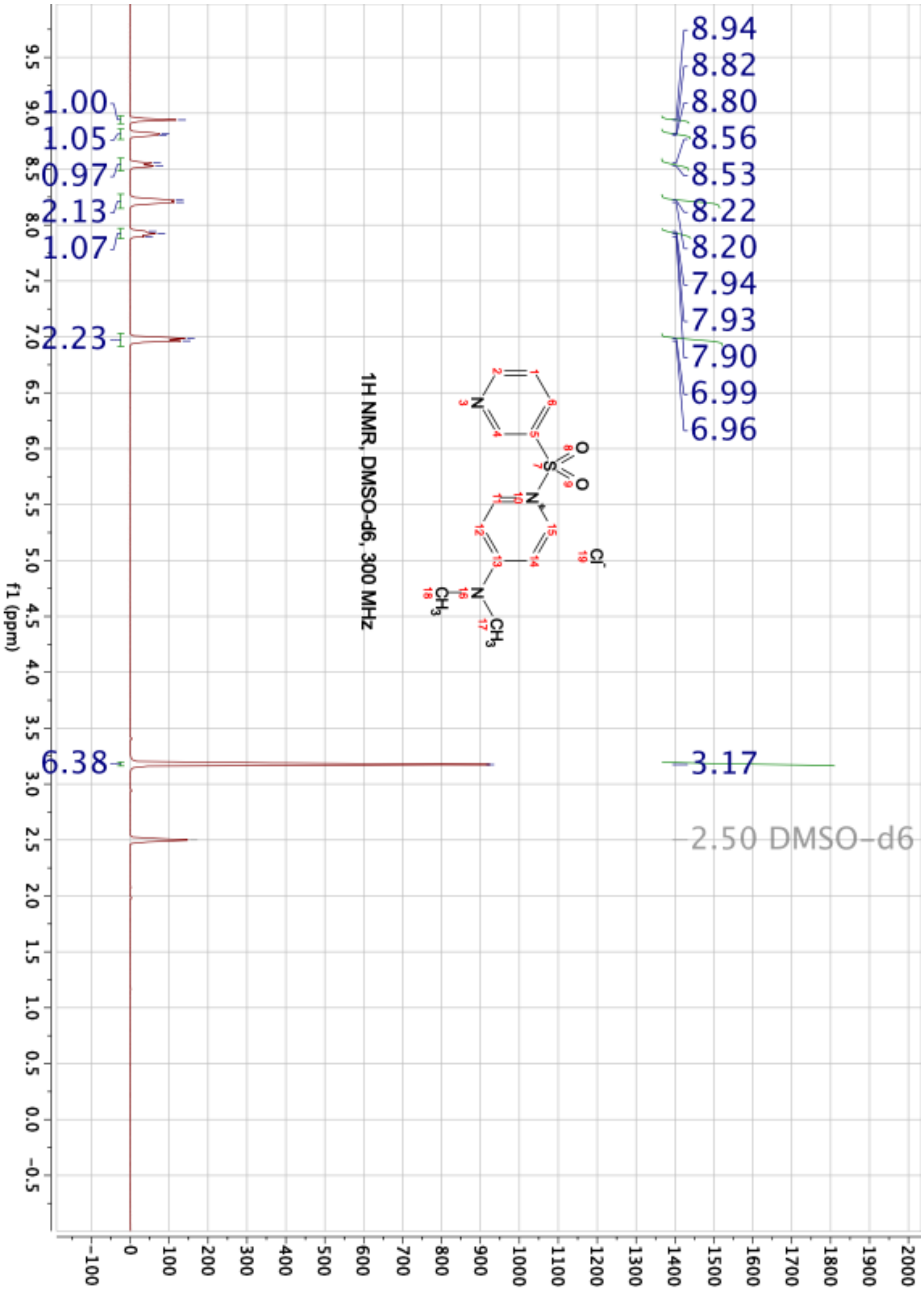
1H NMR, DMSO-d6, 300 MHz
8.94
8.82
8.80
8.56
8.53
8.22
8.20
7.94
7.93
7.90
6.99
6.96
3.17
2.50 DMSO-d6
1.00
1.05
0.97
2.13
1.07
2.23
6.38
f1 (ppm)

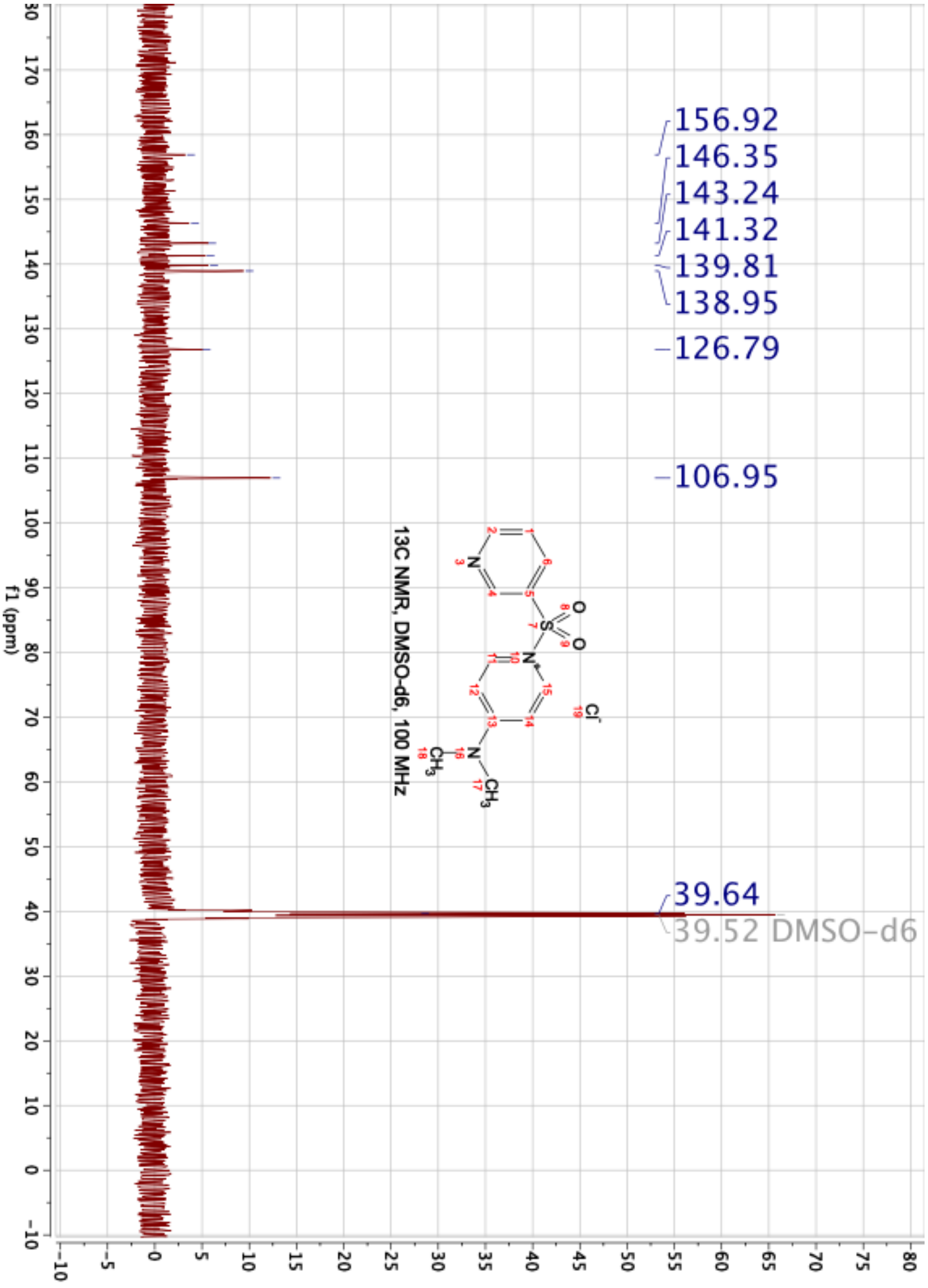
156.92
146.35
143.24
141.32
139.81
138.95
126.79
106.95
39.64
39.52 DMSO-d6
13C NMR, DMSO-d6, 100 MHz
f1 (ppm)

14

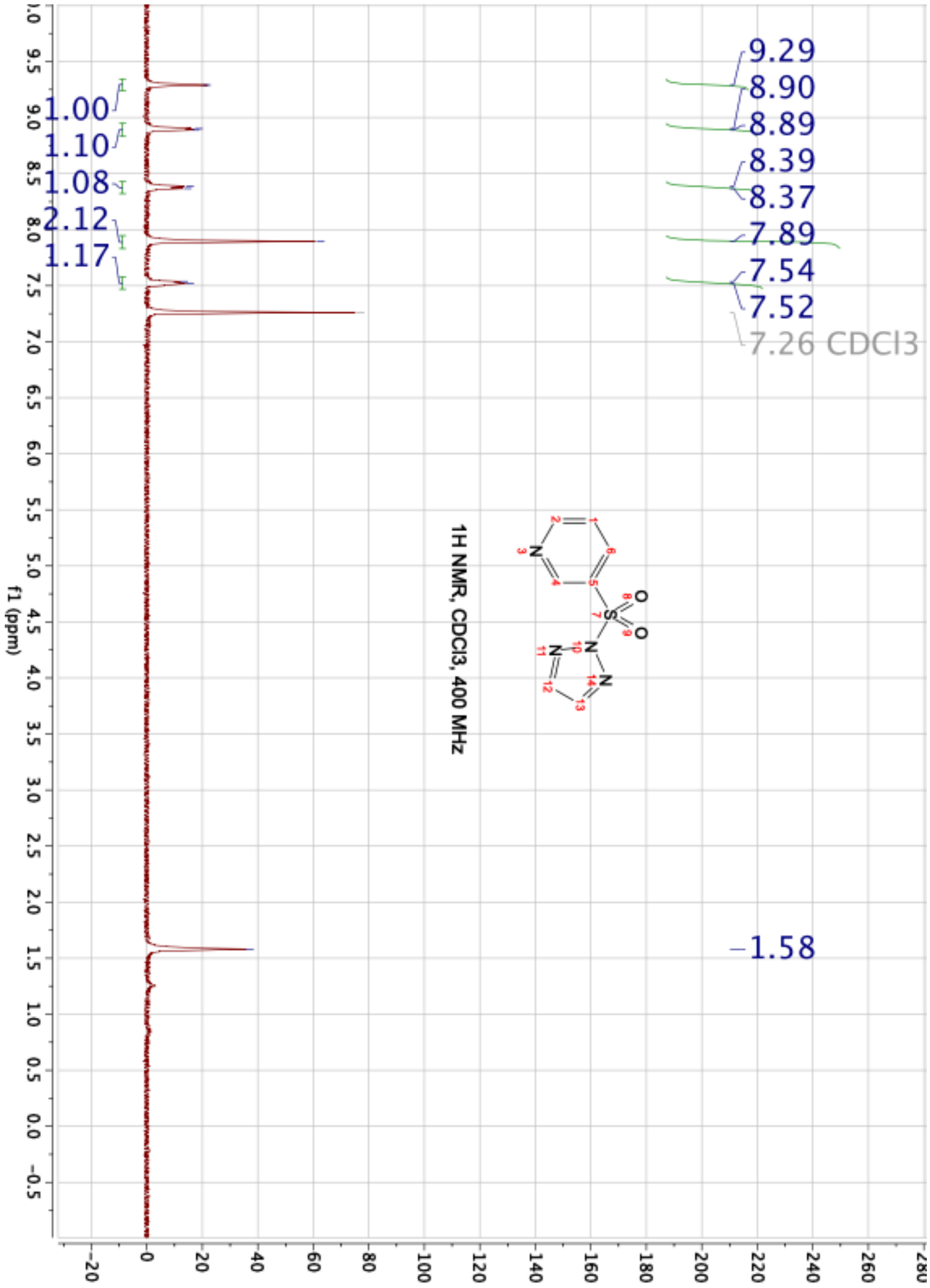
1H NMR, CDCl3, 400 MHz
9.29
8.90
8.89
8.39
8.37
7.89
7.54
7.52
7.26 CDCl3
1.58
1.00
1.10
1.08
2.12
1.17
f1 (ppm)

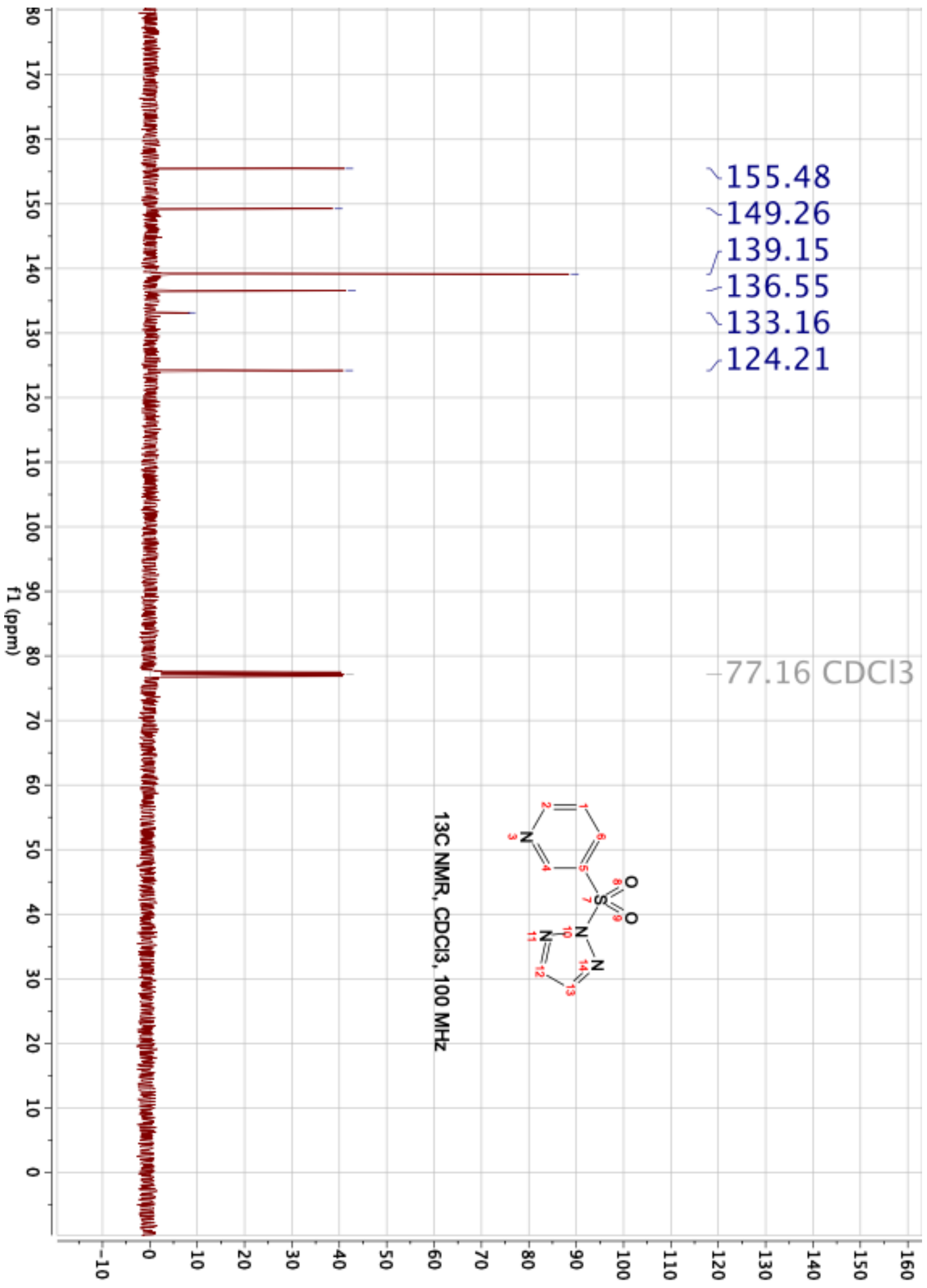
155.48
149.26
139.15
136.55
133.16
124.21
77.16 CDCl3
13C NMR, CDCl3, 100 MHz
f1 (ppm)

15

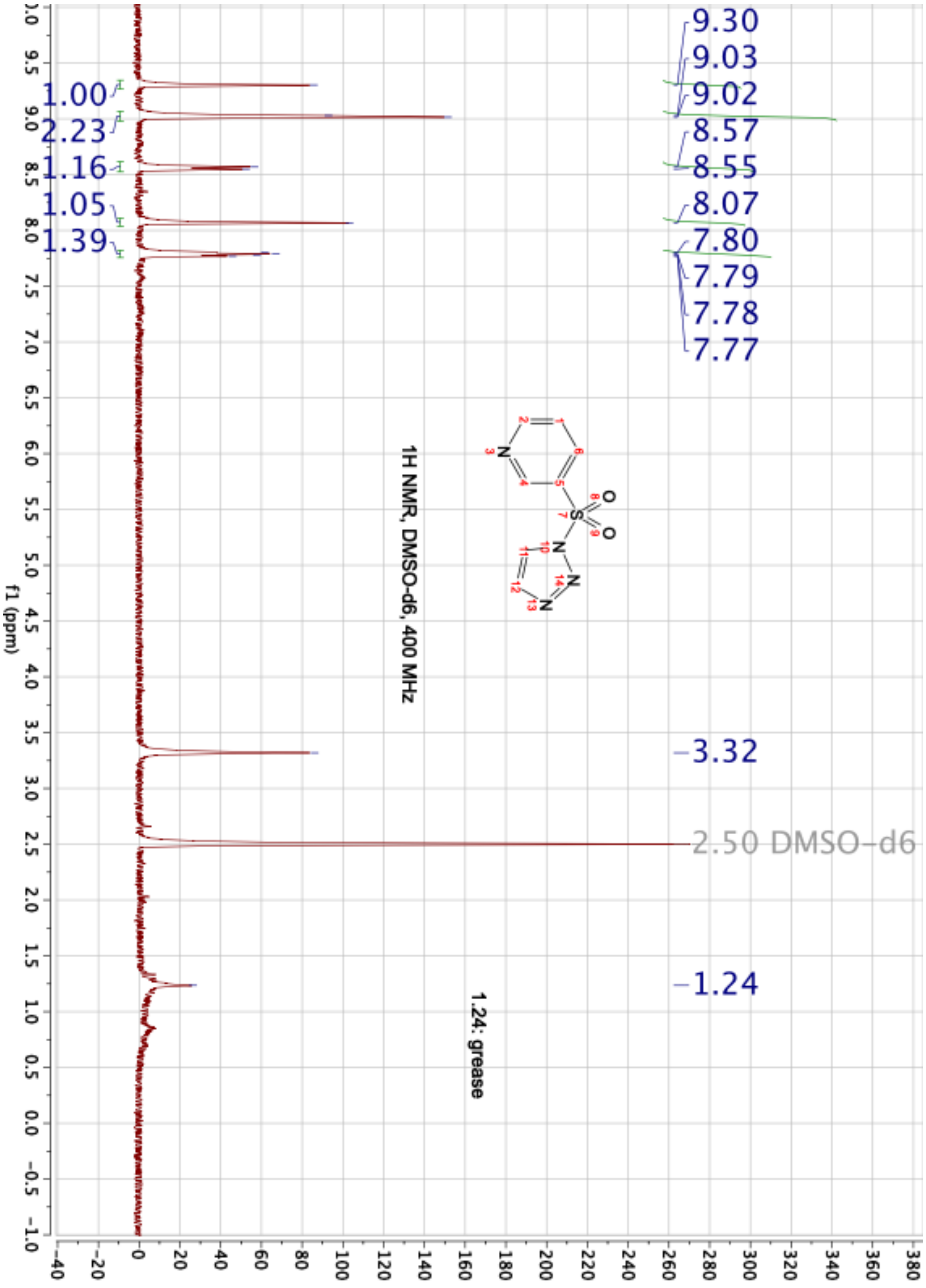

1H NMR, DMSO-d6, 400 MHz
1.24: grease
9.30
9.03
9.02
8.57
8.55
8.07
7.80
7.79
7.78
7.77
3.32
2.50 DMSO-d6
1.24
1.00
2.23
1.16
1.05
1.39
f1 (ppm)

16 (P3S)

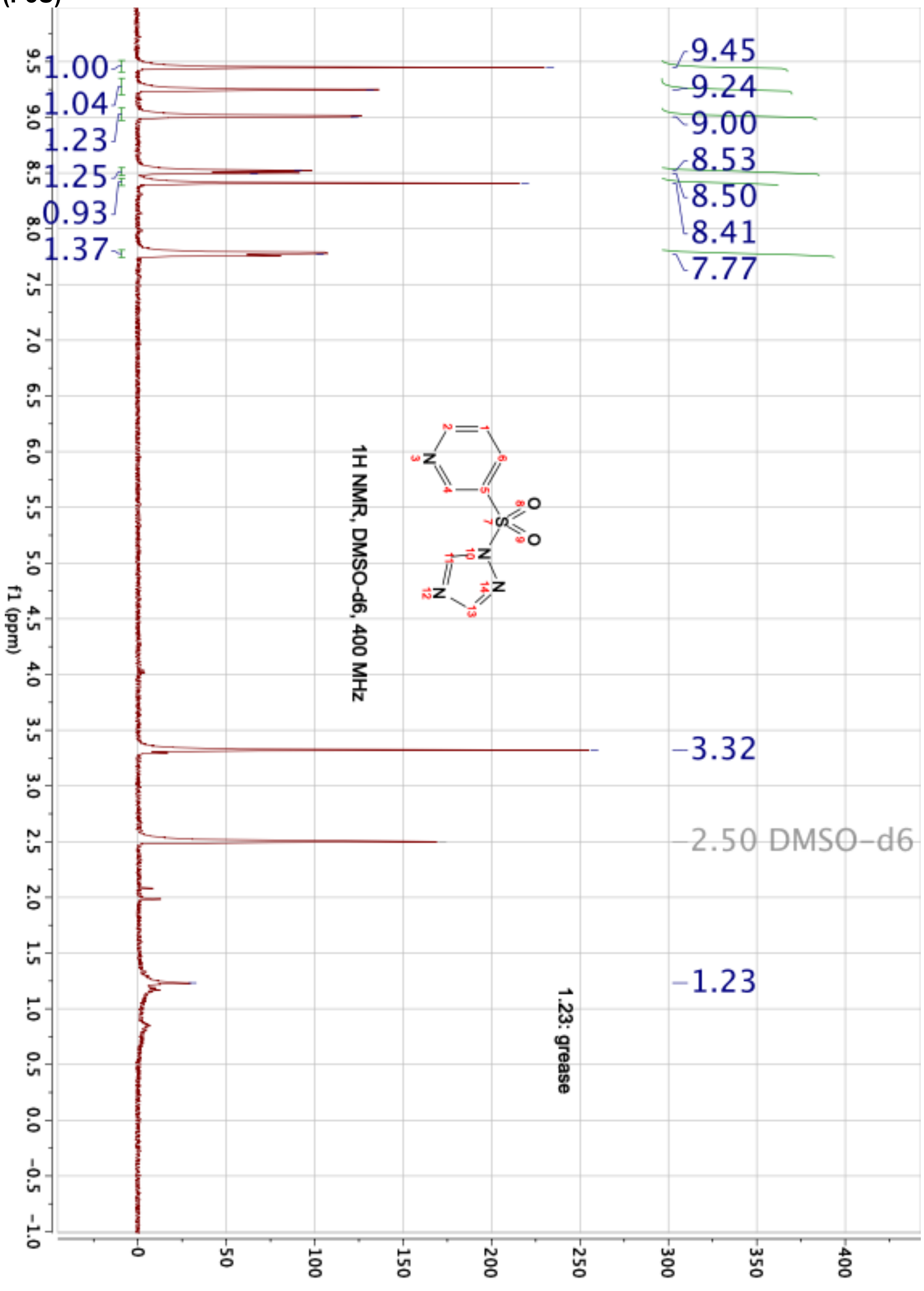
1H NMR, DMSO-d6, 400 MHz
1.23: grease
9.45
9.24
9.00
8.53
8.50
8.41
7.77
3.32
2.50 DMSO-d6
1.23
1.00
1.04
1.23
1.25
0.93
1.37
f1 (ppm)

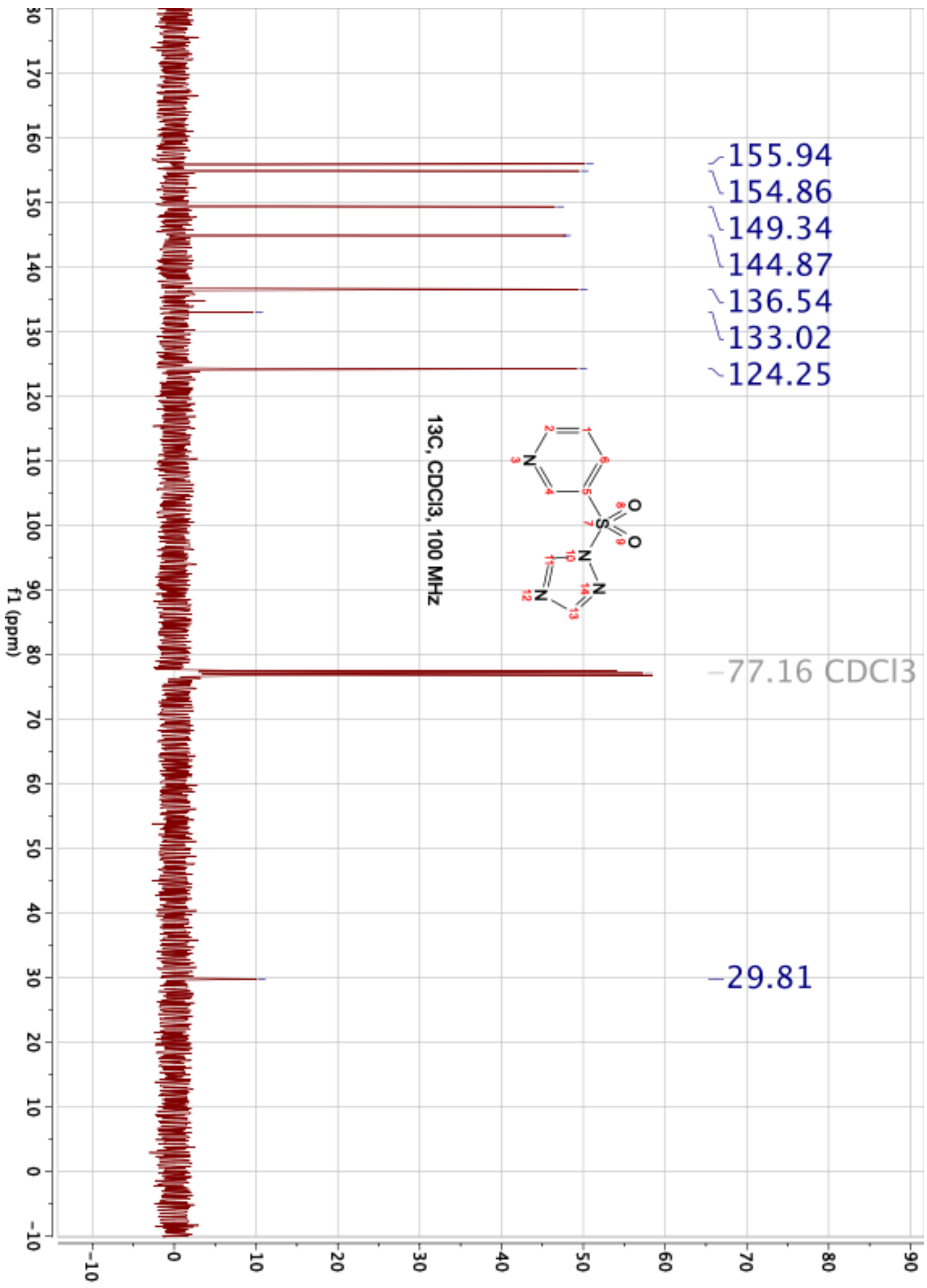
155.94
154.86
149.34
144.87
136.54
133.02
124.25
77.16 CDCl3
29.81
13C, CDCl3, 100 MHz
f1 (ppm)

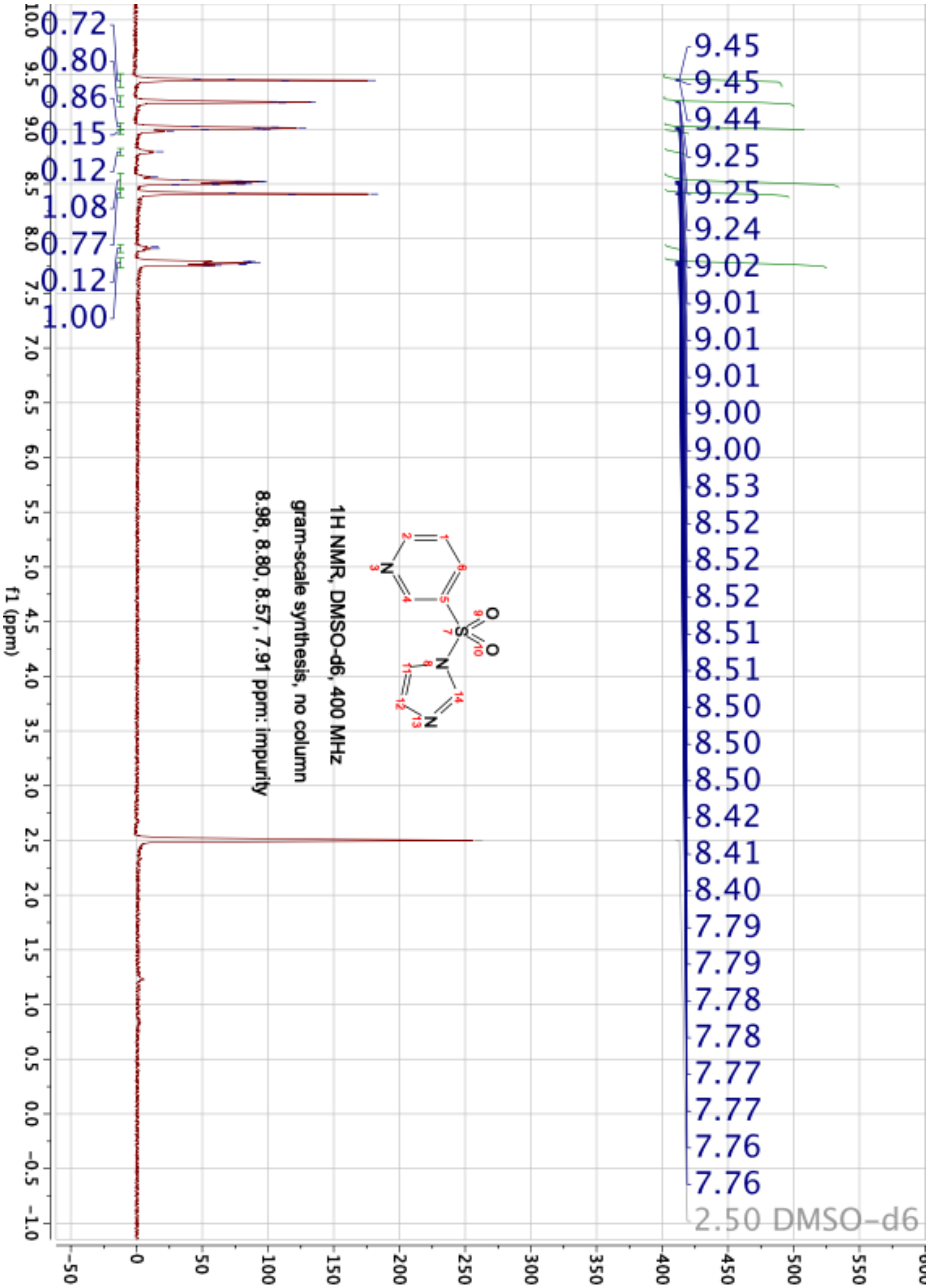
1H NMR, DMSO-d6, 400 MHz
gram-scale synthesis, no column
8.98, 8.80, 8.57, 7.91 ppm: impurity
f1 (ppm)
2.50 DMSO-d6

17

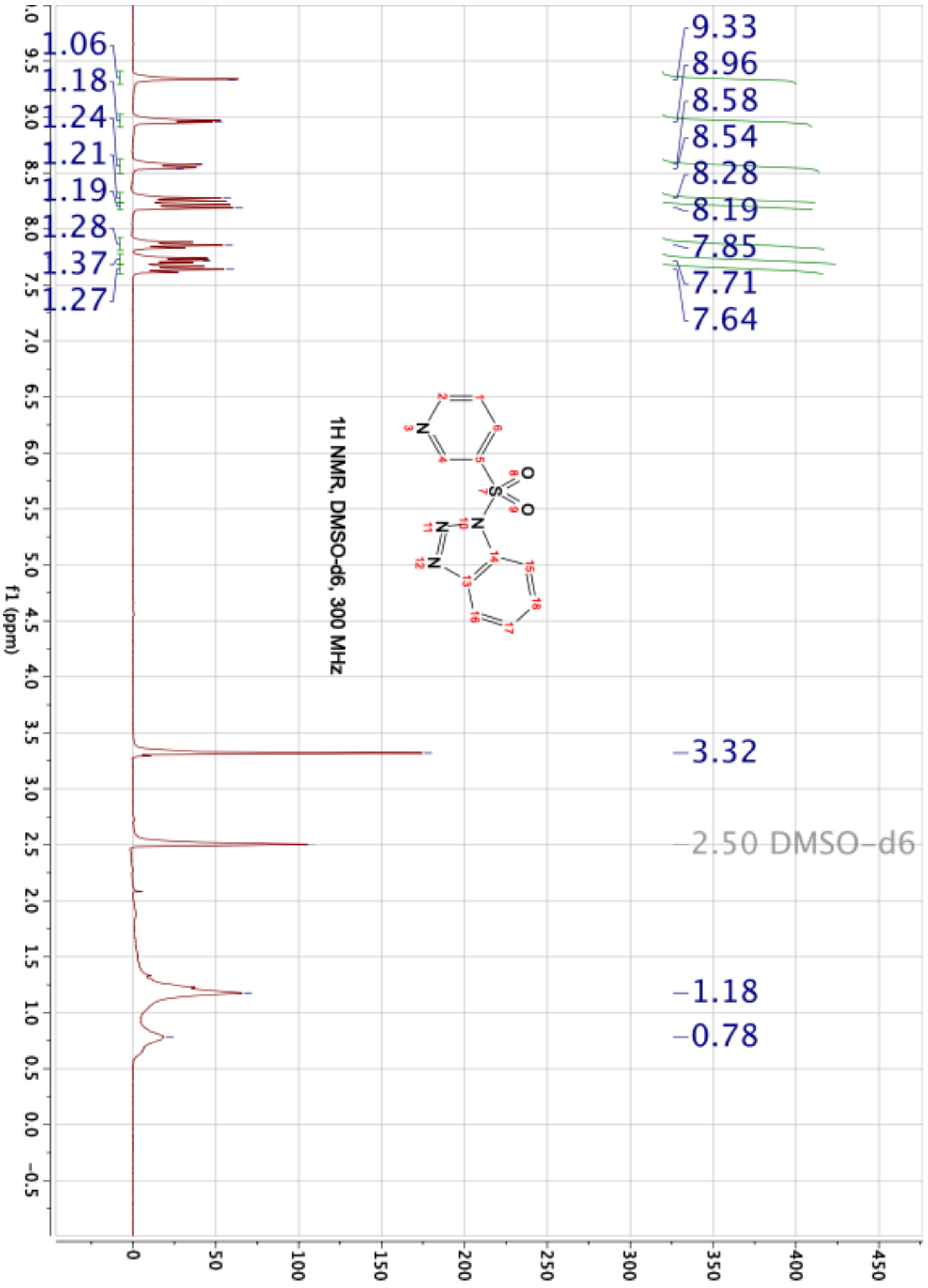
1H NMR, DMSO-d6, 300 MHz
9.33
8.96
8.58
8.54
8.28
8.19
7.85
7.71
7.64
3.32
2.50 DMSO-d6
1.18
0.78
1.06
1.18
1.24
1.21
1.19
1.28
1.37
1.27
f1 (ppm)

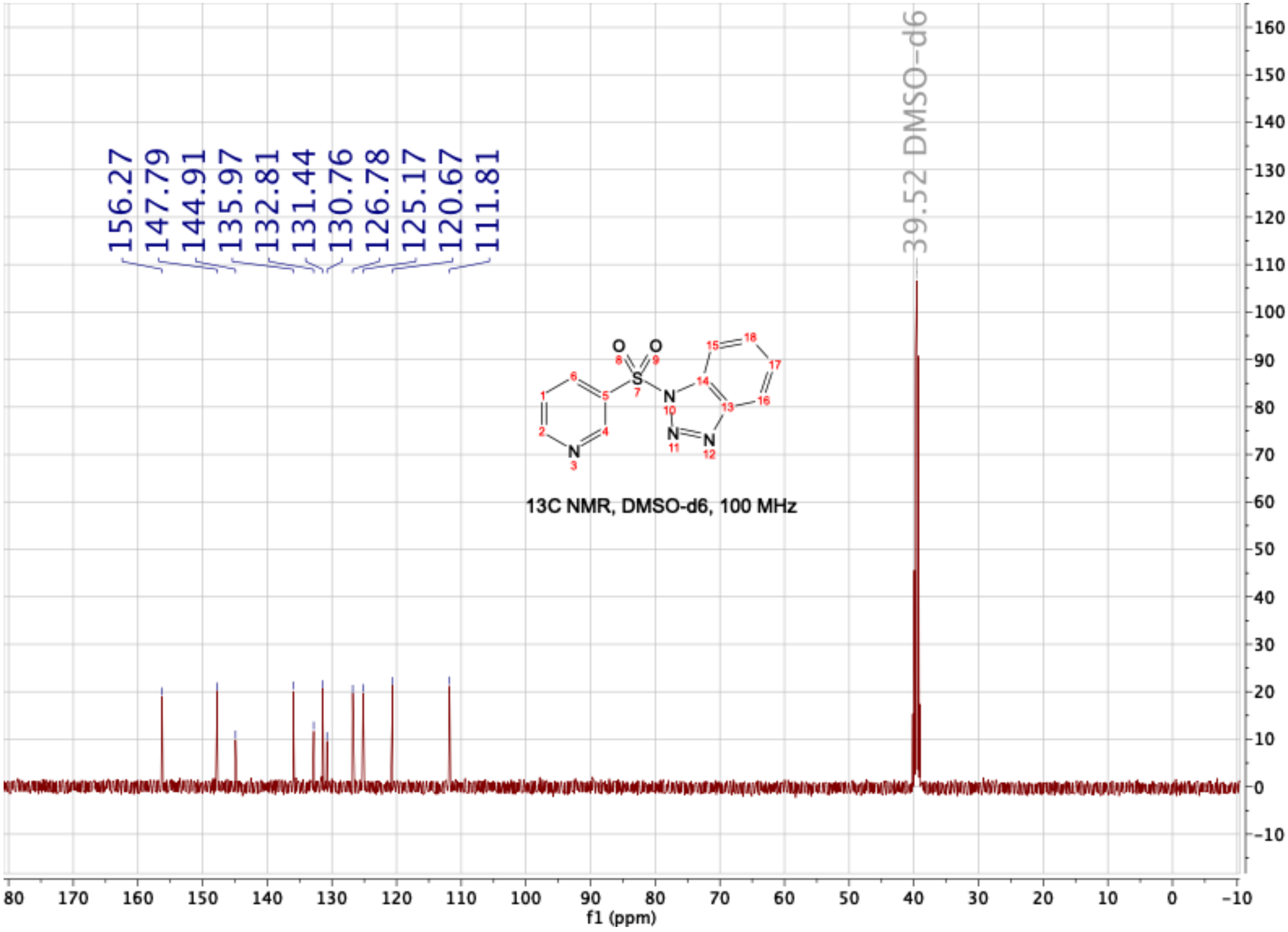

156.27
147.79
144.91
135.97
132.81
131.44
130.76
126.78
125.17
120.67
111.81
39.52 DMSO-d6
13C NMR, DMSO-d6, 100 MHz
f1 (ppm)

18

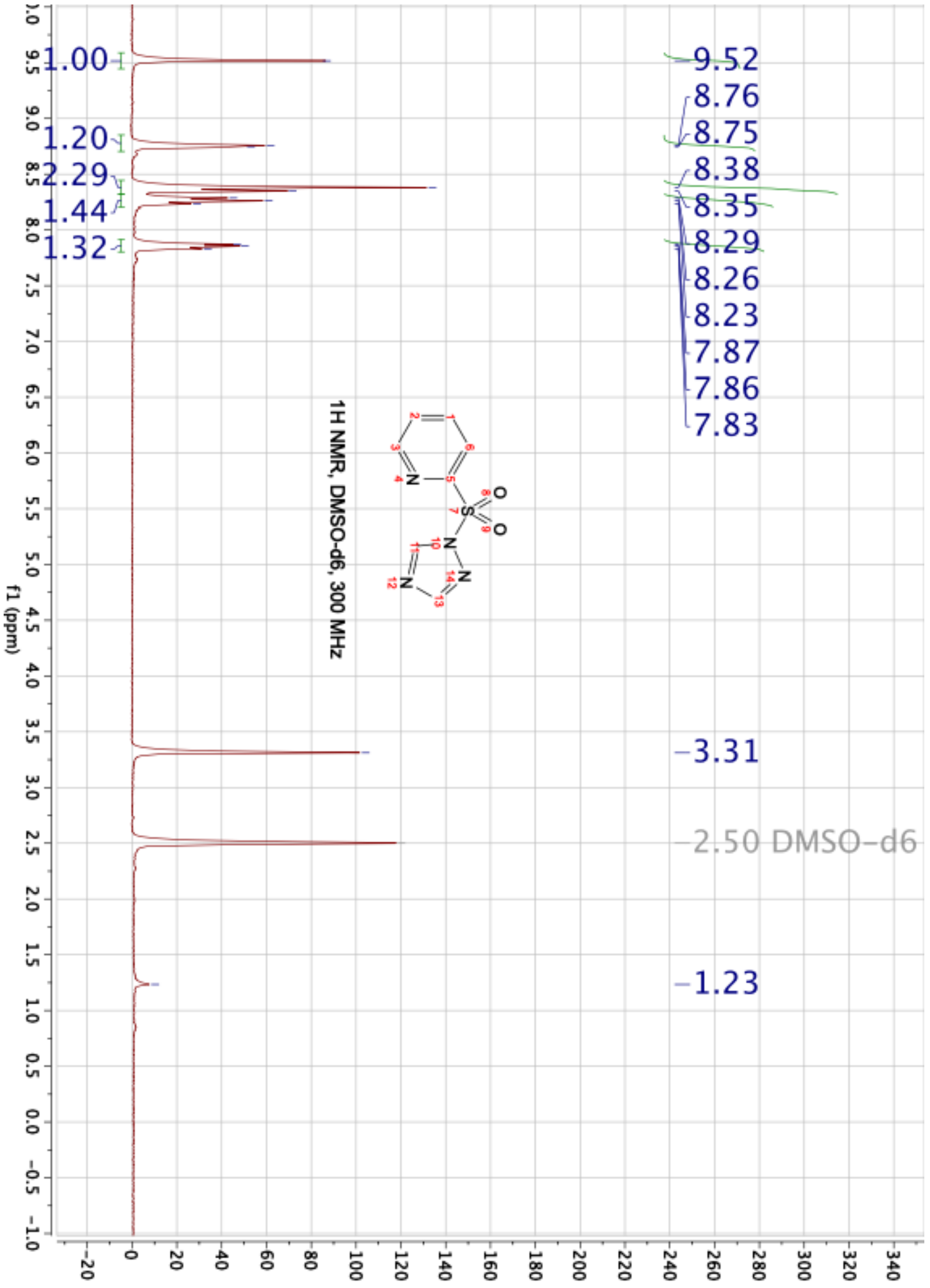
1H NMR, DMSO-d6, 300 MHz
9.52
8.76
8.75
8.38
8.35
8.29
8.26
8.23
7.87
7.86
7.83
3.31
2.50 DMSO-d6
1.23
1.00
1.20
2.29
1.44
1.32
f1 (ppm)

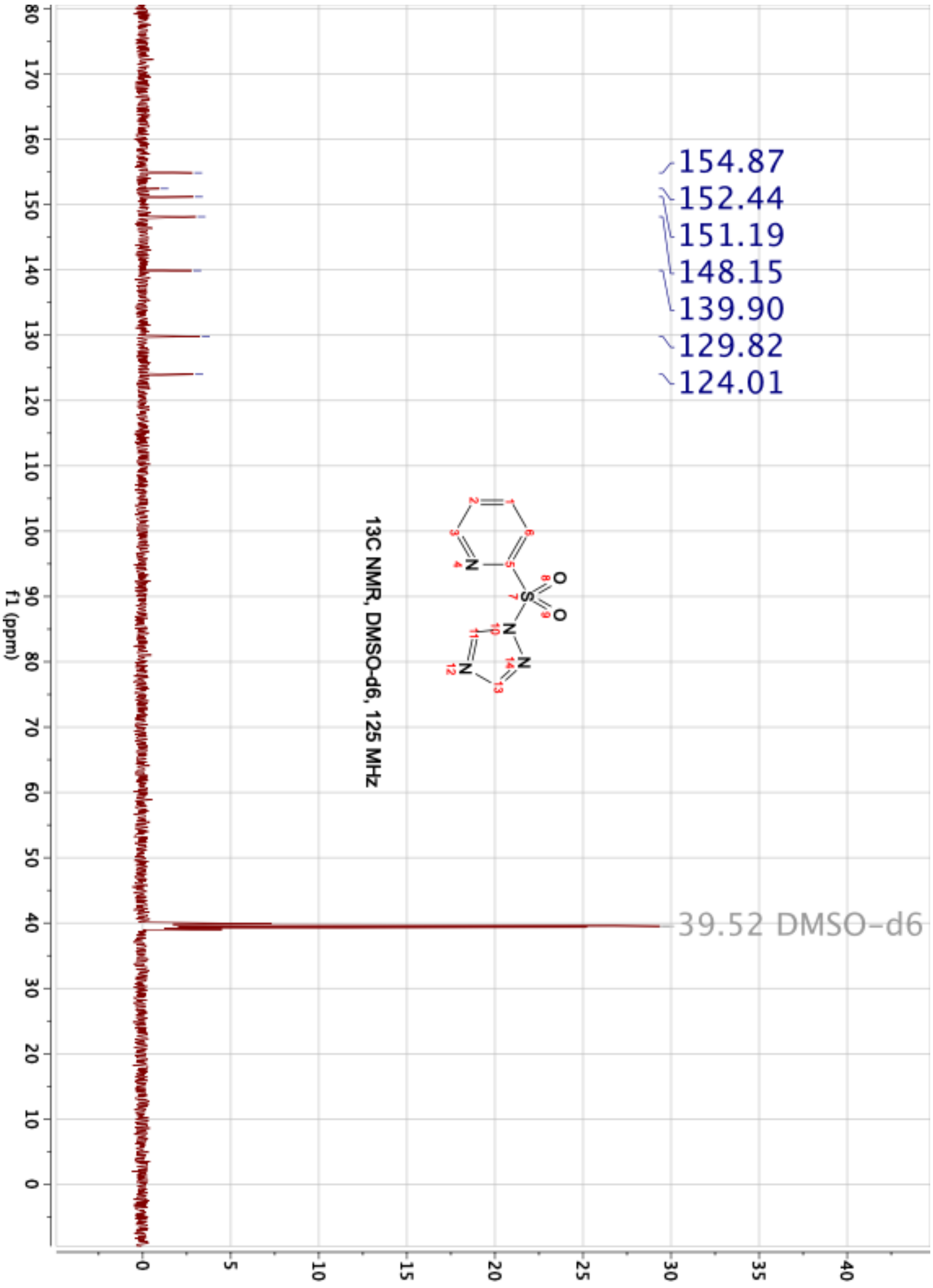

13C NMR, DMSO-d6, 125 MHz
154.87
152.44
151.19
148.15
139.90
129.82
124.01
39.52 DMSO-d6
f1 (ppm)

19 (CP5S)

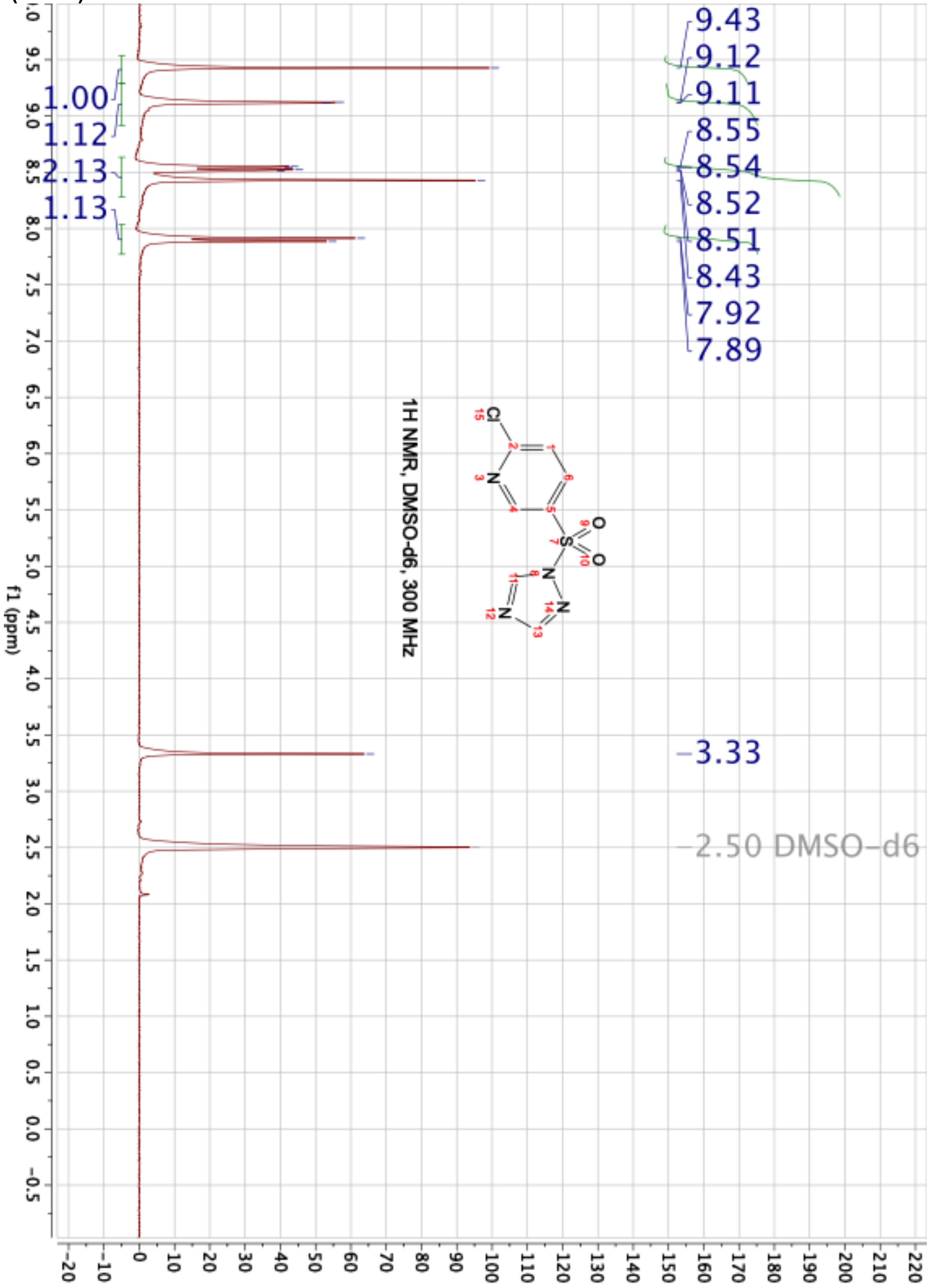

1H NMR, DMSO-d6, 300 MHz
9.43
9.12
9.11
8.55
8.54
8.52
8.51
8.43
7.92
7.89
3.33
2.50 DMSO-d6
1.00
1.12
2.13
1.13
f1 (ppm)

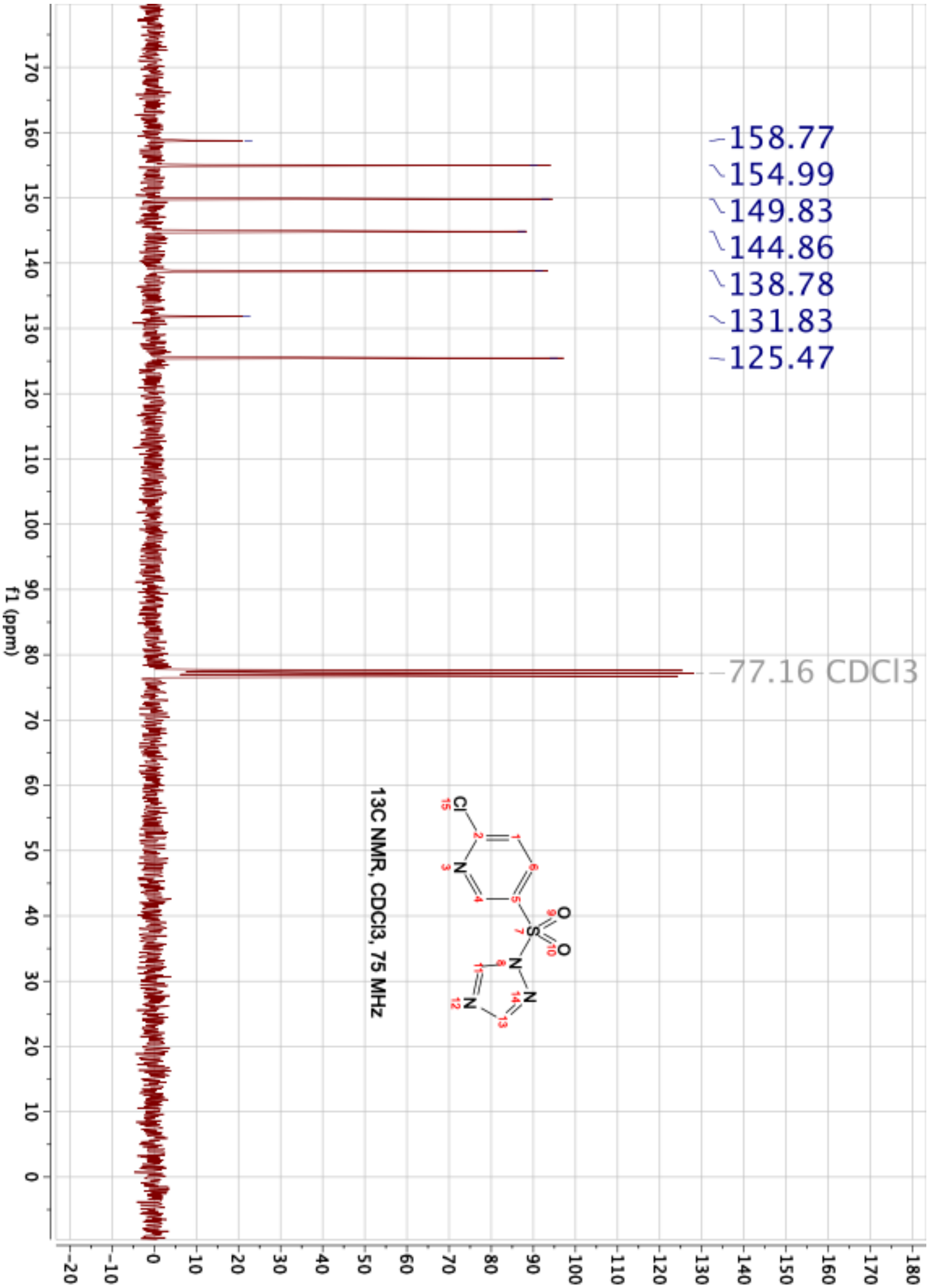
158.77
154.99
149.83
144.86
138.78
131.83
125.47
77.16 CDCl3
13C NMR, CDCl3, 75 MHz
f1 (ppm)

20

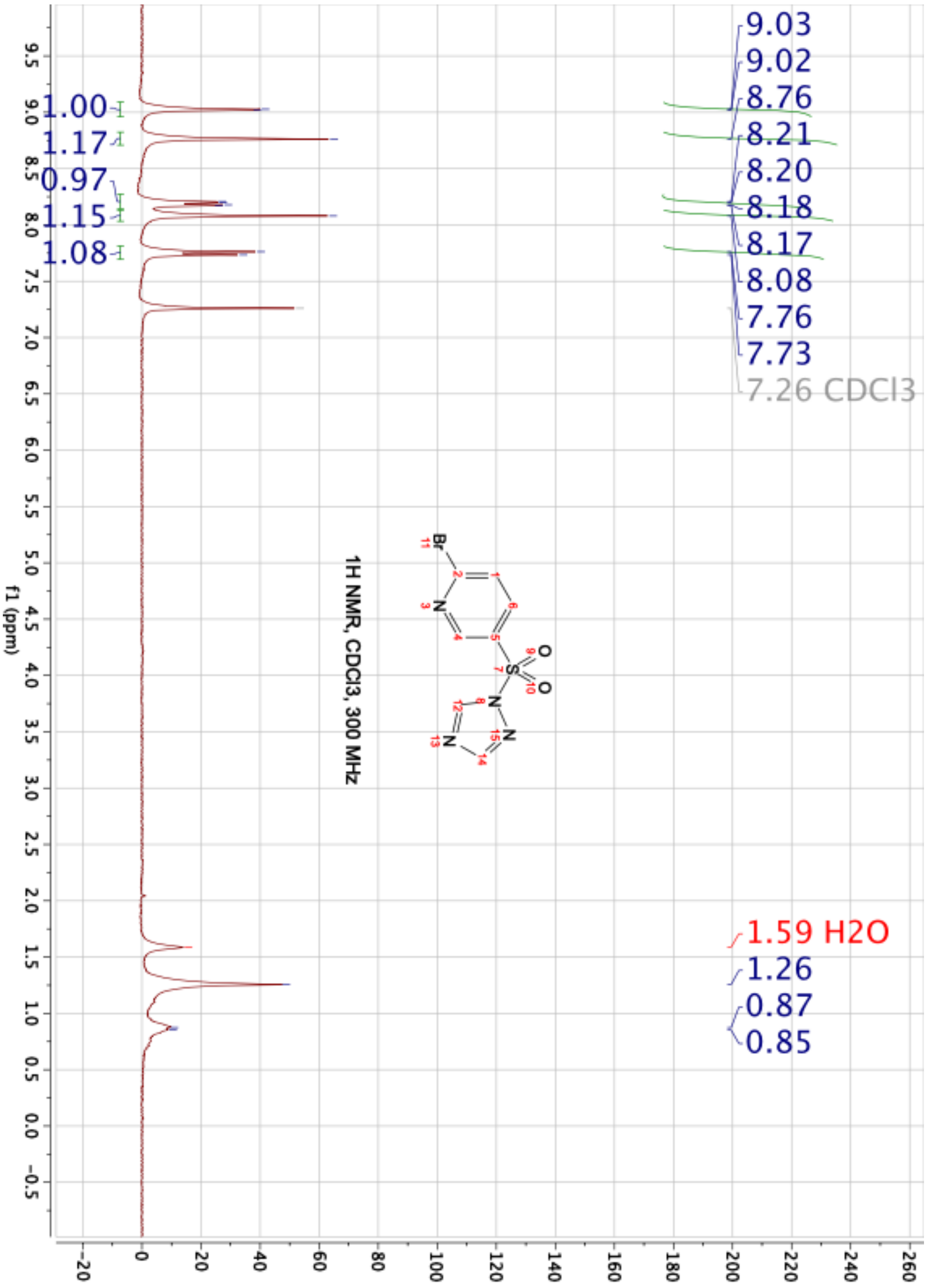

1H NMR, CDCl3, 300 MHz
9.03
9.02
8.76
8.21
8.20
8.18
8.17
8.08
7.76
7.73
7.26 CDCl3
1.59 H2O
1.26
0.87
0.85
1.00
1.17
0.97
1.15
1.08
f1 (ppm)

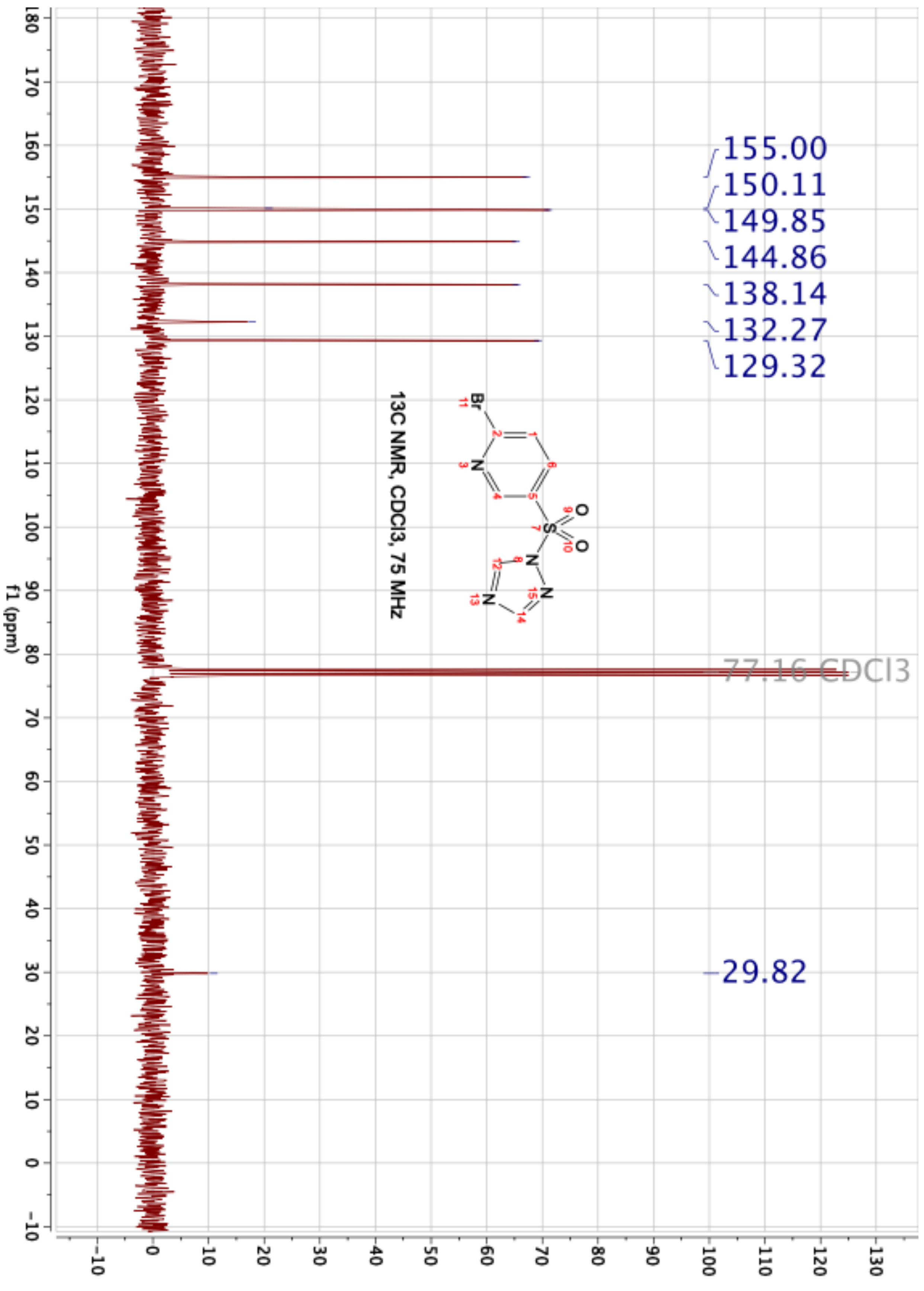
13C NMR, CDCl3, 75 MHz
155.00
150.11
149.85
144.86
138.14
132.27
129.32
77.16 CDCl3
29.82
f1 (ppm)

21

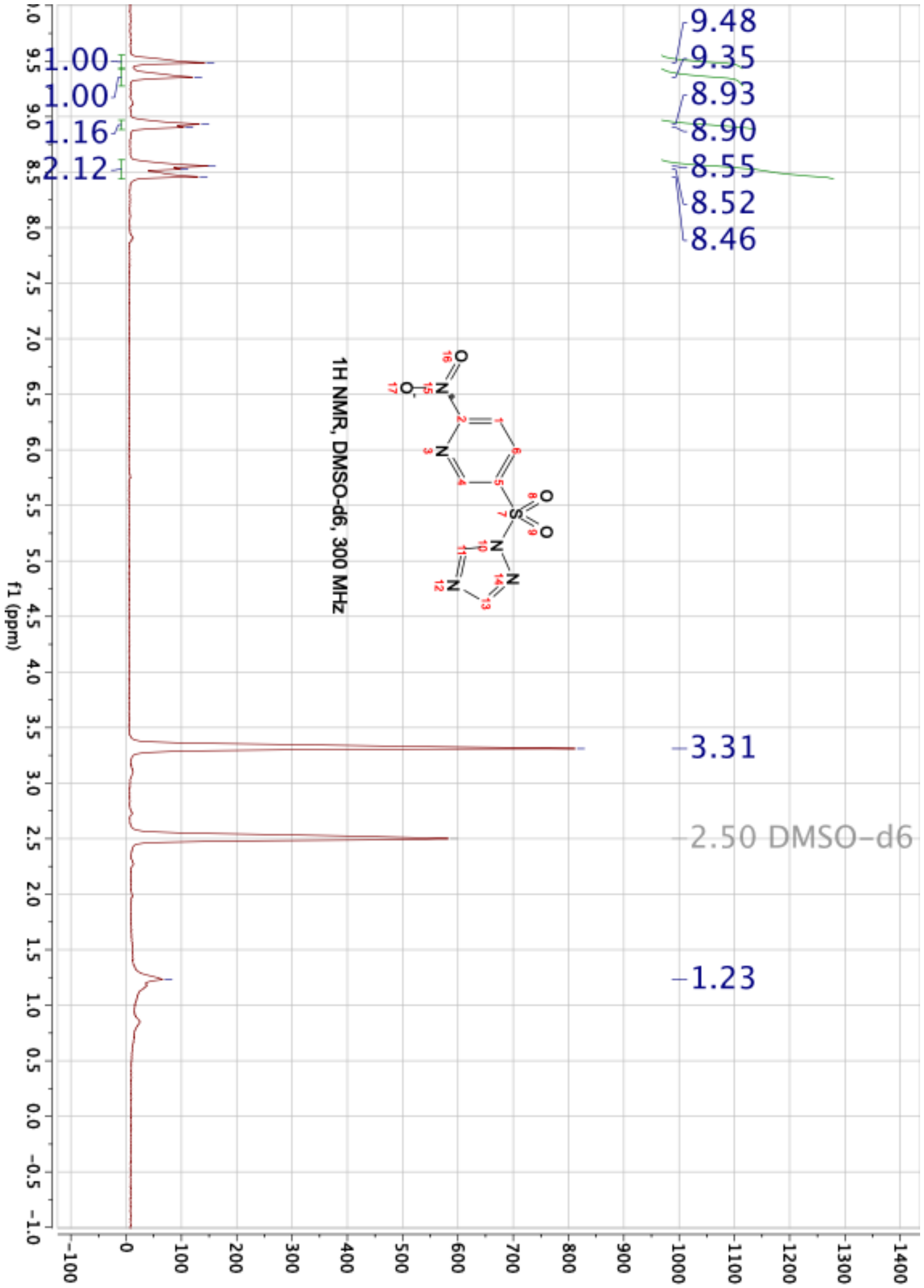

1H NMR, DMSO-d6, 300 MHz
9.48
9.35
8.93
8.90
8.55
8.52
8.46
3.31
2.50 DMSO-d6
1.23
1.00
1.00
1.16
2.12
f1 (ppm)

22

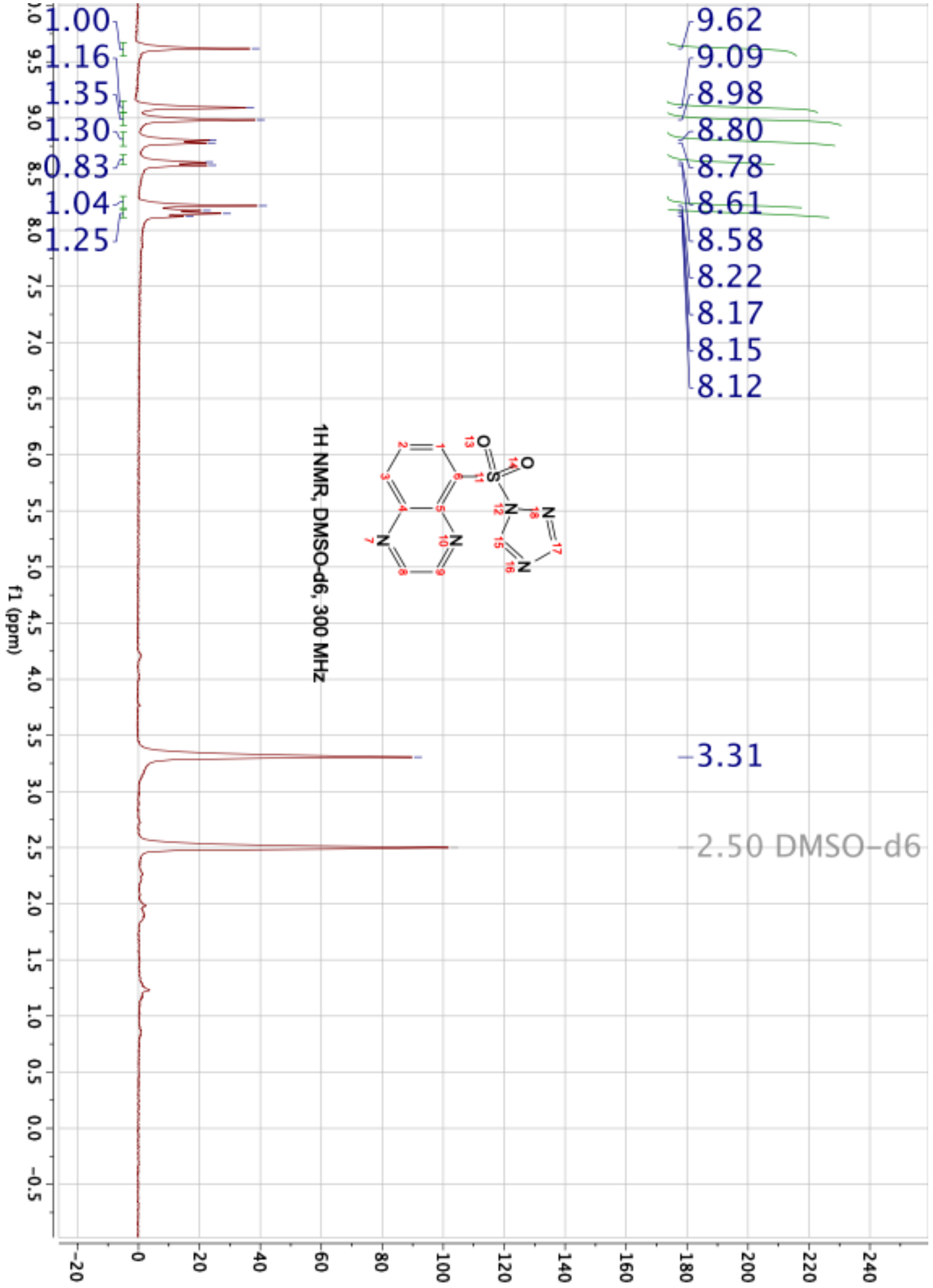
1H NMR, DMSO-d6, 300 MHz
9.62
9.09
8.98
8.80
8.78
8.61
8.58
8.22
8.17
8.15
8.12
3.31
2.50 DMSO-d6
1.00
1.16
1.35
1.30
0.83
1.04
1.25
f1 (ppm)

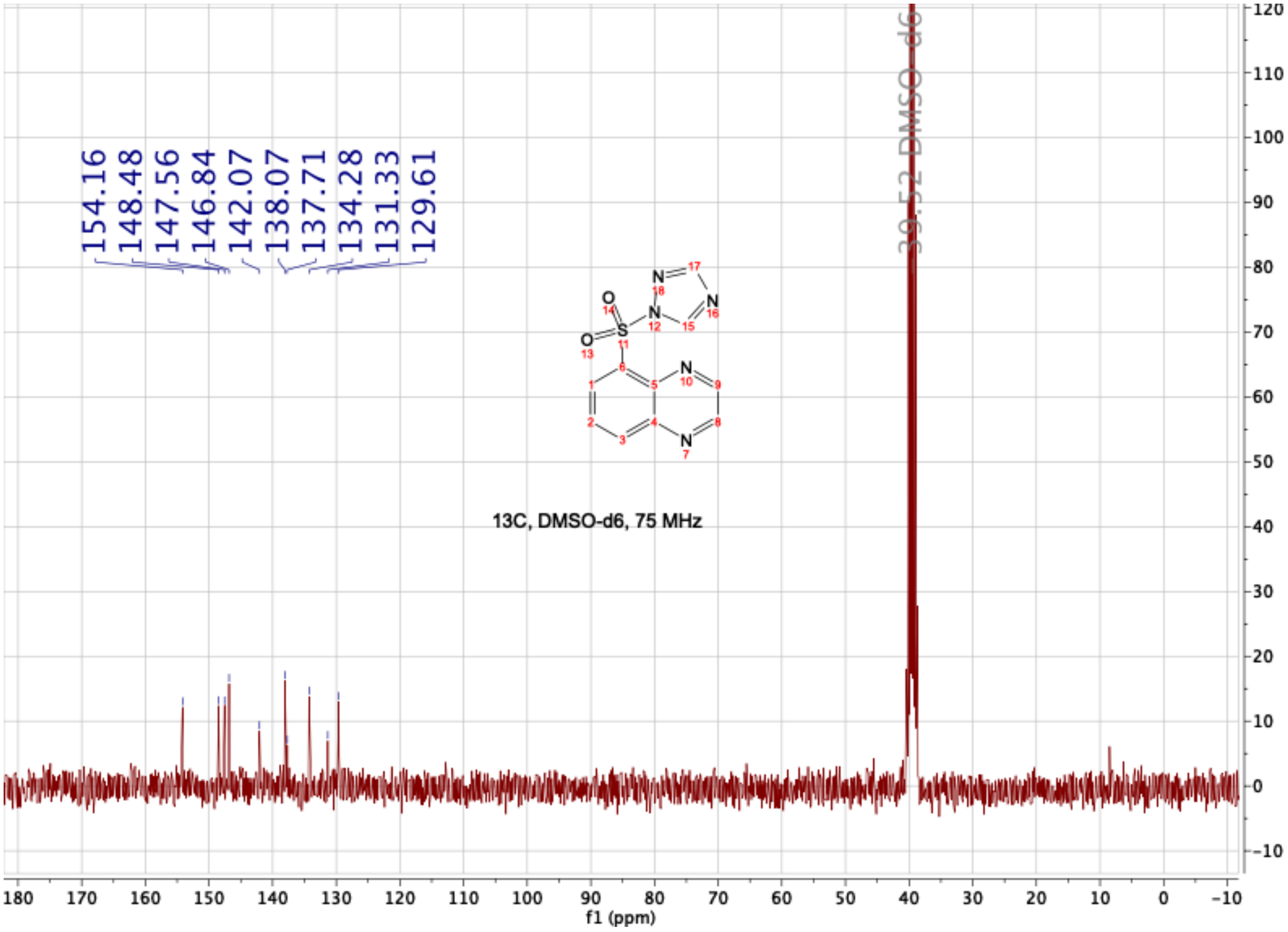
154.16
148.48
147.56
146.84
142.07
138.07
137.71
134.28
131.33
129.61
39.52 DMSO-d6
13C, DMSO-d6, 75 MHz
f1 (ppm)

23

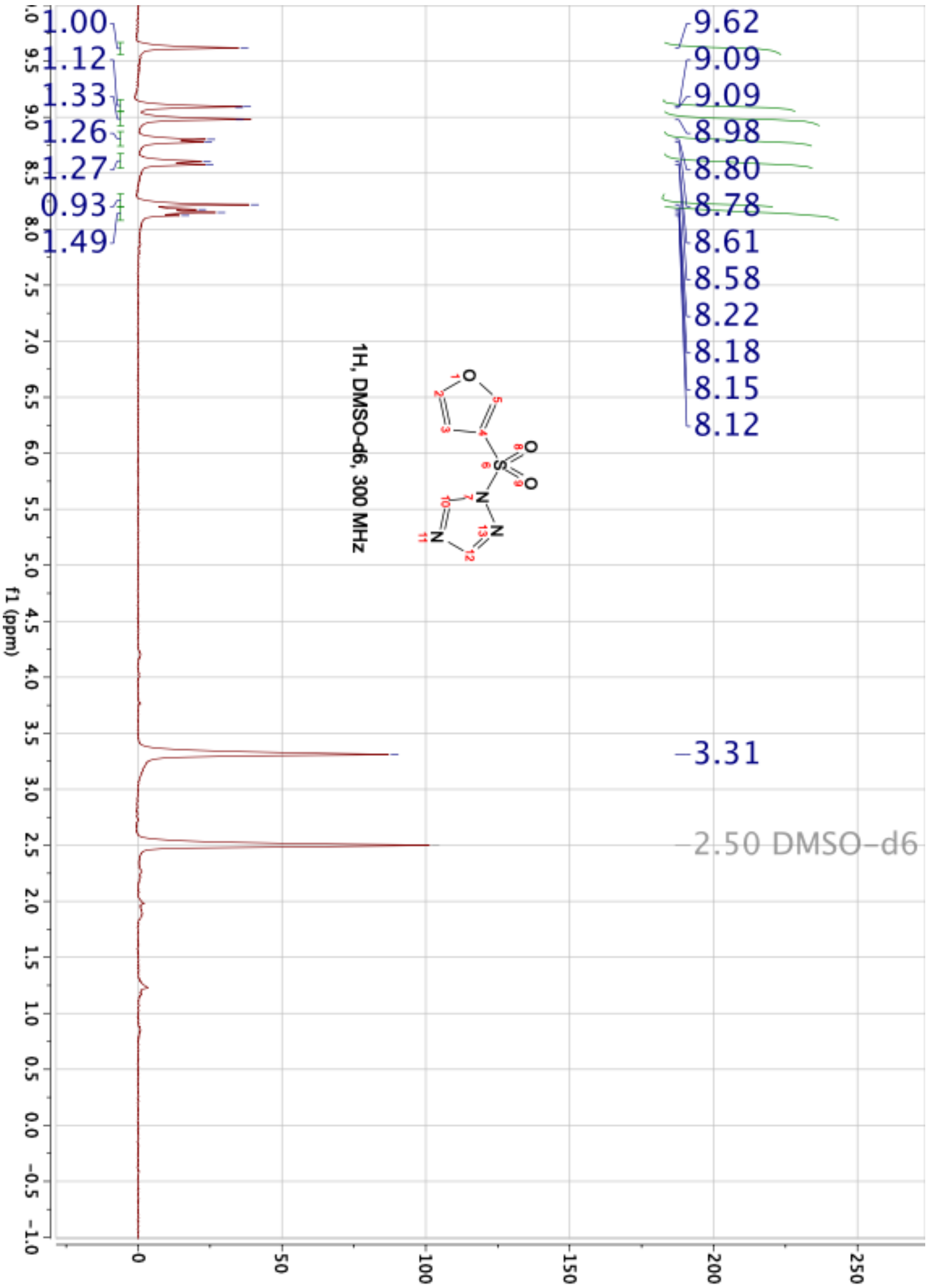
1H, DMSO-d6, 300 MHz
9.62
9.09
9.09
8.98
8.80
8.78
8.61
8.58
8.22
8.18
8.15
8.12
3.31
2.50 DMSO-d6
1.00
1.12
1.33
1.26
1.27
0.93
1.49
f1 (ppm)

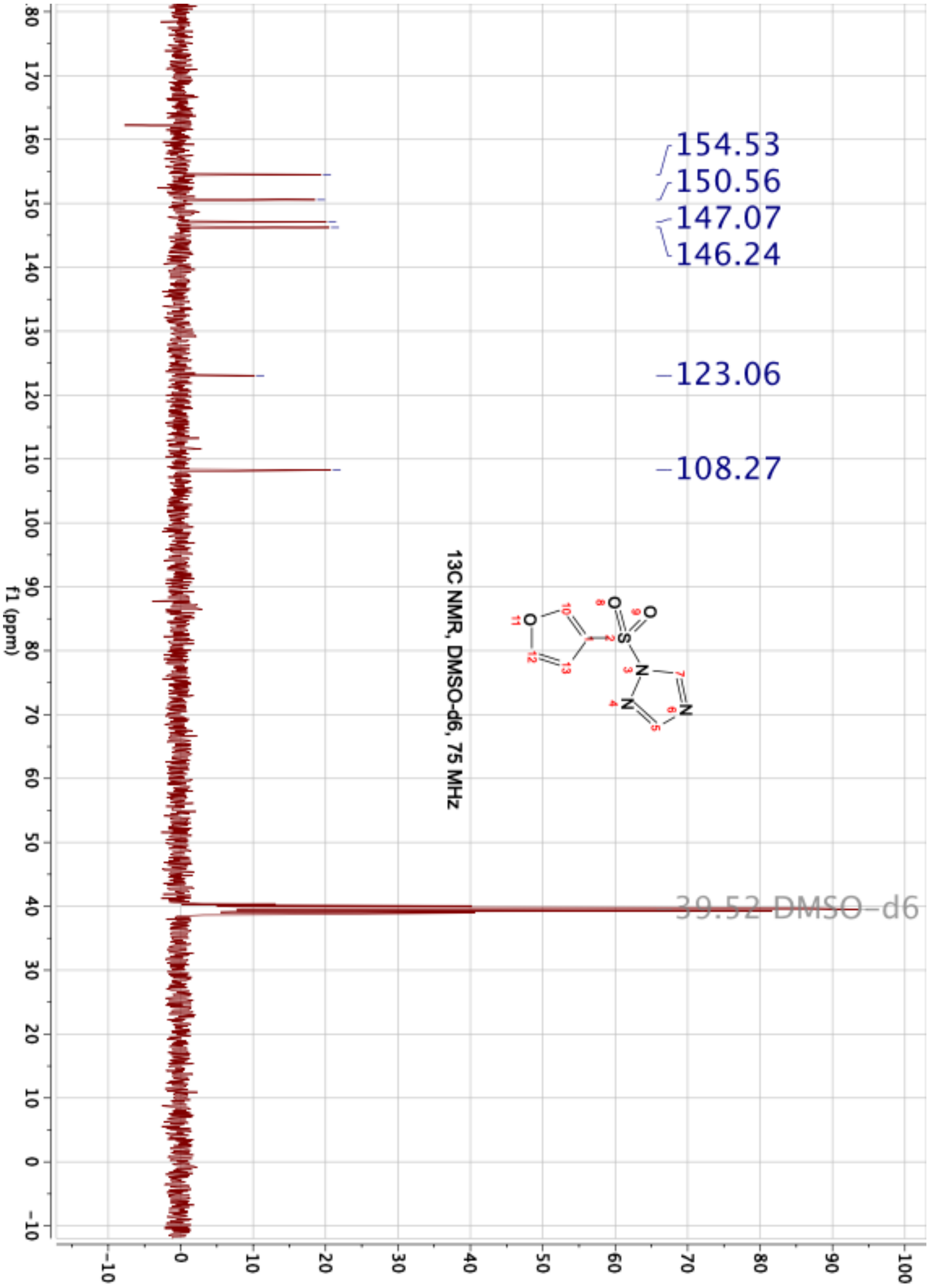

154.53
150.56
147.07
146.24
123.06
108.27
39.52 DMSO-d6
13C NMR, DMSO-d6, 75 MHz
f1 (ppm)

24

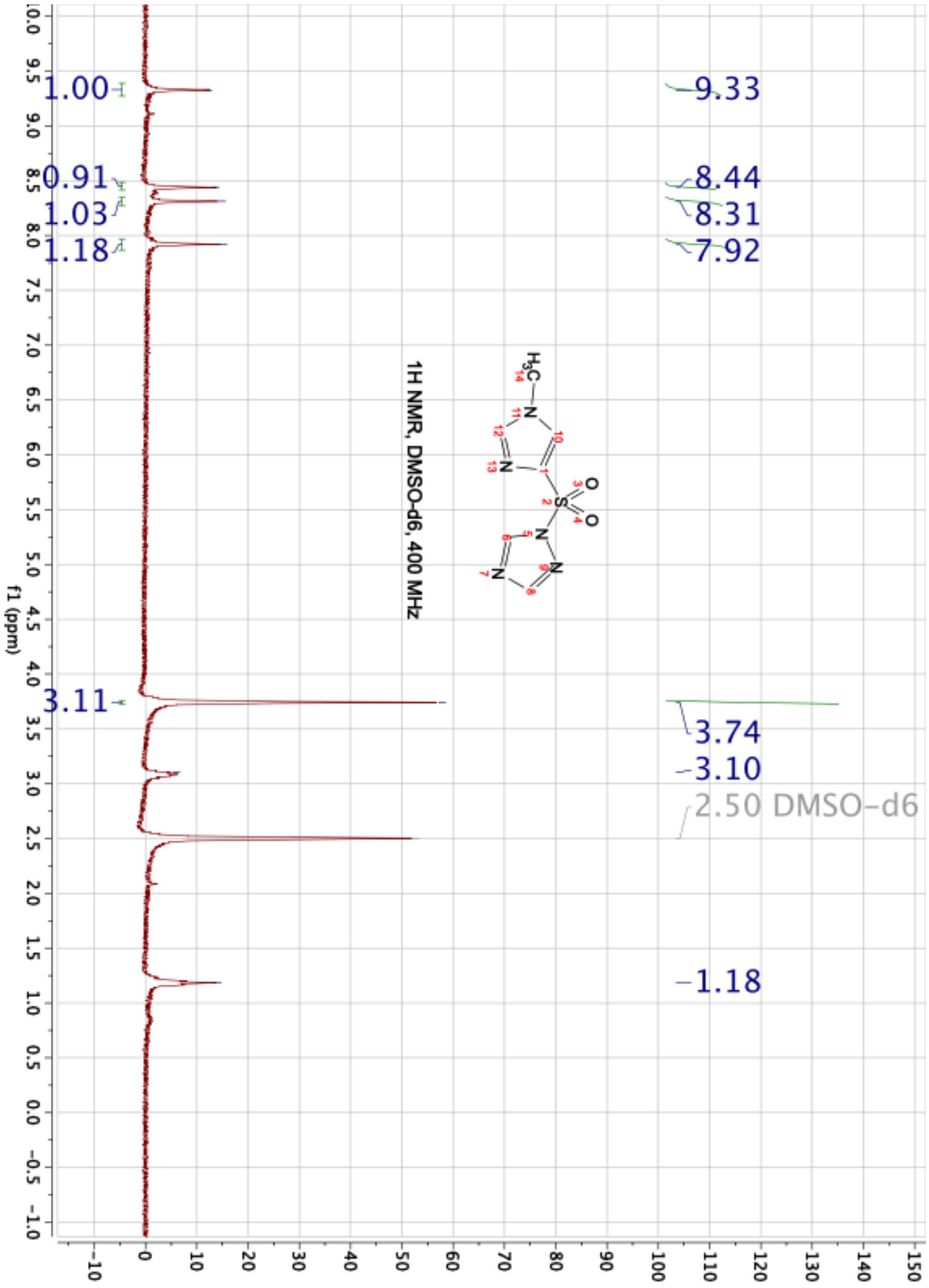
1H NMR, DMSO-d6, 400 MHz
9.33
8.44
8.31
7.92
3.74
3.10
2.50 DMSO-d6
1.18
1.00
0.91
1.03
1.18
3.11
f1 (ppm)

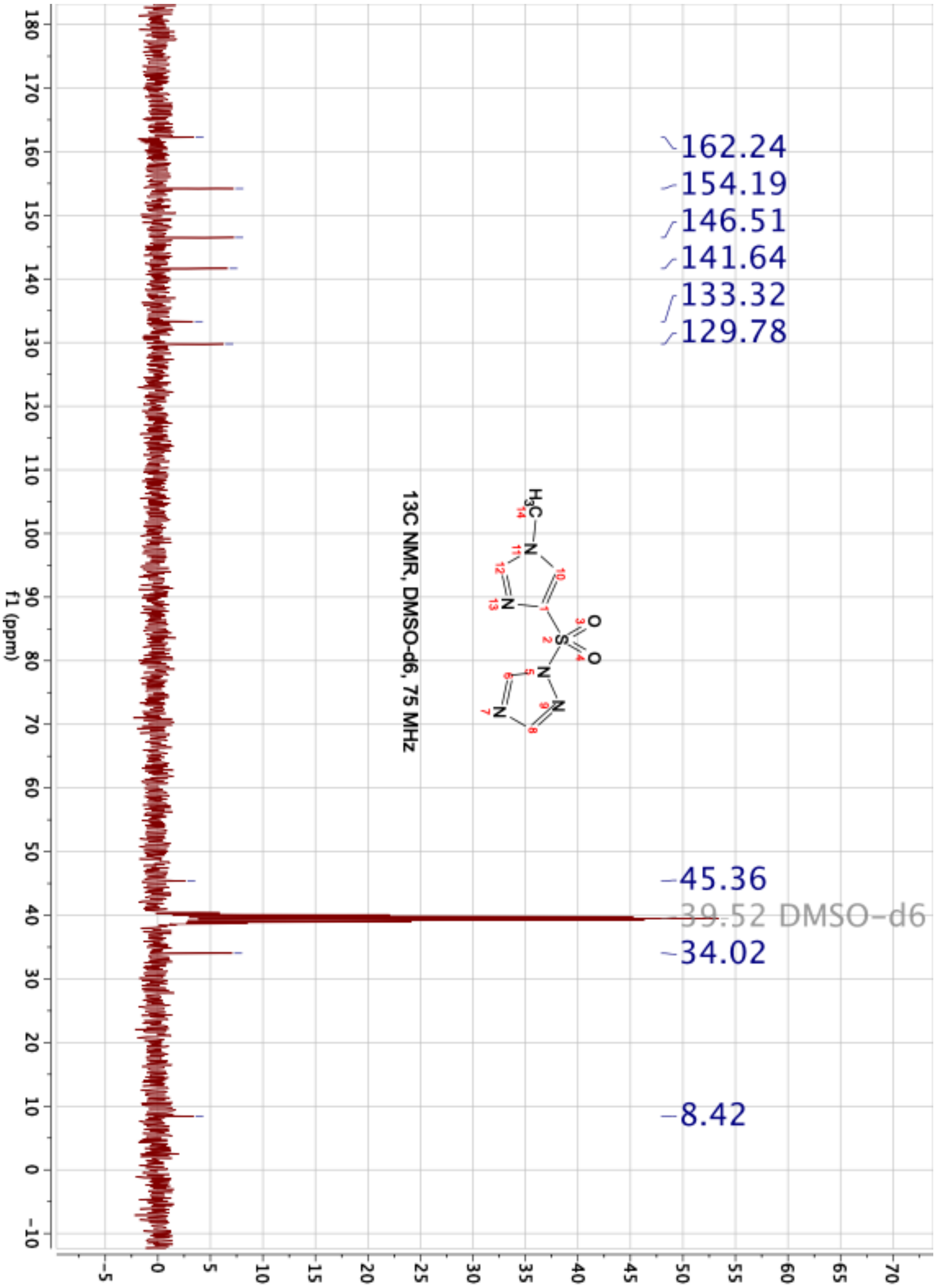
13C NMR, DMSO-d6, 75 MHz
162.24
154.19
146.51
141.64
133.32
129.78
45.36
39.52 DMSO-d6
34.02
8.42
f1 (ppm)

25

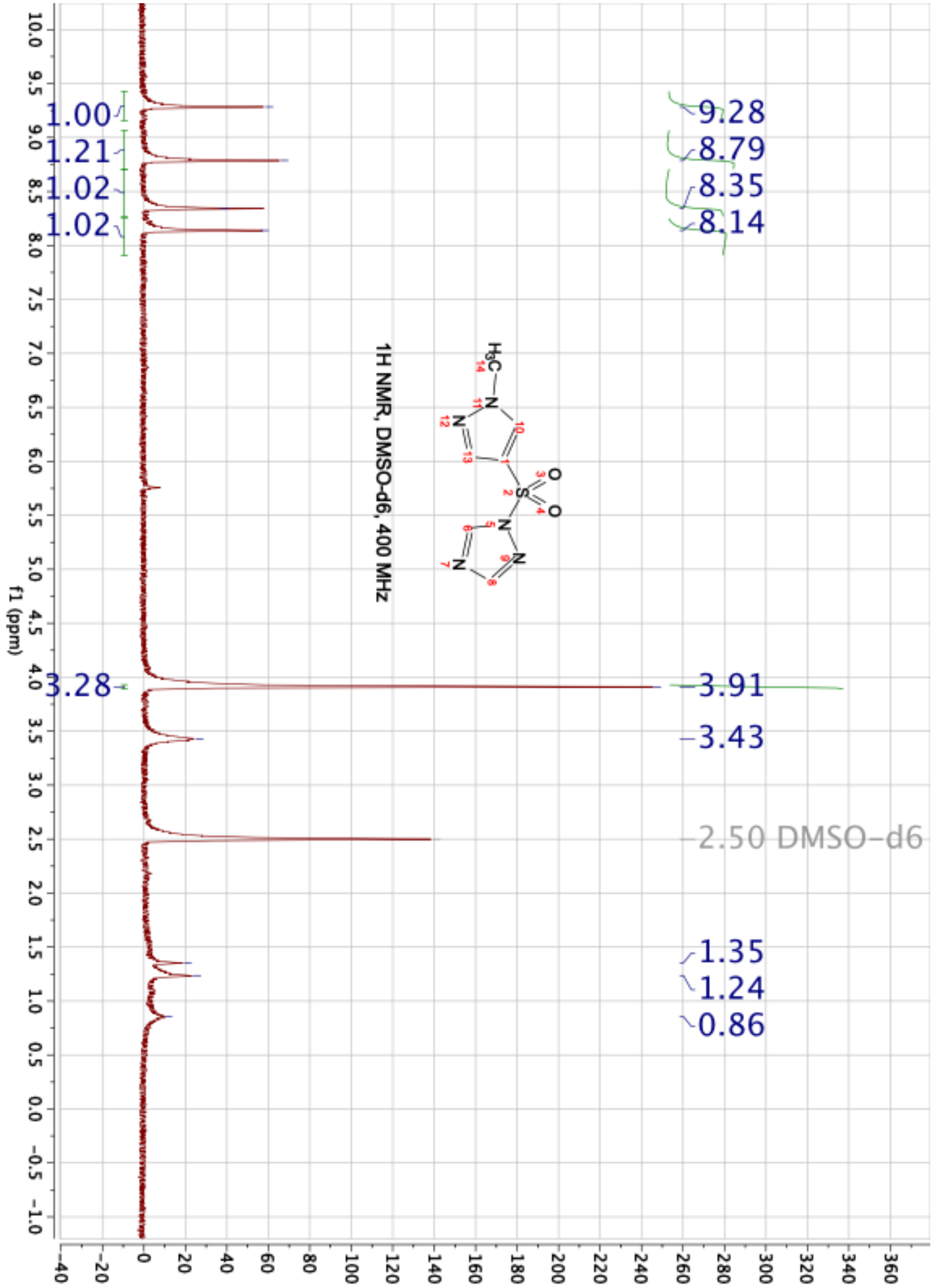
1H NMR, DMSO-d6, 400 MHz
9.28
8.79
8.35
8.14
3.91
3.43
2.50 DMSO-d6
1.35
1.24
0.86
1.00
1.21
1.02
1.02
3.28
f1 (ppm)

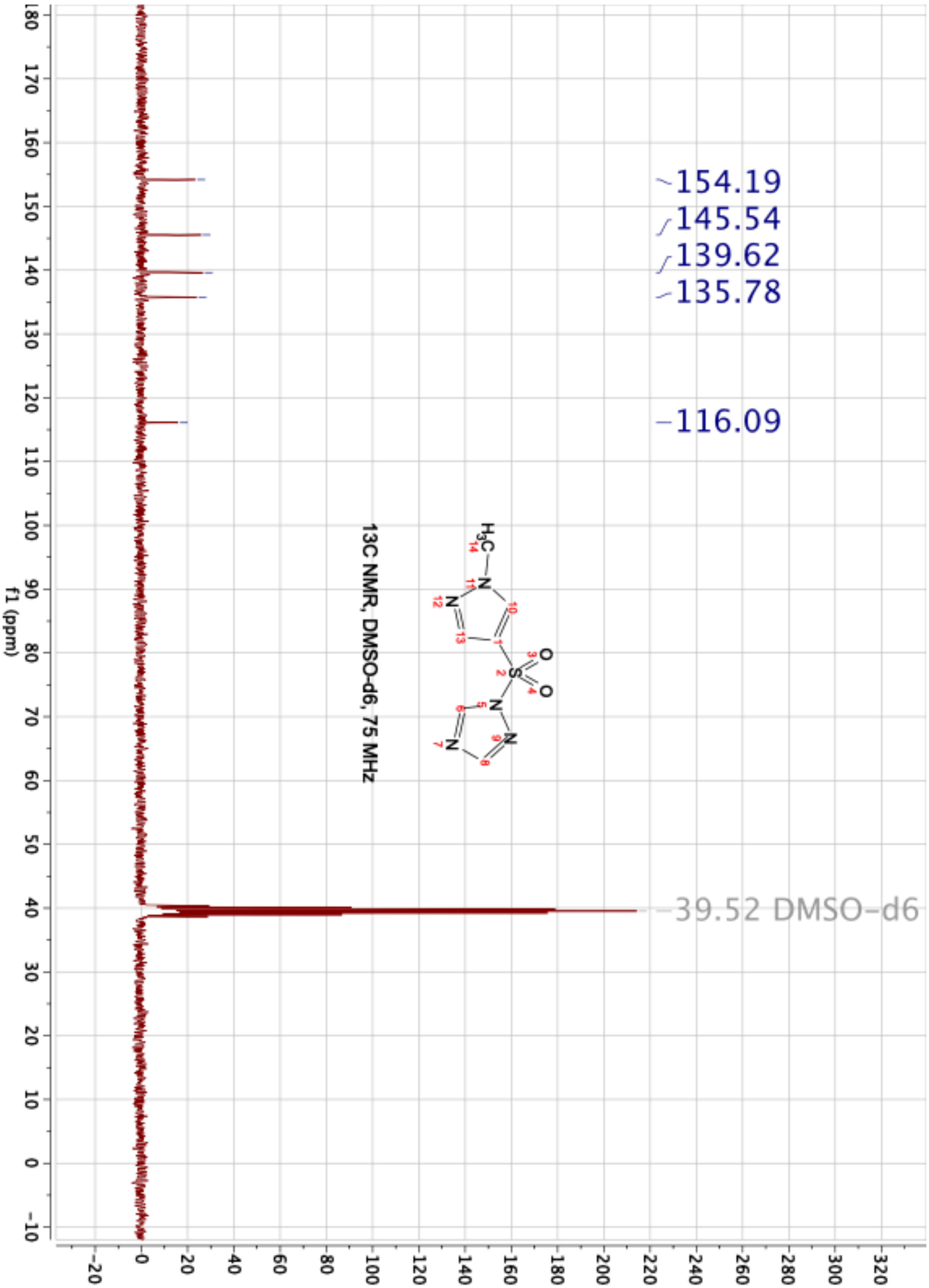
13C NMR, DMSO-d6, 75 MHz
154.19
145.54
139.62
135.78
116.09
39.52 DMSO-d6
f1 (ppm)

26 (AzP3S)

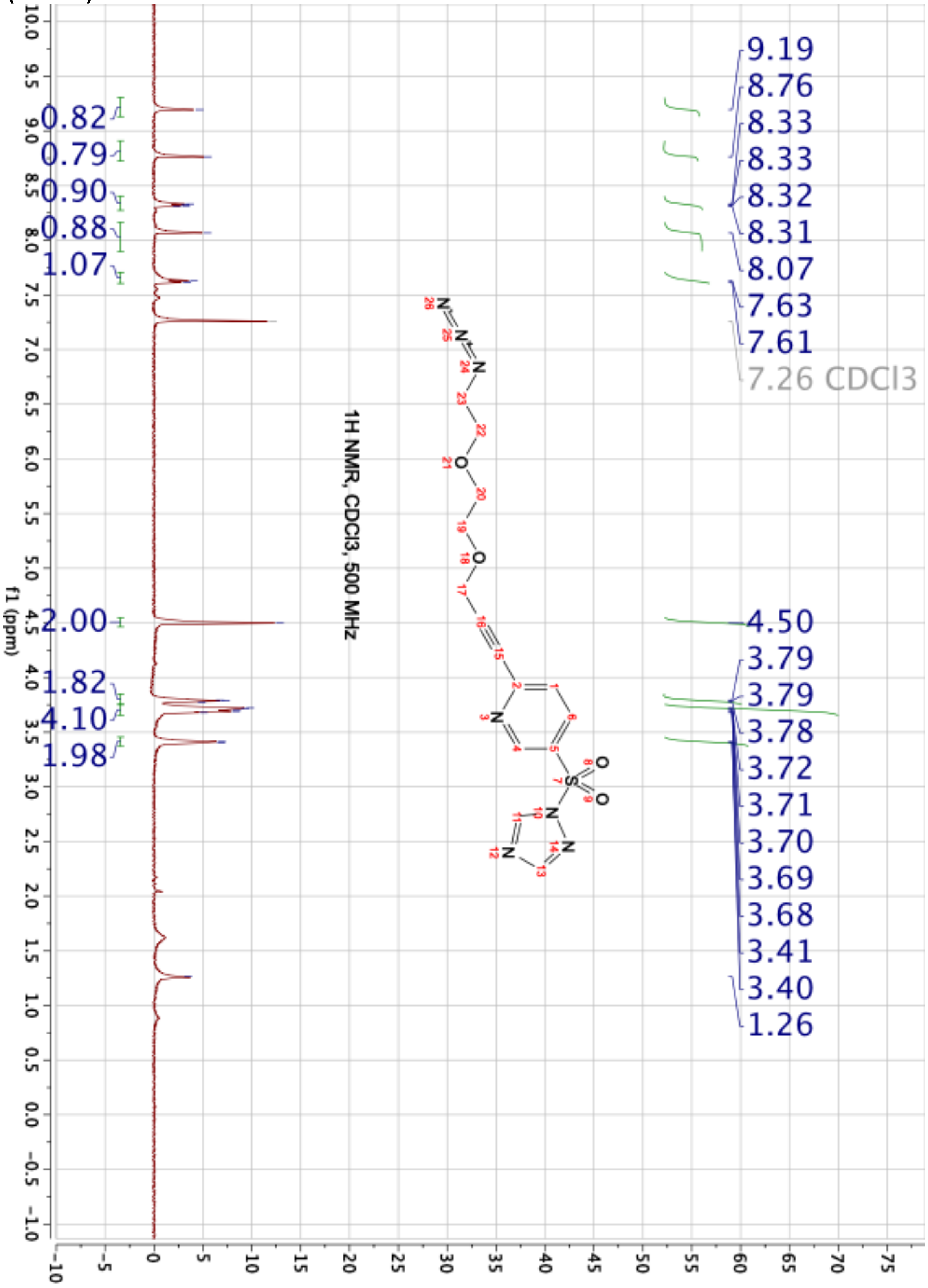
1H NMR, CDCl3, 500 MHz
f1 (ppm)
9.19
8.76
8.33
8.33
8.32
8.31
8.07
7.63
7.61
7.26 CDCl3
4.50
3.79
3.79
3.78
3.72
3.71
3.70
3.69
3.68
3.41
3.40
1.26
0.82
0.79
0.90
0.88
1.07
2.00
1.82
4.10
1.98

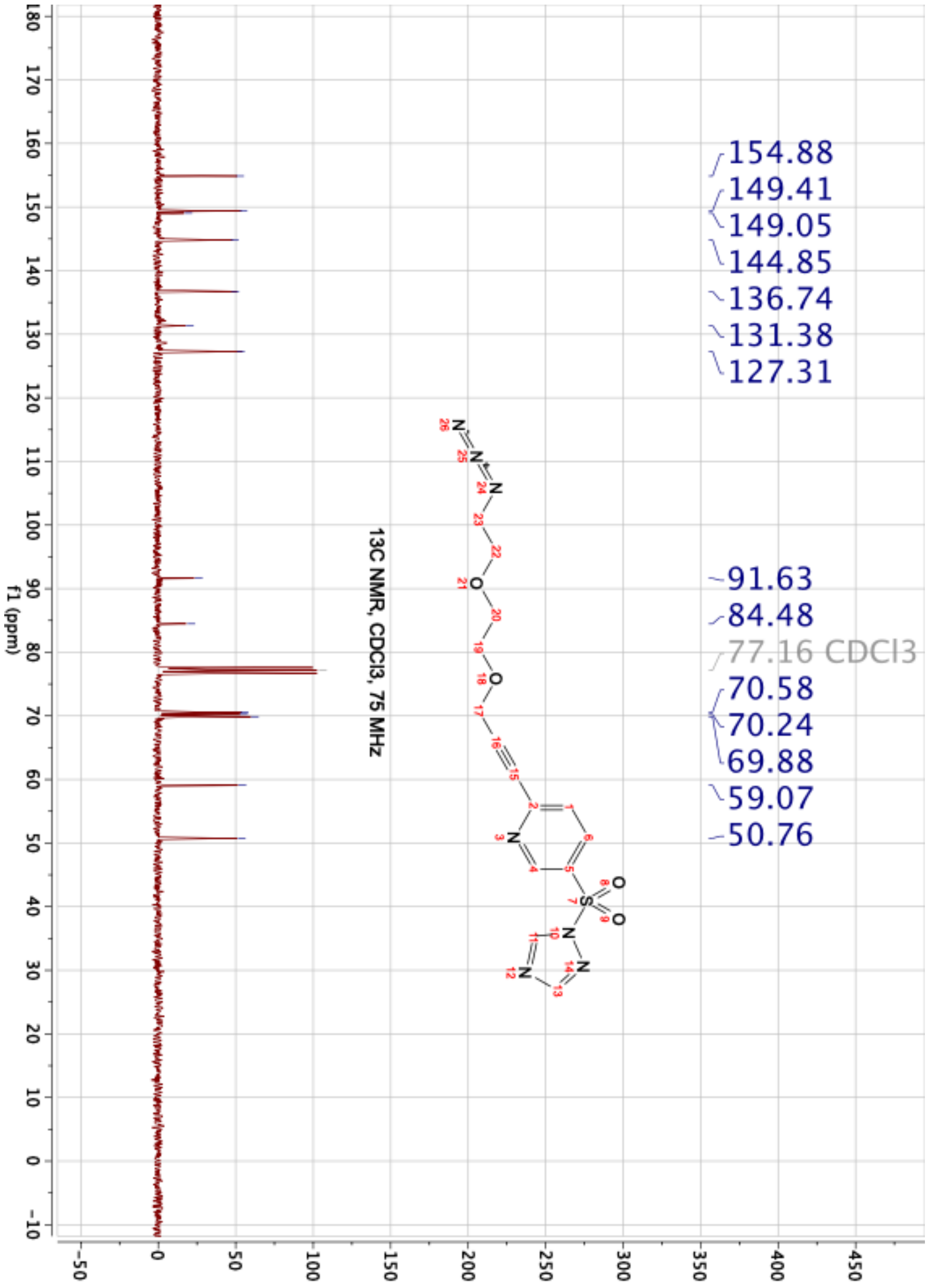
13C NMR, CDCl3, 75 MHz
154.88
149.41
149.05
144.85
136.74
131.38
127.31
91.63
84.48
77.16 CDCl3
70.58
70.24
69.88
59.07
50.76
f1 (ppm)

27

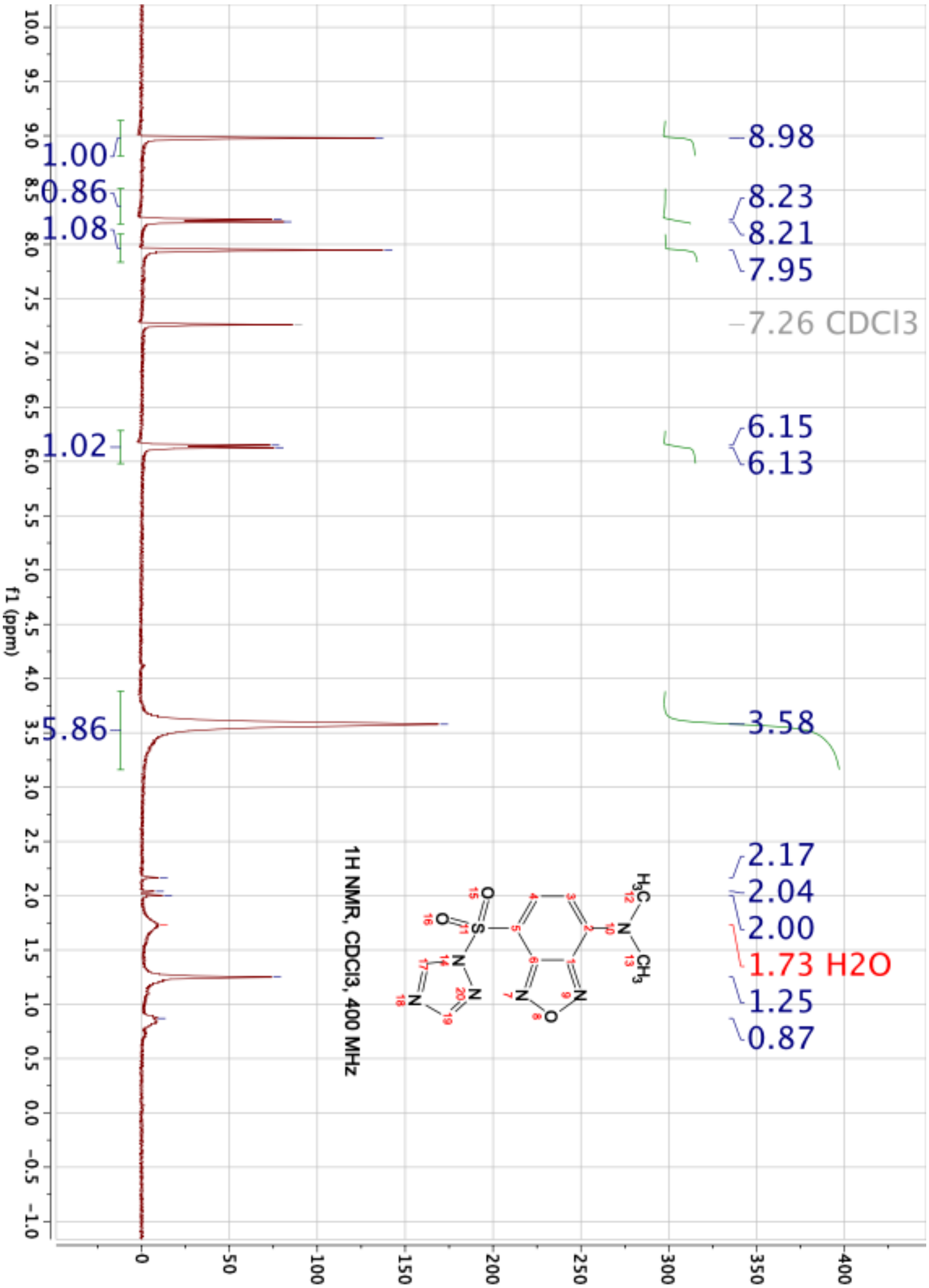
1H NMR, CDCl3, 400 MHz
8.98
8.23
8.21
7.95
7.26 CDCl3
6.15
6.13
3.58
2.17
2.04
2.00
1.73 H2O
1.25
0.87
1.00
0.86
1.08
1.02
5.86
f1 (ppm)

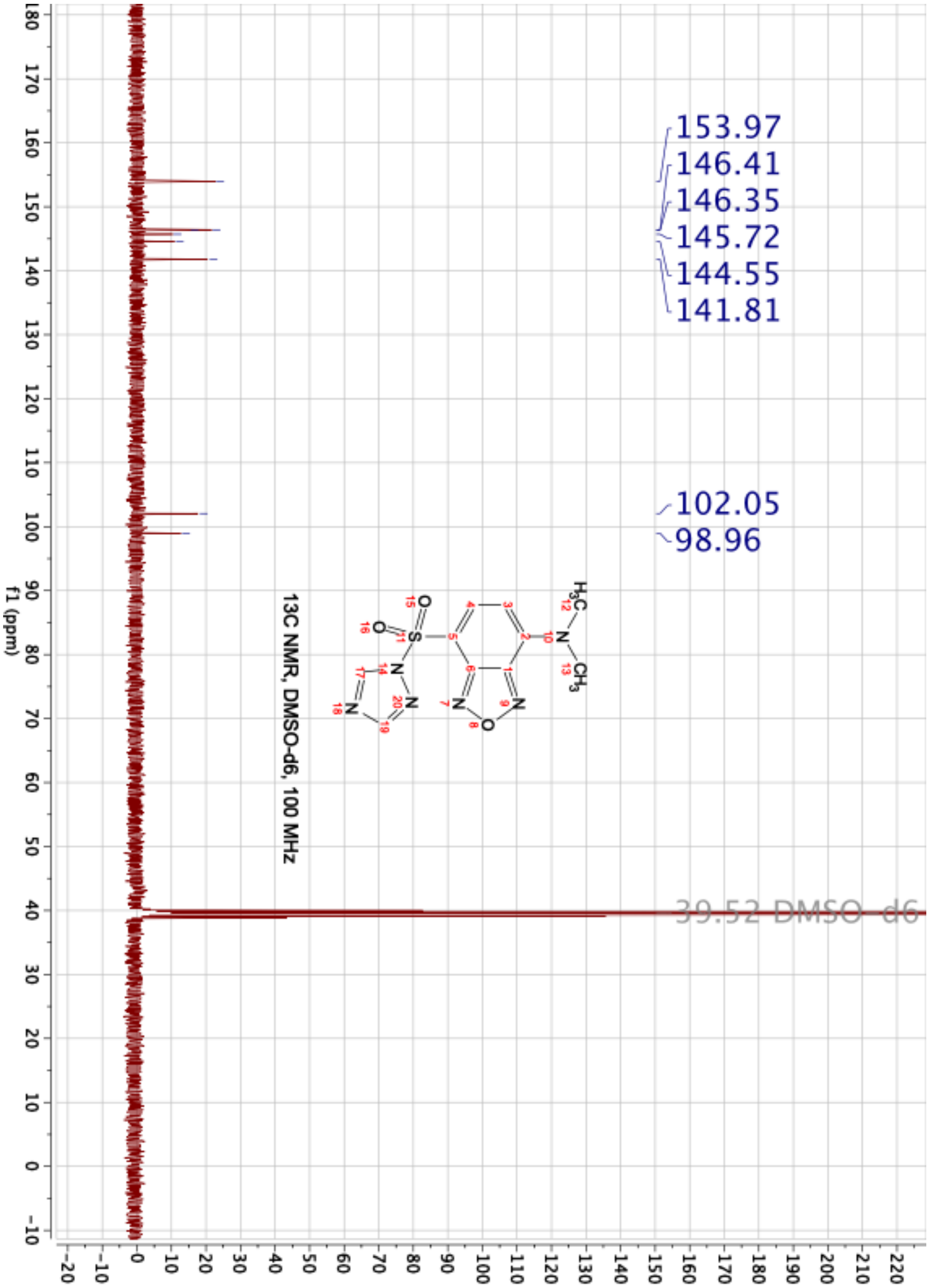
153.97
146.41
146.35
145.72
144.55
141.81
102.05
98.96
39.52 DMSO-d6
13C NMR, DMSO-d6, 100 MHz
f1 (ppm)

28

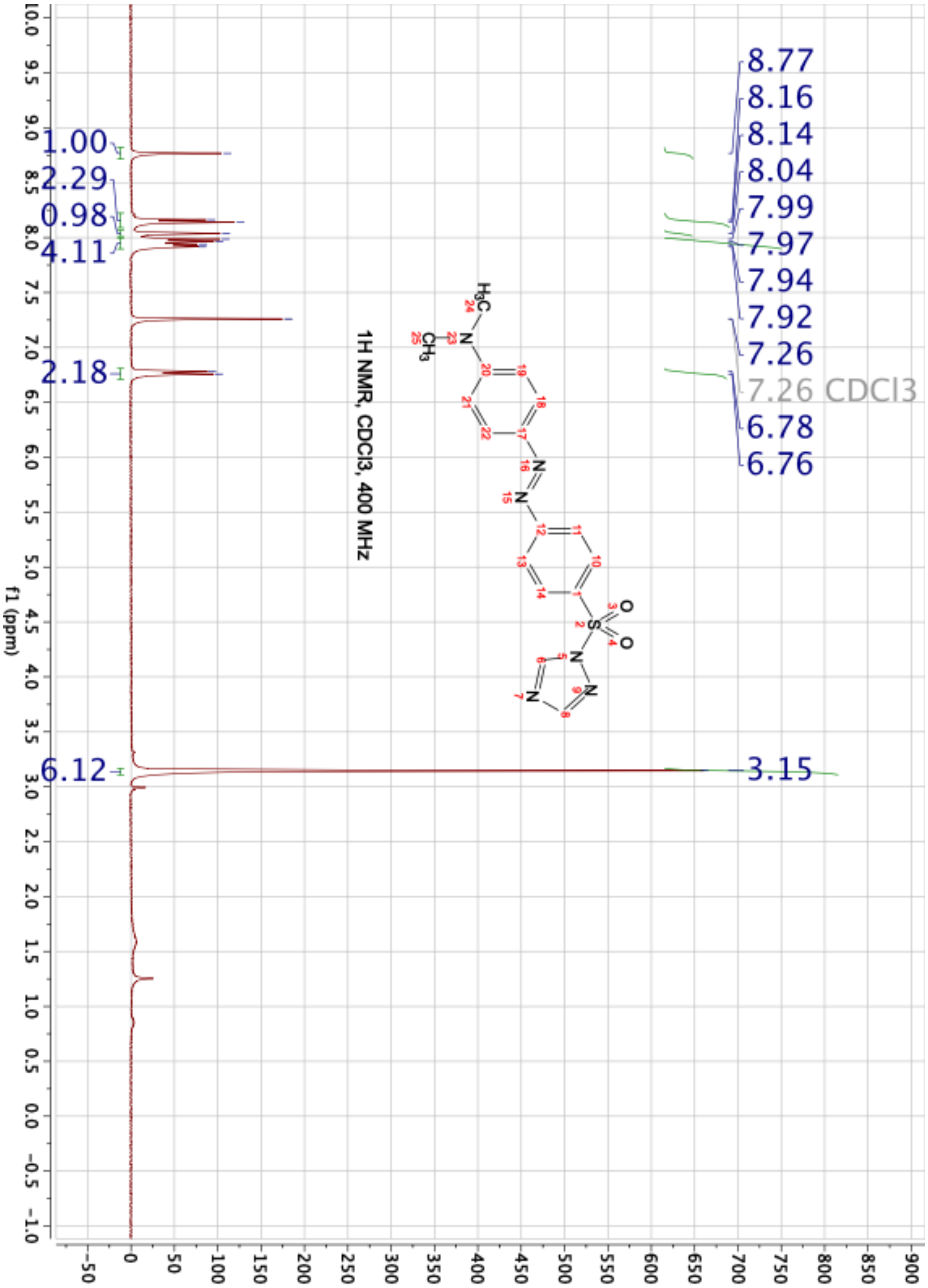
1H NMR, CDCl3, 400 MHz
8.77
8.16
8.14
8.04
7.99
7.97
7.94
7.92
7.26
7.26 CDCl3
6.78
6.76
3.15
1.00
2.29
0.98
4.11
2.18
6.12
f1 (ppm)

29

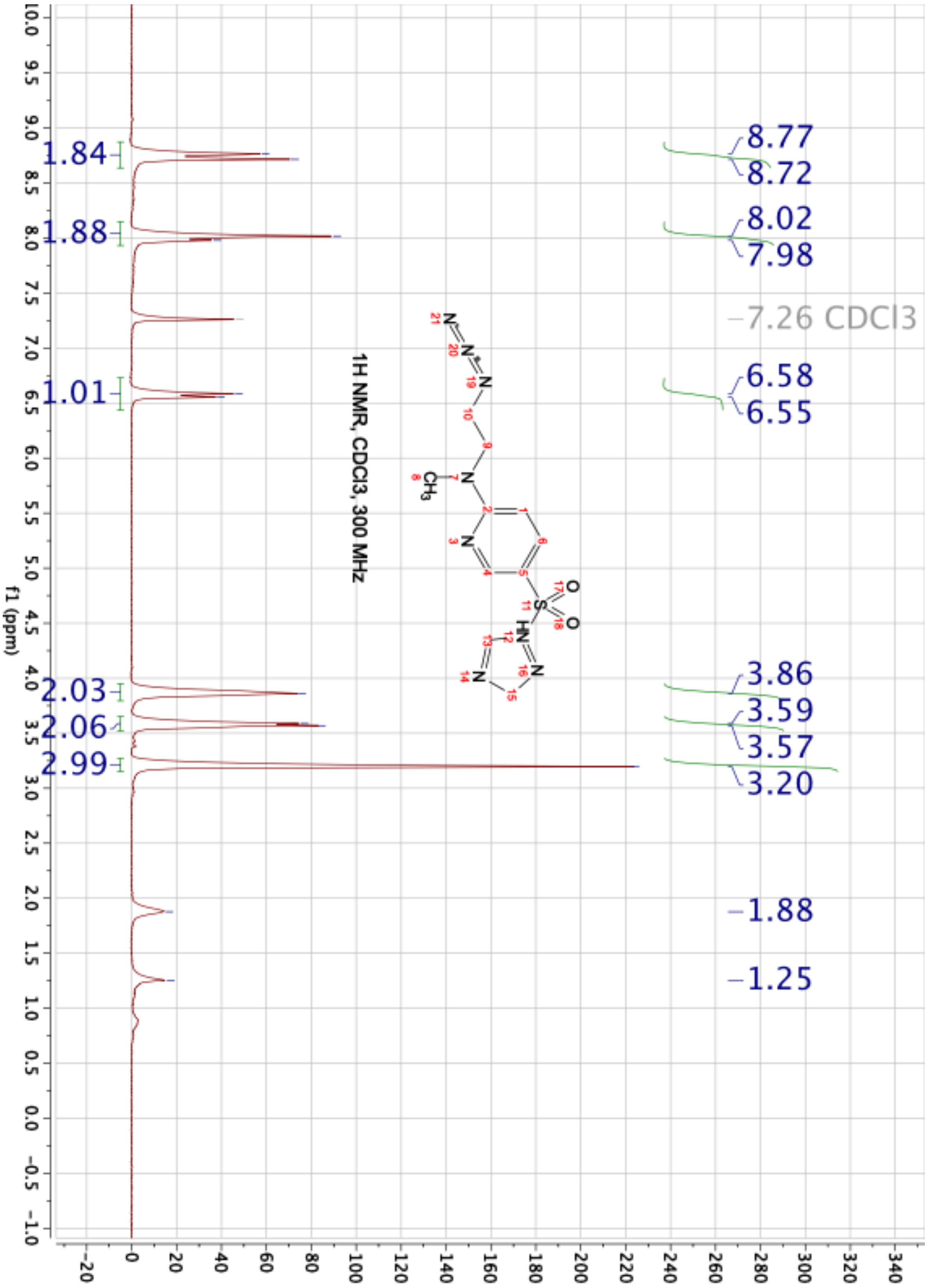
1H NMR, CDCl3, 300 MHz
8.77
8.72
8.02
7.98
7.26 CDCl3
6.58
6.55
3.86
3.59
3.57
3.20
1.88
1.25
1.84
1.88
1.01
2.03
2.06
2.99
f1 (ppm)

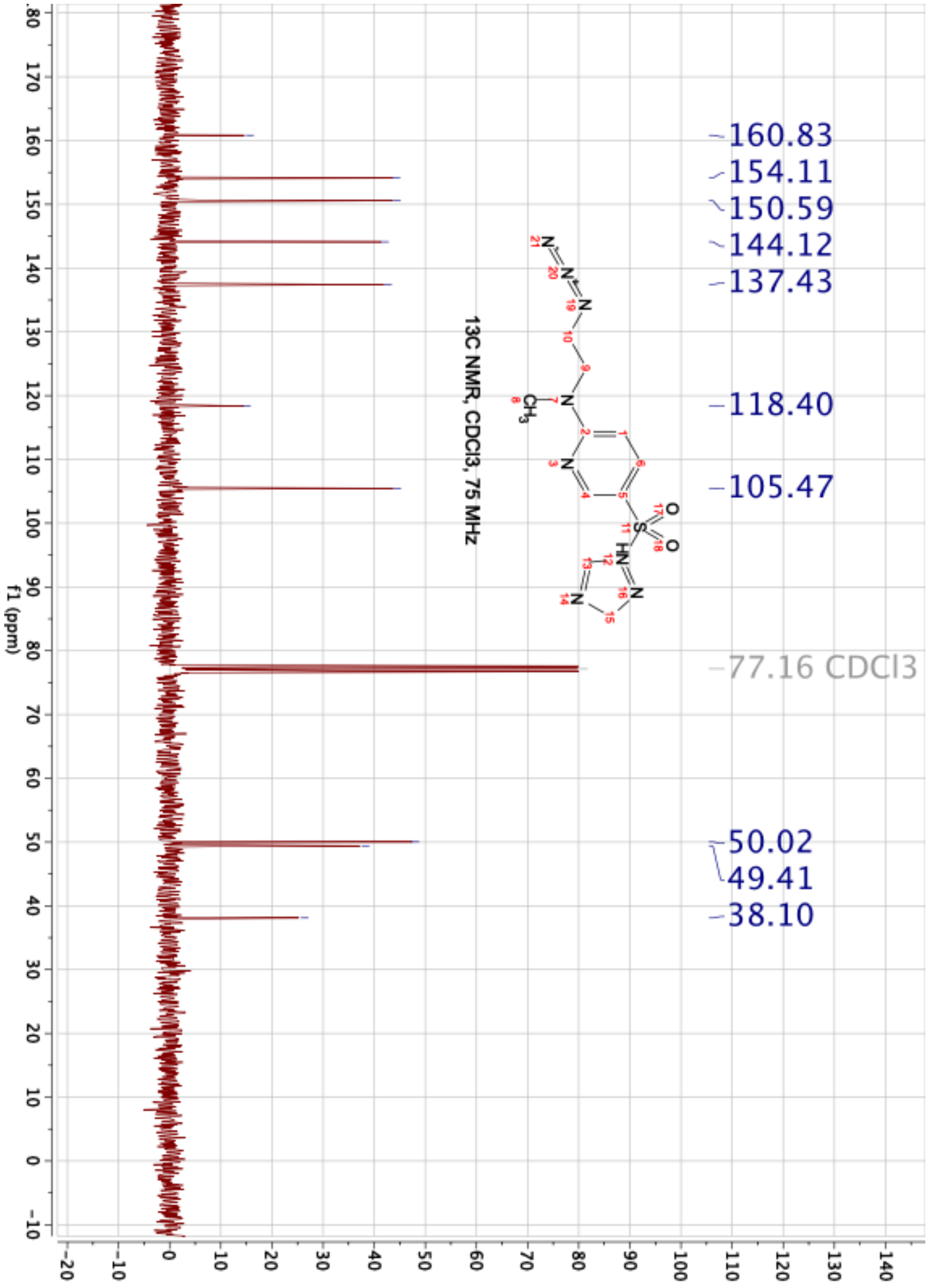
13C NMR, CDCl3, 75 MHz
160.83
154.11
150.59
144.12
137.43
118.40
105.47
77.16 CDCl3
50.02
49.41
38.10
f1 (ppm)

30

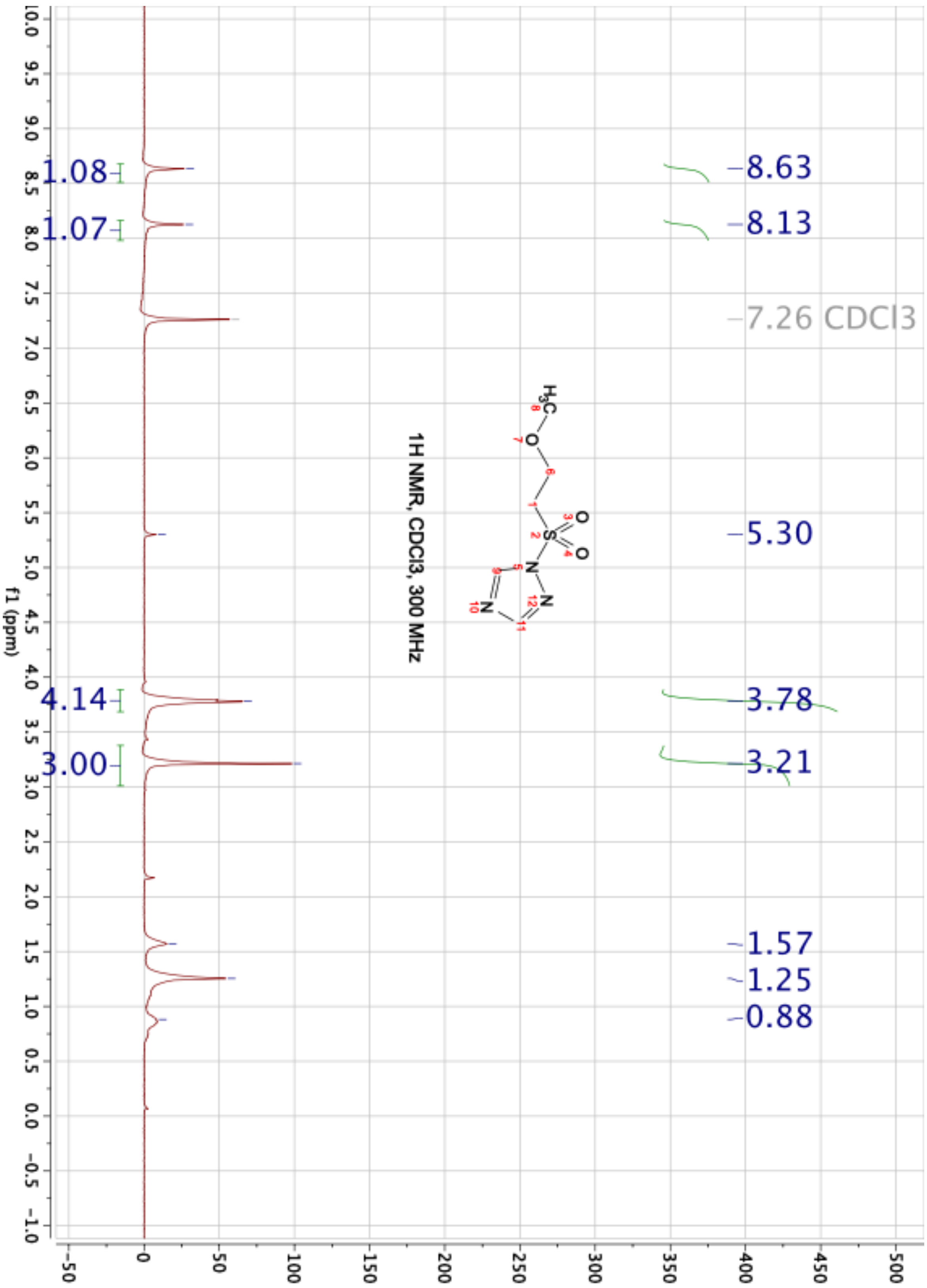
1H NMR, CDCl3, 300 MHz
8.63
8.13
7.26 CDCl3
5.30
3.78
3.21
1.57
1.25
0.88
1.08
1.07
4.14
3.00
f1 (ppm)

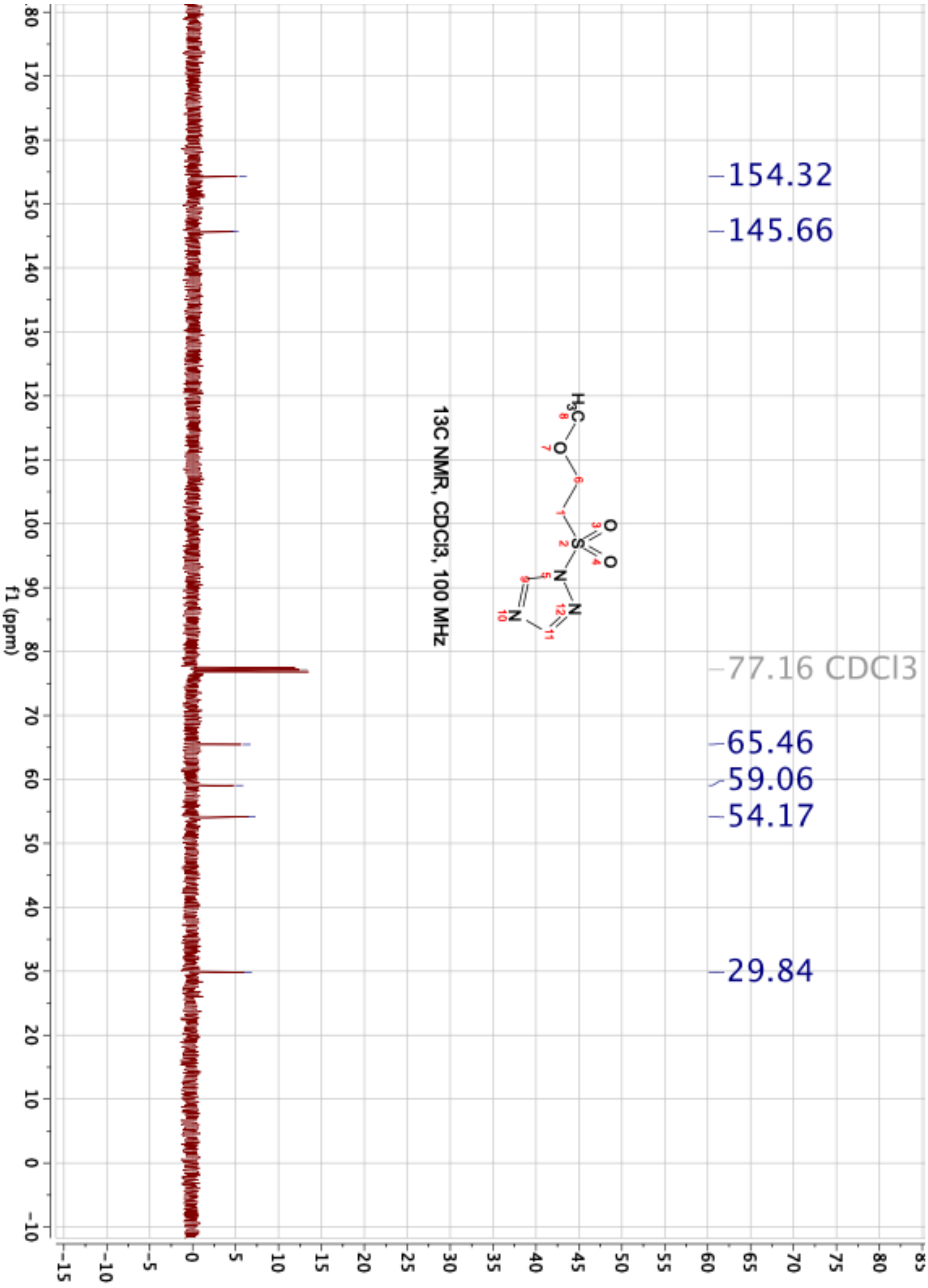
13C NMR, CDCl3, 100 MHz
154.32
145.66
77.16 CDCl3
65.46
59.06
54.17
29.84
f1 (ppm)

31

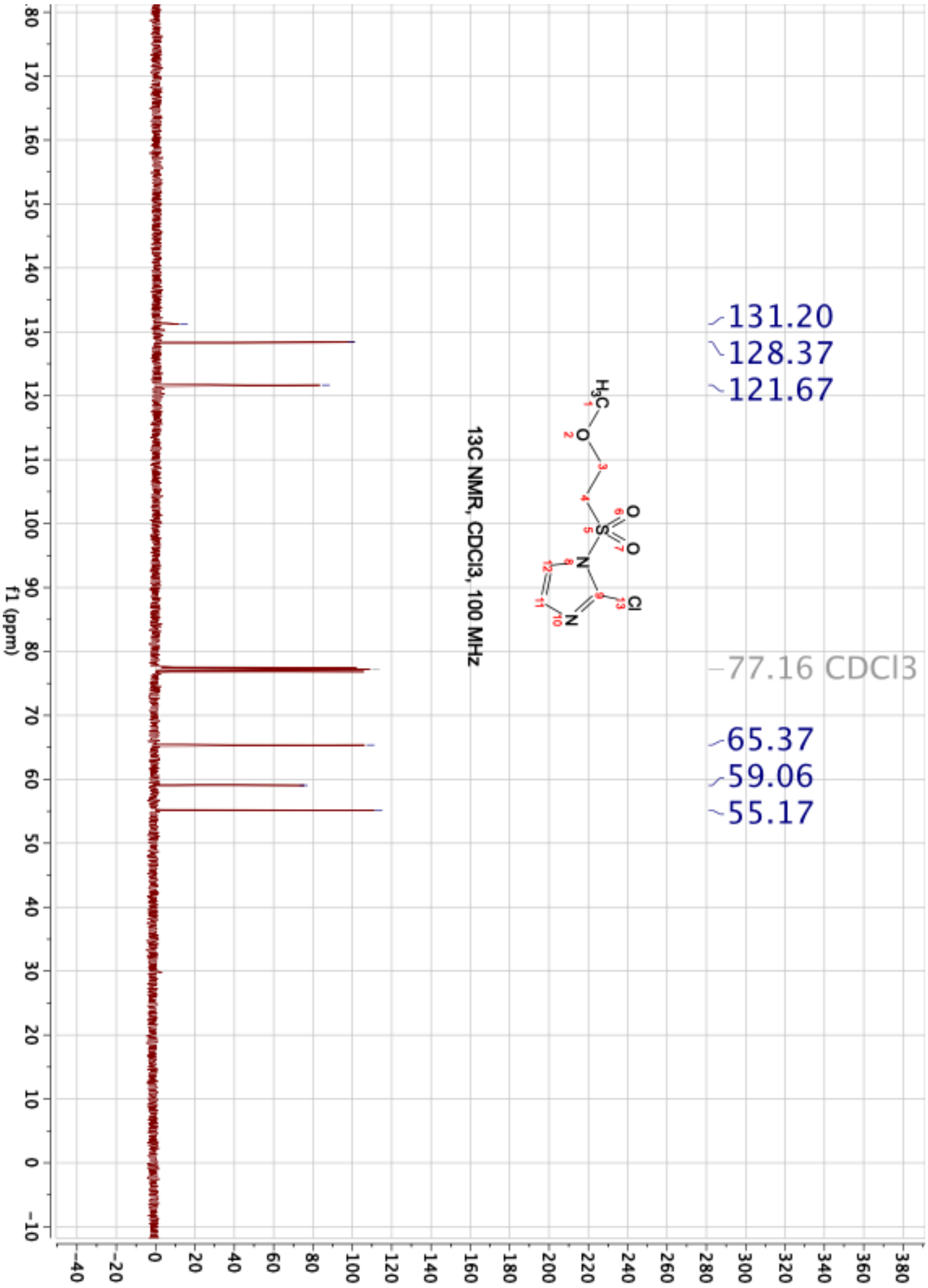
13C NMR, CDCl3, 100 MHz
131.20
128.37
121.67
77.16 CDCl3
65.37
59.06
55.17
f1 (ppm)

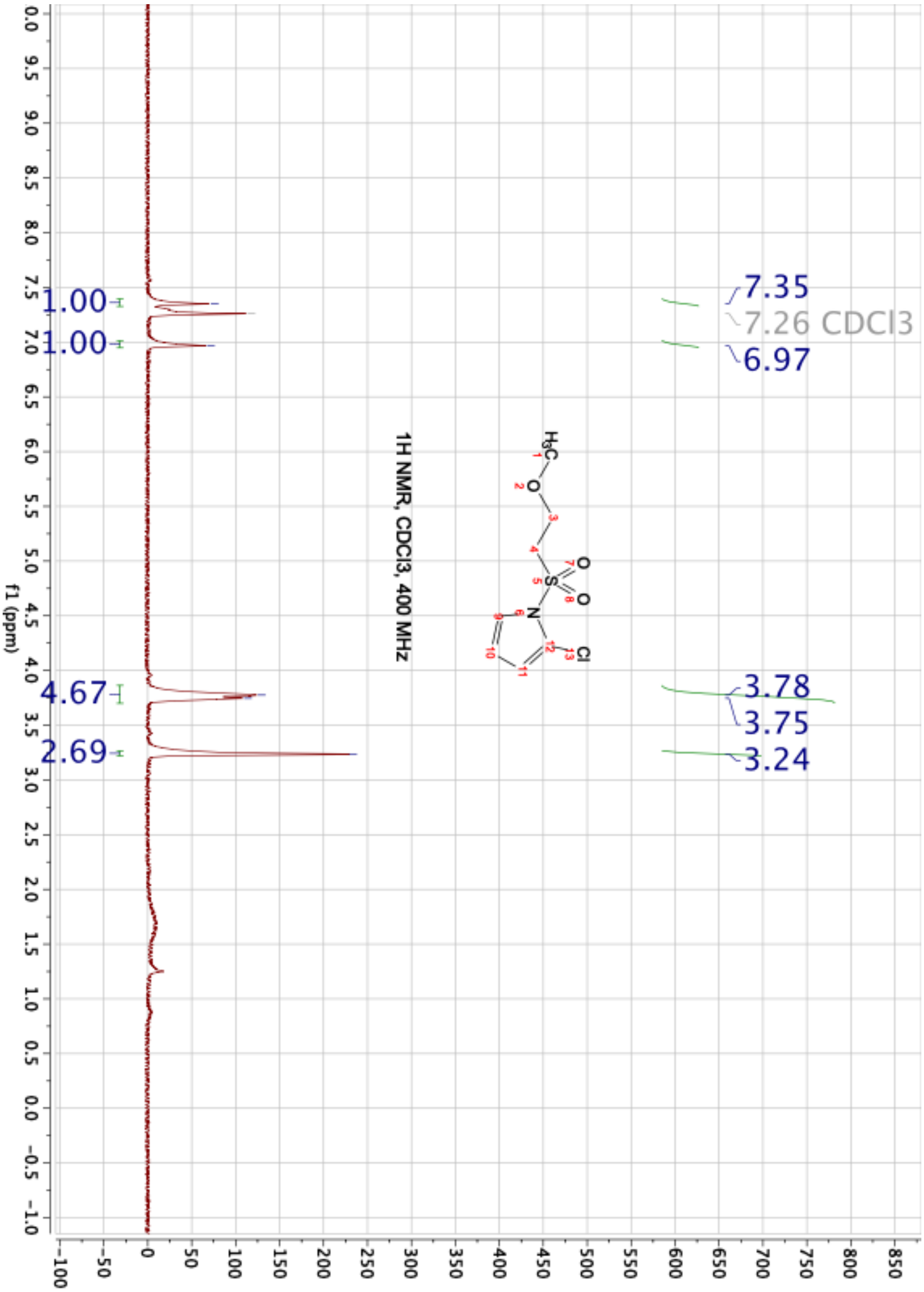
1H NMR, CDCl3, 400 MHz
7.35
7.26 CDCl3
6.97
3.78
3.75
3.24
1.00
1.00
4.67
2.69
f1 (ppm)

32

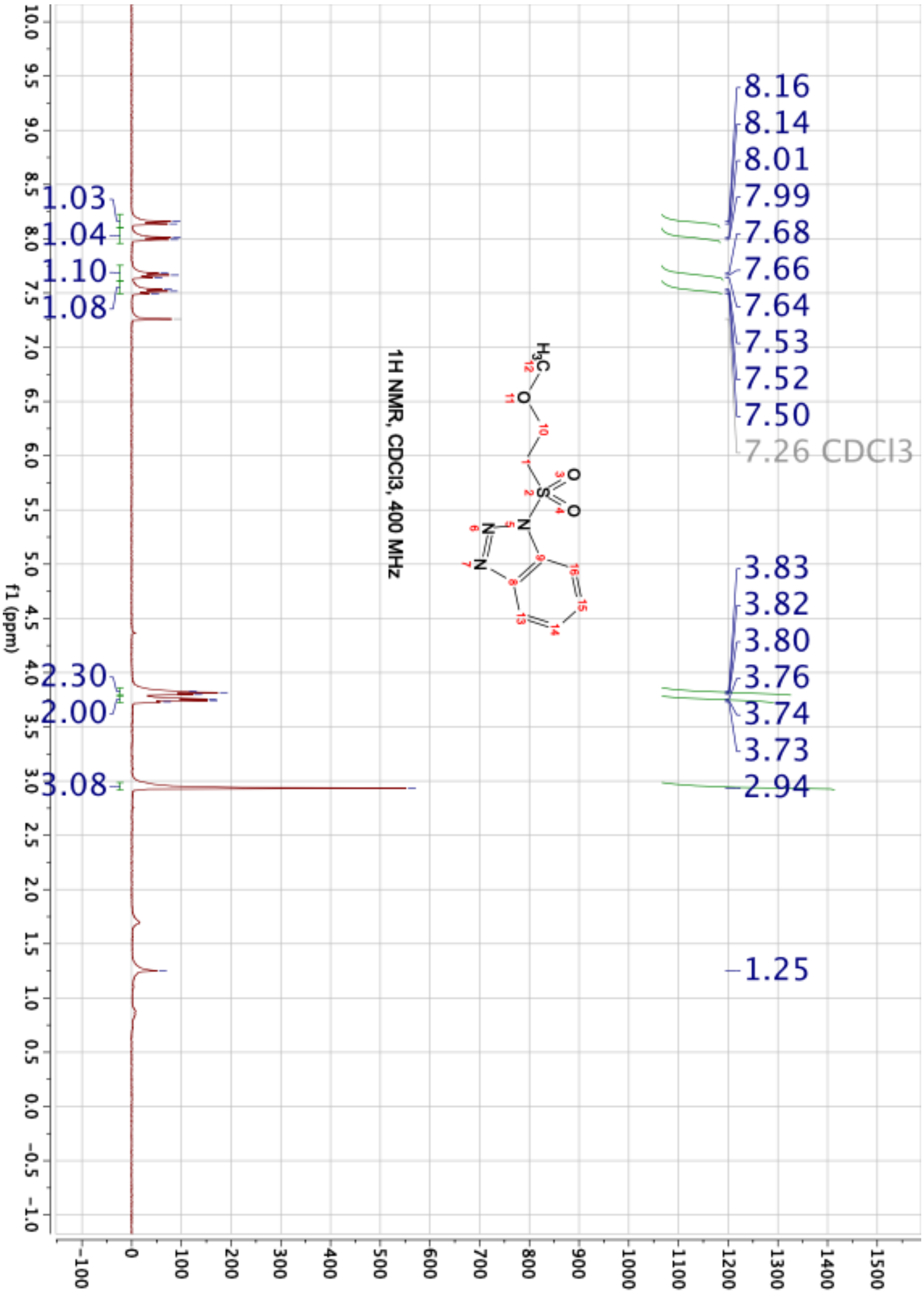
1H NMR, CDCl3, 400 MHz
8.16
8.14
8.01
7.99
7.68
7.66
7.64
7.53
7.52
7.50
7.26 CDCl3
3.83
3.82
3.80
3.76
3.74
3.73
2.94
1.25
1.03
1.04
1.10
1.08
2.30
2.00
3.08
f1 (ppm)

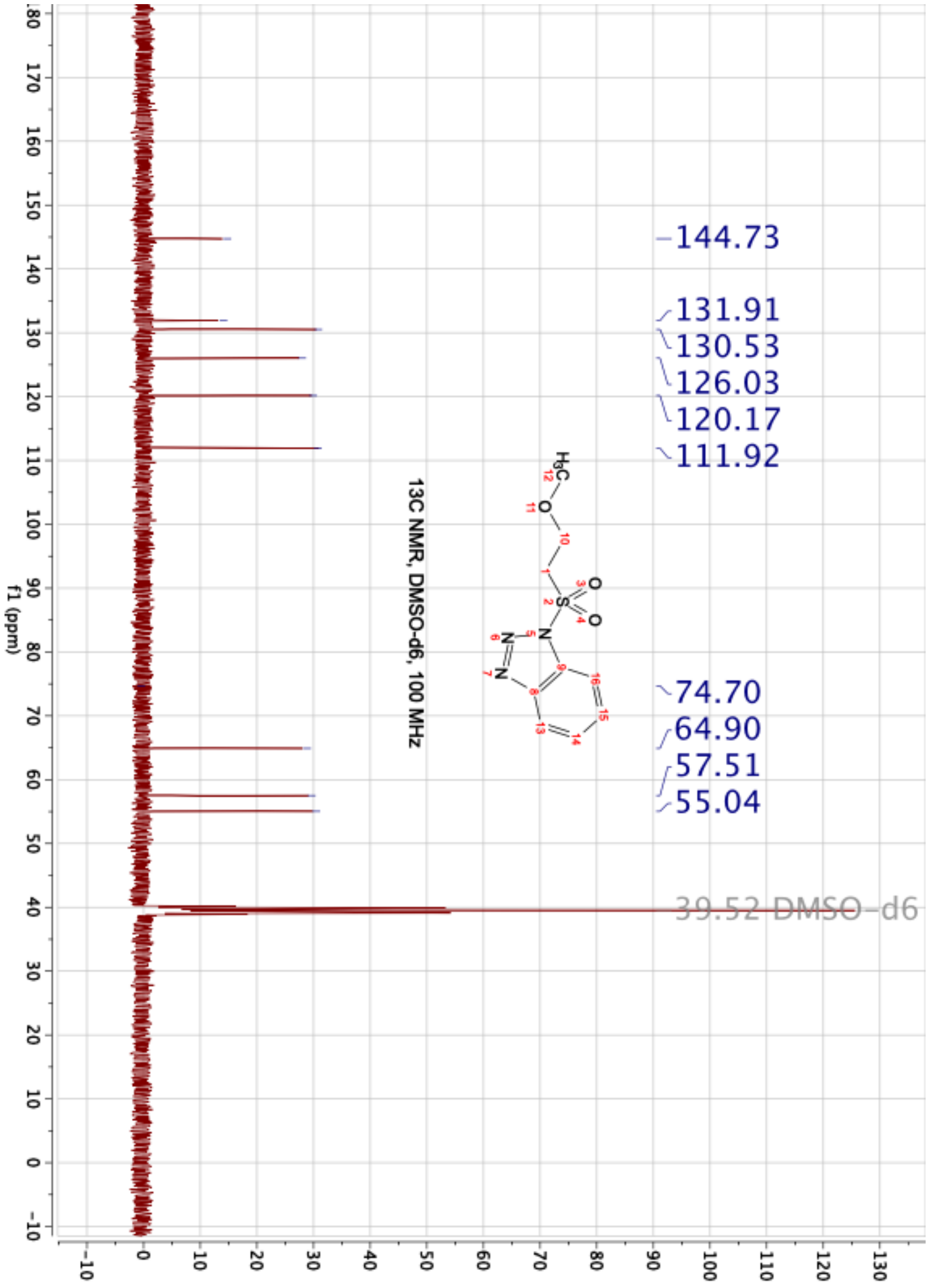
13C NMR, DMSO-d6, 100 MHz
144.73
131.91
130.53
126.03
120.17
111.92
74.70
64.90
57.51
55.04
39.52 DMSO-d6
f1 (ppm)

33

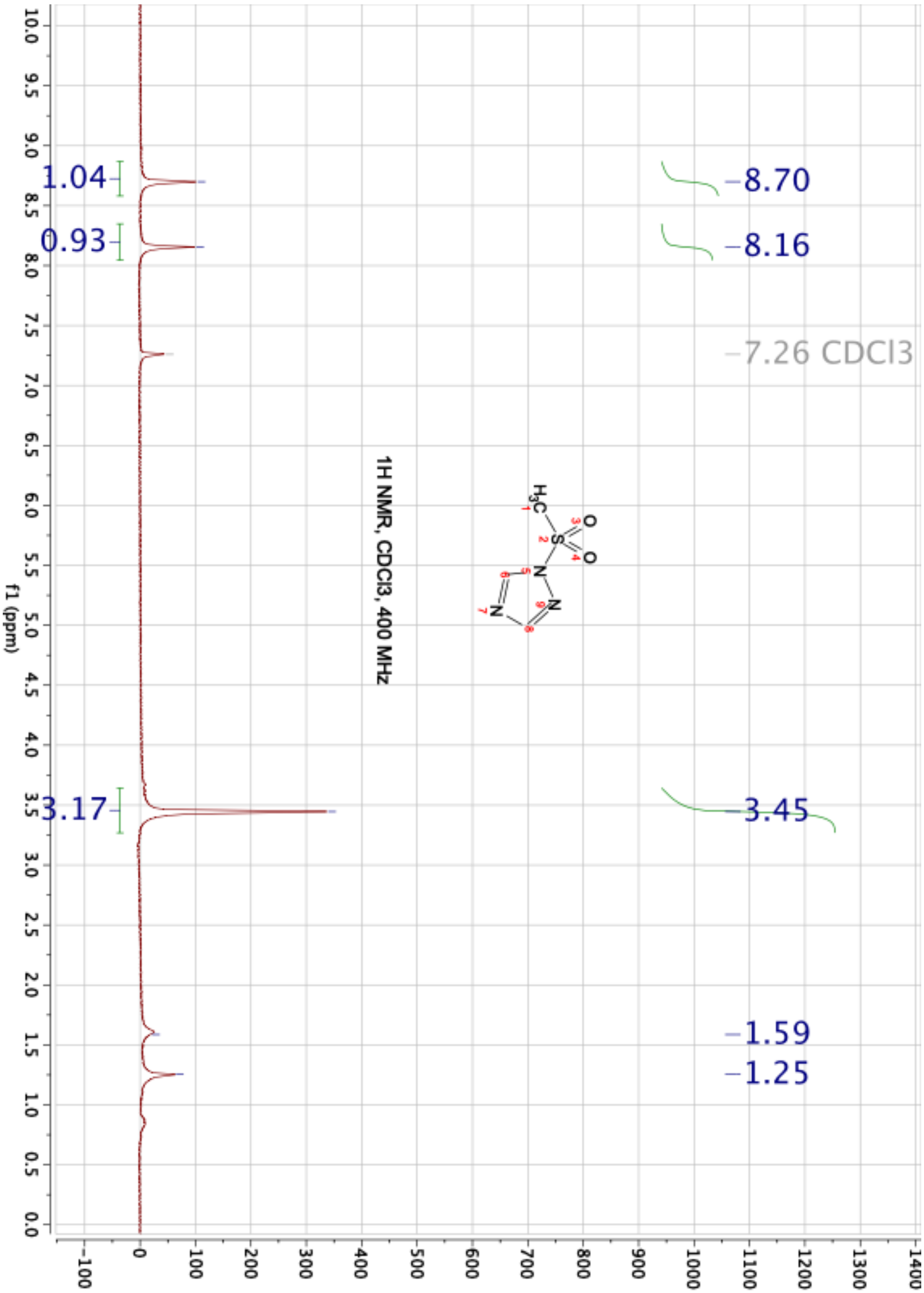
1H NMR, CDCl3, 400 MHz
8.70
8.16
7.26 CDCl3
3.45
1.59
1.25
1.04
0.93
3.17
f1 (ppm)
H3C
O
S
N

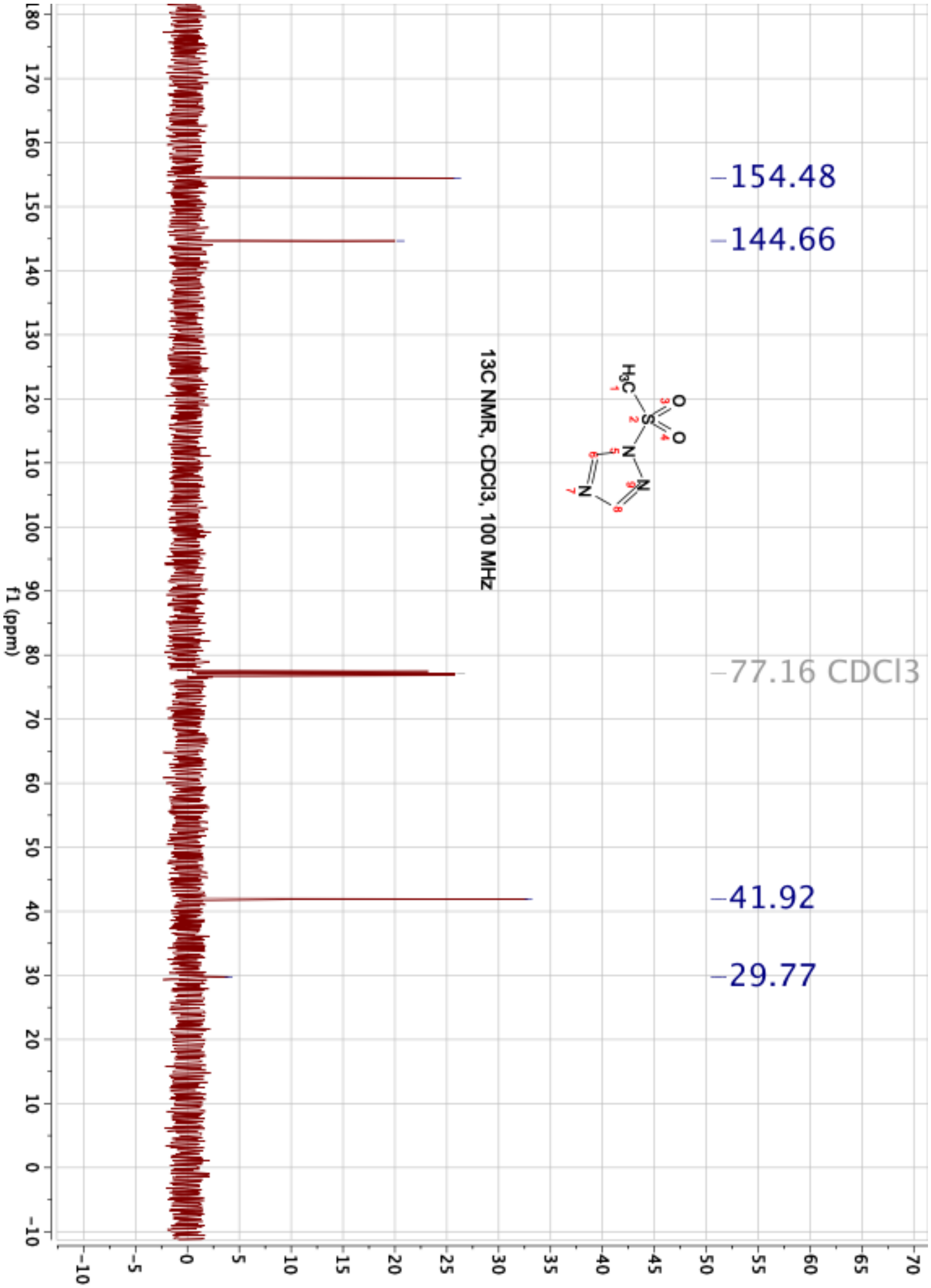
13C NMR, CDCl3, 100 MHz
154.48
144.66
77.16 CDCl3
41.92
29.77
f1 (ppm)
H3C
S
O
O
N
N
N

36

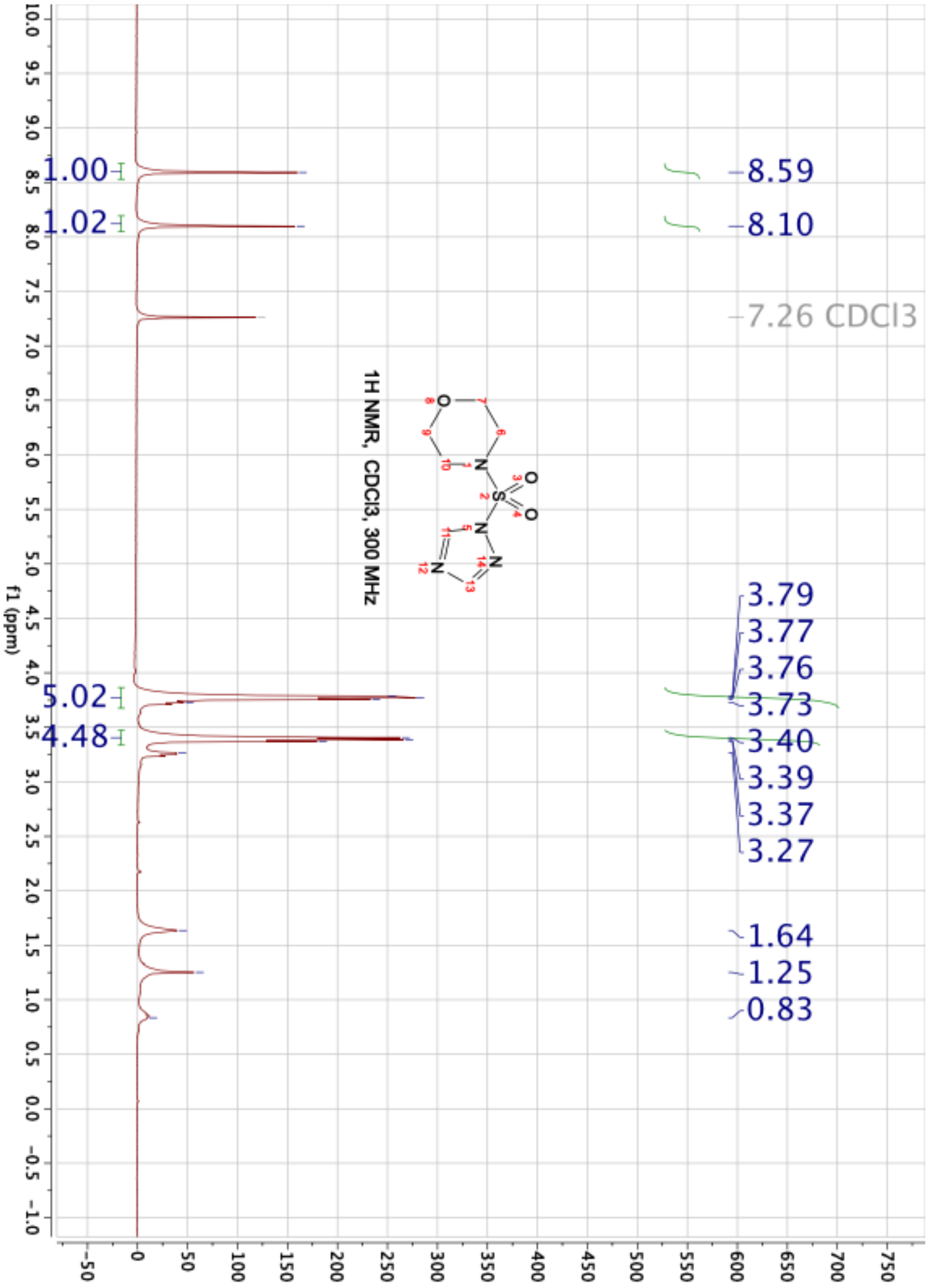
1H NMR, CDCl3, 300 MHz
8.59
8.10
7.26 CDCl3
3.79
3.77
3.76
3.73
3.40
3.39
3.37
3.27
1.64
1.25
0.83
1.00
1.02
5.02
4.48
f1 (ppm)

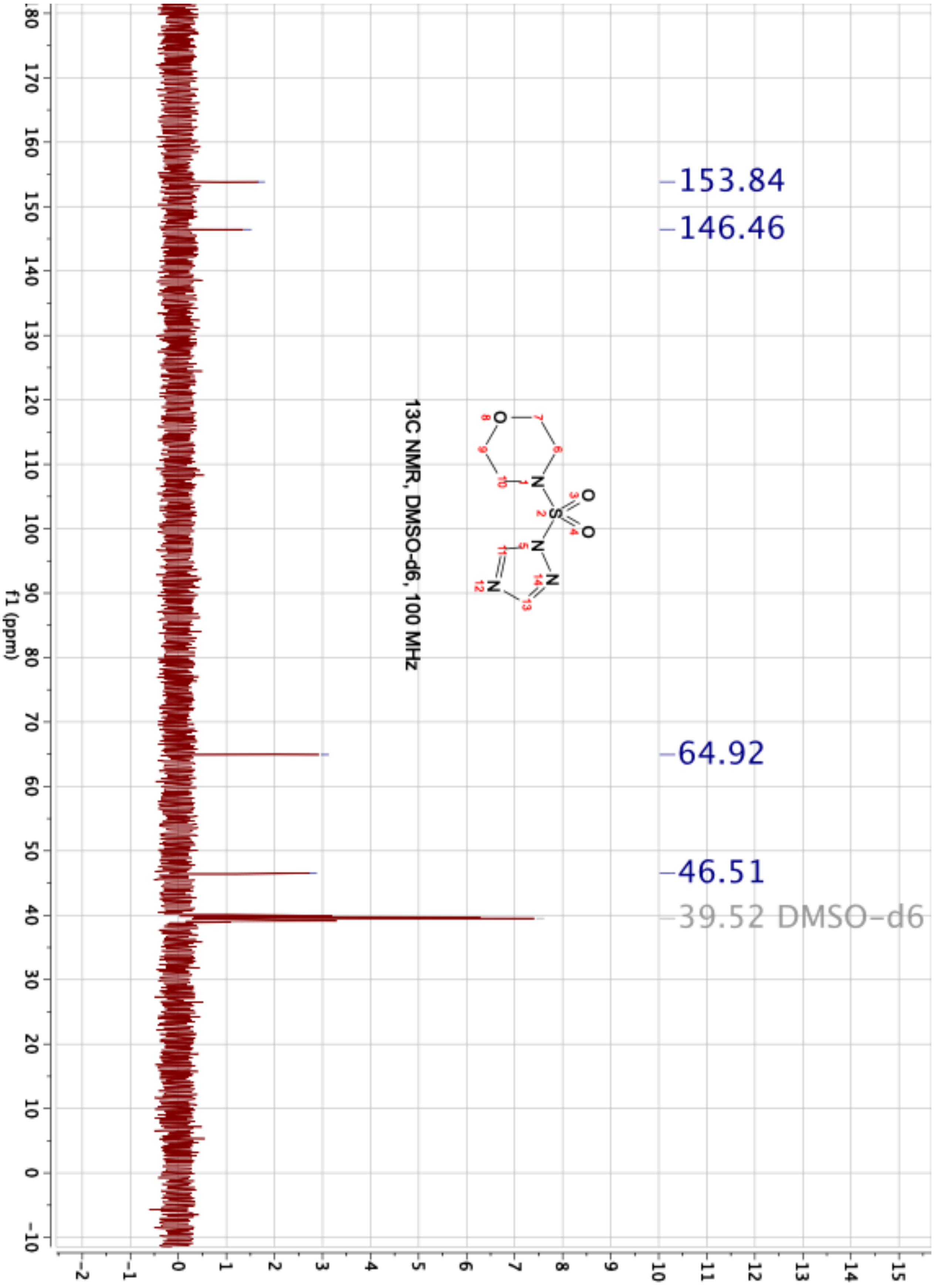
13C NMR, DMSO-d6, 100 MHz
153.84
146.46
64.92
46.51
39.52 DMSO-d6
f1 (ppm)

37

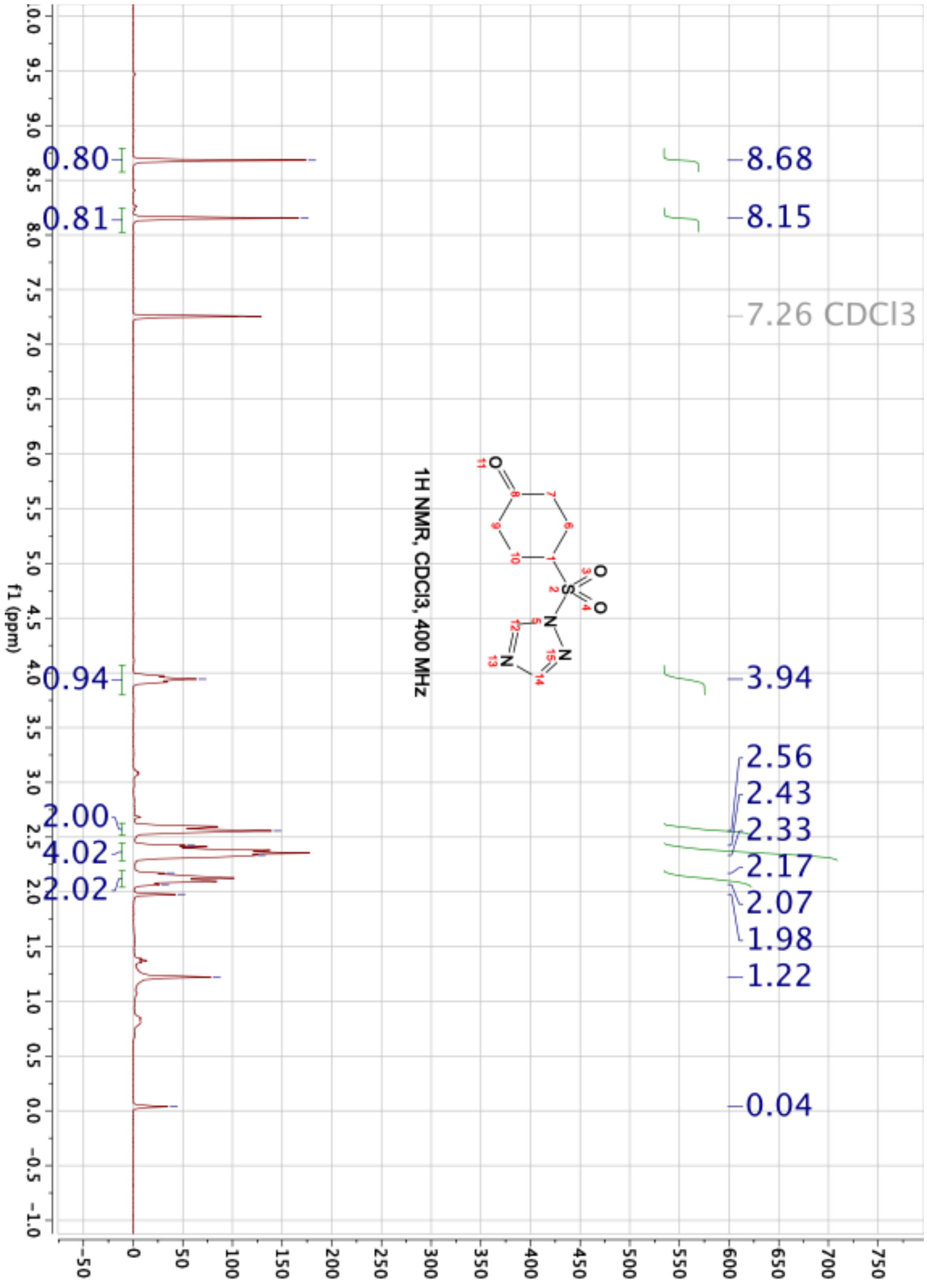
1H NMR, CDCl3, 400 MHz
8.68
8.15
7.26 CDCl3
3.94
2.56
2.43
2.33
2.17
2.07
1.98
1.22
0.04
0.80
0.81
0.94
2.00
4.02
2.02
f1 (ppm)

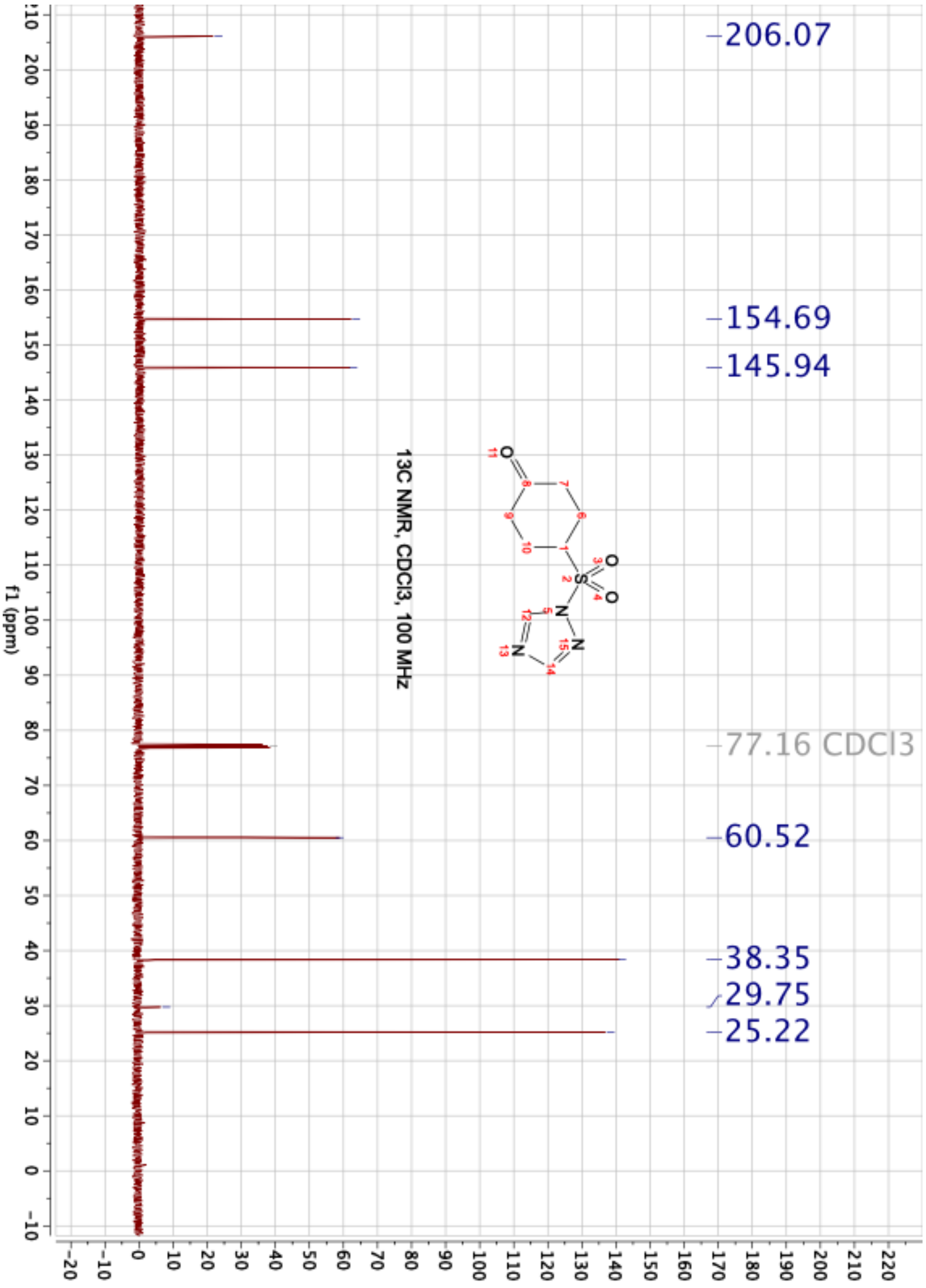
13C NMR, CDCl3, 100 MHz
206.07
154.69
145.94
77.16 CDCl3
60.52
38.35
29.75
25.22
f1 (ppm)

38

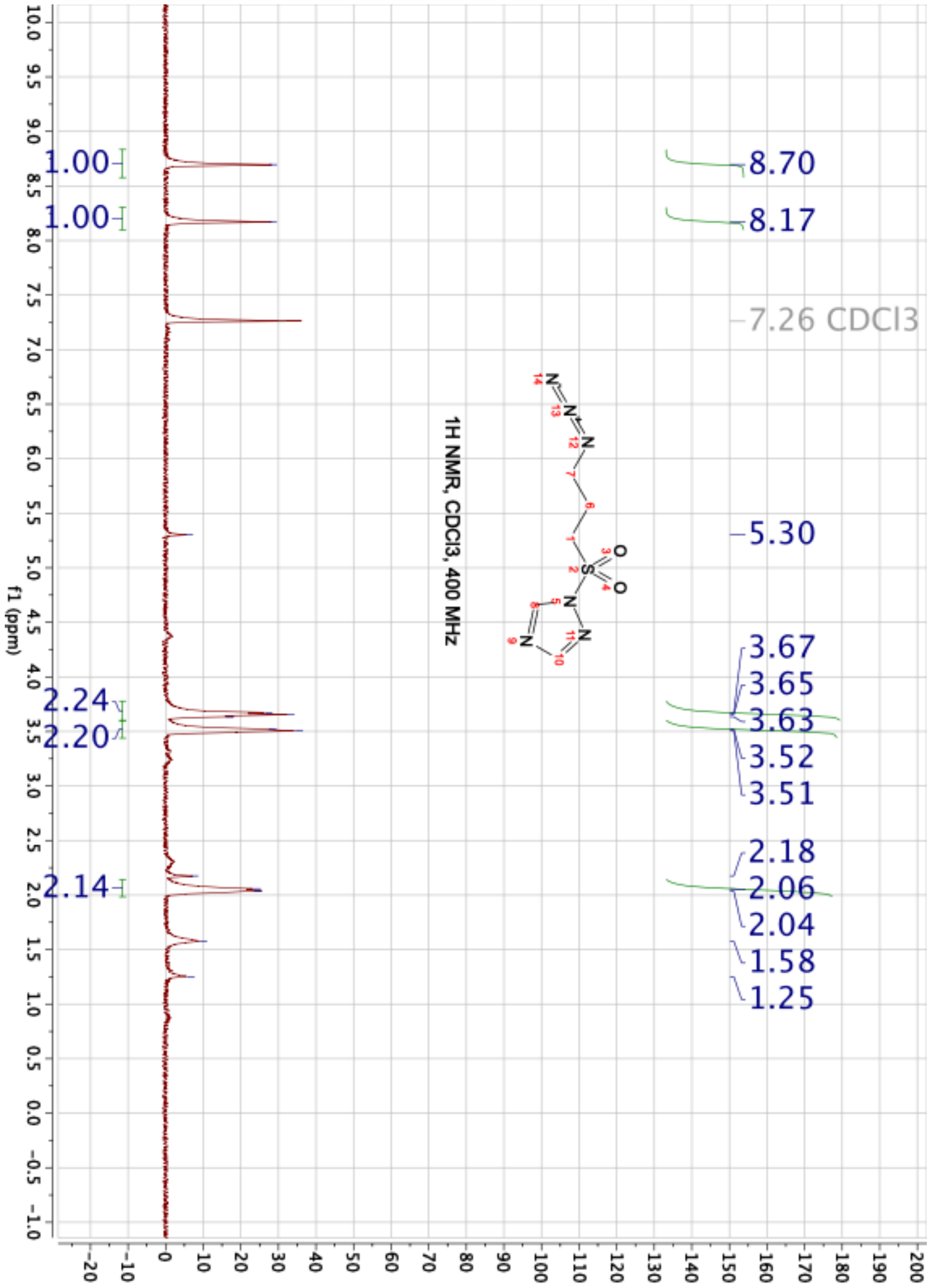
1H NMR, CDCl3, 400 MHz
8.70
8.17
7.26 CDCl3
5.30
3.67
3.65
3.63
3.52
3.51
2.18
2.06
2.04
1.58
1.25
1.00
1.00
2.24
2.20
2.14
f1 (ppm)

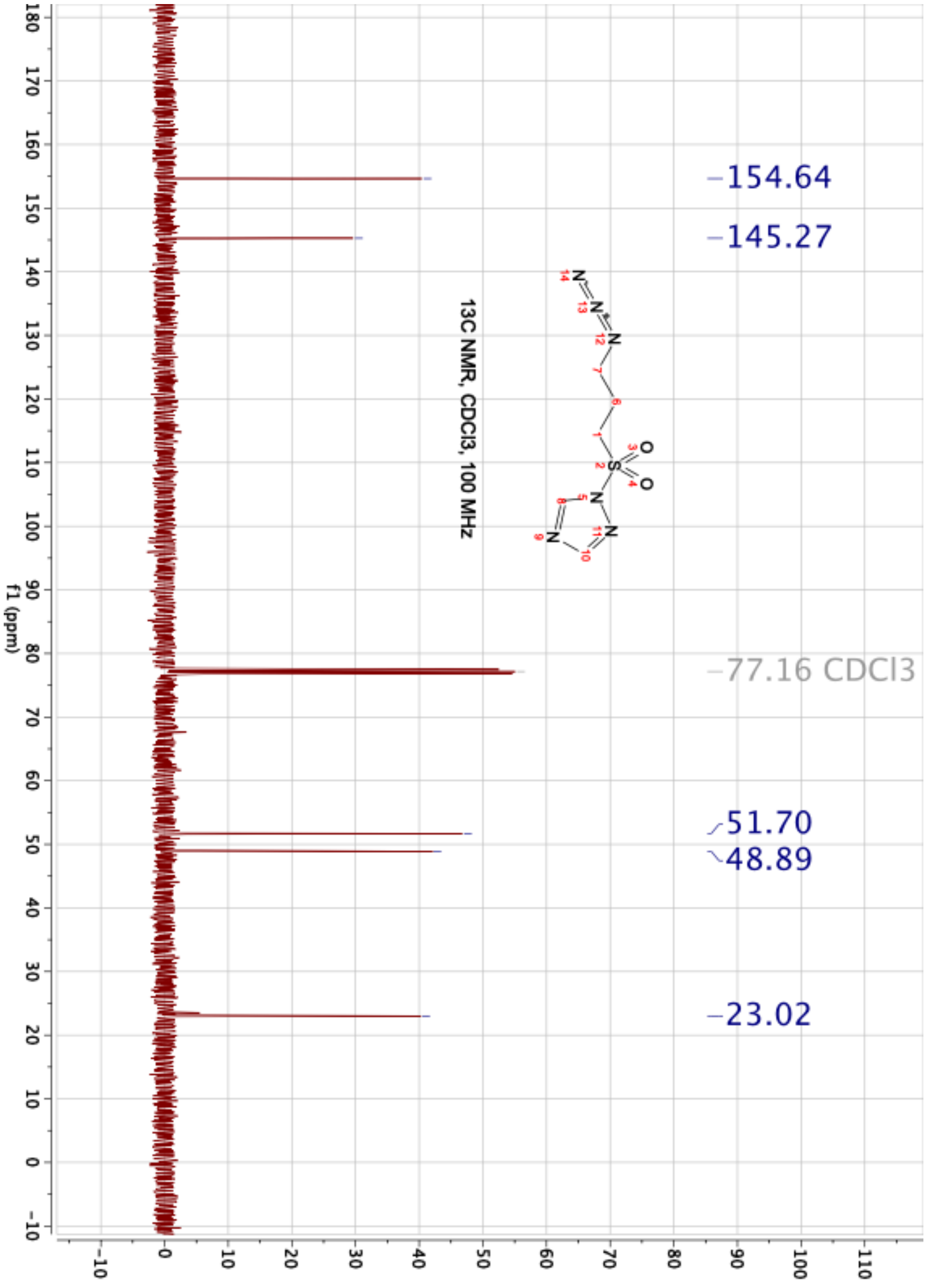
13C NMR, CDCl3, 100 MHz
154.64
145.27
77.16 CDCl3
51.70
48.89
23.02
f1 (ppm)

39

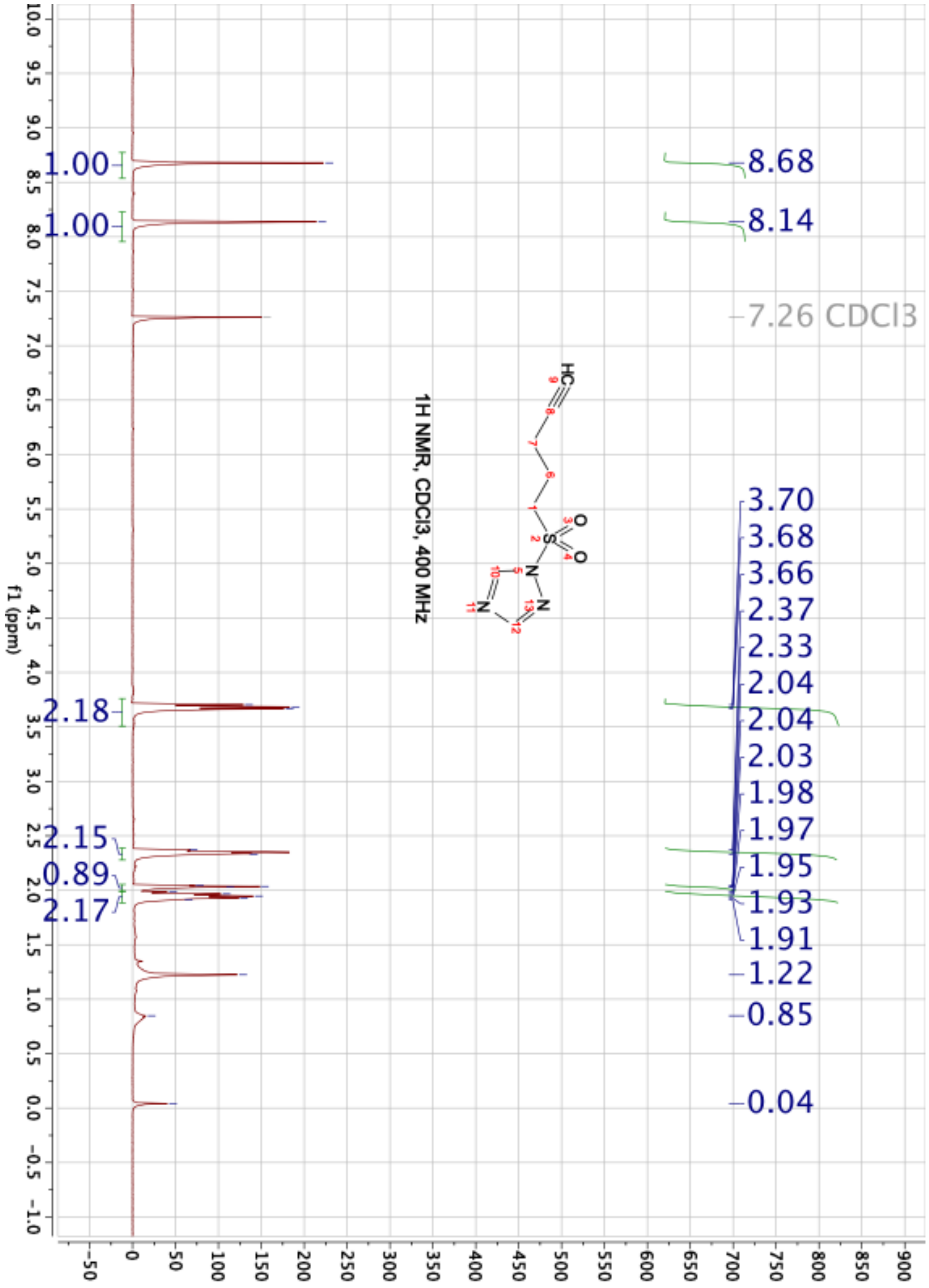
1H NMR, CDCl3, 400 MHz
8.68
8.14
7.26 CDCl3
3.70
3.68
3.66
2.37
2.33
2.04
2.04
2.03
1.98
1.97
1.95
1.93
1.91
1.22
0.85
0.04
1.00
1.00
2.18
2.15
0.89
2.17
f1 (ppm)
HC
O
S
O
N
N
N
N

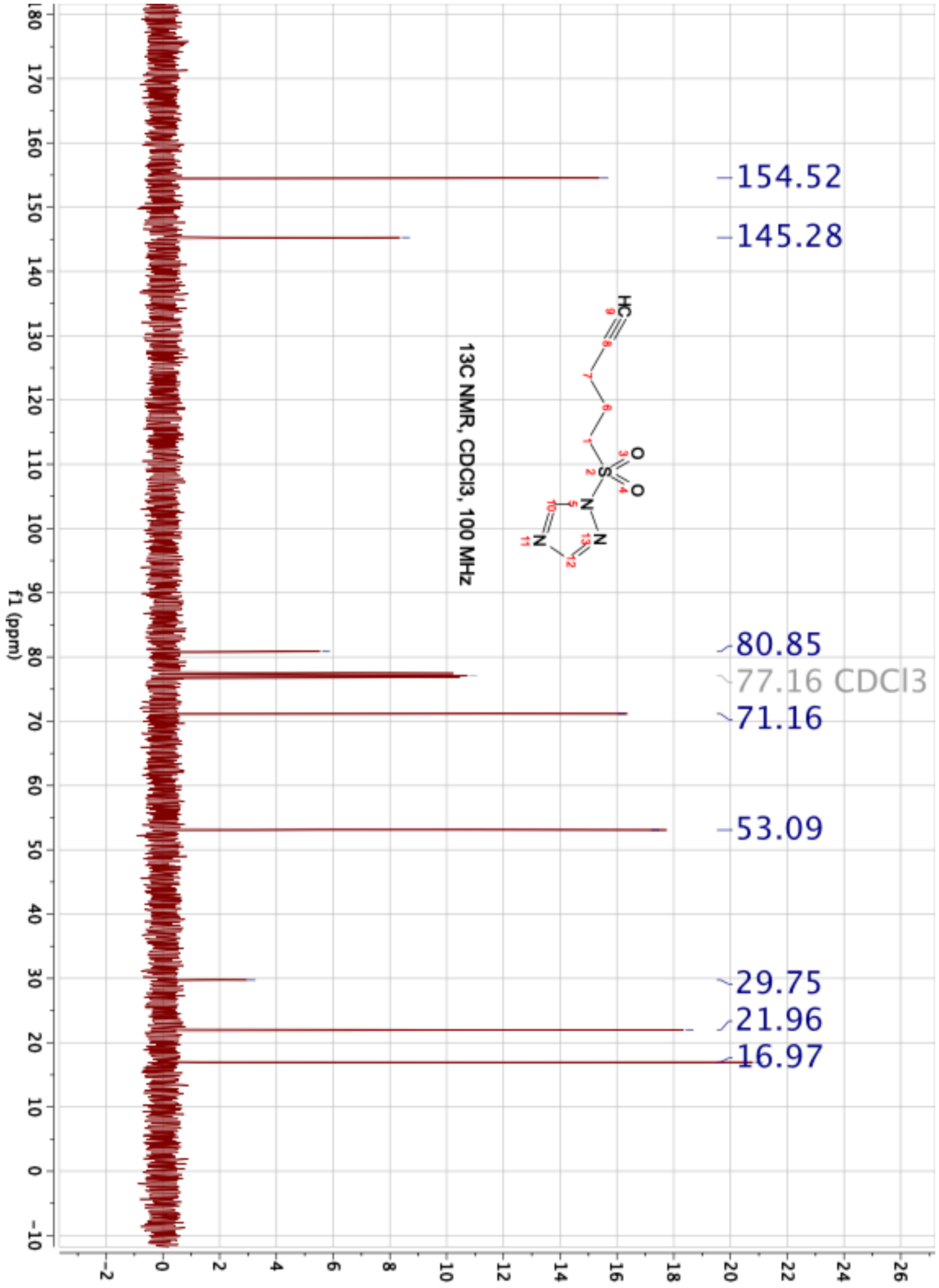
13C NMR, CDCl3, 100 MHz
154.52
145.28
80.85
77.16 CDCl3
71.16
53.09
29.75
21.96
16.97
f1 (ppm)

40

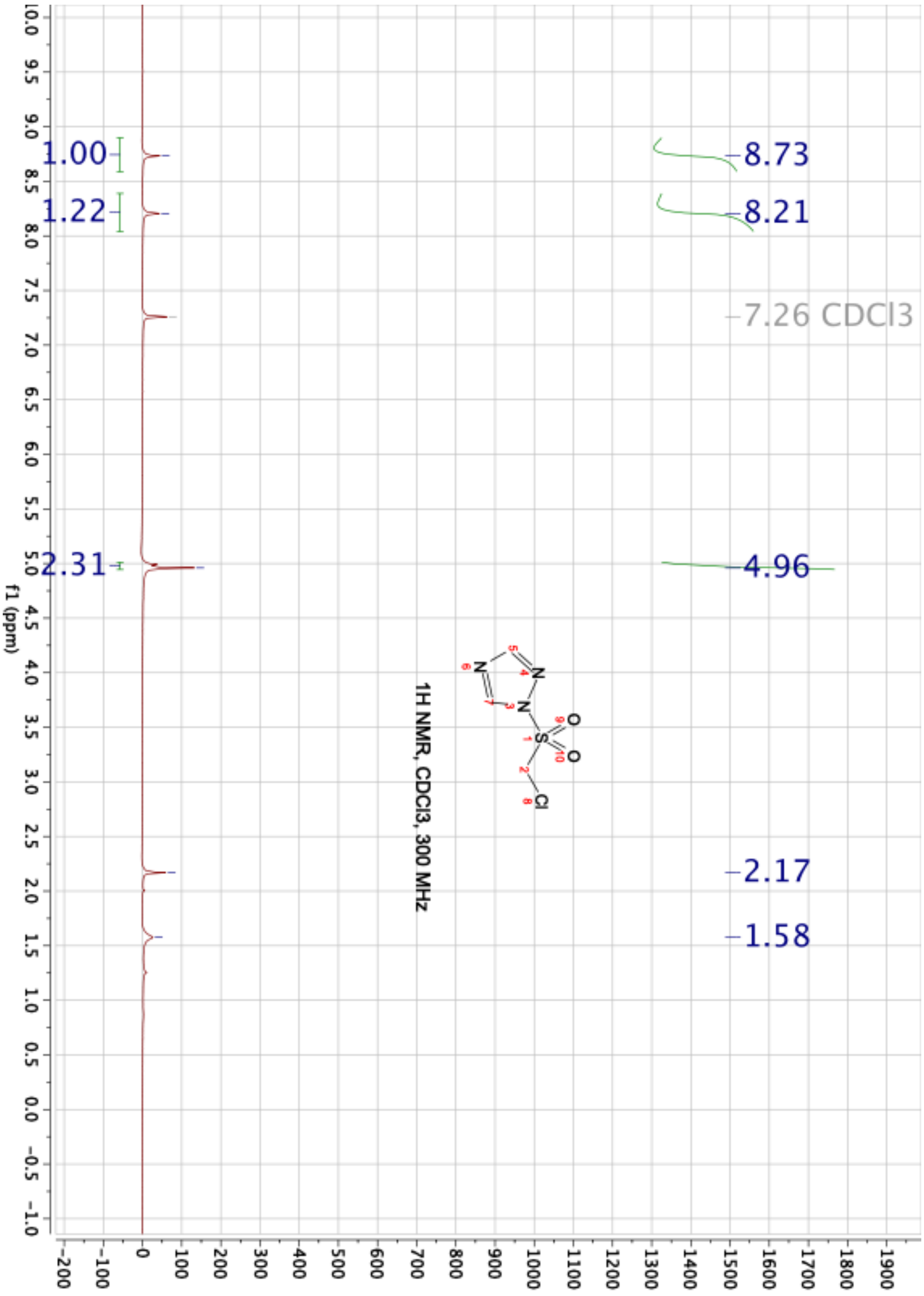
1H NMR, CDCl3, 300 MHz
8.73
8.21
7.26 CDCl3
4.96
2.17
1.58
1.00
1.22
2.31
f1 (ppm)

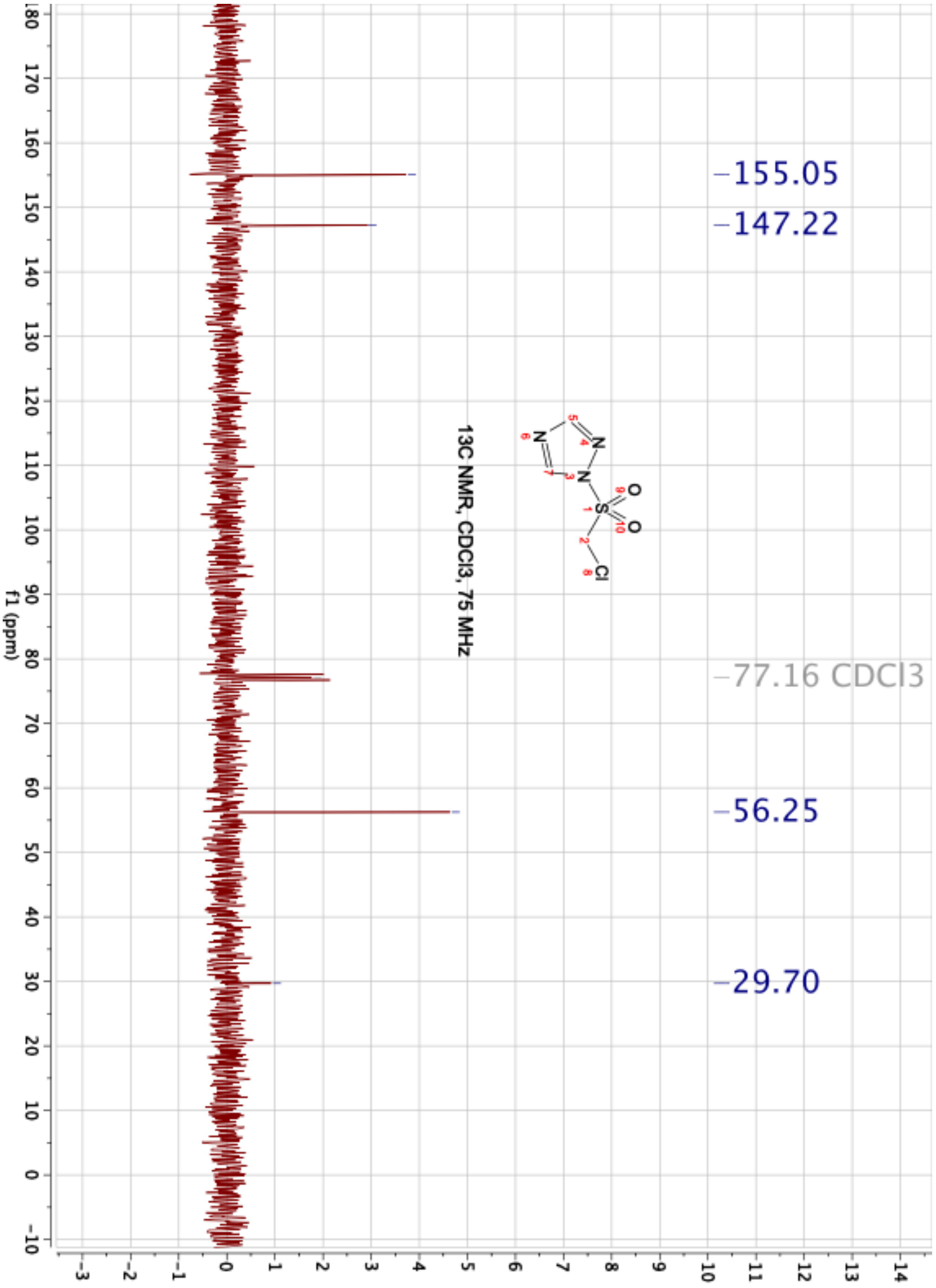

13C NMR, CDCl3, 75 MHz
155.05
147.22
77.16 CDCl3
56.25
29.70
f1 (ppm)

References

(1) Kerpedjiev, P.; Hammer, S.; Hofacker, I. L. Forna (Force-Directed RNA): Simple and Effective Online RNA Secondary Structure Diagrams. *Bioinformatics* **2015**, *31* (20), 3377–3379. https://doi.org/10.1093/BIOINFORMATICS/BTV372.

(2) Spitale, R. C.; Crisalli, P.; Flynn, R. A.; Torre, E. A.; Kool, E. T.; Chang, H. Y. RNA SHAPE Analysis in Living Cells. *Nat. Chem. Biol. 2012 91* **2012**, *9* (1), 18–20. https://doi.org/10.1038/nchembio.1131.

(3) Spitale, R. C.; Flynn, R. A.; Zhang, Q. C.; Crisalli, P.; Lee, B.; Jung, J. W.; Kuchelmeister, H. Y.; Batista, P. J.; Torre, E. A.; Kool, E. T.; Chang, H. Y. Structural Imprints in Vivo Decode RNA Regulatory Mechanisms. *Nat. 2015 5197544* **2015**, *519* (7544), 486–490. https://doi.org/10.1038/nature14263.

(4) Song, X.; He, Y.; Wang, B.; Peng, S.; Pan, X.; Wei, M.; Liu, Q.; Qin, H. L.; Tang, H. Synthesis of Aryl Sulfonyl Fluorides from Aryl Sulfonyl Chlorides Using Sulfuryl Fluoride (SO2F2) as Fluoride Provider. *Tetrahedron* **2022**, *108*, 132657. https://doi.org/10.1016/J.TET.2022.132657.

(5) Toyo'oka, T.; Imai, K. New Fluorogenic Reagent Having Halogenobenzofurazan Structure for Thiols: 4-(Aminosulfonyl)-7-Fluoro-2,1,3-Benzoxadiazole. *Anal. Chem.* **1984**, *56* (13), 2461–2464. https://doi.org/10.1021/AC00277A044/ASSET/AC00277A044.FP.PNG_V03.

(6) Pan, S.; Jang, S. Y.; Liew, S. S.; Fu, J.; Wang, D.; Lee, J. S.; Yao, S. Q. A Vinyl Sulfone-Based Fluorogenic Probe Capable of Selective Labeling of PHGDH in Live Mammalian Cells. *Angew. Chemie Int. Ed.* **2018**, *57* (2), 579–583. https://doi.org/10.1002/ANIE.201710856.

(7) Rasale, D.; Patil, K.; Sauter, B.; Geigle, S.; Zhanybekova, S.; Gillingham, D. A New Water Soluble Copper N-Heterocyclic Carbene Complex Delivers Mild O 6 G-Selective RNA Alkylation. *Chem. Commun.* **2018**, *54* (66), 9174–9177. https://doi.org/10.1039/C8CC04476G.

(8) Giovannuzzi, S.; D'ambrosio, M.; Luceri, C.; Osman, S. M.; Pallecchi, M.; Bartolucci, G.; Nocentini, A.; Supuran, C. T. Aromatic Sulfonamides Including a Sulfonic Acid Tail: New Membrane Impermeant Carbonic Anhydrase Inhibitors for Targeting Selectively the Cancer-Associated Isoforms. *Int. J. Mol. Sci. 2022, Vol. 23, Page 461* **2021**, *23* (1), 461. https://doi.org/10.3390/IJMS23010461.
